# Supplementary material for: Interaction of 7SK with the Smn complex modulates snRNP production
Source: Nat Commun. 2021 Feb 24;12:1278. doi: 10.1038/s41467-021-21529-1 (PMC7904863; doi:10.1038/s41467-021-21529-1)
Supplement: Supplementary file 1 — Supplementary Information [file 41467_2021_21529_MOESM1_ESM.pdf]

SUPPLEMENTARY INFORMATION

Interaction of 7SK with the Smn complex modulates snRNP production

Changhe Ji, Jakob Bader, Pradhipa Ramanathan, Luisa Hennlein, Felix Meissner, Sibylle Jablonka, Matthias Mann, Utz Fischer, Michael Sendtner and Michael Briese

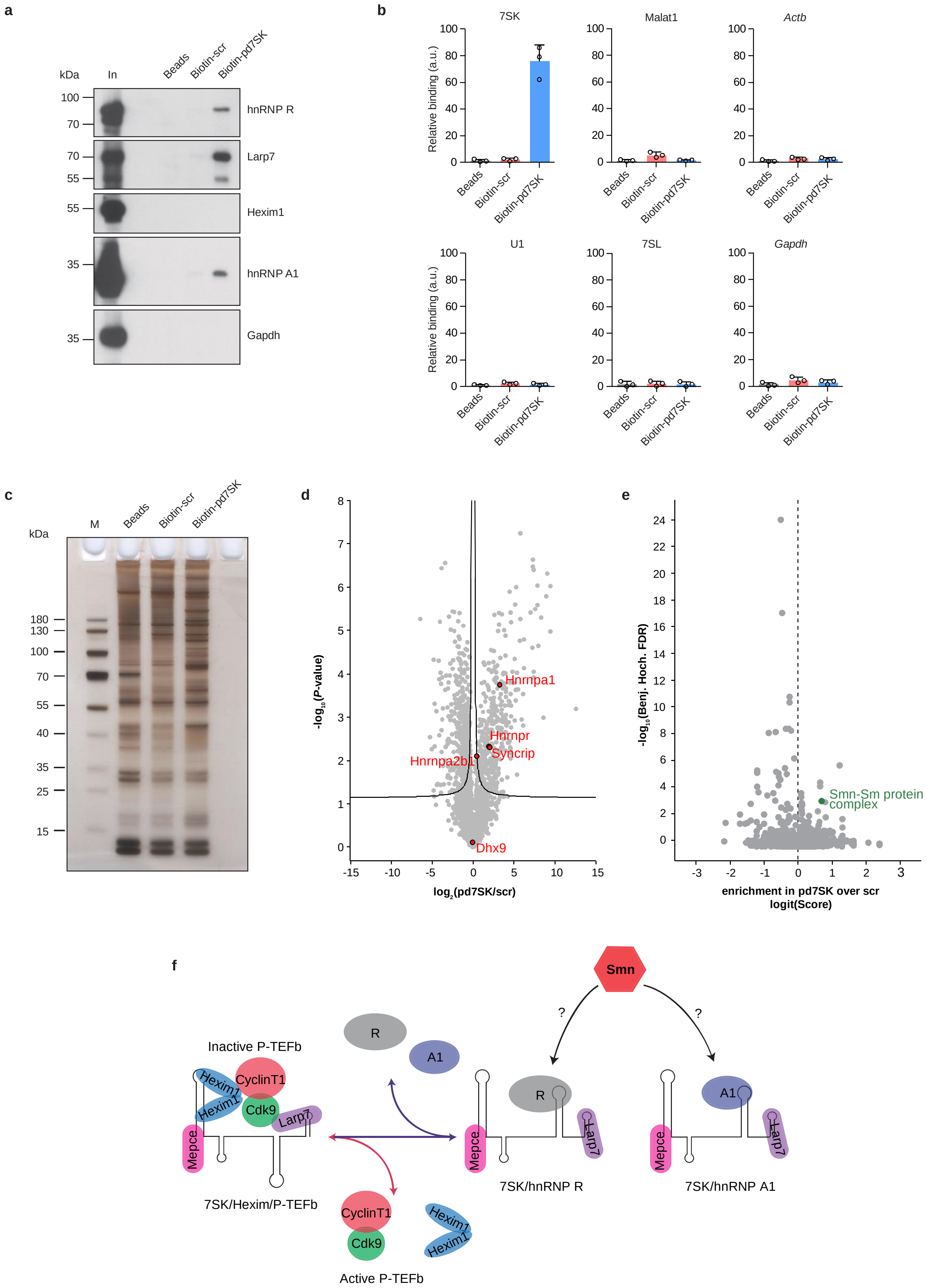
a
kDa
In
Beads
Biotin-scr
Biotin-pd7SK
hnRNP R
Larp7
Hexim1
hnRNP A1
Gapdh
b
7SK
Malat1
Actb
U1
7SL
Gapdh
Relative binding (a.u.)
c
M
kDa
d
-log10(P-value)
log2(pd7SK/scr)
Hnrnpa1
Hnrnpr
Syncrip
Hnrnpa2b1
Dhx9
e
-log10(Benj. Hoch. FDR)
enrichment in pd7SK over scr
logit(Score)
Smn-Sm protein complex
f
Smn
Inactive P-TEFb
CyclinT1
Hexim1
Cdk9
Larp7
Mepce
7SK/Hexim/P-TEFb
R
A1
Active P-TEFb
7SK/hnRNP R
7SK/hnRNP A1

Supplementary Fig. 1 Proteomic analysis of 7SK-interacting proteins reveals Smn as a novel component of 7SK complexes. a Western blot analysis of proteins co-precipitated by Biotin-pd7SK, Biotin-scr or beads from NSC-34 cells. **b** qPCR analysis of RNAs co-precipitated by Biotin-pd7SK, Biotin-scr or beads from NSC-34 cells. Data are mean ± s.d. (*n*=3). **c** Analysis of proteins co-precipitated by Biotin-pd7SK, Biotin-scr or beads from NSC-34 cells by SDS-PAGE and silver staining. **d** Volcano plot of the 7SK interaction proteome following pulldown with Biotin-pd7SK and scr control from NSC-34 cell lysate. The proteins hnRNP A1, A2/B1, Q (Syncrip), R and RNA helicase A (Dhx9) are labeled. **e** Gene ontology annotation term analysis of proteins enriched by Biotin-pd7SK relative to Biotin-scr pulldown. Plot depicts strength of enrichment shown by logit value of 1D enrichment score (x axis) vs statistical significance of enrichment shown by $-\log_{10}$ of Benjamini-Hochberg-adjusted *P*-value (y axis). **f** Model showing different 7SK subcomplexes and indicating the interaction of Smn with 7SK/hnRNP complexes. Source data are provided as a Source Data file.

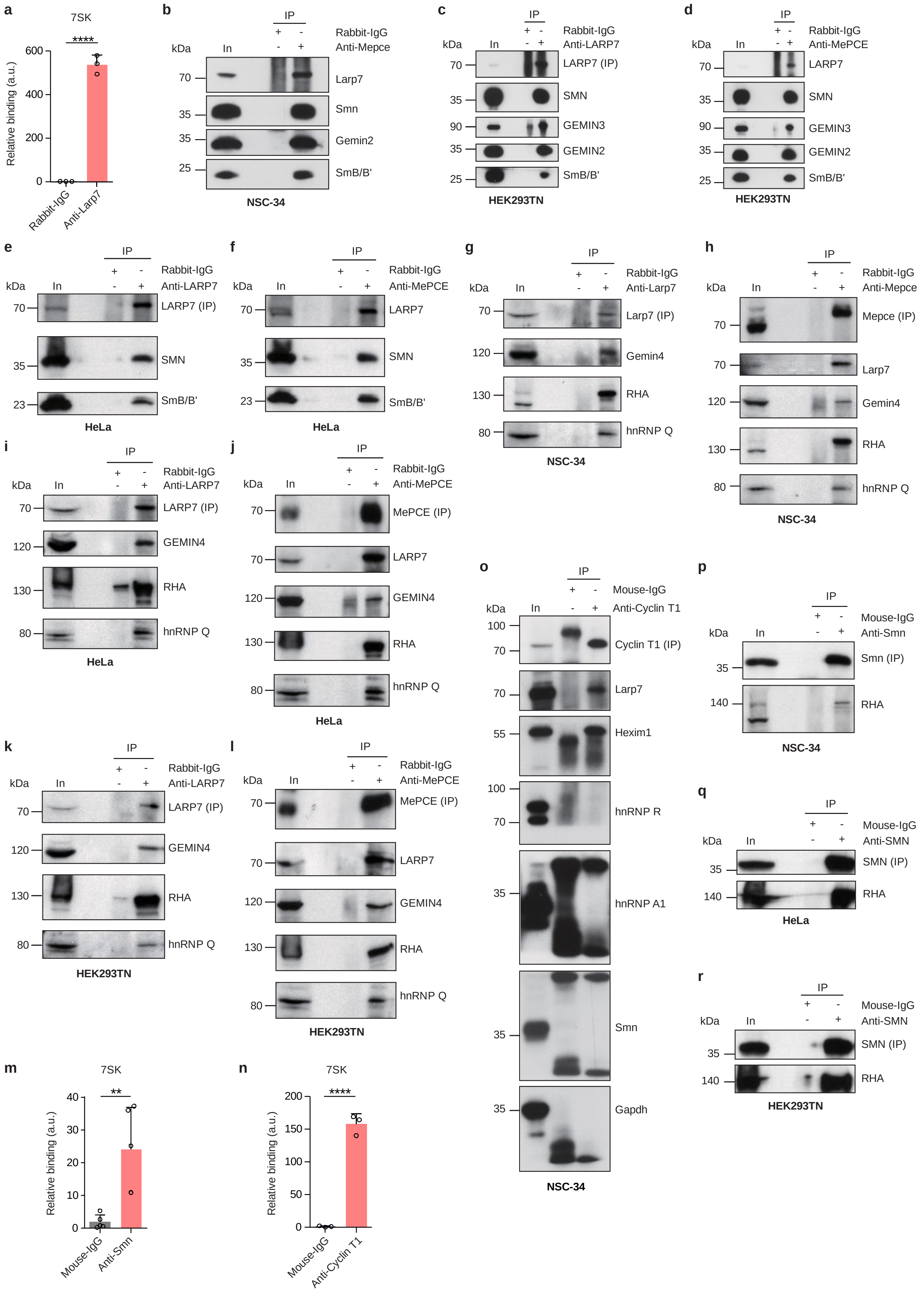
a
7SK
****
Relative binding (a.u.)
Rabbit-IgG
Anti-Larp7
b
IP
Rabbit-IgG
Anti-Mepce
kDa
In
Larp7
Smn
Gemin2
SmB/B'
NSC-34
c
Anti-LARP7
LARP7 (IP)
SMN
GEMIN3
GEMIN2
HEK293TN
d
Anti-MePCE
LARP7
HeLa
Gemin4
RHA
hnRNP Q
Mepce (IP)
MePCE (IP)
GEMIN4
m
Mouse-IgG
Anti-Smn
n
Anti-Cyclin T1
Cyclin T1 (IP)
Hexim1
hnRNP R
hnRNP A1
Gapdh
Smn (IP)
Anti-SMN
SMN (IP)

Supplementary Fig. 2 Smn interacts with 7SK/hnRNP complexes. a qPCR analysis of 7SK co-precipitated by anti-Larp7 from NSC-34 cells. Data are mean ± s.d.; ****$P \leq 0.0001$; unpaired two-tailed t-test (n=3). **b** Co-immunoprecipitation of proteins by anti-Mepce from NSC-34 cells. **c** Co-immunoprecipitation of proteins by anti-LARP7 from HEK293TN cells. **d** Co-immunoprecipitation of proteins by anti-MePCE from HEK293TN cells. **e** Western blot analysis of proteins co-immunoprecipitated by anti-LARP7 from HeLa cells. **f** Co-immunoprecipitation of proteins by anti-MePCE from HeLa cells. **g** Co-immunoprecipitation of proteins by anti-Larp7 from NSC-34 cells. **h** Co-immunoprecipitation of proteins by anti-Mepce from NSC-34 cells. **i** Co-immunoprecipitation of proteins by anti-LARP7 from HeLa cells. **j** Co-immunoprecipitation of proteins by anti-MePCE from HeLa cells. **k** Co-immunoprecipitation of proteins by anti-LARP7 from HEK293TN cells. **l** Co-immunoprecipitation of proteins by anti-MePCE from HEK293TN cells. **m** qPCR analysis of 7SK co-precipitated by anti-Smn from NSC-34 cells. Data are mean ± s.d.; **$P \leq 0.01$; unpaired two-tailed t-test (n=5). **n** qPCR analysis of 7SK co-precipitated by anti-Cyclin T1 from NSC-34 cells. Data are mean ± s.d.; ****$P \leq 0.0001$; unpaired two-tailed t-test (n=3). **o** Co-immunoprecipitation of proteins by anti-Cyclin T1 from NSC-34 cells. Lack of Gapdh co-precipitation serves as specificity control. **p** Co-immunoprecipitation of proteins by anti-Smn from NSC-34 cells. **q** Co-immunoprecipitation of proteins by anti-SMN from HeLa cells. **r** Co-immunoprecipitation of proteins by anti-SMN from HEK293TN cells. Source data are provided as a Source Data file.

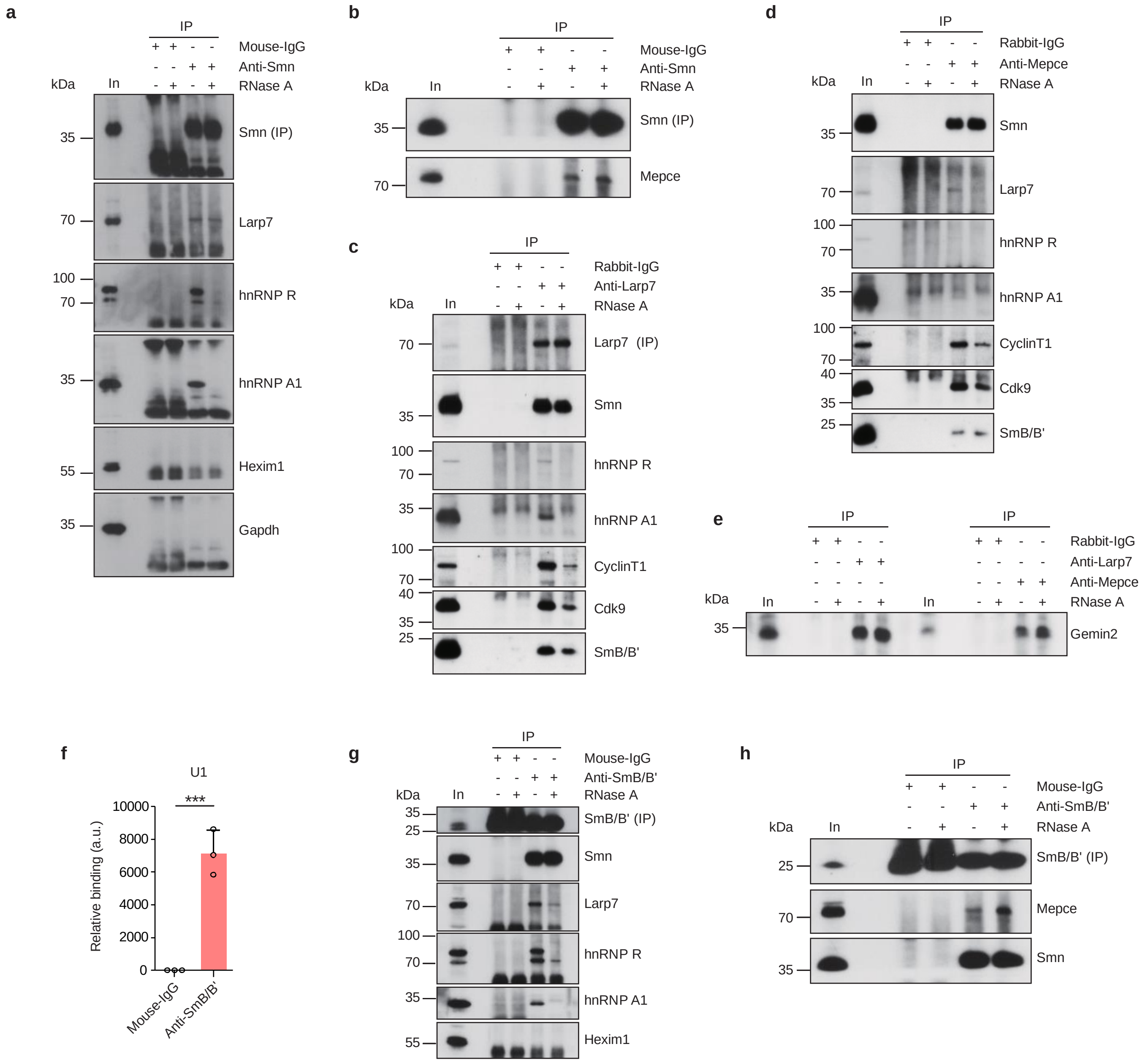

Supplementary Fig. 3 The Smn complex interacts with Larp7 and Mepce in an RNA-independent manner. a,**b** Western blot analysis of proteins co-immunoprecipitated by anti-Smn from NSC-34 cells. Lysates were pretreated with RNase as indicated. **c** Co-immunoprecipitation of proteins by anti-Larp7 from NSC-34 cells. Lysates were pretreated with RNase as indicated. **d** Co-immunoprecipitation of proteins by anti-Mepce from NSC-34 cells. Lysates were pretreated with RNase as indicated. **e** Co-immunoprecipitation of Gemin2 by anti-Mepce or anti-Larp7 from NSC-34 cells. Lysates were pretreated with RNase as indicated. **f** qPCR analysis of U1 snRNA co-precipitated by anti-SmB/B' from NSC-34 cells. Data are mean ± s.d.; ****P* ≤ 0.001; unpaired two-tailed t-test (*n*=3). **g**,**h** Co-immunoprecipitation of proteins by anti-SmB/B' from NSC-34 cells. Lysates were pretreated with RNase as indicated. Note that the immunosignal at 25 kDa for mouse-IgG immunoprecipitation is non-specific from the antibody light chain. Source data are provided as a Source Data file.

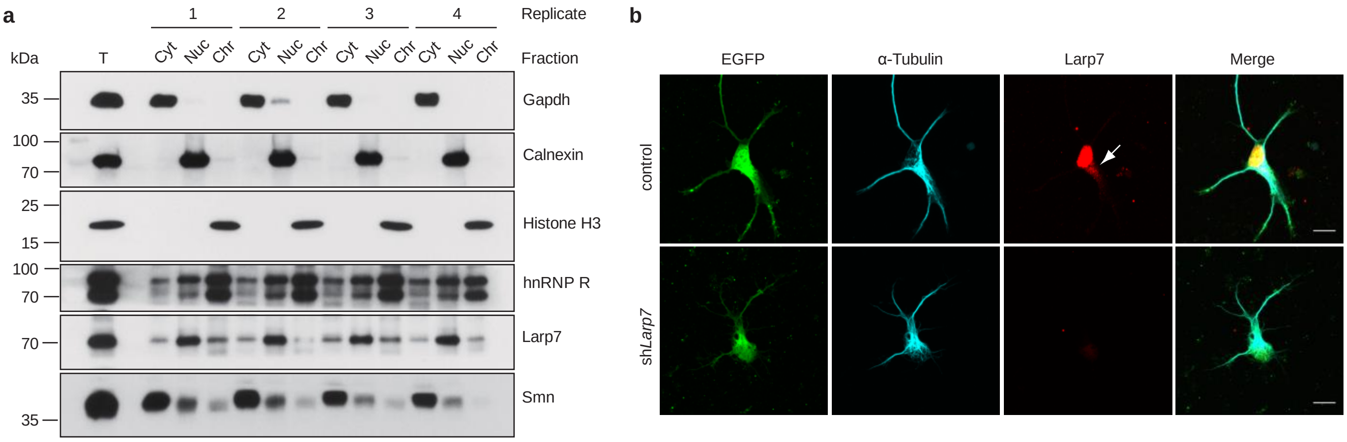

Supplementary Fig. 4 Subcellular distribution of 7SK-associated proteins in cultured primary mouse motoneurons. a Western blot analysis of motoneuron subcellular fractions. Cyt: cytosol; Nuc: nuclear soluble proteins and organelles; Chr: chromatin-associated proteins. Results of four different motoneuron cultures are shown. T, total lysate. **b** Primary mouse motoneurons transduced with Larp7 knockdown (sh*Larp7*) and control virus were cultured for 5 d and immunostained with antibodies against EGFP (to visualize virus transduction), α-Tubulin and Larp7. Cytoplasmic Larp7 immunostaining is indicated with an arrow. Scale bar: 10 µm. Source data are provided as a Source Data file.

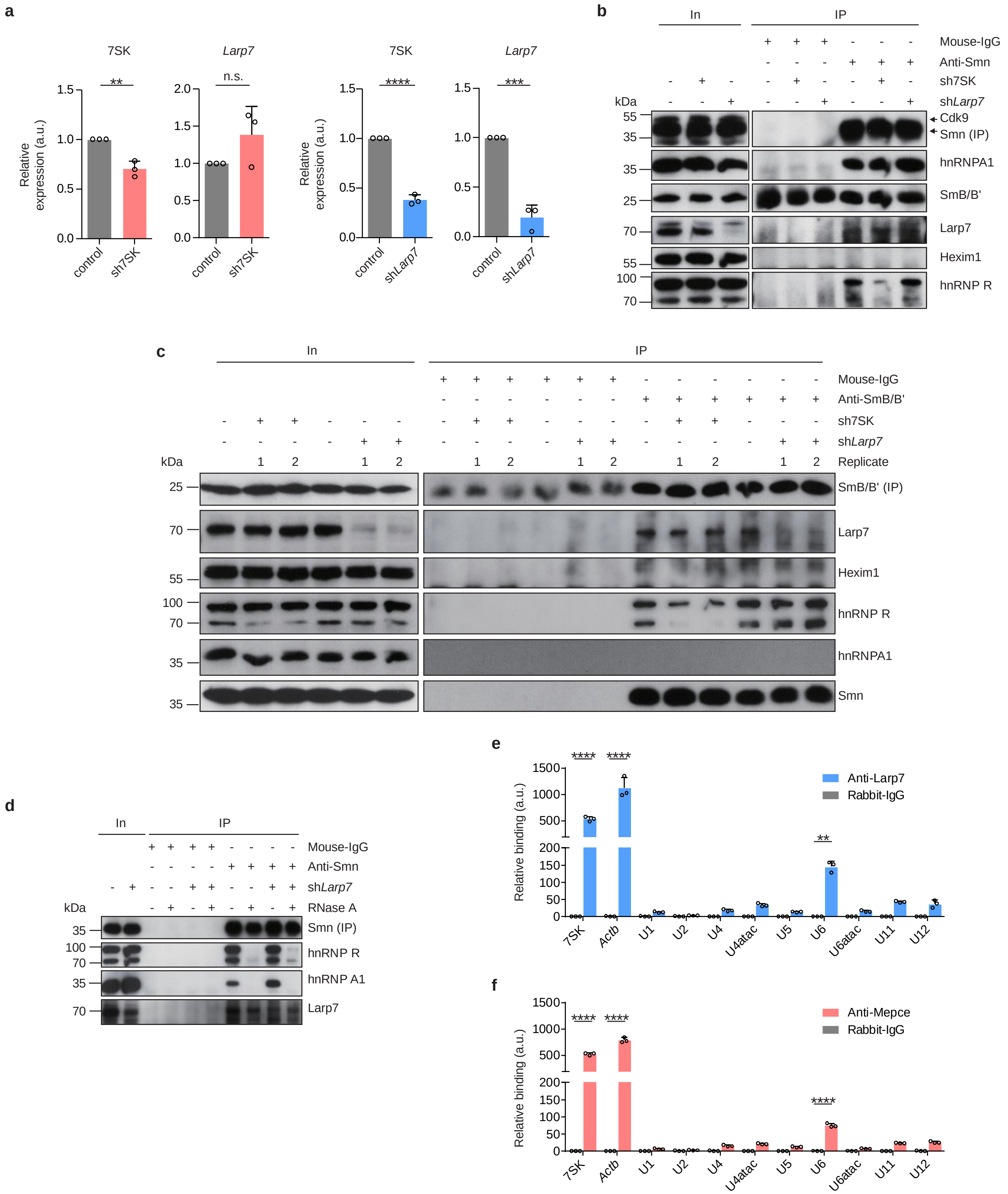

Supplementary Fig. 5 Smn binds to hnRNP R in a 7SK-dependent manner. a qPCR analysis of 7SK and *Larp7* in NSC-34 cells 96 h after transduction with lentiviral constructs expressing shRNAs (sh7SK or sh*Larp7*) or with an empty lentiviral construct as control. Data are mean ± s.d.; ***P* ≤ 0.01, ****P* ≤ 0.001, *****P* ≤ 0.0001, n.s.: not significant; unpaired two-tailed t-test (*n*=3). **b** Western blot analysis of proteins co-immunoprecipitated by anti-Smn from control, 7SK knockdown or Larp7 knockdown NSC-34 cells. Note that the immunosignal at 25 kDa for mouse-IgG immunoprecipitation is non-specific from the

antibody light chain. **c** Co-immunoprecipitation of proteins by anti-SmB/B' from control, 7SK knockdown or Larp7 knockdown NSC-34 cells. Replicate 1 and 2 represent different virus preparations. **d** Co-immunoprecipitation of proteins by anti-Smn from control and Larp7 knockdown NSC-34 cells. Lysates were pretreated with RNase as indicated. **e** qPCR analysis of 7SK, *Actb* and snRNAs co-precipitated by anti-Larp7 from NSC-34 cells. Data are mean ± s.d.; $^{**}P \leq 0.01$, $^{****}P \leq 0.0001$; two-way ANOVA with Sidak's multiple-comparisons test (n=3). **f** qPCR analysis of 7SK, *Actb* and snRNAs co-precipitated by anti-Mepce from NSC-34 cells. Data are mean ± s.d.; $^{****}P \leq 0.0001$; two-way ANOVA with Sidak's multiple-comparisons test (n=3). Source data are provided as a Source Data file.

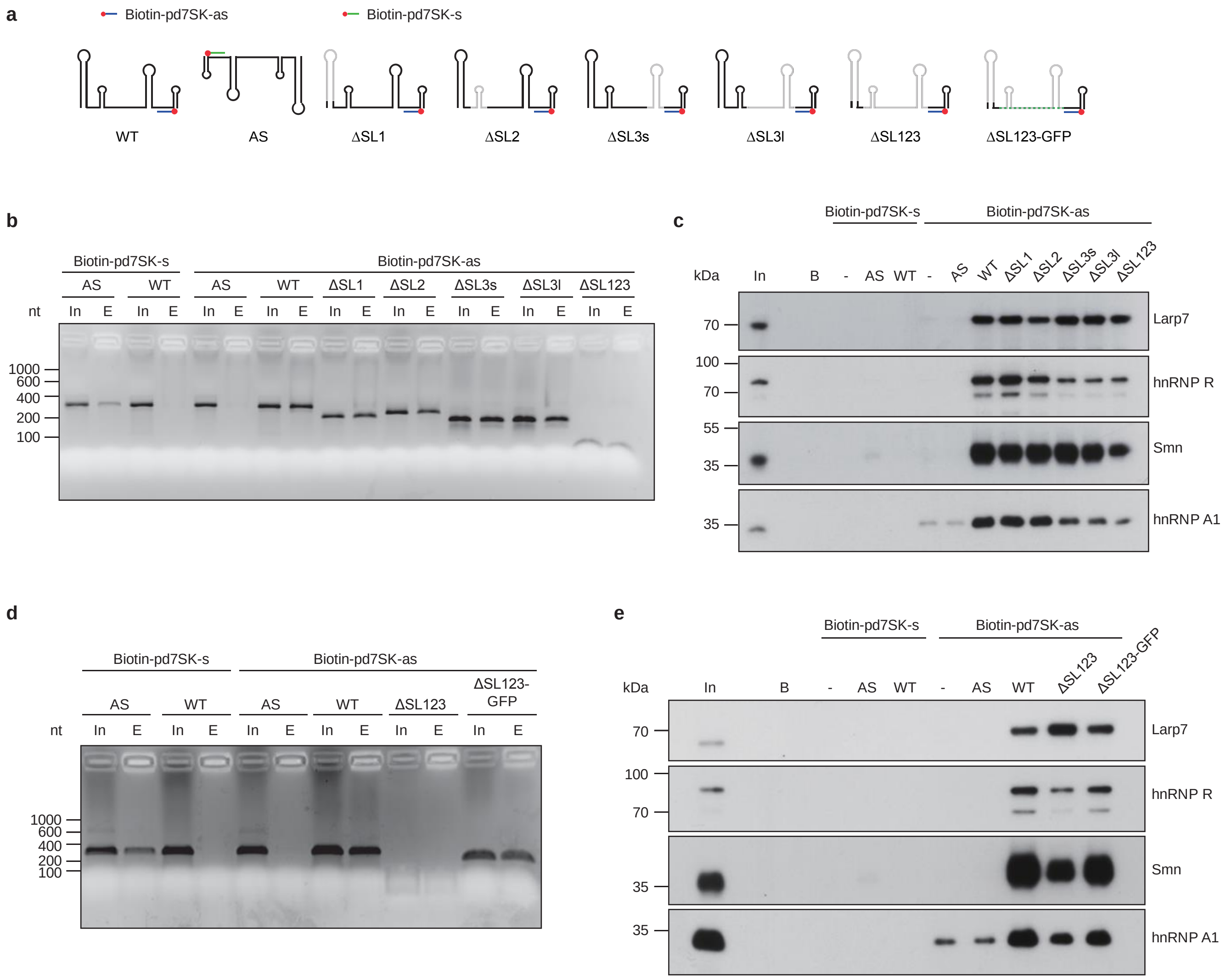

Supplementary Fig. 6 7SK RNA regions required for the assembly of 7SK/hnRNP/Smn complexes. a Schematic representation of 7SK RNAs and biotinylated oligonucleotides used for the RNA pulldown assay. WT, wildtype; AS, antisense; ΔSL, deletions in the stem-loop regions. ΔSL123-GFP is a chimeric 7SK in which SL1-3 were replaced with non-related sequence from *GFP*. Biotin-pd7SK-as oligonucleotide was used for 7SK WT and deletion mutants and Biotin-pd7SK-s oligonucleotide for 7SK AS. **b** Immobilization of *in vitro* transcribed 7SK RNAs on streptavidin-coated beads using the indicated biotinylated oligonucleotides. Agarose gel electrophoresis of the eluted RNA. E, eluate (100% loading); In, input (1 µg loading); nt, nucleotides. **c** Western blot analysis following incubation of 7SK RNAs with NSC-34 cell lysate and pulldown with streptavidin-coated beads. In, input; B, beads control; -, no RNA. **d** Same as in (**b**) but with different 7SK RNA constructs. **e** Same as in (**c**) but with different 7SK RNA constructs. Source data are provided as a Source Data file.

SUPPLEMENTARY TABLES

Supplementary Table 1: Sequences of oligonucleotides for shRNA cloning

Name	Sequence (5'-3')
shLarp7_F	GATCCGCTAATCACCAAAGCTGAGAACTTCCTGTCAGATTCTCAGCTTTGGT GATTAGCTTTTTG
shLarp7_R	AATTCAAAAAGCTAATCACCAAAGCTGAGAATCTGACAGGAAGTTCTCAGCT TTGGTGATTAGCG
shMepce_F	GATCCGAAAGTCTCTCACGGAAACAATCAAGAGTTGTTTCCGTGAGAGACT TTCTTTTTG
shMepce_R	AATTCAAAAAGAAAGTCTCTCACGGAAACAACTCTTGATTGTTTCCGTGAGA GACTTTCG
shGemin2_F	GATCCGAATCTGCTGATCTGCTTAGTTCAAGAGACTAAGCAGATCAGCAGA TTCTTTTTG
shGemin2_R	AATTCAAAAAGAATCTGCTGATCTGCTTAGTCTCTTGAACTAAGCAGATCAG CAGATTCG
shSnrpb_F	GATCCGCTTCAAAGCCTTTGACAATACCTGACCCATATTGTCAAAGGCTTTG AAGCTTTTTG
shSnrpb_R	AATTCAAAAAGCTTCAAAGCCTTTGACAATATGGGTCAGGTATTGTCAAAGG CTTTGAAGCG

Supplementary Table 2: Sequences of biotinylated oligonucleotides

Name	Sequence (5'-3')
Biotin-scr	Biotin-CAAUCAUACUGCUCACU
Biotin-pd7SK	Biotin-GACAGAUGUCGCAGCCA
Biotin-pd7SK-as	Biotin-TEG-GATGTGTCTGGAGTCTTGGA
Biotin-pd7SK-s	Biotin-TEG-TCCAAGACTCCAGACACATC

Supplementary Table 3: Sequences of oligonucleotides for PCR

Name	Sequence (5'-3')
T7-U2-F	TAATACGACTCACTATAGGGATCGCTTCTCGGCCTTTTGGCT
U2-R	GGTGCACCGTTCCTGGAGGTA

Supplementary Table 4: Sequences of oligonucleotides for qPCR

Gene	Forward primer (5'–3')	Reverse primer (5'–3')
7SK	ATTGATCGCCAGGGTTGATTCG	ATGGACCTTGAGAGCTTGTTTG
Actb	TTCTTTGCAGCTCCTTCGTTGCCG	TGGATGGCTACGTACATGGCTGGG
Gapdh	GCAAATTCAACGGCACA	CACCAGTAGACTCCACGAC
7SL	CCTGTAGTCCCAGCTACTCG	CTGCTCCGTTTCCGACCTGG
Malat1	TGCAGTGTGCCAATGTTTCG	AGTCTGCTGTTTCCTGCTCC
Larp7	CAGGCAGTAATGAATGCACAGA	GAAACGGATGTGCTGACTGG

Supplementary Table 5: Antibodies used for Western blotting (WB), immunoprecipitation (IP), immunofluorescence (IF) or motoneuron cell culture

Antibody	Application	Dilution	Source	Identifier
Goat polyclonal anti-HEXIM1	WB	1:2000	Bio-Rad	Cat#VPA00125
Rabbit polyclonal anti-HEXIM1	WB	1:1000	Bethyl	Cat#A303-113A; RRID: AB_10892626
Rabbit polyclonal anti-LARP7	IF<br>IP	1:100	Proteintech	Cat#17067-1-AP; RRID: AB_2132693
Rabbit polyclonal anti-LARP7	IP		Aviva Systems Biology	Cat# ARP40847_P050; RRID: AB_1294403
Rabbit polyclonal anti-LARP7	WB	1:1000	MyBioSource	Cat#MBS9127367
Mouse monoclonal anti-LARP7 (clone E-5)	WB	1:100	Santa Cruz	Cat#sc-515209; RRID: AB_2728652
Rabbit polyclonal anti-MEPCE	IP		Proteintech	Cat#14917-1-AP; RRID: AB_2250635
Rabbit polyclonal anti-MEPCE	WB	1:1000	Abcam	Cat#ab185991
Mouse monoclonal anti-SmB/B'/N (clone 12F5)	WB<br>IP	1:100	Santa Cruz	Cat#sc-130670; RRID: AB_2193856
Mouse monoclonal anti-SmD1 (clone A-9)	IP		Santa Cruz	Cat#sc-166650; RRID: AB_2255185
Mouse monoclonal anti-SMN (clone 8)	WB<br>IP	1:3000	BD Biosciences	Cat#610647; RRID: AB_397973
Rabbit polyclonal anti-hnRNP R	WB	1:4000	Abcam	Cat#ab30930; RRID: AB_2295532
Mouse monoclonal anti-Gemin2 (clone 3F8)	WB	1:100	Santa Cruz	Cat#sc-32806; RRID: AB_627669
Mouse monoclonal anti-Gemin2 (clone 2E17)	WB	1:1000	Millipore	Cat#05-1540; RRID: AB_10562958
Mouse monoclonal anti-Gemin3 (clone D-5)	WB	1:100	Santa Cruz	Cat#sc-271853; RRID: AB_10708130
Mouse monoclonal anti-Gemin4 (clone 17D10)	WB	1:100	Santa Cruz	Cat#sc-136199; RRID: AB_10916876
Rabbit polyclonal anti-DHX9	WB	1:1000	Proteintech	Cat#17721-1-AP; RRID: AB_2092506
Mouse monoclonal anti-hnRNP Q (clone I8E4)	WB	1:100	Santa Cruz	Cat#sc-56703; RRID: AB_2200715
Mouse monoclonal anti-Cdk9 (clone D-7)	WB	1:100	Santa Cruz	Cat#sc-13130; RRID: AB_627245
Mouse monoclonal anti-hnRNP A1 (clone 4B10)	WB	1:1000	Santa Cruz	Cat#sc-32301; RRID: AB_627729
Mouse monoclonal anti-GAPDH (clone 6C5)	WB	1:3000	Calbiochem	Cat#CB1001; RRID: AB_2107426
Mouse monoclonal anti-Cyclin T1 (clone C-6)	WB<br>IP	1:100	Santa Cruz	Cat#sc-271575; RRID: AB_10650141
Goat polyclonal anti-CALNEXIN	WB	1:2000	SICGEN	Cat#AB0041-200; RRID: AB_2333115
Rabbit polyclonal anti-Histone H3	WB	1:5000	Abcam	Cat#ab1791; RRID: AB_302613
Chicken polyclonal anti-GFP	IF	1:1000	Abcam	Cat#ab13970; RRID: AB_300798
Mouse monoclonal anti-α-Tubulin (clone B-5-1-2)	IF	1:1000	Sigma-Aldrich	Cat#T5168; RRID: AB_477579
Mouse monoclonal anti-p75NTR (clone MLR2)	Cell culture	1:10000	Biosensis	Cat#M-009-100; RRID: AB_2492396
Mouse IgG control	IP		Santa Cruz	Cat#sc-2025; RRID: AB_737182

Rabbit IgG control	IP		PeproTech	Cat#500-P00; RRID: AB_2722620
Goat polyclonal anti-Mouse, Peroxidase conjugated	WB	1:5000	Jackson ImmunoResearch	Cat#115-035-146; RRID: AB_2307392
Donkey polyclonal anti-Rabbit, Peroxidase conjugated	WB	1:5000	Jackson ImmunoResearch	Cat#711-035-152; RRID: AB_10015282
Donkey polyclonal anti-Goat, Peroxidase conjugated	WB	1:5000	Jackson ImmunoResearch	Cat#705-035-003; RRID: AB_2340390
Donkey polyclonal anti-Chicken, Alexa Fluor® 488 conjugated	IF	1:800	Jackson ImmunoResearch	Cat#703-545-155; RRID: AB_2340375
Donkey polyclonal anti-Mouse, Cy™3 conjugated	IF	1:800	Jackson ImmunoResearch	Cat#715-165-151; RRID: AB_2315777
Donkey polyclonal anti-Rabbit, Cy™5 conjugated	IF	1:800	Jackson ImmunoResearch	Cat#711-175-152; RRID: AB_2340607
