# Supplementary material for: Interaction of 7SK with the Smn complex modulates snRNP production
Source: Nat Commun. 2021 Feb 24;12:1278. doi: 10.1038/s41467-021-21529-1 (PMC7904863; doi:10.1038/s41467-021-21529-1)
Supplement: Supplementary file 6 — Description of Additional Supplementary Files [file 41467_2021_21529_MOESM6_ESM.docx]

Description of Additional Supplementary information

Title: Supplementary Data 1

Description: Unprocessed proteomics data

Title: Supplementary Data 2

Description: Processed proteomics data

Title: Supplementary Data 3

Description: Annotation enrichment analysis of proteomics data
