# Supplementary material for: Interaction of 7SK with the Smn complex modulates snRNP production
Source: Nat Commun. 2021 Feb 24;12:1278. doi: 10.1038/s41467-021-21529-1 (PMC7904863; doi:10.1038/s41467-021-21529-1)
Supplement: Supplementary file 7 — Reporting Summary [file 41467_2021_21529_MOESM7_ESM.pdf]

nature research

Corresponding author(s): Michael Sendtner, Michael Briese

Last updated by author(s): Jan 27, 2021

Reporting Summary

Nature Research wishes to improve the reproducibility of the work that we publish. This form provides structure for consistency and transparency in reporting. For further information on Nature Research policies, see our Editorial Policies and the Editorial Policy Checklist.

Statistics

For all statistical analyses, confirm that the following items are present in the figure legend, table legend, main text, or Methods section.

n/a	Confirmed	
☐	☒	The exact sample size (*n*) for each experimental group/condition, given as a discrete number and unit of measurement
☒	☐	A statement on whether measurements were taken from distinct samples or whether the same sample was measured repeatedly
☐	☒	The statistical test(s) used AND whether they are one- or two-sided<br>*Only common tests should be described solely by name; describe more complex techniques in the Methods section.*
☒	☐	A description of all covariates tested
☒	☐	A description of any assumptions or corrections, such as tests of normality and adjustment for multiple comparisons
☐	☒	A full description of the statistical parameters including central tendency (e.g. means) or other basic estimates (e.g. regression coefficient) AND variation (e.g. standard deviation) or associated estimates of uncertainty (e.g. confidence intervals)
☐	☒	For null hypothesis testing, the test statistic (e.g. F, t, r) with confidence intervals, effect sizes, degrees of freedom and P value noted<br>*Give P values as exact values whenever suitable.*
☒	☐	For Bayesian analysis, information on the choice of priors and Markov chain Monte Carlo settings
☒	☐	For hierarchical and complex designs, identification of the appropriate level for tests and full reporting of outcomes
☒	☐	Estimates of effect sizes (e.g. Cohen's *d*, Pearson's *r*), indicating how they were calculated

Our web collection on statistics for biologists contains articles on many of the points above.

Software and code

Policy information about availability of computer code

Data collection	Xcalibur (Thermo Fisher Scientific)
Data analysis	MaxQuant (version 1.6.0.15); PERSEUS (version 1.5.2.11); Fiji (2017 May 30); Prism (version 6)

For manuscripts utilizing custom algorithms or software that are central to the research but not yet described in published literature, software must be made available to editors and reviewers. We strongly encourage code deposition in a community repository (e.g. GitHub). See the Nature Research guidelines for submitting code & software for further information.

Data

Policy information about availability of data

All manuscripts must include a data availability statement. This statement should provide the following information, where applicable:

- Accession codes, unique identifiers, or web links for publicly available datasets
- A list of figures that have associated raw data
- A description of any restrictions on data availability

The mass spectrometry proteomics data have been deposited to the ProteomeXchange Consortium via the PRIDE partner repository with the dataset identifier PXD021360.

Fig. 1a and Supplementary Fig. 1d, e have associated raw data in Supplementary Data 1-3.

Field-specific reporting

Please select the one below that is the best fit for your research. If you are not sure, read the appropriate sections before making your selection.

☒ Life sciences ☐ Behavioural & social sciences ☐ Ecological, evolutionary & environmental sciences

For a reference copy of the document with all sections, see nature.com/documents/nr-reporting-summary-flat.pdf

Life sciences study design

All studies must disclose on these points even when the disclosure is negative.

Sample size	All experiments were performed at least in triplicates. No calculations were done to predetermine sample size. Sample size was chosen based on established standards for tissues and primary neurons from animal models and cell lines.
Data exclusions	Based on principal component analysis, the fourth of four replicates of both the Biotin-pd7SK and Biotin-scr interaction proteomes were outliers and thus removed from further analysis. Otherwise no data were excluded from the analysis.
Replication	All experiments were performed at least in triplicates. Replication was successful, no replicates were excluded except as indicated in the Data exclusions section above.
Randomization	All experiments were conducted with defined cell lines or, in case of primary neurons and tissues, with defined genetic backgrounds. For all experiments, samples were allocated randomly, and control and treatment conditions were derived from the same batch of cells or tissue.
Blinding	Samples for different experimental conditions were processed simultaneously such that blinding was not necessary.

Reporting for specific materials, systems and methods

We require information from authors about some types of materials, experimental systems and methods used in many studies. Here, indicate whether each material, system or method listed is relevant to your study. If you are not sure if a list item applies to your research, read the appropriate section before selecting a response.

Materials & experimental systems

n/a	Involved in the study	
☐	☒	Antibodies
☐	☒	Eukaryotic cell lines
☒	☐	Palaeontology and archaeology
☐	☒	Animals and other organisms
☒	☐	Human research participants
☒	☐	Clinical data
☒	☐	Dual use research of concern

Methods

n/a	Involved in the study	
☒	☐	ChIP-seq
☒	☐	Flow cytometry
☒	☐	MRI-based neuroimaging

Antibodies

Antibodies used

Goat polyclonal anti-HEXIM1, Bio-Rad, Cat#VPA00125;
Rabbit polyclonal anti-HEXIM1, Bethyl, Cat#A303-113A, RRID: AB_10892626;
Rabbit polyclonal anti-LARP7, Proteintech, Cat#17067-1-AP, RRID: AB_2132693;
Rabbit polyclonal anti-LARP7, Aviva Systems Biology, Cat#ARP40847_P050, RRID: AB_1294403;
Rabbit polyclonal anti-LARP7, MyBioSource, Cat#MBS9127367;
Mouse monoclonal anti-LARP7 (clone E-5), Santa Cruz, Cat#sc-515209, RRID: AB_2728652;
Rabbit polyclonal anti-MEPCE, Proteintech, Cat#14917-1-AP, RRID: AB_2250635;
Rabbit polyclonal anti-MEPCE, Abcam, Cat#ab185991;
Mouse monoclonal anti-SmB/B'/N (clone 12F5), Santa Cruz, Cat#sc-130670, RRID: AB_2193856;
Mouse monoclonal anti-SmD1 (clone A-9), Santa Cruz, Cat#sc-166650, RRID: AB_2255185;
Mouse monoclonal anti-SMN (clone 8), BD Biosciences, Cat#610647, RRID: AB_397973;
Rabbit polyclonal anti-hnRNP R, Abcam, Cat#ab30930, RRID: AB_2295532;
Mouse monoclonal anti-Gemin2 (clone 3F8), Santa Cruz, Cat#sc-32806, RRID: AB_627669;
Mouse monoclonal anti-Gemin2 (clone 2E17), Millipore, Cat#05-1540, RRID: AB_10562958;
Mouse monoclonal anti-Gemin3 (clone D-5), Santa Cruz, Cat#sc-271853, RRID: AB_10708130;
Mouse monoclonal anti-Gemin4 (clone 17D10), Santa Cruz, Cat# sc-136199, RRID: AB_10916876;
Rabbit polyclonal anti-DHX9, Proteintech, Cat#17721-1-AP, RRID: AB_2092506;
Mouse monoclonal anti-hnRNP Q (clone I8E4), Santa Cruz, Cat#sc-56703, RRID: AB_2200715;
Mouse monoclonal anti-Cdk9 (clone D-7), Santa Cruz, Cat#sc-13130, RRID: AB_627245;
Mouse monoclonal anti-hnRNP A1 (clone 4B10), Santa Cruz, Cat#sc-32301, RRID: AB_627729;
Mouse monoclonal anti-GAPDH (clone 6C5), Calbiochem, Cat#CB1001, RRID: AB_2107426;

Mouse monoclonal anti-Cyclin T1 (clone C-6), Santa Cruz, Cat#sc-271575, RRID: AB_10650141;
Goat polyclonal anti-CALNEXIN, SICGEN, Cat#AB0041-200, RRID: AB_2333115;
Rabbit polyclonal anti-Histone H3, Abcam, Cat#ab1791, RRID: AB_302613;
Chicken polyclonal anti-GFP, Abcam, Cat#ab13970, RRID: AB_300798;
Mouse monoclonal anti-α-Tubulin (clone B-5-1-2), Sigma-Aldrich, Cat#T5168, RRID: AB_477579;
Mouse monoclonal anti-p75NTR (clone MLR2), Biosensis, Cat#M-009-100, RRID: AB_2492396;
Goat polyclonal anti-Mouse (Peroxidase conjugated), Jackson ImmunoResearch, Cat#115-035-146, RRID: AB_2307392;
Donkey polyclonal anti-Rabbit (Peroxidase conjugated), Jackson ImmunoResearch, Cat#711-035-152, RRID: AB_10015282;
Donkey polyclonal anti-Goat (Peroxidase conjugated), Jackson ImmunoResearch, Cat#705-035-003, RRID: AB_2340390;
Donkey polyclonal anti-Chicken (Alexa Fluor® 488 conjugated), Jackson ImmunoResearch, Cat#703-545-155, RRID: AB_2340375;
Donkey polyclonal anti-Mouse (Cy™3 conjugated), Jackson ImmunoResearch, Cat#715-165-151, RRID: AB_2315777;
Donkey polyclonal anti-Rabbit (Cy™5 conjugated), Jackson ImmunoResearch, Cat#711-175-152, RRID: AB_2340607

Validation

Goat polyclonal anti-HEXIM1, Bio-Rad, Cat#VPA00125: validation data provided by the supplier (https://www.bio-rad-antibodies.com/polyclonal/human-hexim1-antibody-vpa00125.html);
Rabbit polyclonal anti-HEXIM1, Bethyl, Cat#A303-113A, RRID: AB_10892626: validation data provided by the supplier (https://www.bethyl.com/product/A303-113A/HEXIM1+Antibody);
Rabbit polyclonal anti-LARP7, Proteintech, Cat#17067-1-AP, RRID: AB_2132693: validation data provided by the supplier (https://www.ptglab.com/products/LARP7-Antibody-17067-1-AP.htm) and validated in our lab by shRNA knockdown;
Rabbit polyclonal anti-LARP7, Aviva Systems Biology, Cat#ARP40847_P050, RRID: AB_1294403: validation data provided by the supplier (https://www.avivasysbio.com/larp7-antibody-c-terminal-region-biotin-arp40847-p050-biotin.html);
Rabbit polyclonal anti-LARP7, MyBioSource, Cat#MBS9127367: validation data provided by the supplier (https://www.mybiosource.com/polyclonal-human-mouse-rat-antibody/larp7/2530149) and validated in our lab by shRNA knockdown;
Mouse monoclonal anti-LARP7 (clone E-5), Santa Cruz, Cat#sc-515209, RRID: AB_2728652: validation data provided by the supplier (https://www.scbt.com/p/larp7-antibody-e-5);
Rabbit polyclonal anti-MEPCE, Proteintech, Cat#14917-1-AP, RRID: AB_2250635: validation data provided by the supplier (https://www.ptglab.com/products/MEPCE-Antibody-14917-1-AP.htm);
Rabbit polyclonal anti-MEPCE, Abcam, Cat#ab185991: validation data provided by the supplier (https://www.abcam.com/mepce-antibody-c-terminal-ab185991.html), validated in our lab by shRNA knockdown and also characterized in detail in Egloff S et. al., EMBO J, 2017;
Mouse monoclonal anti-SmB/B'/N (clone 12F5), Santa Cruz, Cat#sc-130670, RRID: AB_2193856: validation data provided by the supplier (https://www.scbt.com/p/sm-b-b-n-antibody-12f5) and validated in our lab by shRNA knockdown;
Mouse monoclonal anti-SmD1 (clone A-9), Santa Cruz, Cat#sc-166650, RRID: AB_2255185: validation data provided by the supplier (https://www.scbt.com/p/sm-d1-antibody-a-9);
Mouse monoclonal anti-SMN (clone 8), BD Biosciences, Cat#610647, RRID: AB_397973: validation data provided by the supplier (https://www.bdbiosciences.com/us/applications/research/stem-cell-research/ectoderm-markers/human/purified-mouse-anti-smn-8smn/p/610647) and validated in our lab by shRNA knockdown and by knockout;
Rabbit polyclonal anti-hnRNP R, Abcam, Cat#ab30930, RRID: AB_2295532: validation data provided by the supplier (https://www.abcam.com/hnrnp-r-antibody-ab30930.html) and validated by shRNA knockdown in Briese M et al., PNAS, 2018;
Mouse monoclonal anti-Gemin2 (clone 3F8), Santa Cruz, Cat#sc-32806, RRID: AB_627669: validation data provided by the supplier (https://www.scbt.com/p/gemin2-antibody-3f8) and validated in our lab by shRNA knockdown;
Mouse monoclonal anti-Gemin2 (clone 2E17), Millipore, Cat#05-1540, RRID: AB_10562958: validation data provided by the supplier (https://www.merckmillipore.com/DE/de/product/Anti-Gemin2-Antibody-clone-2E17,MM_NF-05-1540);
Mouse monoclonal anti-Gemin3 (clone D-5), Santa Cruz, Cat#sc-271853, RRID: AB_10708130: validation data provided by the supplier (https://www.scbt.com/p/gemin3-antibody-d-5);
Mouse monoclonal anti-Gemin4 (clone 17D10), Santa Cruz, Cat# sc-136199, RRID: AB_10916876: validation data provided by the supplier (https://www.scbt.com/p/gemin4-antibody-17d10);
Rabbit polyclonal anti-DHX9, Proteintech, Cat#17721-1-AP, RRID: AB_2092506: validation data provided by the supplier (https://www.ptglab.com/products/DHX9-Antibody-17721-1-AP.htm);
Mouse monoclonal anti-hnRNP Q (clone I8E4), Santa Cruz, Cat#sc-56703, RRID: AB_2200715: validation data provided by the supplier (https://www.scbt.com/p/hnrnp-q-antibody-i8e4);
Mouse monoclonal anti-Cdk9 (clone D-7), Santa Cruz, Cat#sc-13130, RRID: AB_627245: validation data provided by the supplier (https://www.scbt.com/p/cdk9-antibody-d-7);
Mouse monoclonal anti-hnRNP A1 (clone 4B10), Santa Cruz, Cat#sc-32301, RRID: AB_627729: validation data provided by the supplier (https://www.scbt.com/p/hnrnp-a1-antibody-4b10);
Mouse monoclonal anti-GAPDH (clone 6C5), Calbiochem, Cat#CB1001, RRID: AB_2107426: validation data provided by the supplier (https://www.merckmillipore.com/DE/de/product/Anti-GAPDH-Mouse-mAb-6C5,EMD_BIO-CB1001);
Mouse monoclonal anti-Cyclin T1 (clone C-6), Santa Cruz, Cat#sc-271575, RRID: AB_10650141: validation data provided by the supplier (https://www.scbt.com/p/cyclin-t1-antibody-c-6);
Goat polyclonal anti-CALNEXIN, SICGEN, Cat#AB0041-200, RRID: AB_2333115: validation data provided by the supplier (http://www.sicgen.pt/product/calnexin-polyclonal-antibody_1_3);
Rabbit polyclonal anti-Histone H3, Abcam, Cat#ab1791, RRID: AB_302613: validation data provided by the supplier (https://www.abcam.com/histone-h3-antibody-nuclear-marker-and-chip-grade-ab1791.html);
Chicken polyclonal anti-GFP, Abcam, Cat#ab13970, RRID: AB_300798: validation data provided by the supplier (https://www.abcam.com/gfp-antibody-ab13970.html);
Mouse monoclonal anti-α-Tubulin (clone B-5-1-2), Sigma-Aldrich, Cat#T5168, RRID: AB_477579: validation data provided by the supplier (https://www.sigmaaldrich.com/catalog/product/sigma/t5168?lang=de&region=DE);
Mouse monoclonal anti-p75NTR (clone MLR2), Biosensis, Cat#M-009-100, RRID: AB_2492396: validation data provided by the supplier (http://www.biosensis.com/product_info.php/mouse-monoclonal-antibody-human-ngfrp75ntr-p-1642) and used in Wiese S et al., Nat Protoc, 2010;
Goat polyclonal anti-Mouse (Peroxidase conjugated), Jackson ImmunoResearch, Cat#115-035-146, RRID: AB_2307392: validated in our lab by omission of primary antibody, absence of target antigen and used under similar conditions also for other projects;
Donkey polyclonal anti-Rabbit (Peroxidase conjugated), Jackson ImmunoResearch, Cat#711-035-152, RRID: AB_10015282: validated in our lab by omission of primary antibody, absence of target antigen and used under similar conditions also for other projects;
Donkey polyclonal anti-Goat (Peroxidase conjugated), Jackson ImmunoResearch, Cat#705-035-003, RRID: AB_2340390: validated in our lab by omission of primary antibody, absence of target antigen and used under similar conditions also for other projects;

Donkey polyclonal anti-Chicken (Alexa Fluor® 488 conjugated), Jackson ImmunoResearch, Cat#703-545-155, RRID: AB_2340375: validated in our lab by omission of primary antibody, absence of target antigen and used under similar conditions also for other projects;
Donkey polyclonal anti-Mouse (Cy™3 conjugated), Jackson ImmunoResearch, Cat#715-165-151, RRID: AB_2315777: validated in our lab by omission of primary antibody, absence of target antigen and used under similar conditions also for other projects;
Donkey polyclonal anti-Rabbit (Cy™5 conjugated), Jackson ImmunoResearch, Cat#711-175-152, RRID: AB_2340607: validated in our lab by omission of primary antibody, absence of target antigen and used under similar conditions also for other projects

Eukaryotic cell lines

Policy information about cell lines

Cell line source(s)	NSC-34 cells (Cedarlane, cat. no. CLU140); HEK293TN cells (System Biosciences, cat. no. LV900A-1); HeLa cells (Leibniz Institute DSMZ-German Collection of Microorganisms and Cell Cultures GmbH, DSMZ no. ACC 57)
Authentication	Cell lines were obtained commercially and were not authenticated.
Mycoplasma contamination	All cell lines tested negative for mycoplasma contamination.
Commonly misidentified lines (See ICLAC register)	No commonly misidentified cell lines were used in the study.

Animals and other organisms

Policy information about studies involving animals; ARRIVE guidelines recommended for reporting animal research

Laboratory animals	CD-1 mice and Smn+/-;SMN2 mice (Monani U et al., Hum Mol Genet, 2000) of both genders were housed in the animal facilities of the Institute of Clinical Neurobiology at the University Hospital Wuerzburg. Breeding animals were between 6 and 20 weeks of age. Pregnancy in female mice was detected by daily plug control, and mouse embryos were isolated at E12.5 of gestation. Tissues were obtained from embryonic and postnatal mice at defined ages as indicated in the manuscript. Mice were kept under controlled conditions in a 12 h/12 h day/night cycle at 20–22°C and 55–65% humidity with abundant supply of food and water.
Wild animals	The study did not involve wild animals.
Field-collected samples	The study did not involve samples collected from the field.
Ethics oversight	Experiments were performed strictly following the regulations on animal protection of the German federal law and of the Association for Assessment and Accreditation of Laboratory Animal Care, in agreement with and under control of the local veterinary authority.

Note that full information on the approval of the study protocol must also be provided in the manuscript.
