# Supplementary material for: Interaction of 7SK with the Smn complex modulates snRNP production
Source: Nat Commun. 2021 Feb 24;12:1278. doi: 10.1038/s41467-021-21529-1 (PMC7904863; doi:10.1038/s41467-021-21529-1)

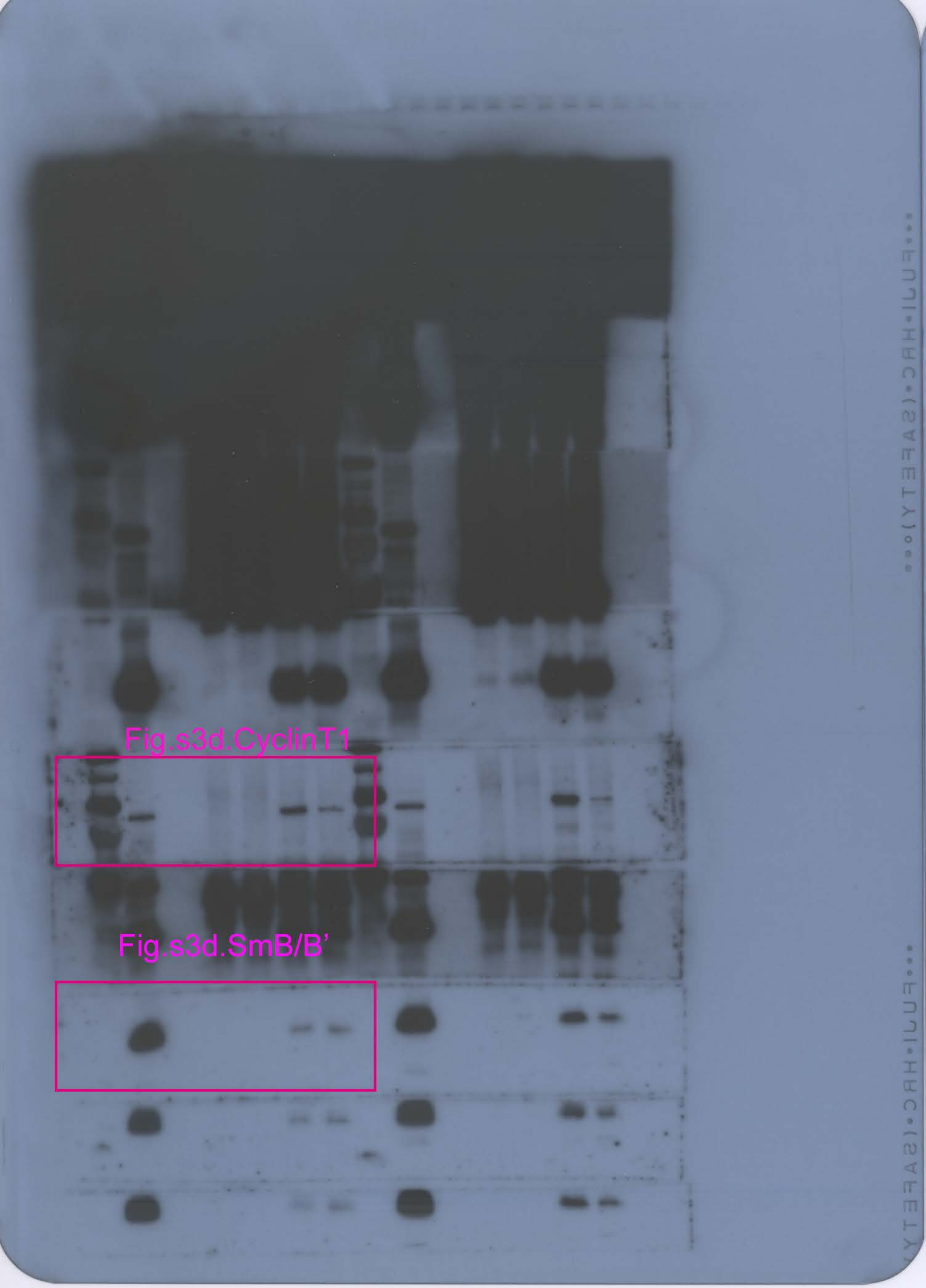
Fig.s3d.CyclinT1
Fig.s3d.SmB/B'

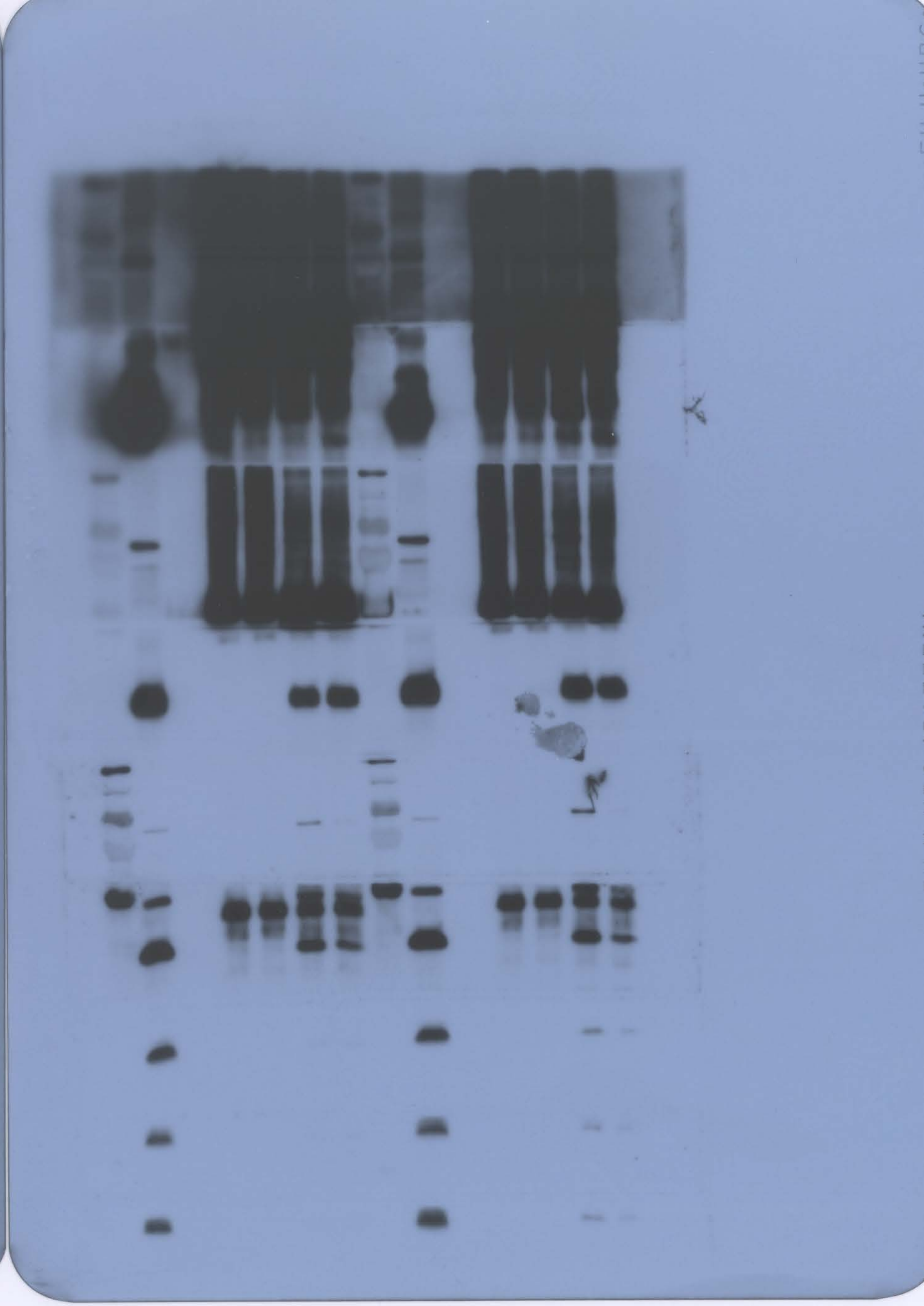

Supplement: Supplementary file 8 — Source Data [file 41467_2021_21529_MOESM8_ESM.zip › Uncropped blot and gel images/FigureS3/FigureS3d/CyclinT1_SmB.pdf]
