# Supplementary material for: Interaction of 7SK with the Smn complex modulates snRNP production
Source: Nat Commun. 2021 Feb 24;12:1278. doi: 10.1038/s41467-021-21529-1 (PMC7904863; doi:10.1038/s41467-021-21529-1)
Supplement: Supplementary file 8 — Source Data [file 41467_2021_21529_MOESM8_ESM.zip › Uncropped blot and gel images/FigureS5/FigureS5c/Larp7_Hexim1_hnRNP R.pdf]

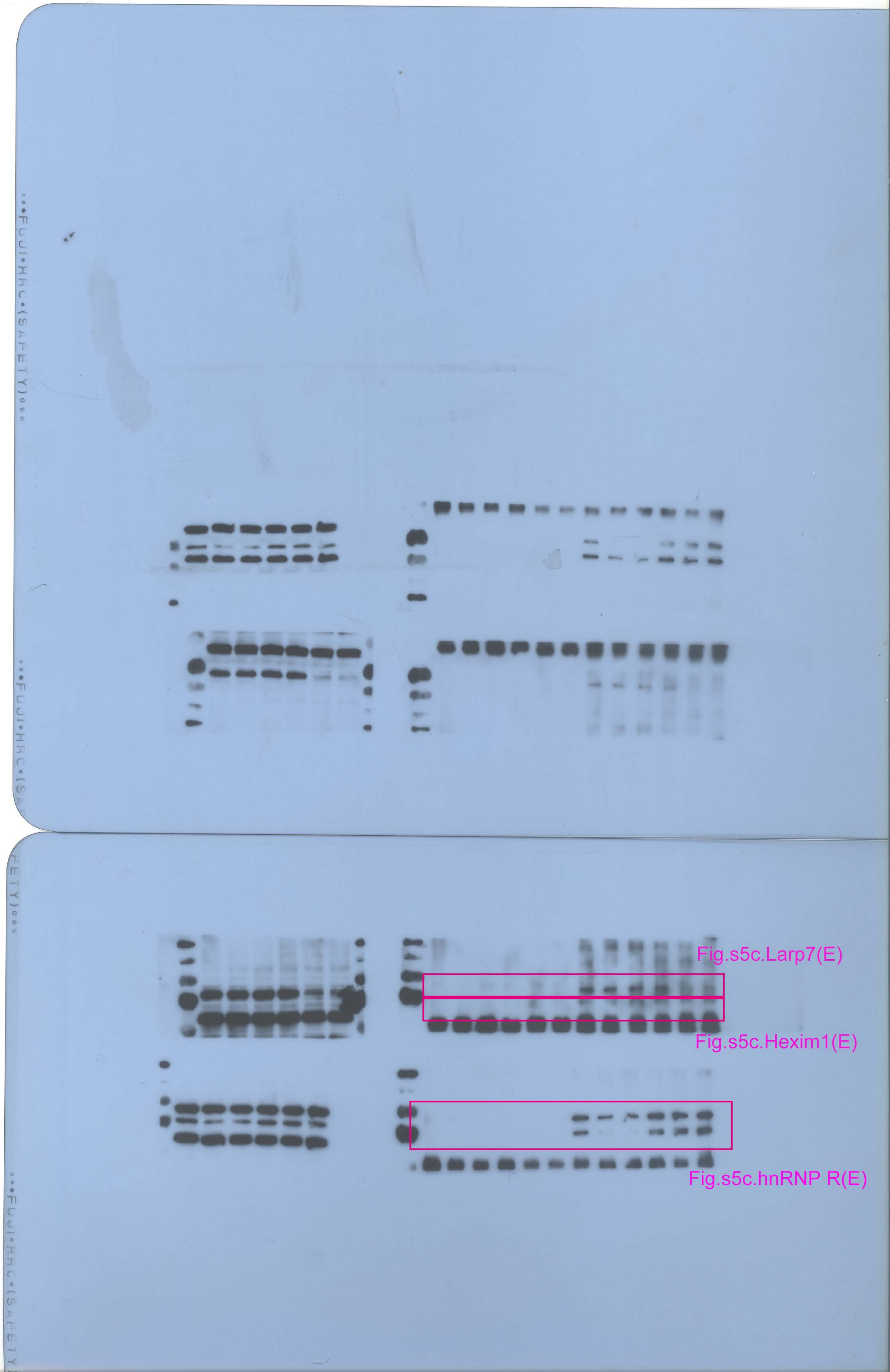

FUJI•HRC•(SAFETY)
Fig.s5c.Larp7(E)
Fig.s5c.Hexim1(E)
Fig.s5c.hnRNP R(E)
