# Supplementary figures and images for: Interaction of 7SK with the Smn complex modulates snRNP production (part 2 of 2)
Source: Nat Commun. 2021 Feb 24;12:1278. doi: 10.1038/s41467-021-21529-1 (PMC7904863; doi:10.1038/s41467-021-21529-1)

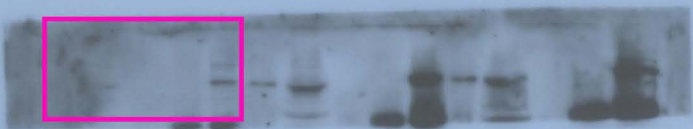

Fig.s2h.

Larp7

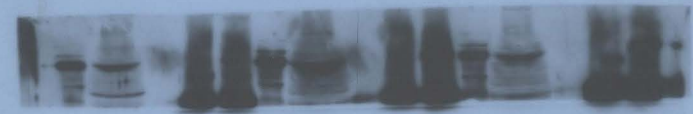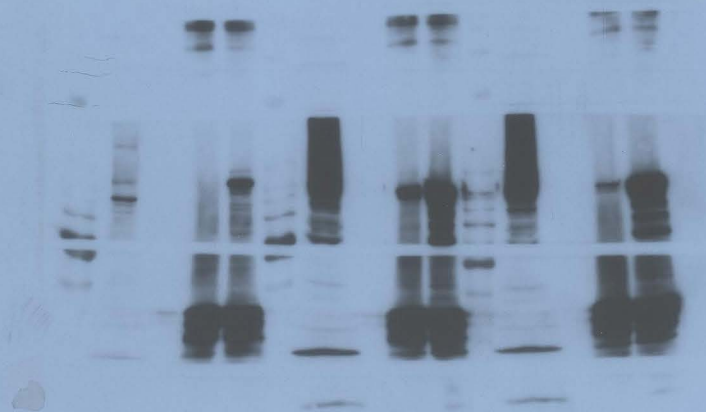

Supplement: Supplementary file 8 — Source Data [file 41467_2021_21529_MOESM8_ESM.zip › Uncropped blot and gel images/FigureS2/FigureS2h/Larp7.pdf]

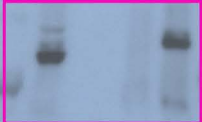

Fig.s2h.  
Mepce

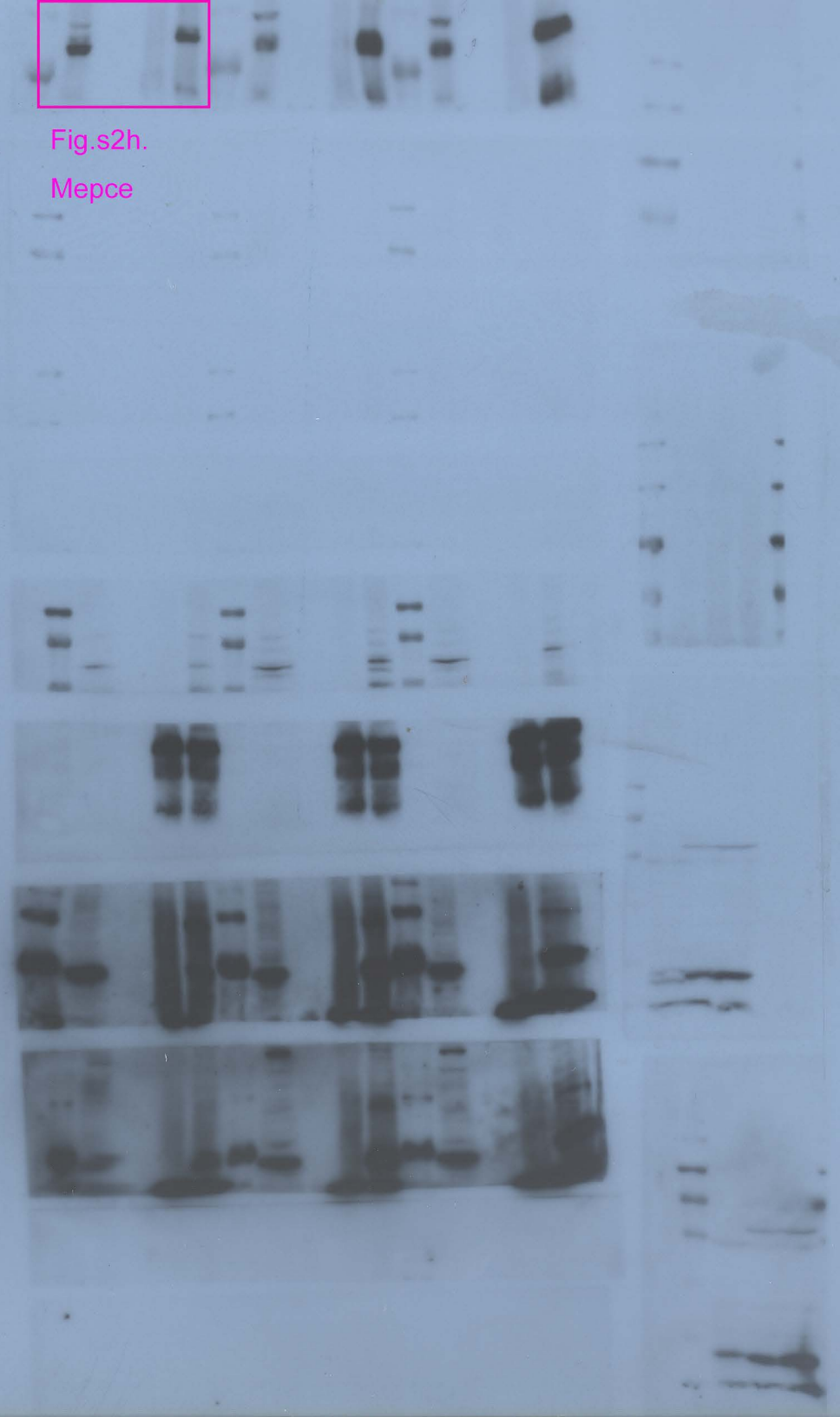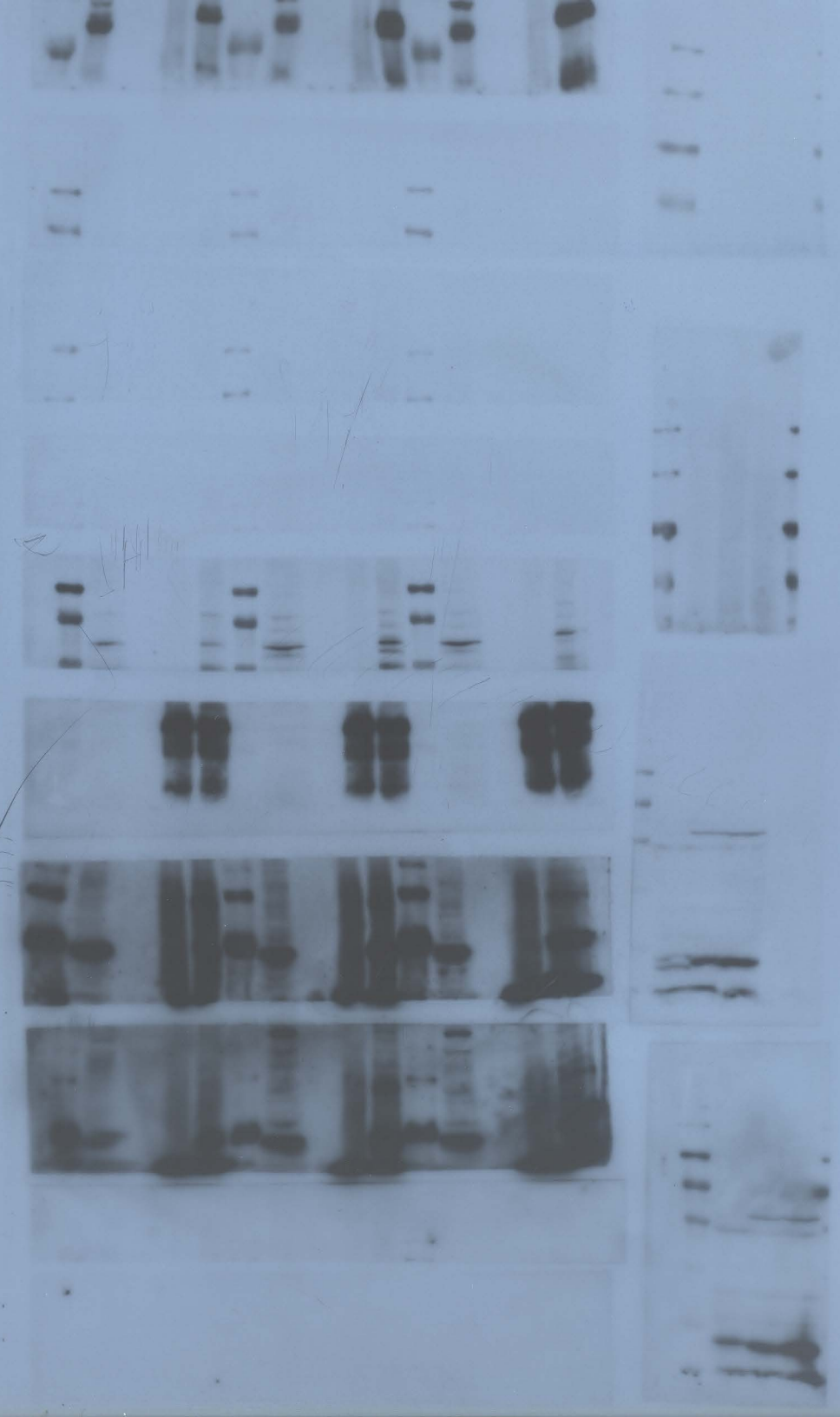

Supplement: Supplementary file 8 — Source Data [file 41467_2021_21529_MOESM8_ESM.zip › Uncropped blot and gel images/FigureS2/FigureS2h/Mepce.pdf]

Fig.s2h.RHA

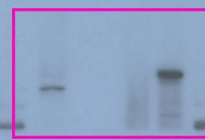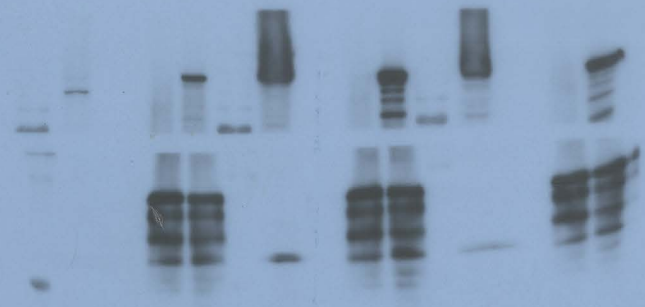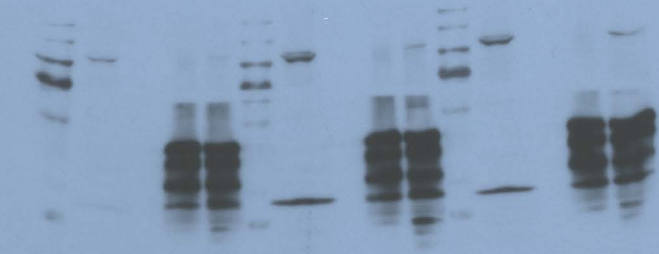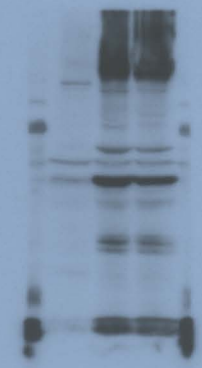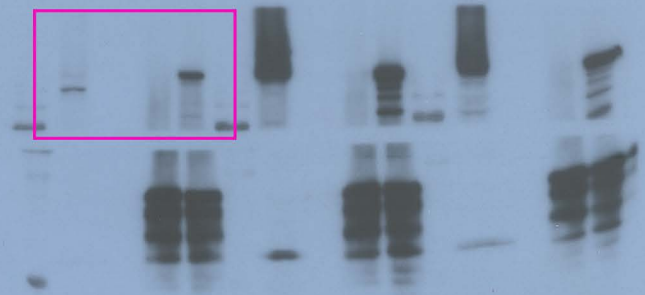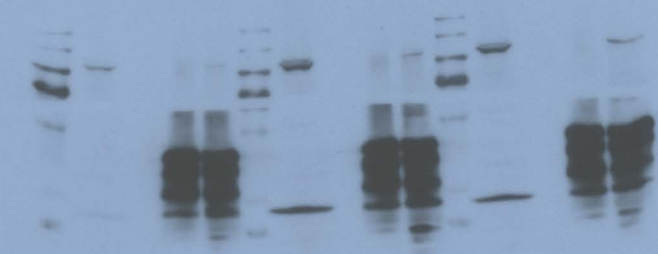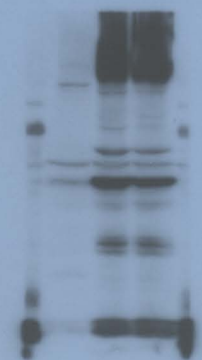

Supplement: Supplementary file 8 — Source Data [file 41467_2021_21529_MOESM8_ESM.zip › Uncropped blot and gel images/FigureS2/FigureS2h/RHA.pdf]

Fig.s2i.GEMIN4

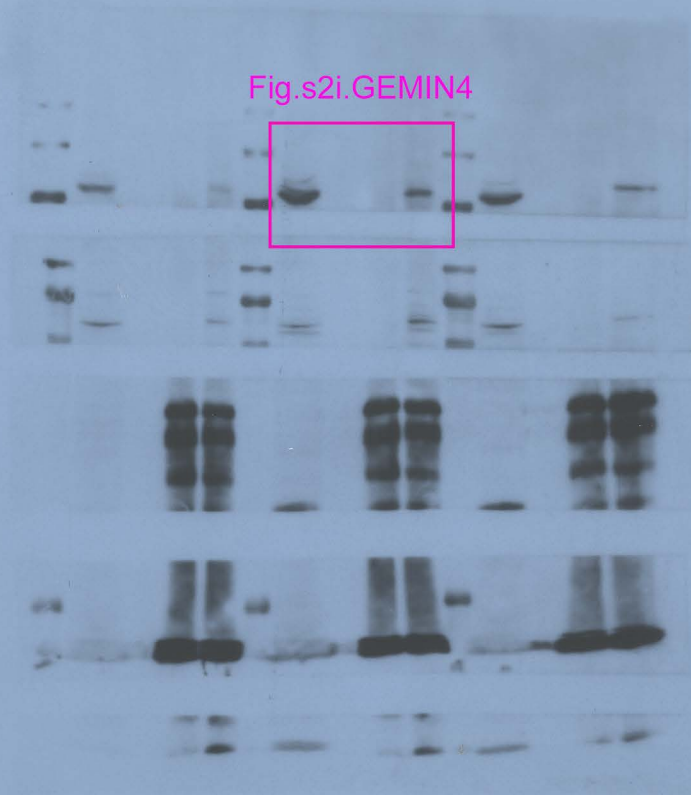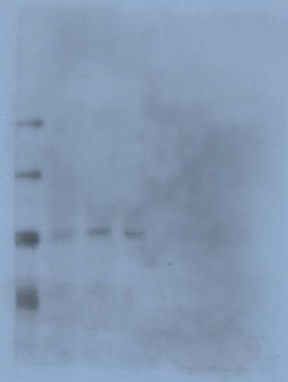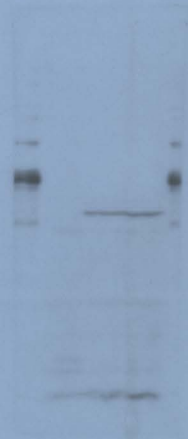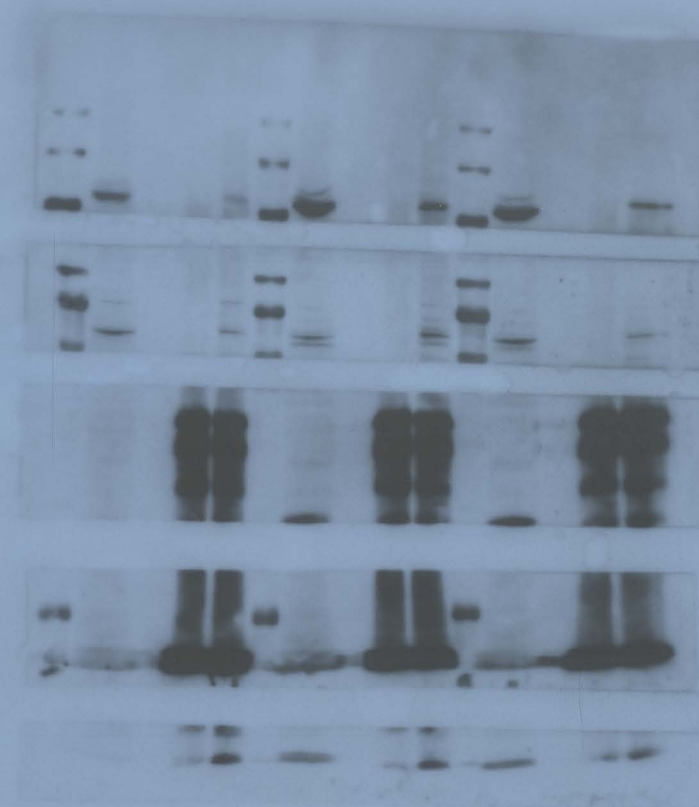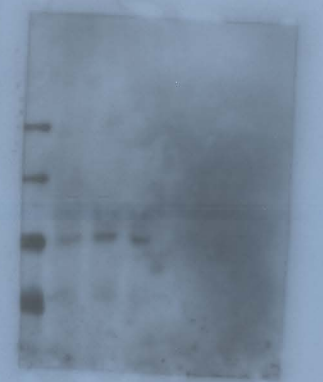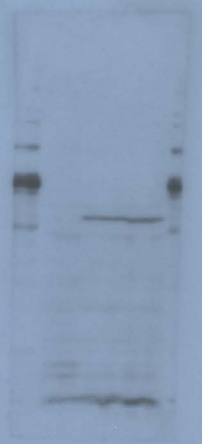

Supplement: Supplementary file 8 — Source Data [file 41467_2021_21529_MOESM8_ESM.zip › Uncropped blot and gel images/FigureS2/FigureS2i/GEMIN4.pdf]

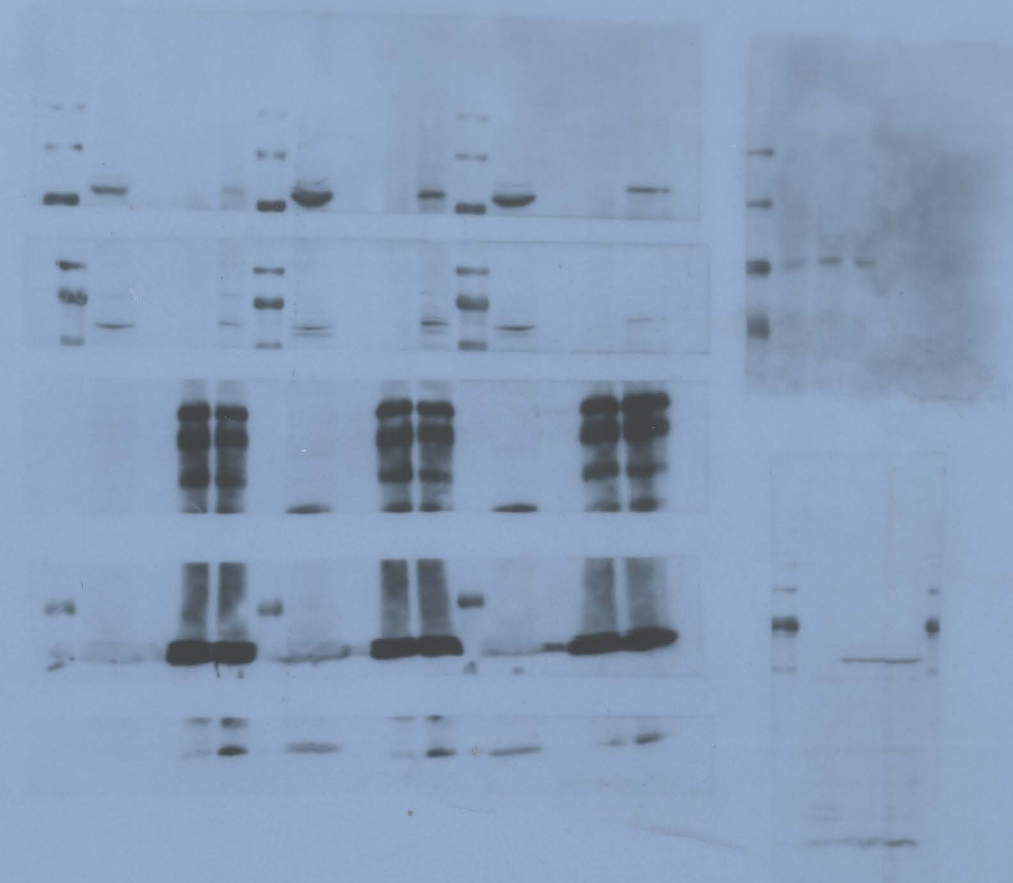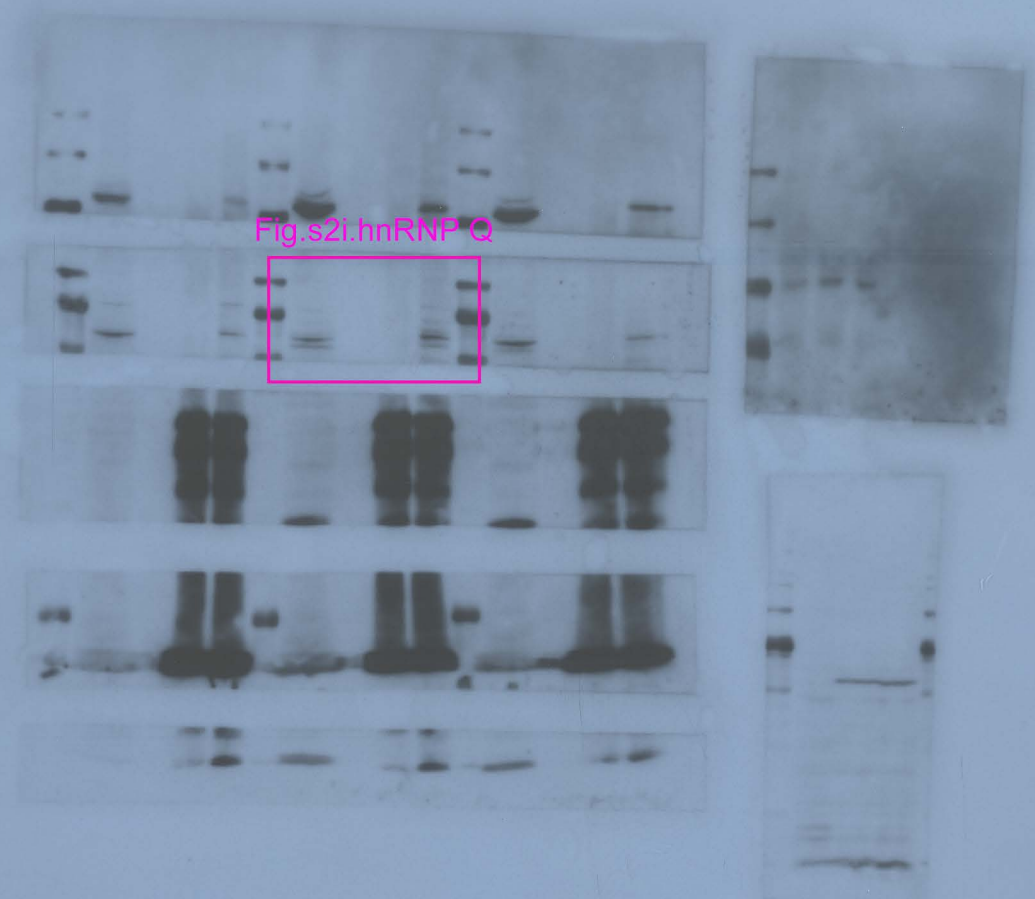

Fig.s21.hnRNP Q

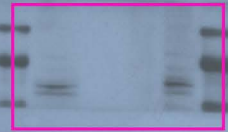

Supplement: Supplementary file 8 — Source Data [file 41467_2021_21529_MOESM8_ESM.zip › Uncropped blot and gel images/FigureS2/FigureS2i/hnRNP Q.pdf]

Fig.s2i.LARP7

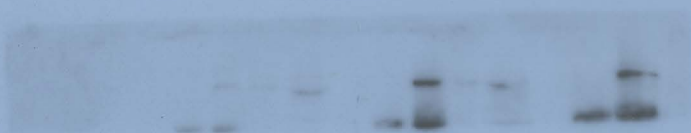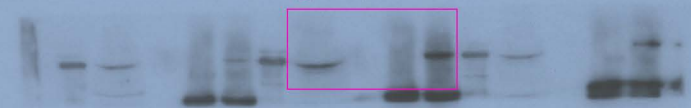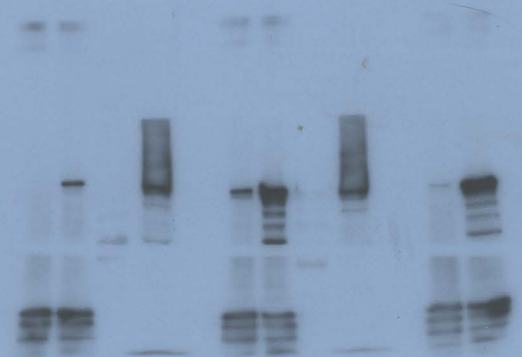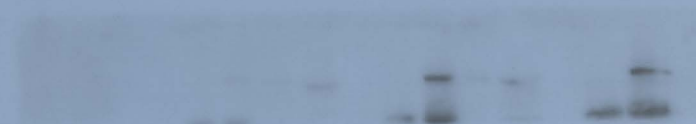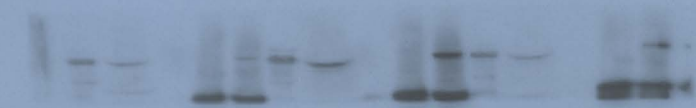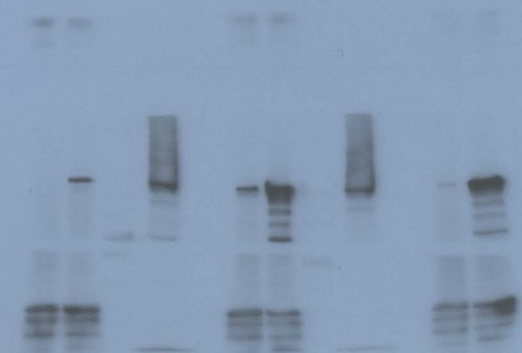

Supplement: Supplementary file 8 — Source Data [file 41467_2021_21529_MOESM8_ESM.zip › Uncropped blot and gel images/FigureS2/FigureS2i/LARP7.pdf]

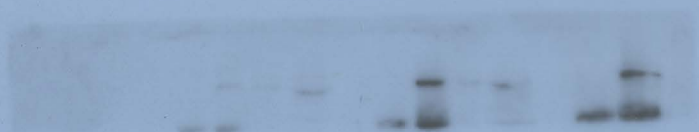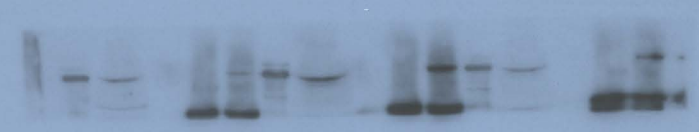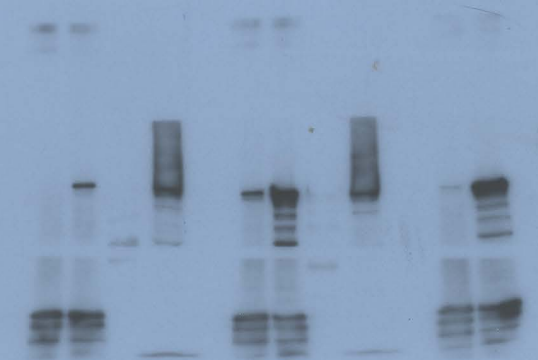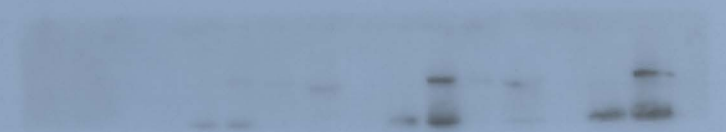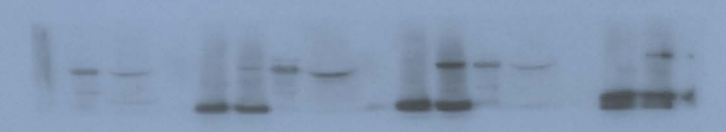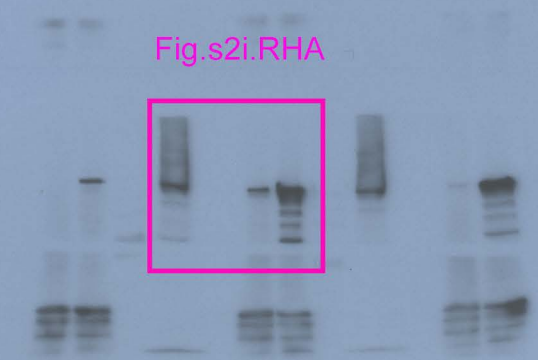

Fig.s21.RHA

Supplement: Supplementary file 8 — Source Data [file 41467_2021_21529_MOESM8_ESM.zip › Uncropped blot and gel images/FigureS2/FigureS2i/RHA.pdf]

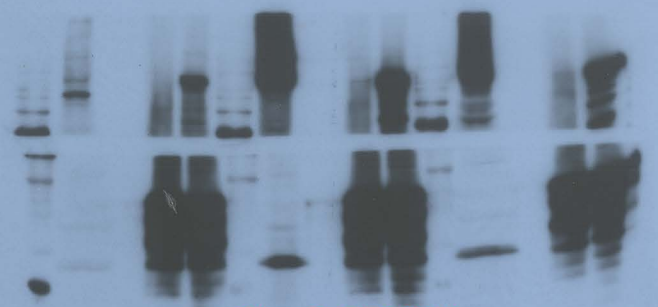

Fig.s2j.GEMIN4

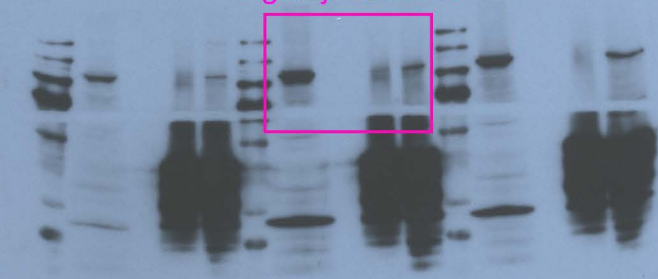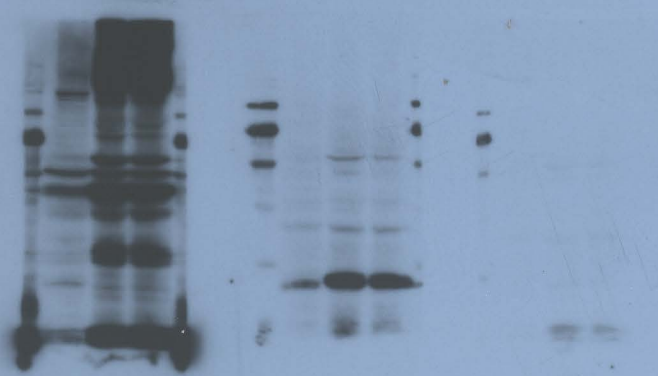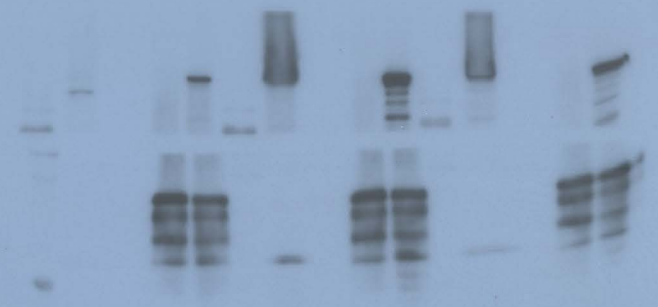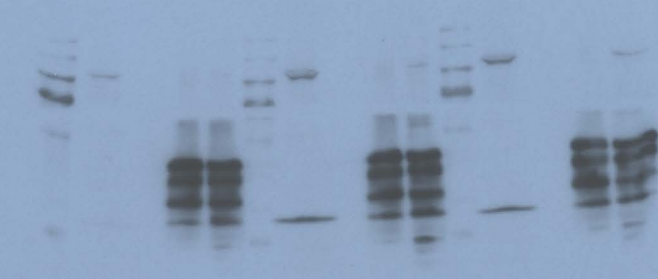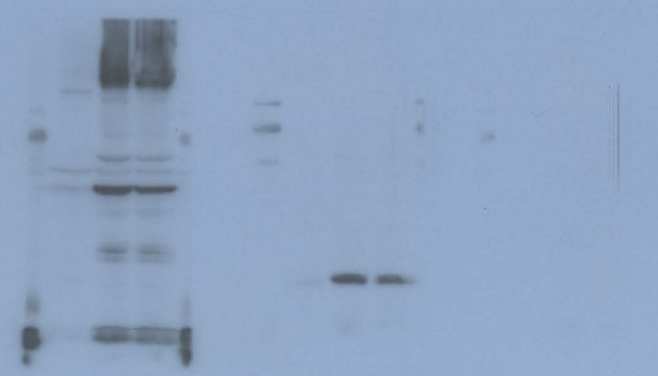

Supplement: Supplementary file 8 — Source Data [file 41467_2021_21529_MOESM8_ESM.zip › Uncropped blot and gel images/FigureS2/FigureS2j/GEMIN4.pdf]

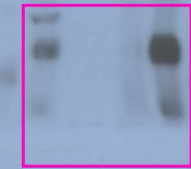

Fig.s2j.MePCE

Fig.s2j.hnRNP Q

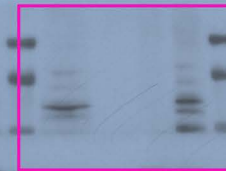

Supplement: Supplementary file 8 — Source Data [file 41467_2021_21529_MOESM8_ESM.zip › Uncropped blot and gel images/FigureS2/FigureS2j/MePCE_hnRNP Q.pdf]

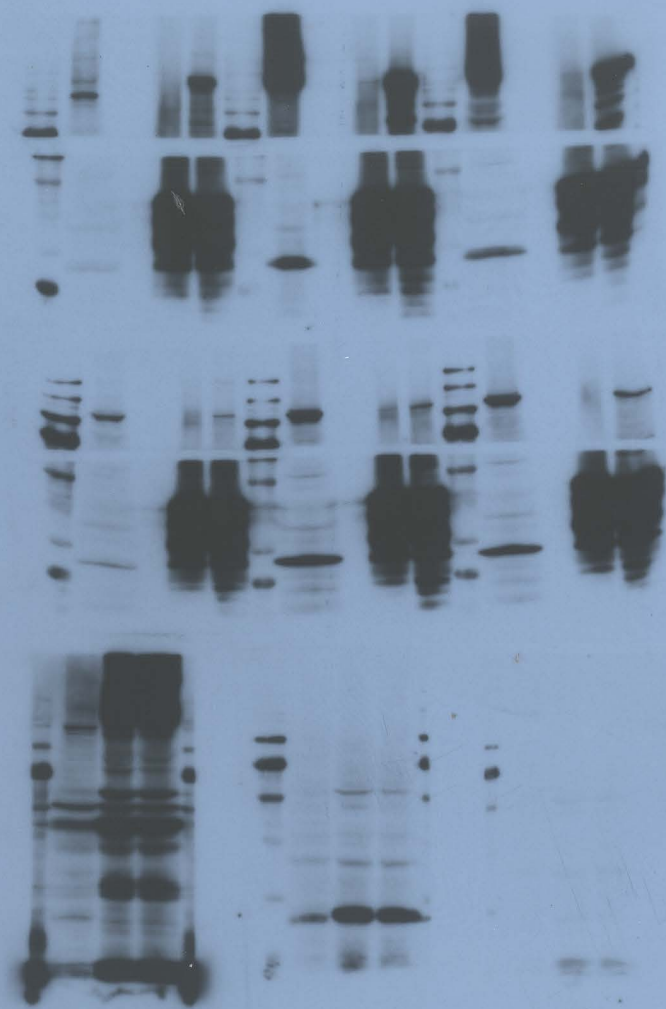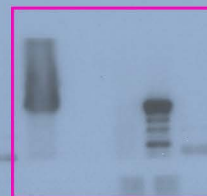

Fig.s2j.RHA

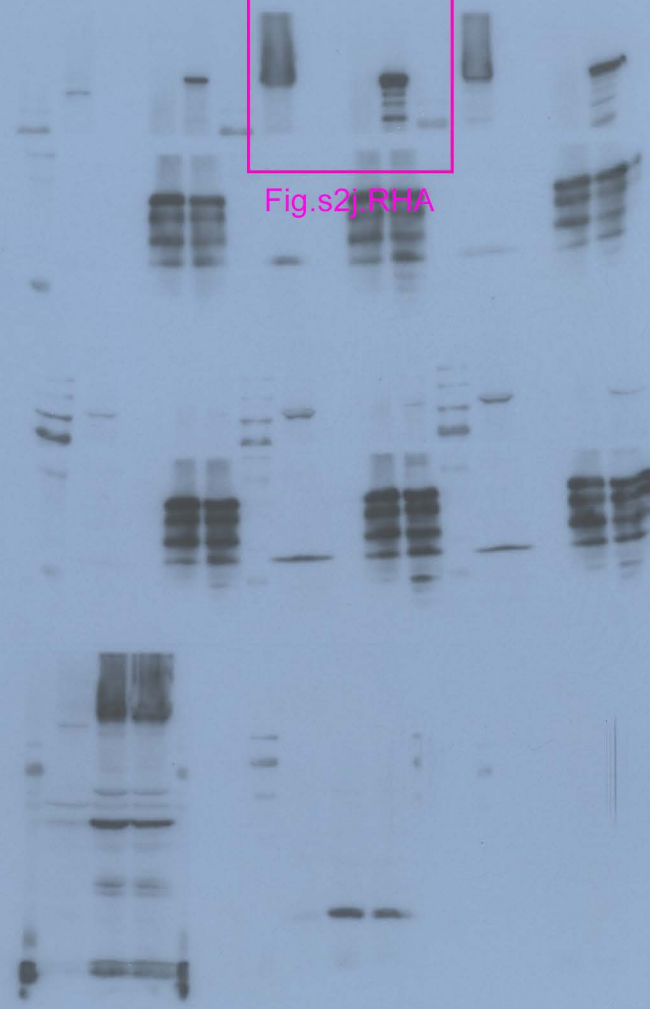

Supplement: Supplementary file 8 — Source Data [file 41467_2021_21529_MOESM8_ESM.zip › Uncropped blot and gel images/FigureS2/FigureS2j/RHA.pdf]

Fig.s2k.GEMIN4

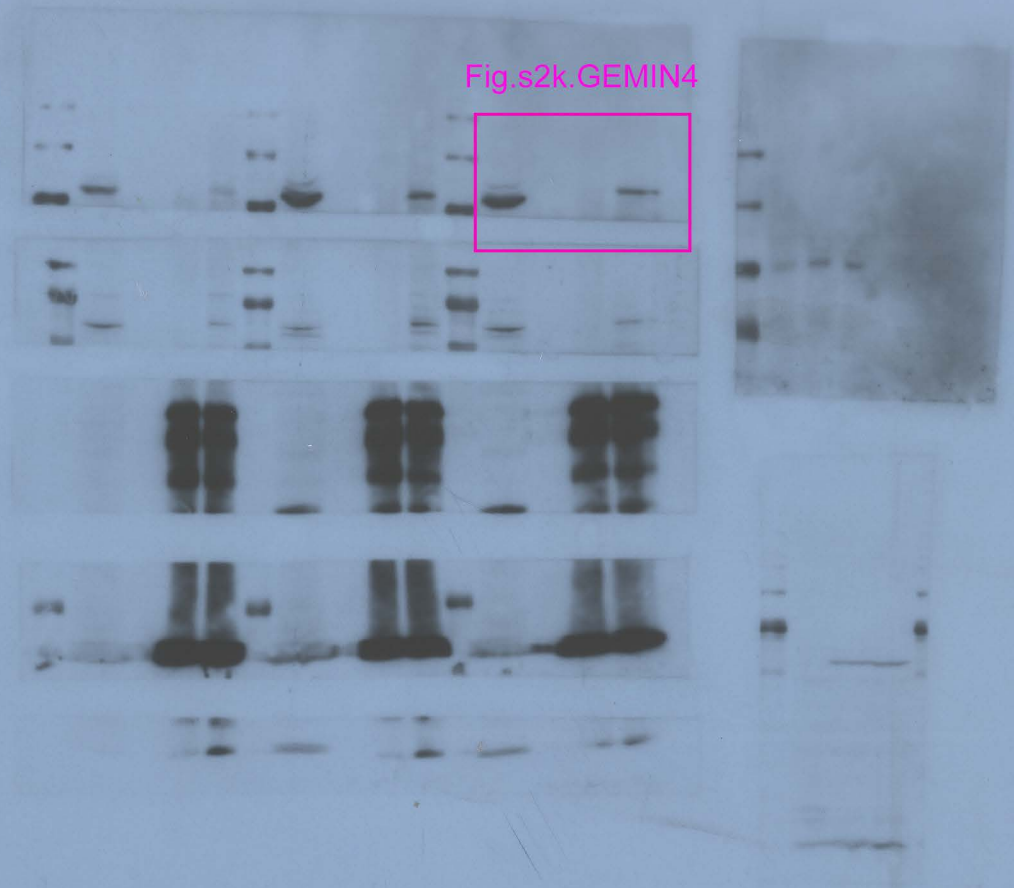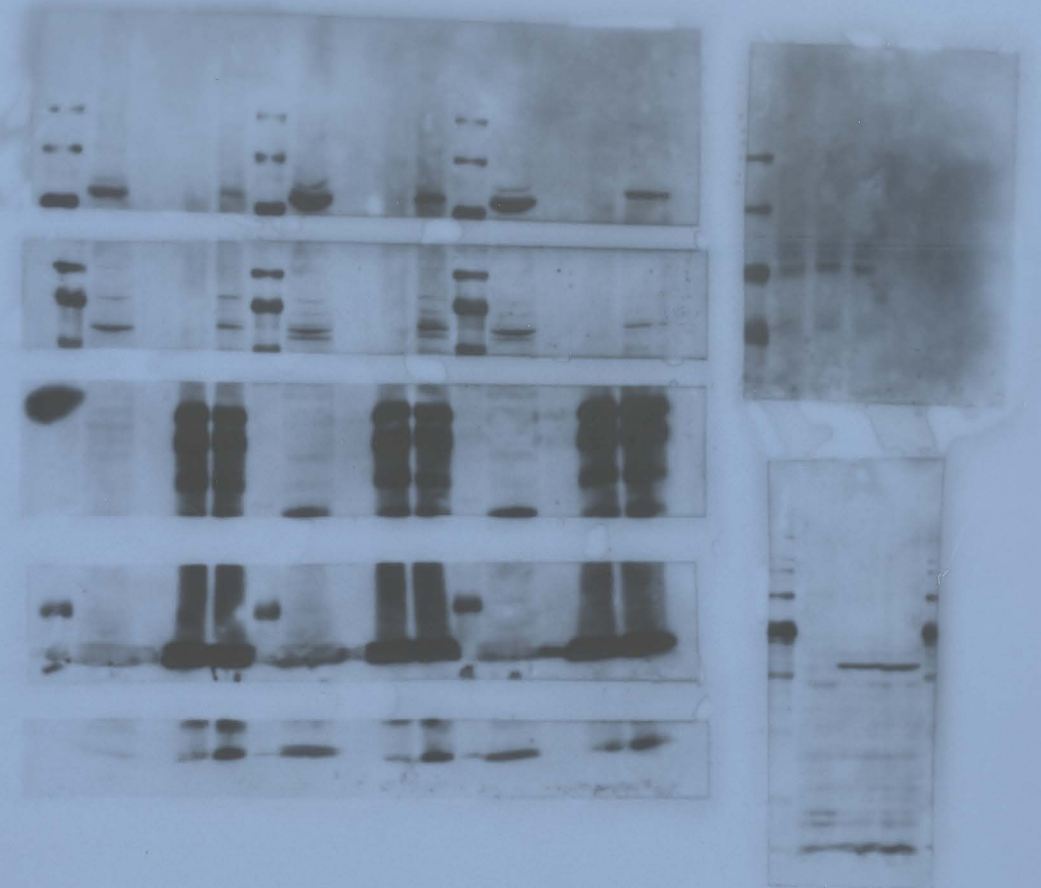

Supplement: Supplementary file 8 — Source Data [file 41467_2021_21529_MOESM8_ESM.zip › Uncropped blot and gel images/FigureS2/FigureS2k/GEMIN4.pdf]

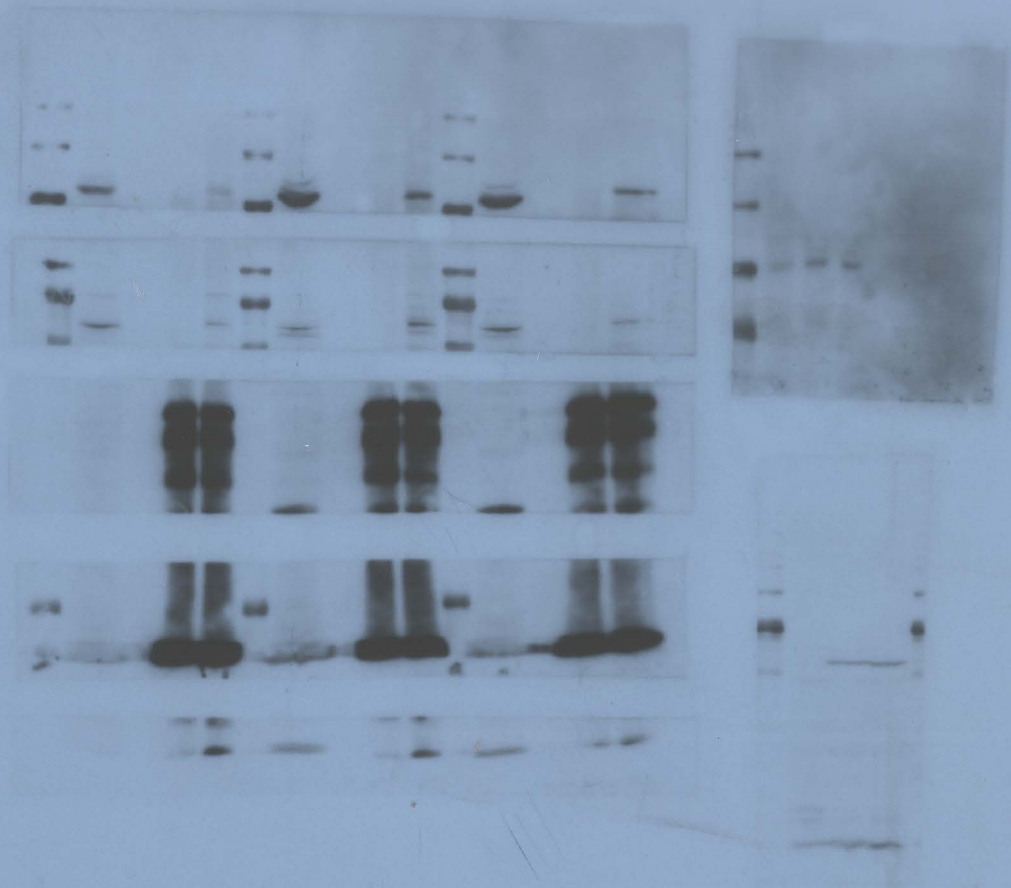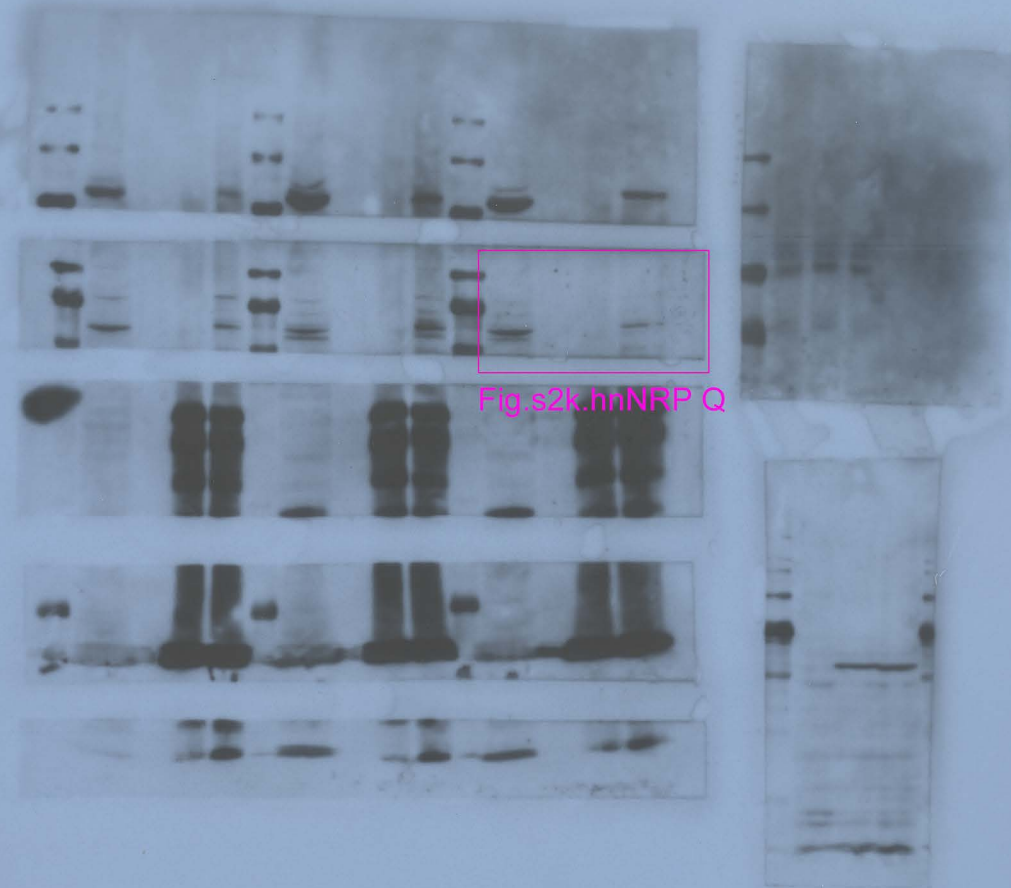

Fig.s2k.hnNRP Q

Supplement: Supplementary file 8 — Source Data [file 41467_2021_21529_MOESM8_ESM.zip › Uncropped blot and gel images/FigureS2/FigureS2k/hnRNP Q.pdf]

Fig.s2k.LARP7

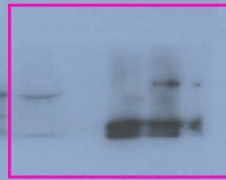

Supplement: Supplementary file 8 — Source Data [file 41467_2021_21529_MOESM8_ESM.zip › Uncropped blot and gel images/FigureS2/FigureS2k/LARP7.pdf]

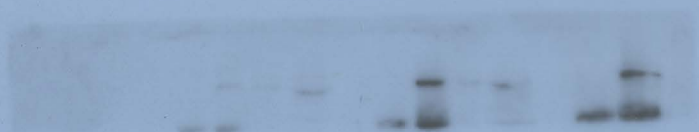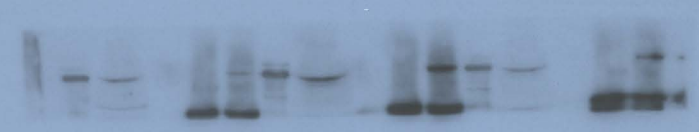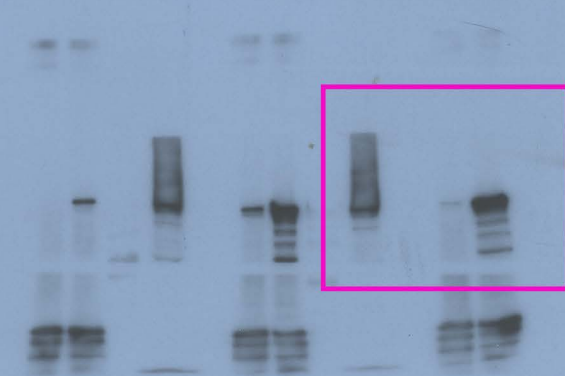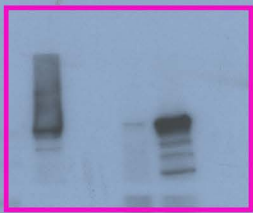

Fig.s2k.RHA

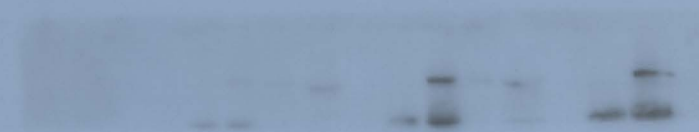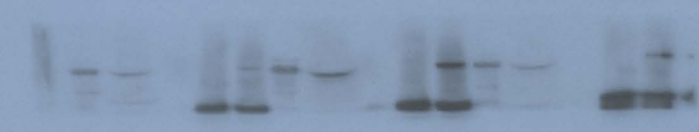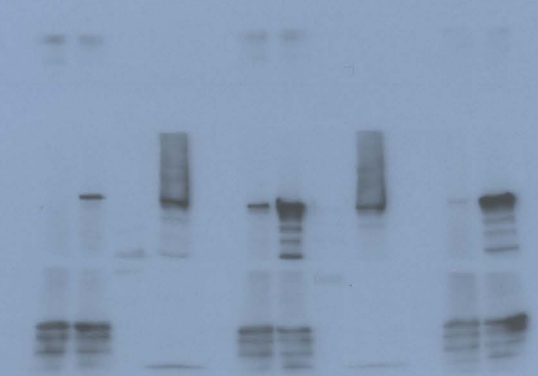

Supplement: Supplementary file 8 — Source Data [file 41467_2021_21529_MOESM8_ESM.zip › Uncropped blot and gel images/FigureS2/FigureS2k/RHA.pdf]

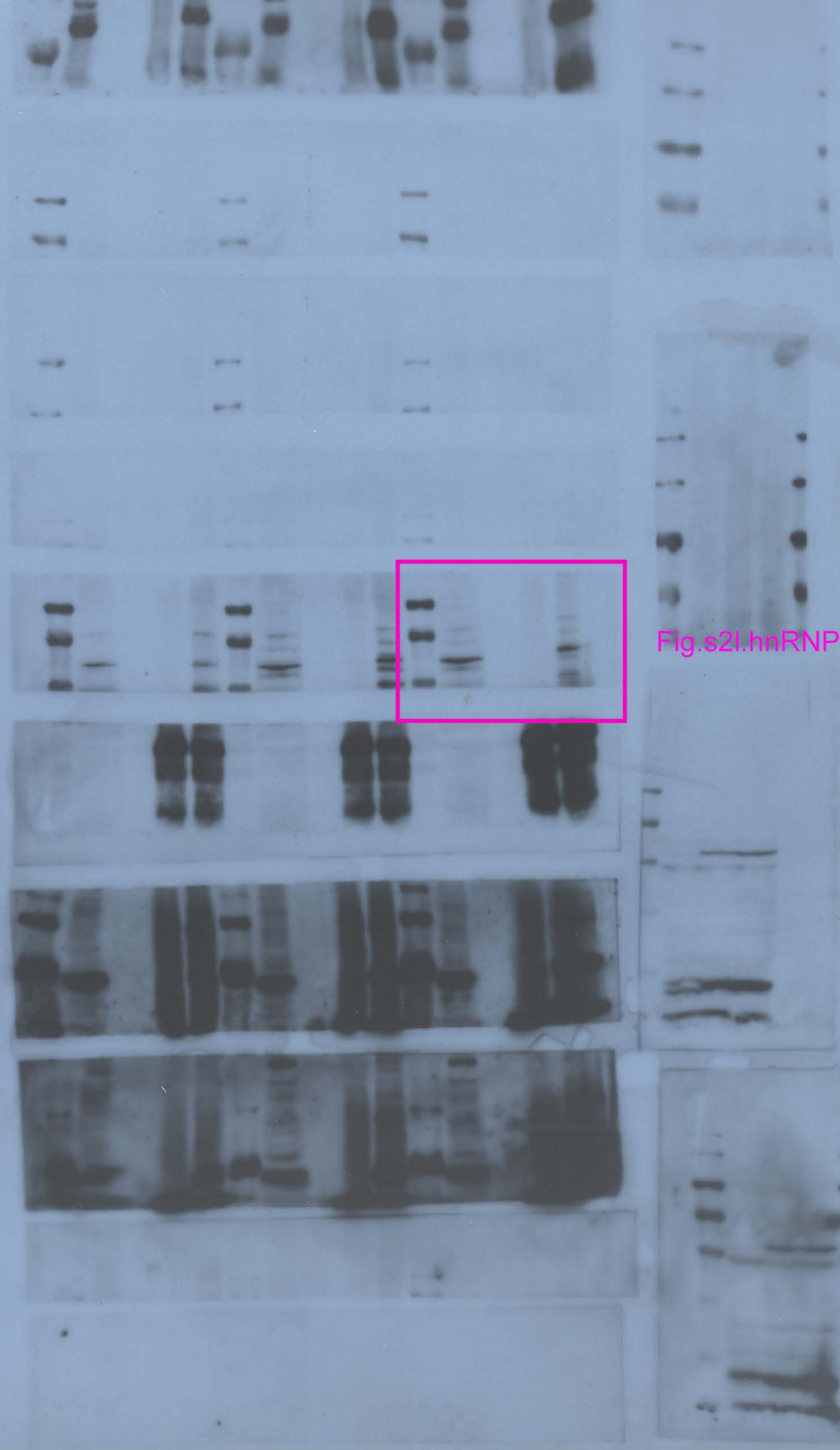

Fig.s21.hnRNP Q

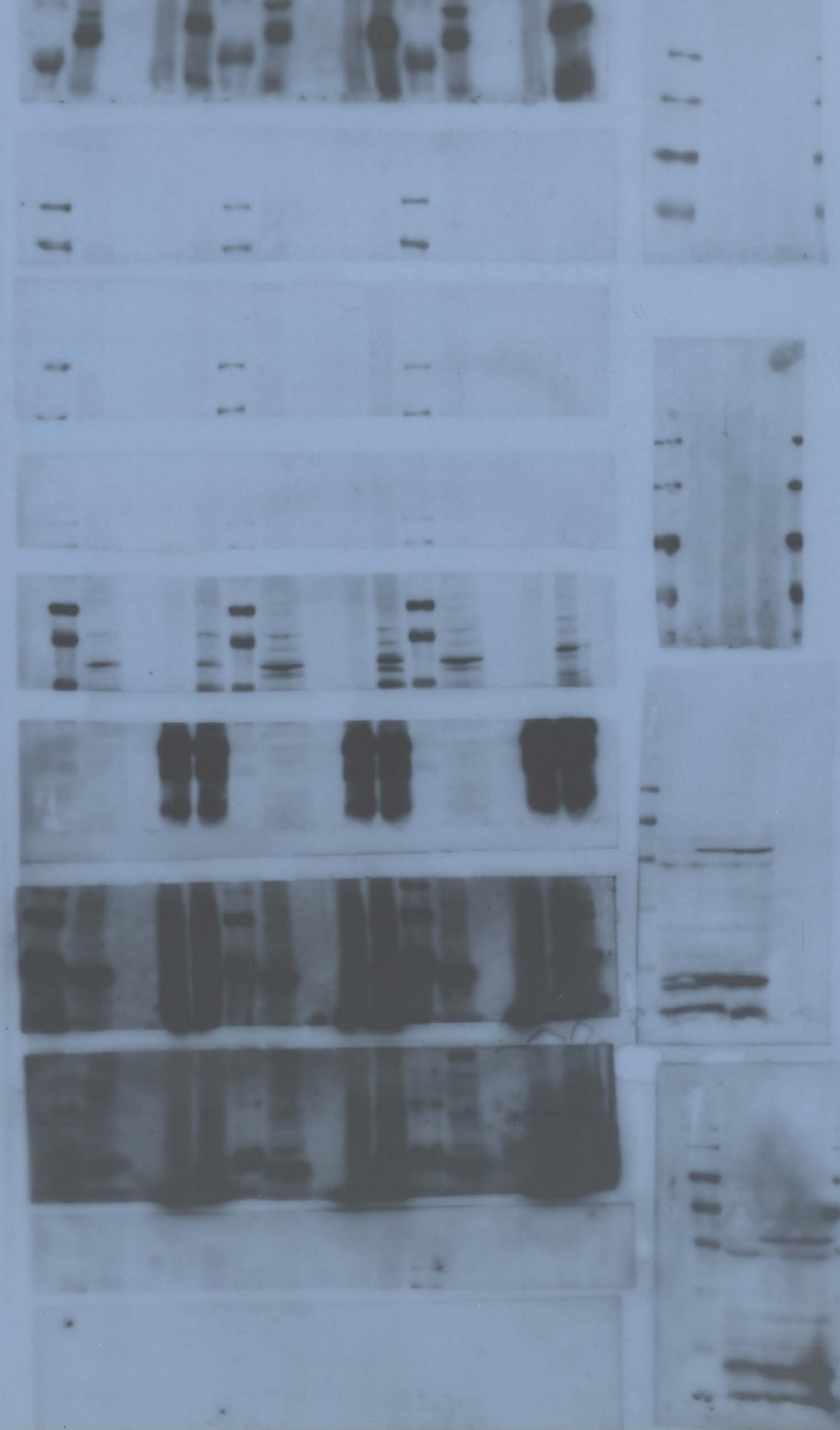

Supplement: Supplementary file 8 — Source Data [file 41467_2021_21529_MOESM8_ESM.zip › Uncropped blot and gel images/FigureS2/FigureS2l/hnRNP Q.pdf]

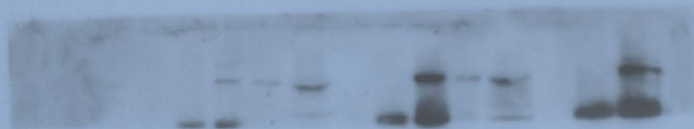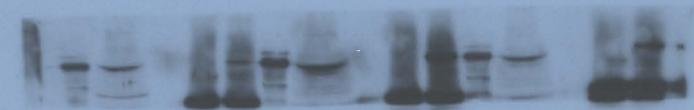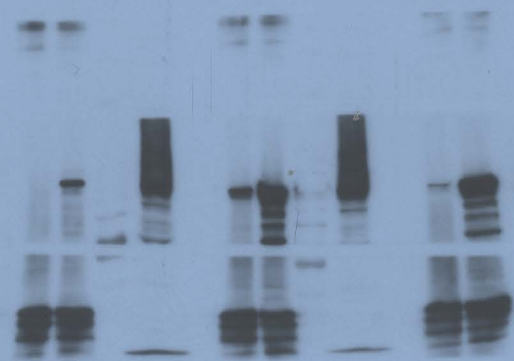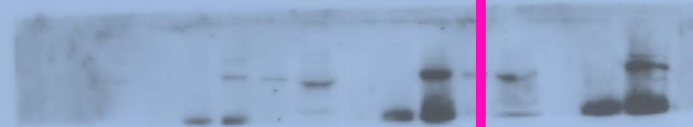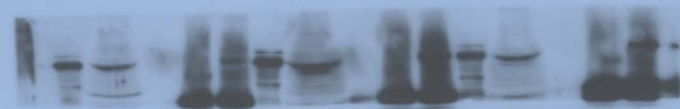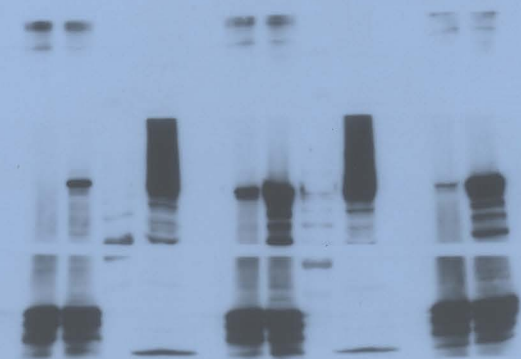

Fig.s2I.LARP7

Supplement: Supplementary file 8 — Source Data [file 41467_2021_21529_MOESM8_ESM.zip › Uncropped blot and gel images/FigureS2/FigureS2l/LARP7.pdf]

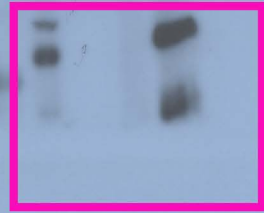

Fig.s2l.MePCE

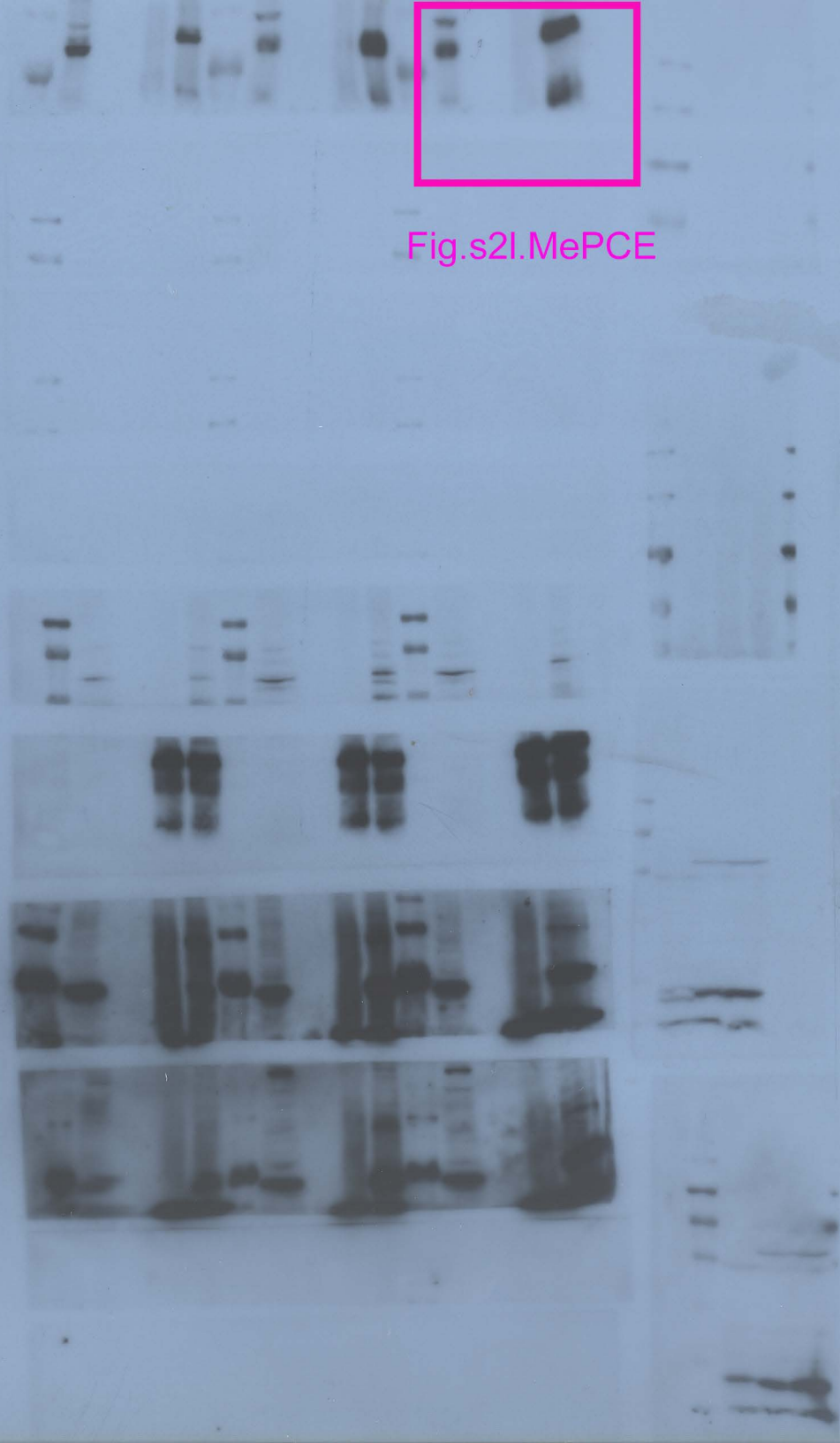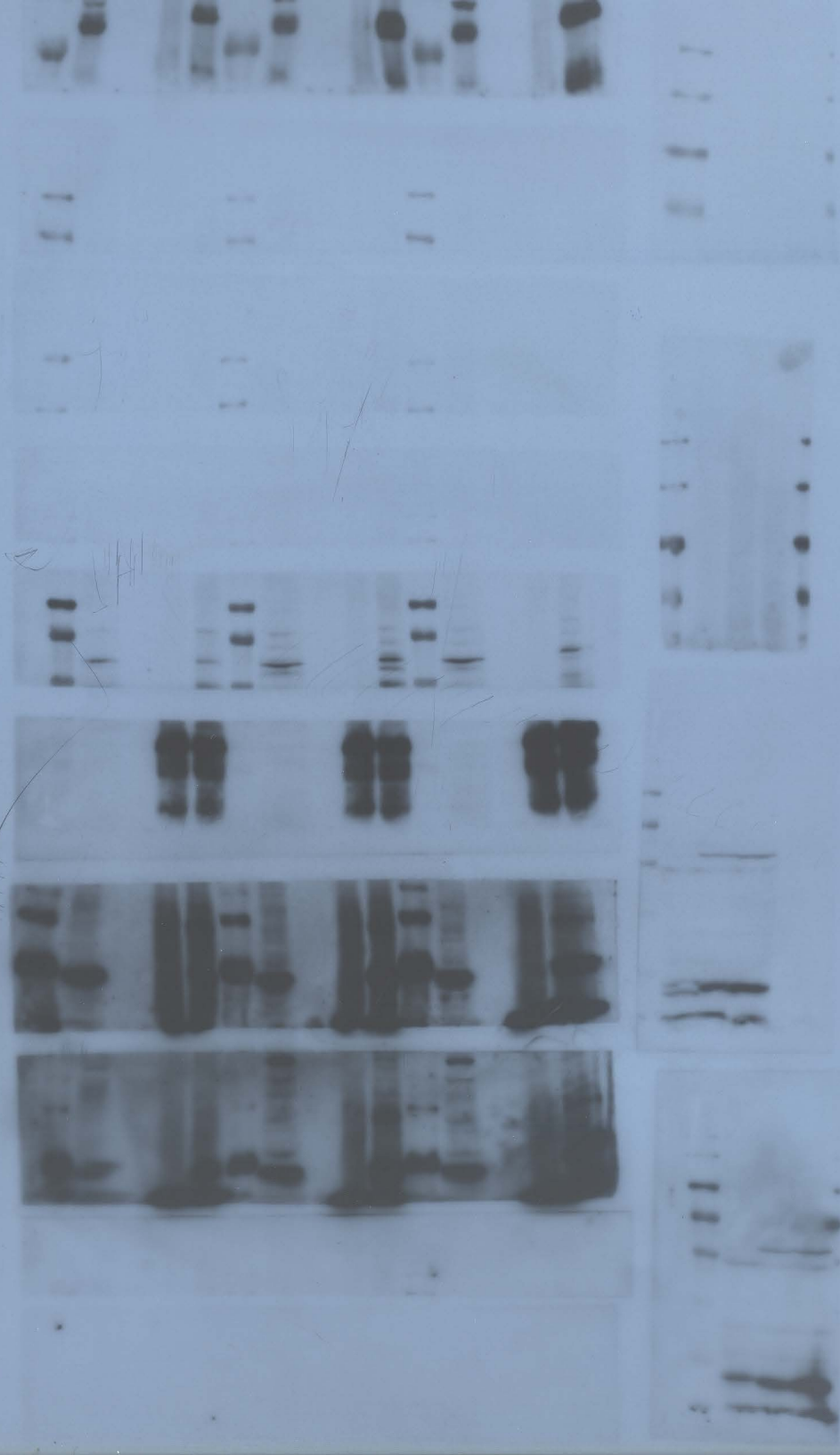

Supplement: Supplementary file 8 — Source Data [file 41467_2021_21529_MOESM8_ESM.zip › Uncropped blot and gel images/FigureS2/FigureS2l/MePCE.pdf]

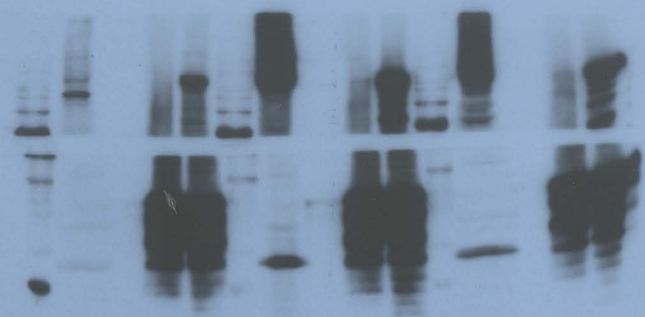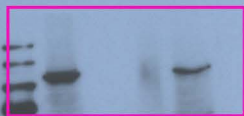

Fig.s2I.GEMIN4

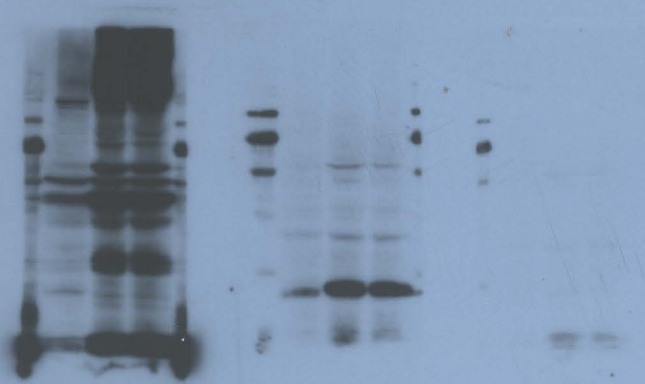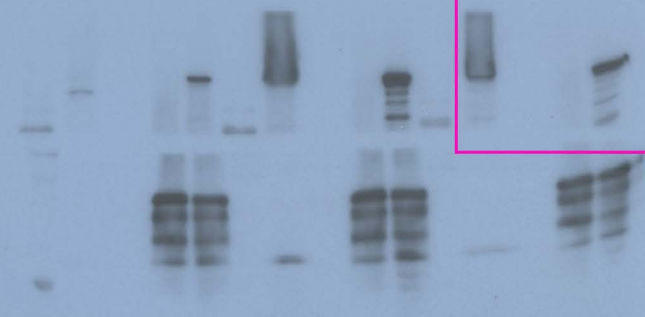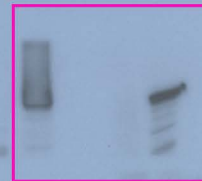

Fig.s2I.RHA

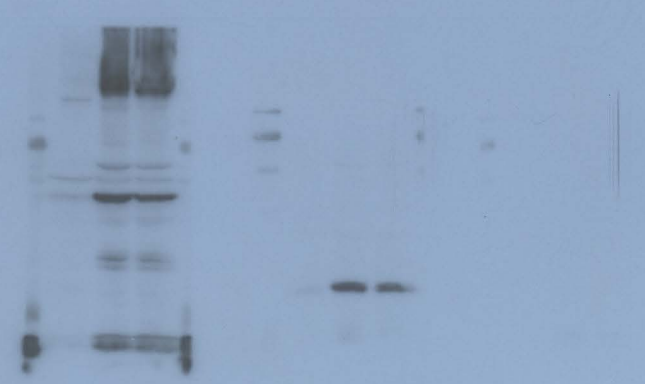

Supplement: Supplementary file 8 — Source Data [file 41467_2021_21529_MOESM8_ESM.zip › Uncropped blot and gel images/FigureS2/FigureS2l/RHA_GEMIN4.pdf]

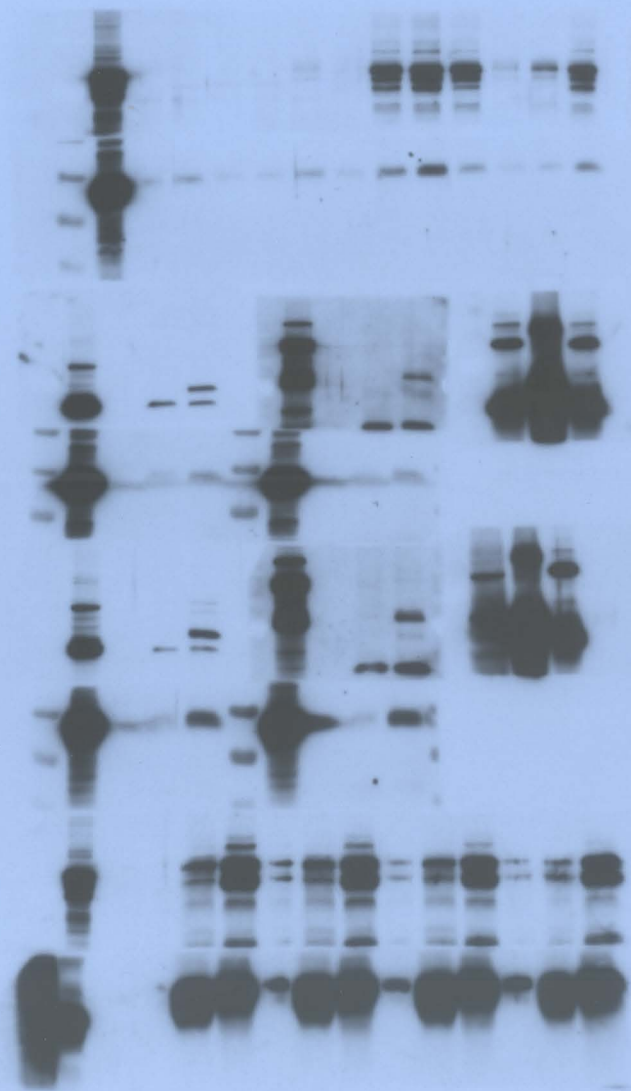

..FUJI•HRC•(SAFETY)••

..FUJI•HRC•(SAFETY)••

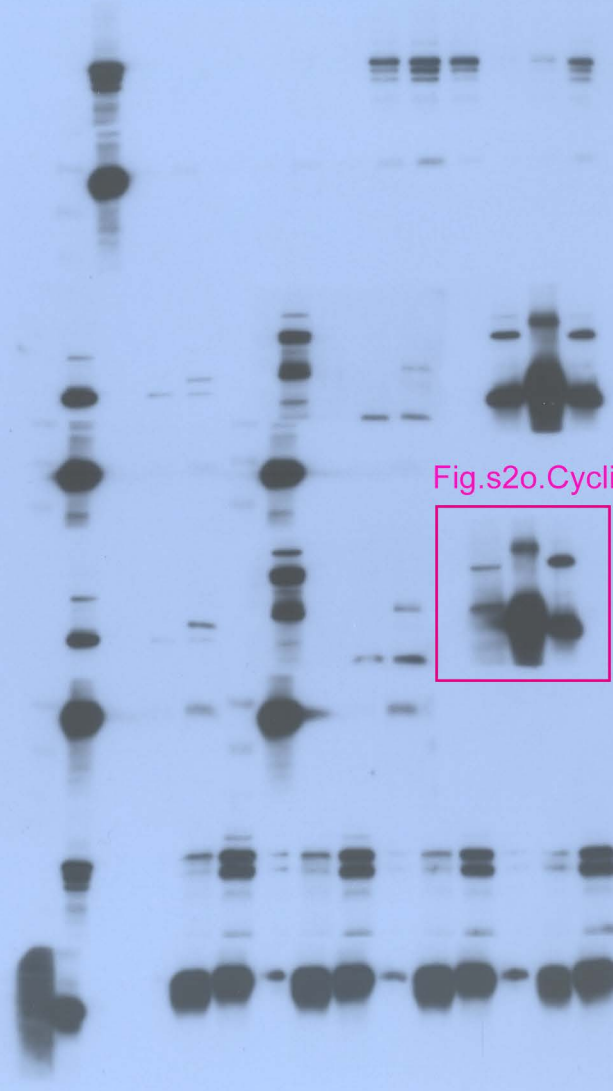

Fig.s2o.CyclinT1

..FUJI•HRC•(SAFETY)••

..FUJI•HRC•(SAFETY)••

Supplement: Supplementary file 8 — Source Data [file 41467_2021_21529_MOESM8_ESM.zip › Uncropped blot and gel images/FigureS2/FigureS2o/CyclinT1.pdf]

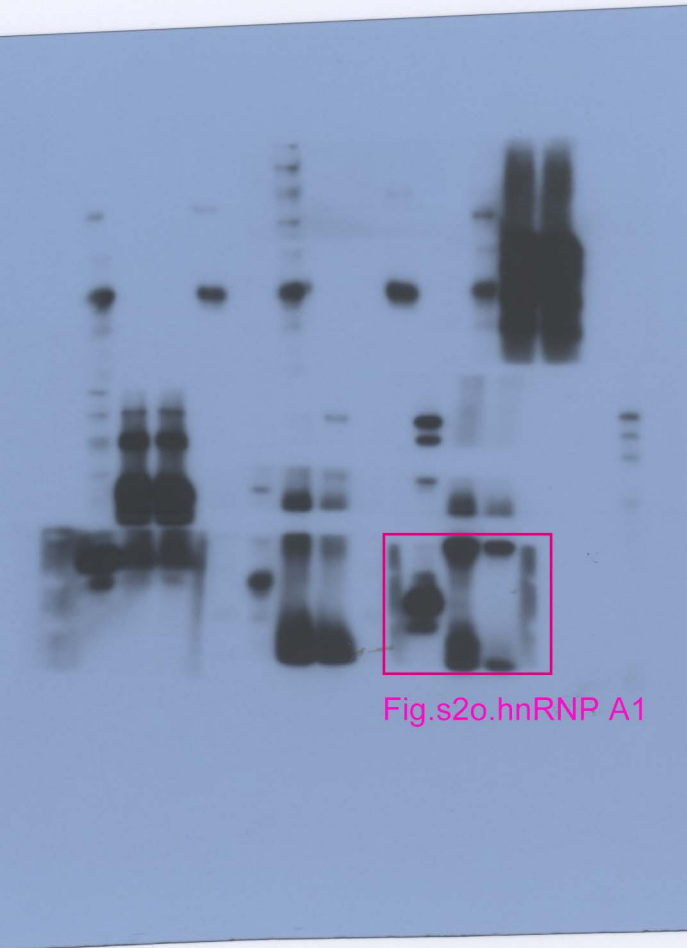

Fig.s2o.hnRNP A1

Supplement: Supplementary file 8 — Source Data [file 41467_2021_21529_MOESM8_ESM.zip › Uncropped blot and gel images/FigureS2/FigureS2o/hnRNP A1.pdf]

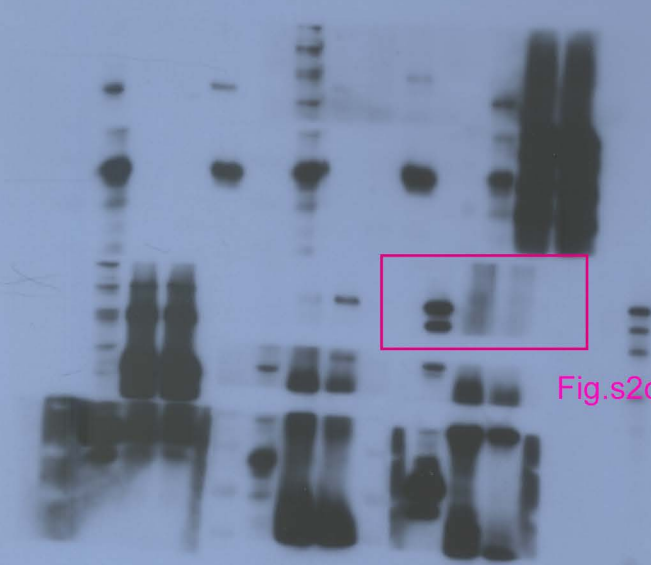

Fig.s2o.hnRNP R

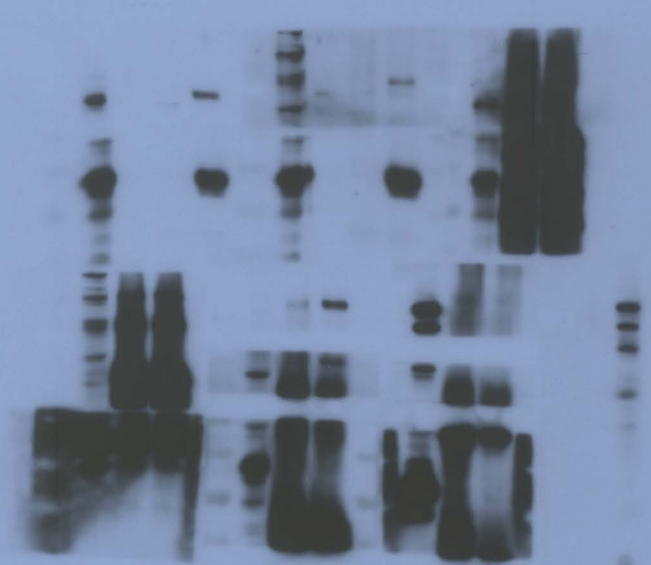

Supplement: Supplementary file 8 — Source Data [file 41467_2021_21529_MOESM8_ESM.zip › Uncropped blot and gel images/FigureS2/FigureS2o/hnRNP R.pdf]

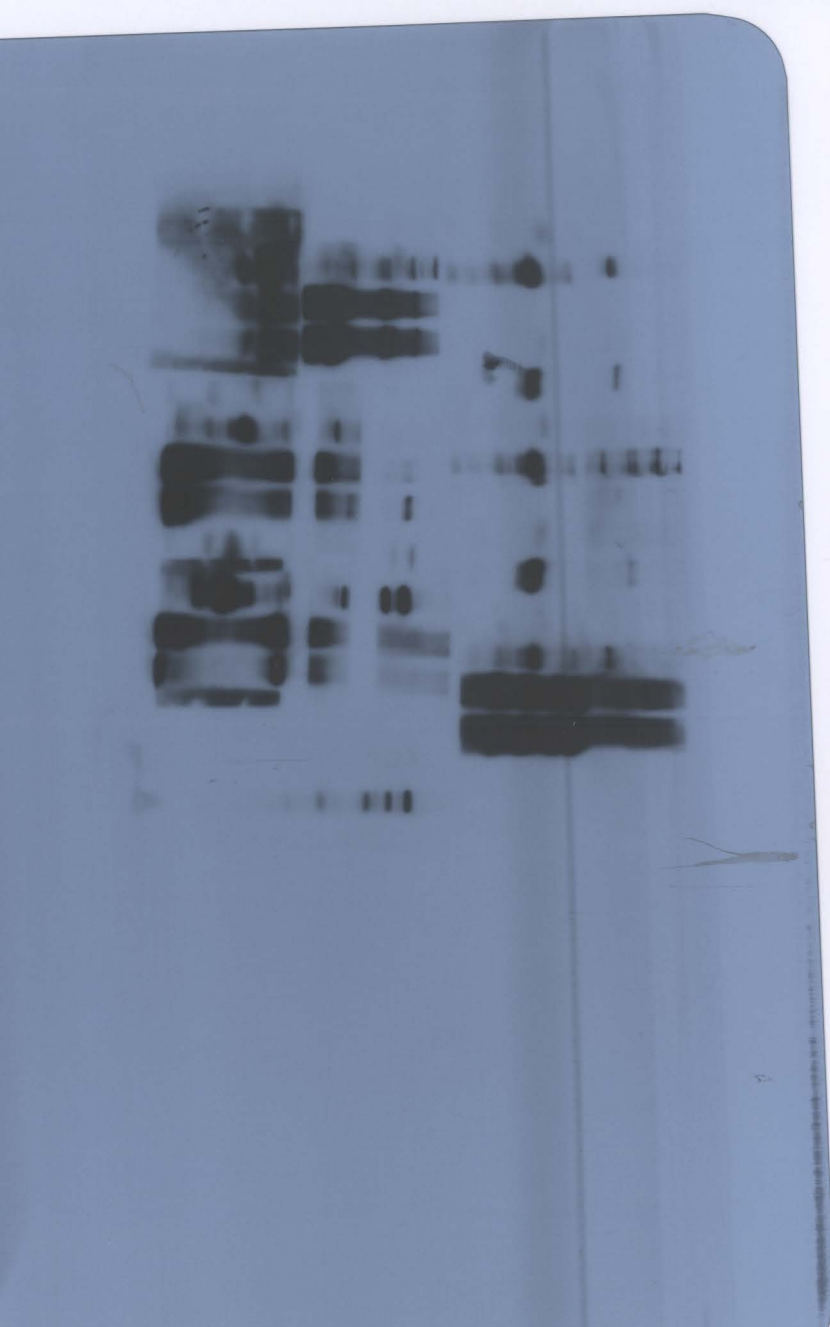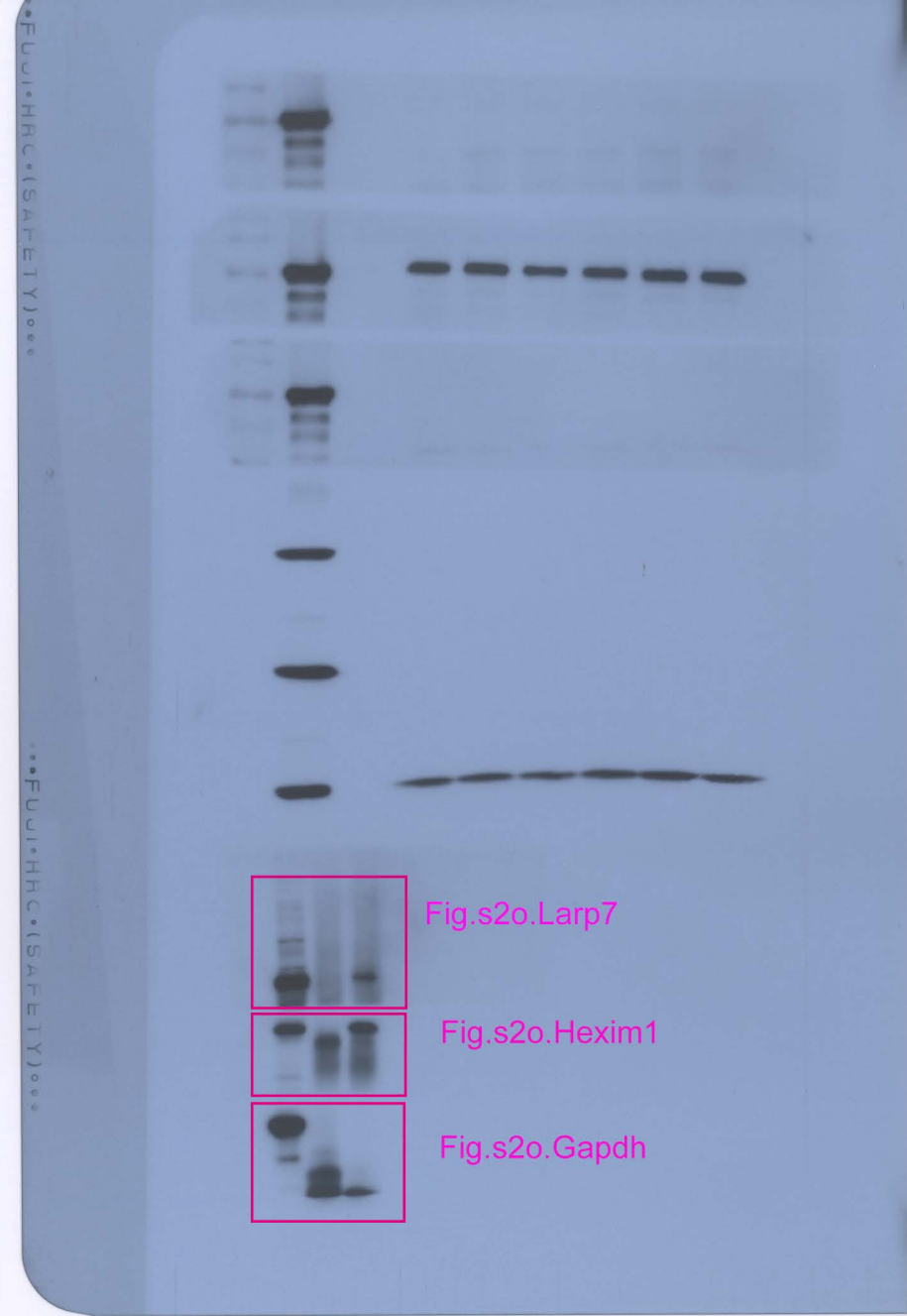

Supplement: Supplementary file 8 — Source Data [file 41467_2021_21529_MOESM8_ESM.zip › Uncropped blot and gel images/FigureS2/FigureS2o/Larp7_Hexim1_Gapdh.pdf]

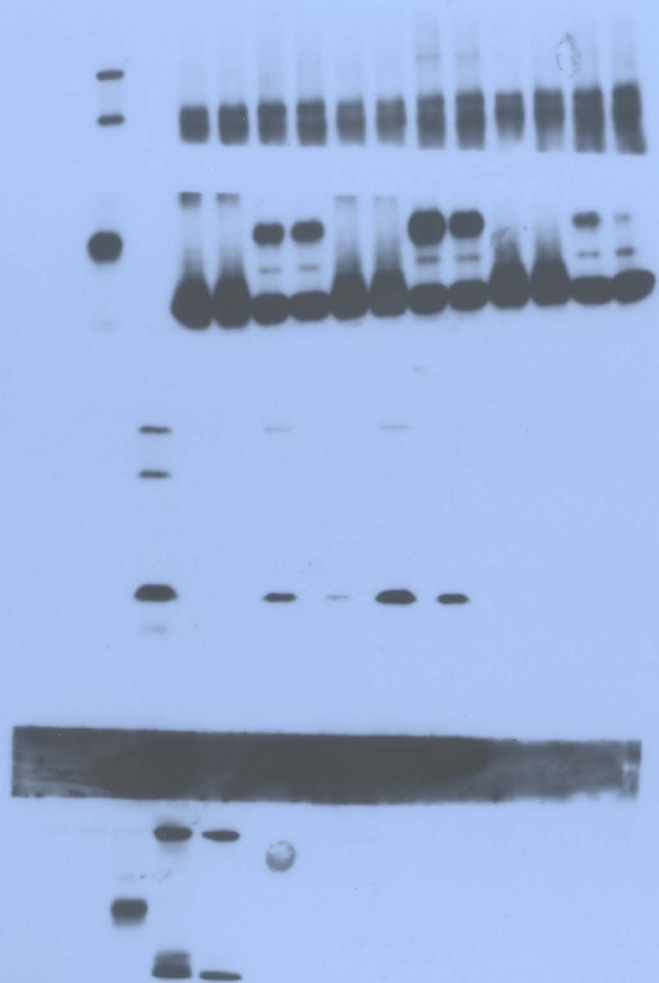

...E0011•HBC•(2VFE1A)•••

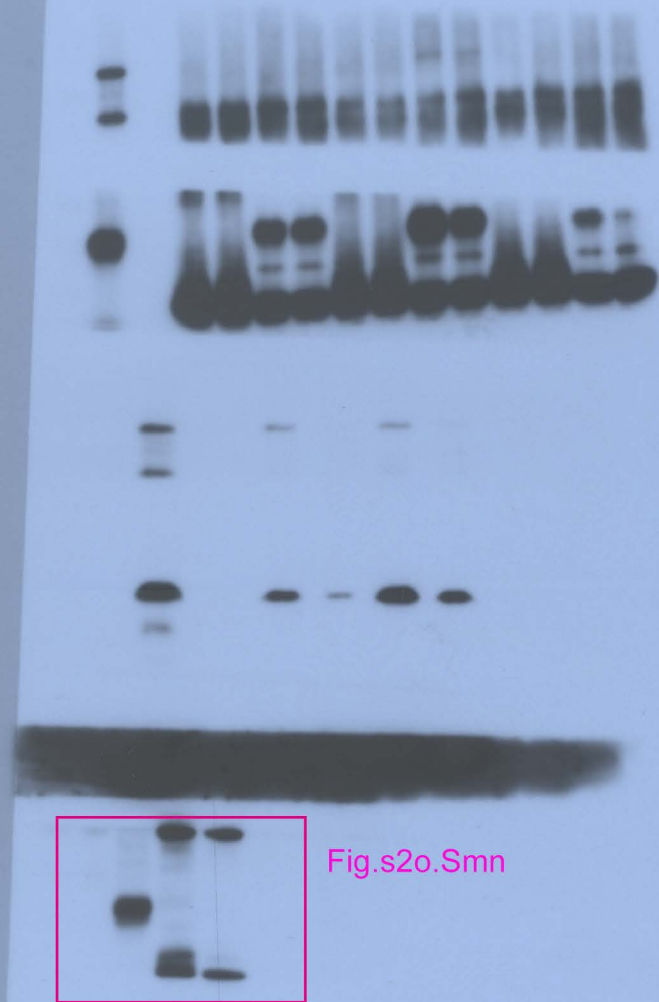

Fig.s2o.Smn

Supplement: Supplementary file 8 — Source Data [file 41467_2021_21529_MOESM8_ESM.zip › Uncropped blot and gel images/FigureS2/FigureS2o/Smn.pdf]

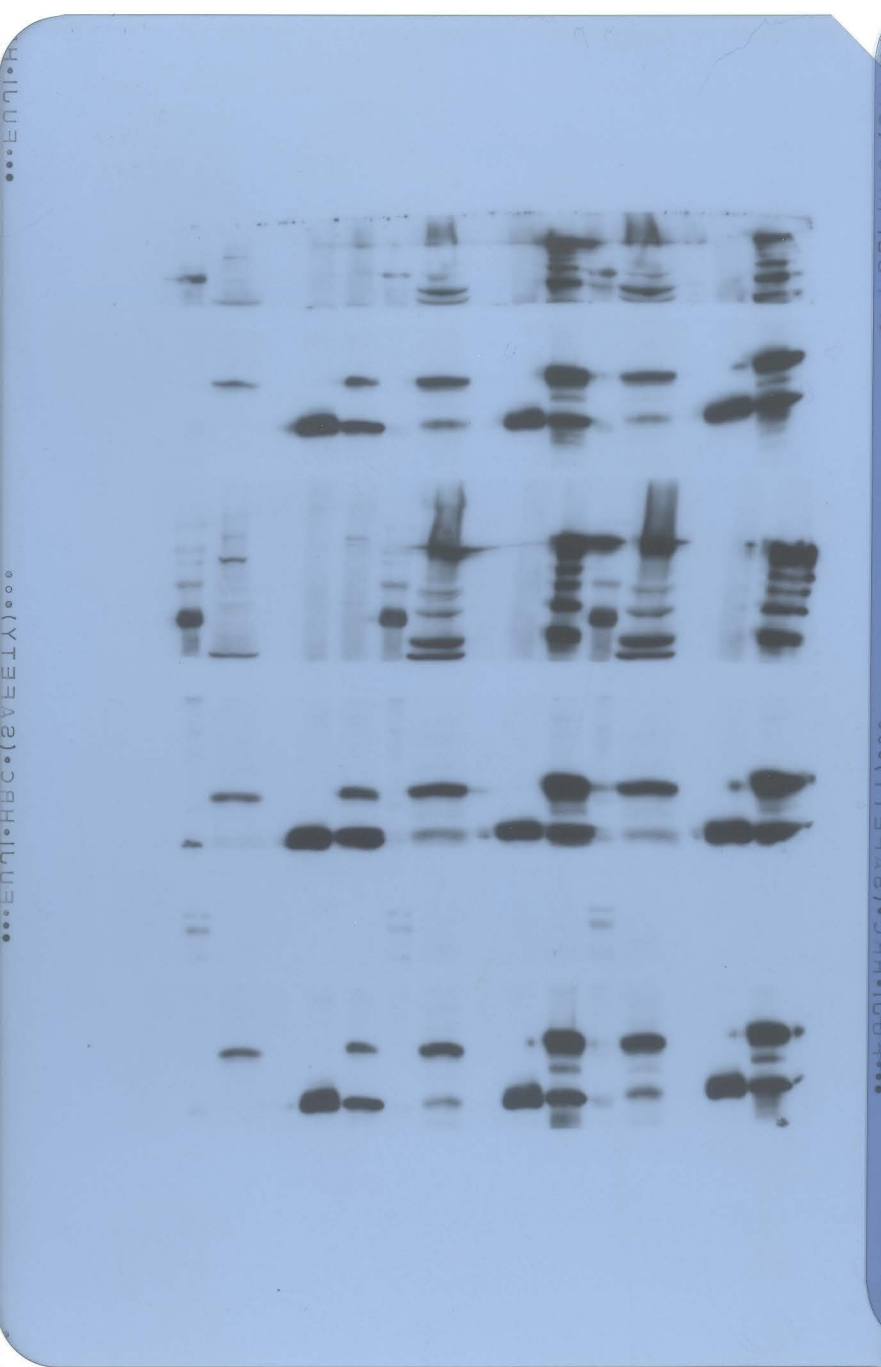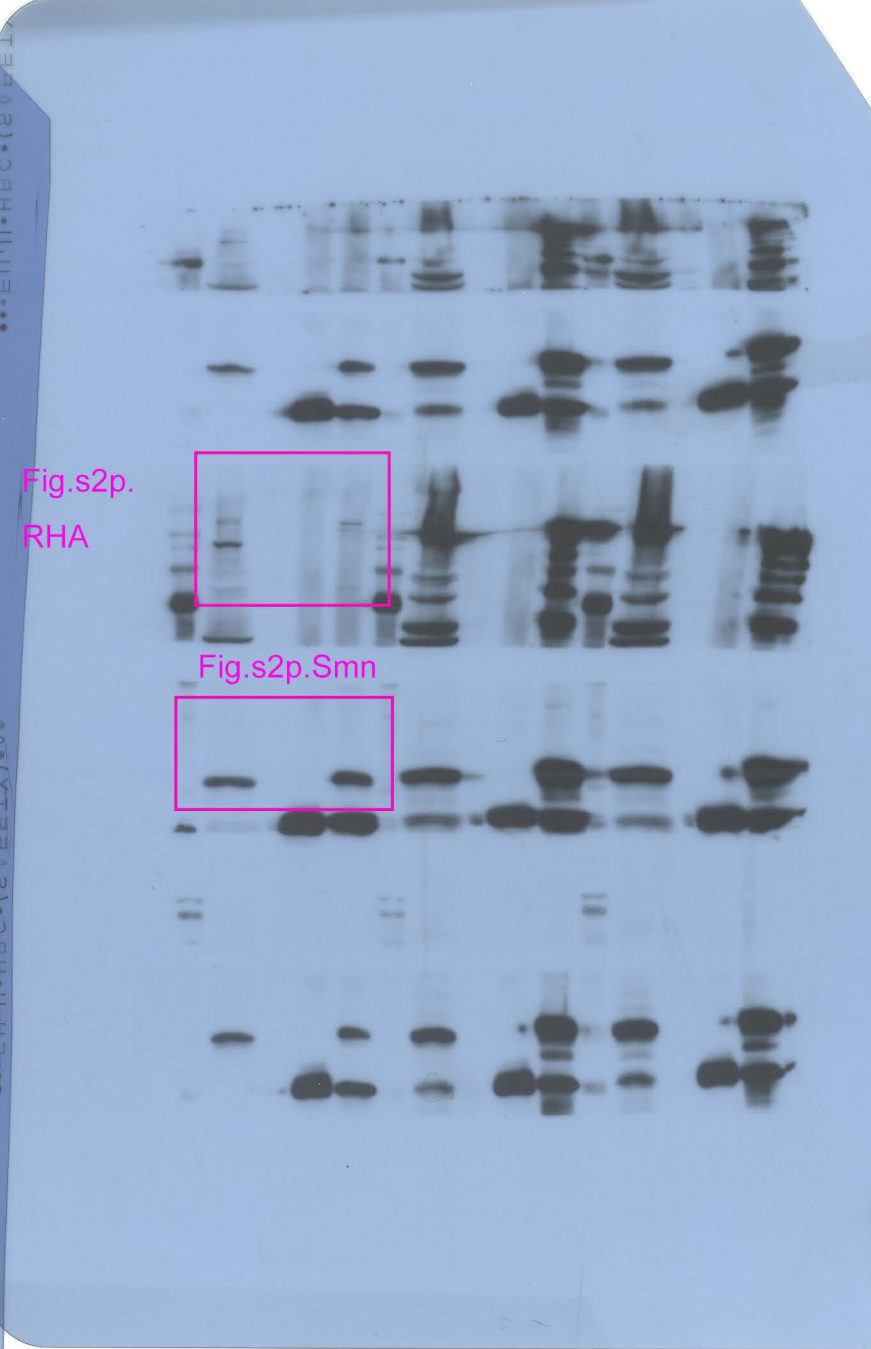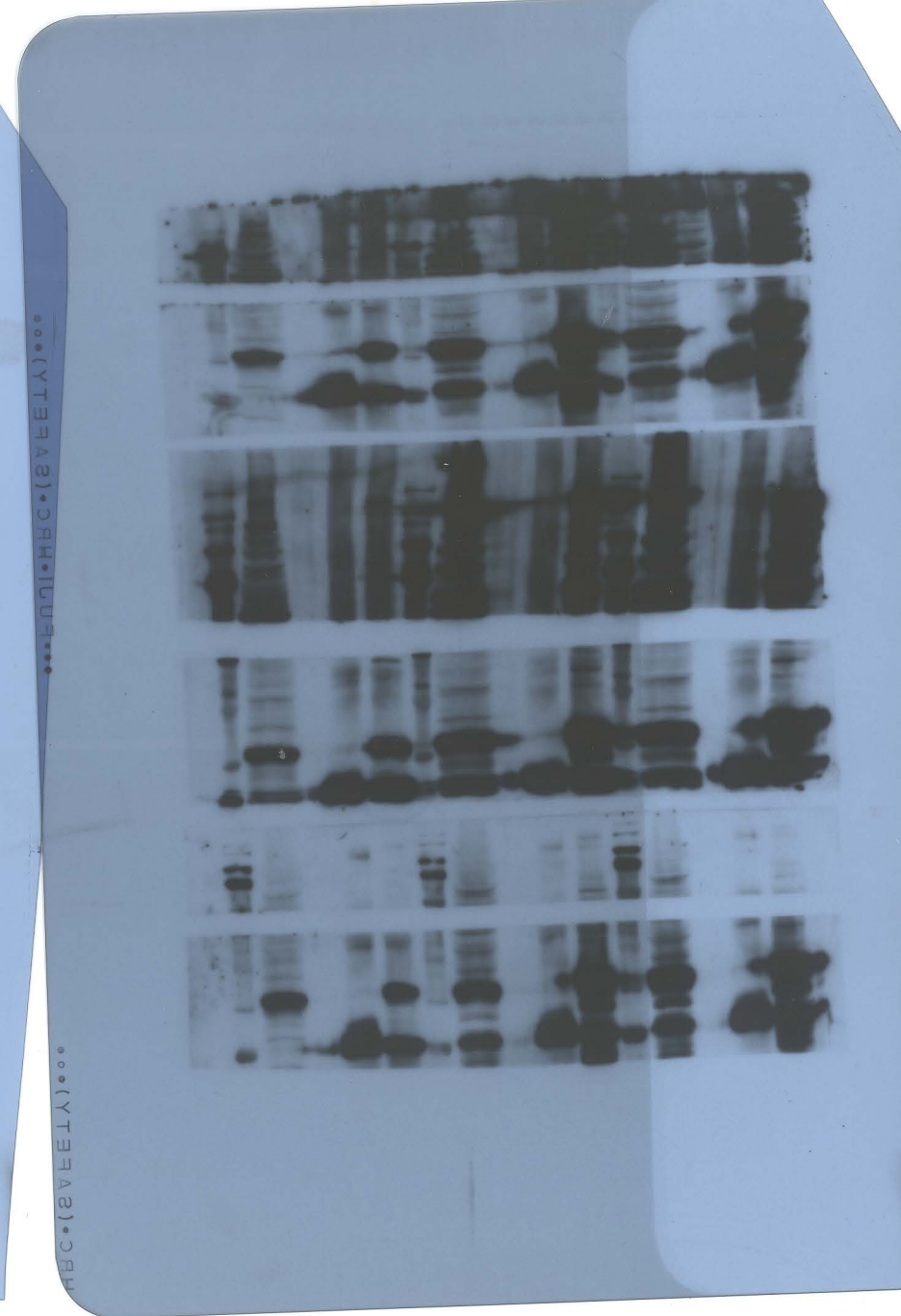

Supplement: Supplementary file 8 — Source Data [file 41467_2021_21529_MOESM8_ESM.zip › Uncropped blot and gel images/FigureS2/FigureS2p/Smn_RHA.pdf]

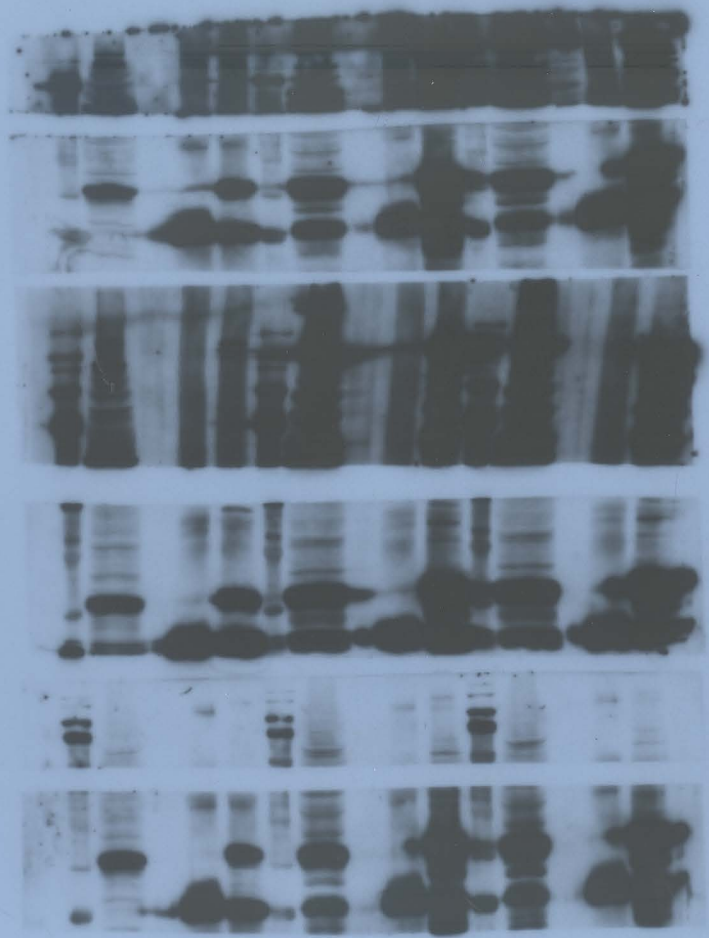

... (YTTAA2) • CPH • LU3 ...

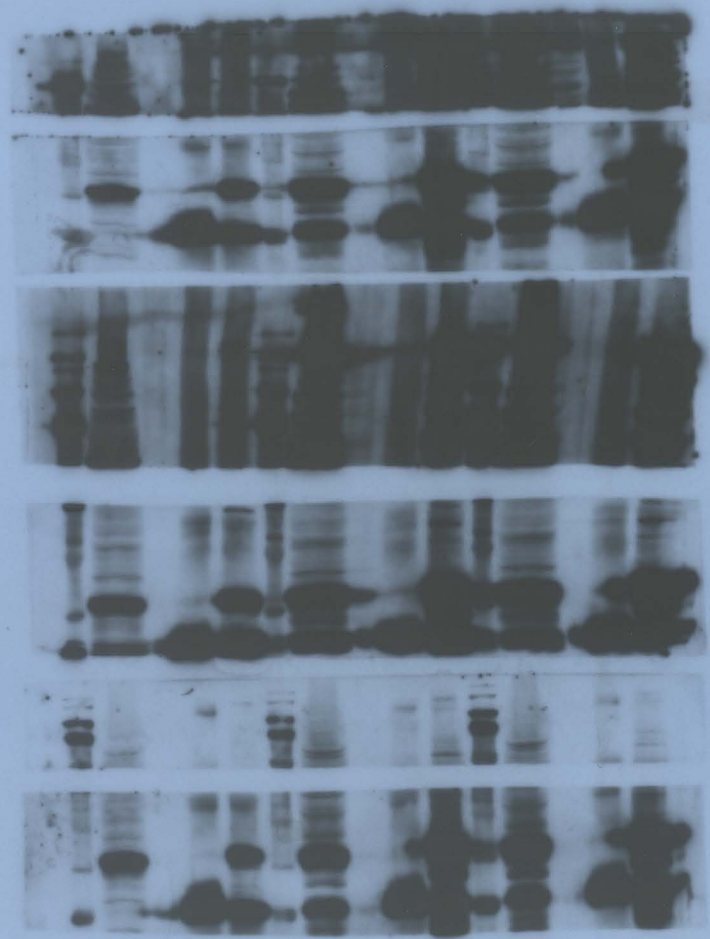

... (YTTAA2) • CPH • LU3 ...

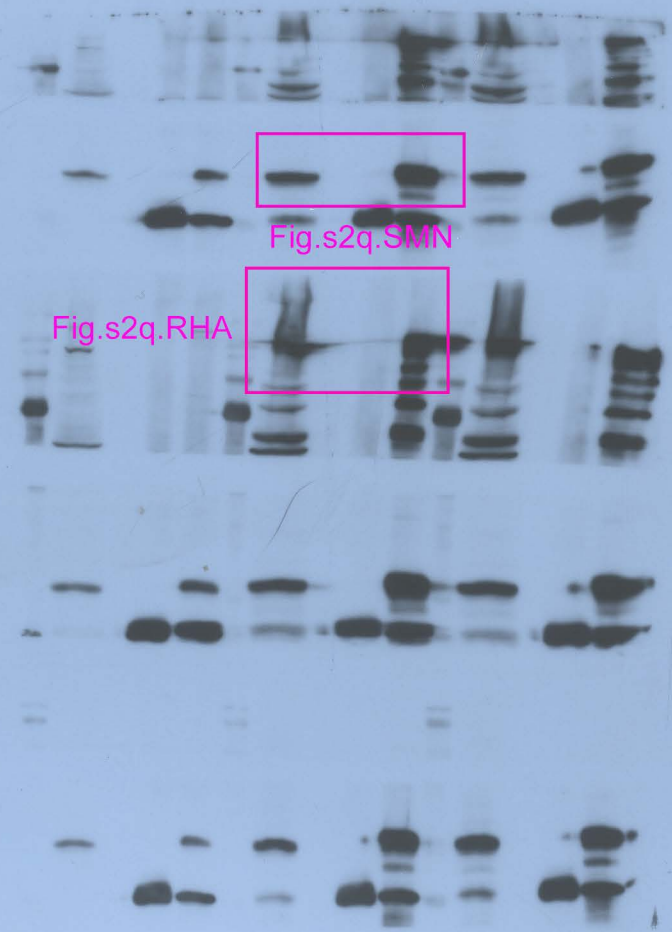

Fig.s2q.SMN

Fig.s2q.RHA

Supplement: Supplementary file 8 — Source Data [file 41467_2021_21529_MOESM8_ESM.zip › Uncropped blot and gel images/FigureS2/FigureS2q/SMN_RHA.pdf]

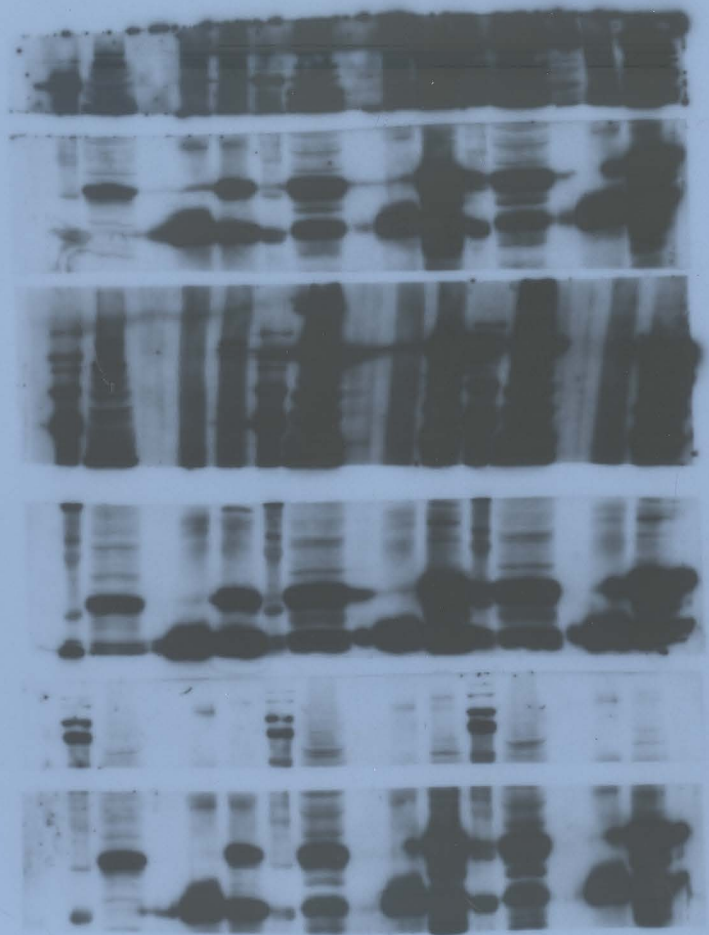

... (YTHA2) • CPH • LU • ...

... (YTHA2) • CPH • LU • ...

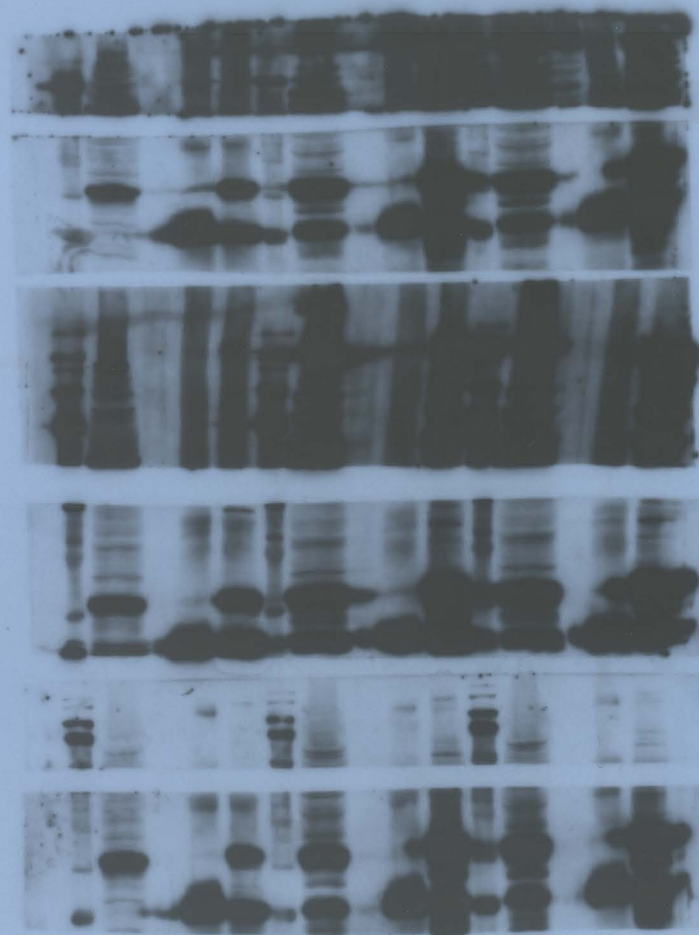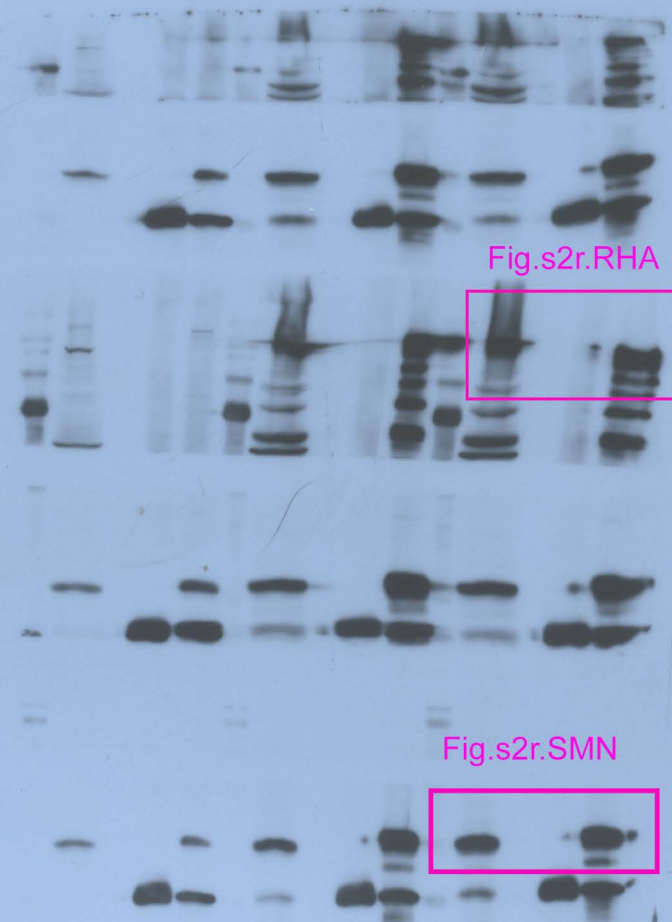

Fig.s2r.RHA

Fig.s2r.SMN

Supplement: Supplementary file 8 — Source Data [file 41467_2021_21529_MOESM8_ESM.zip › Uncropped blot and gel images/FigureS2/FigureS2r/SMN_RHA.pdf]

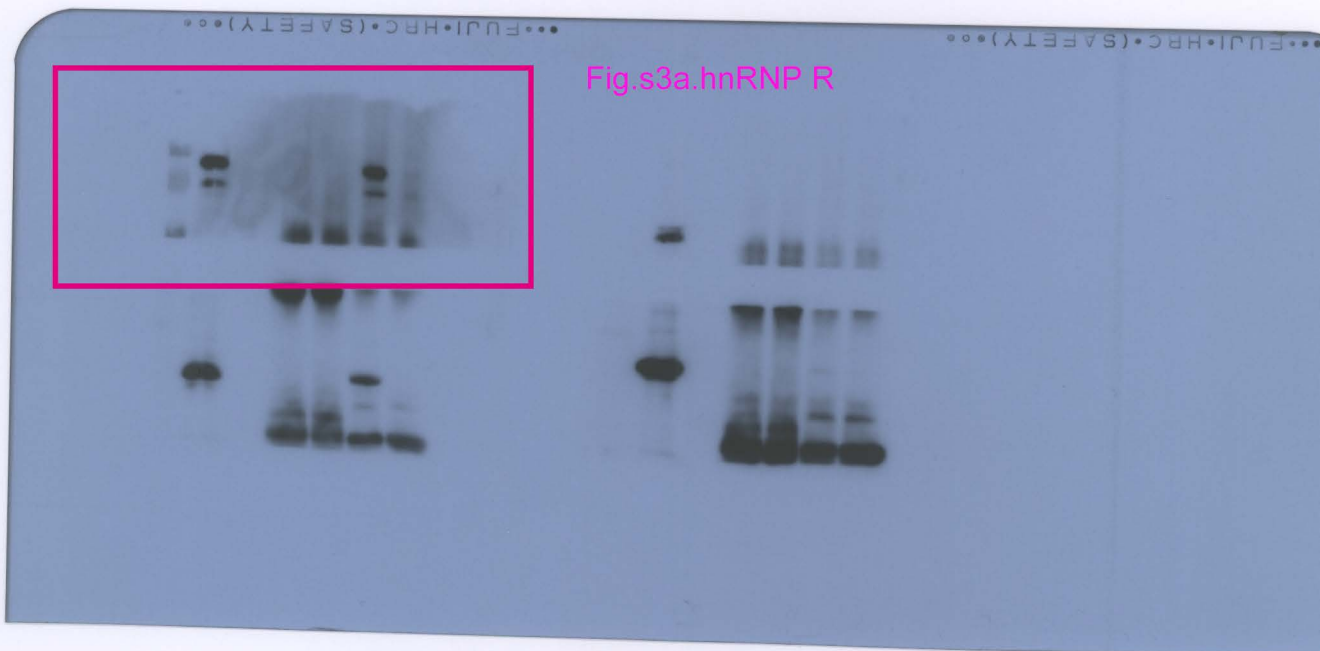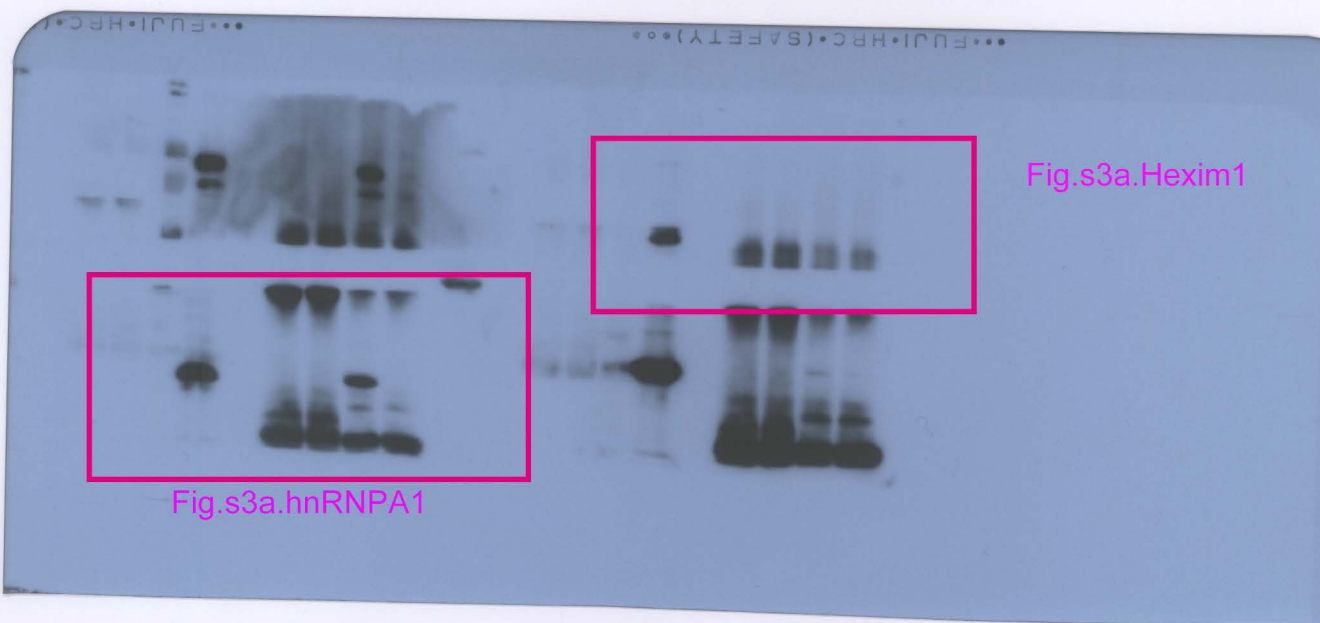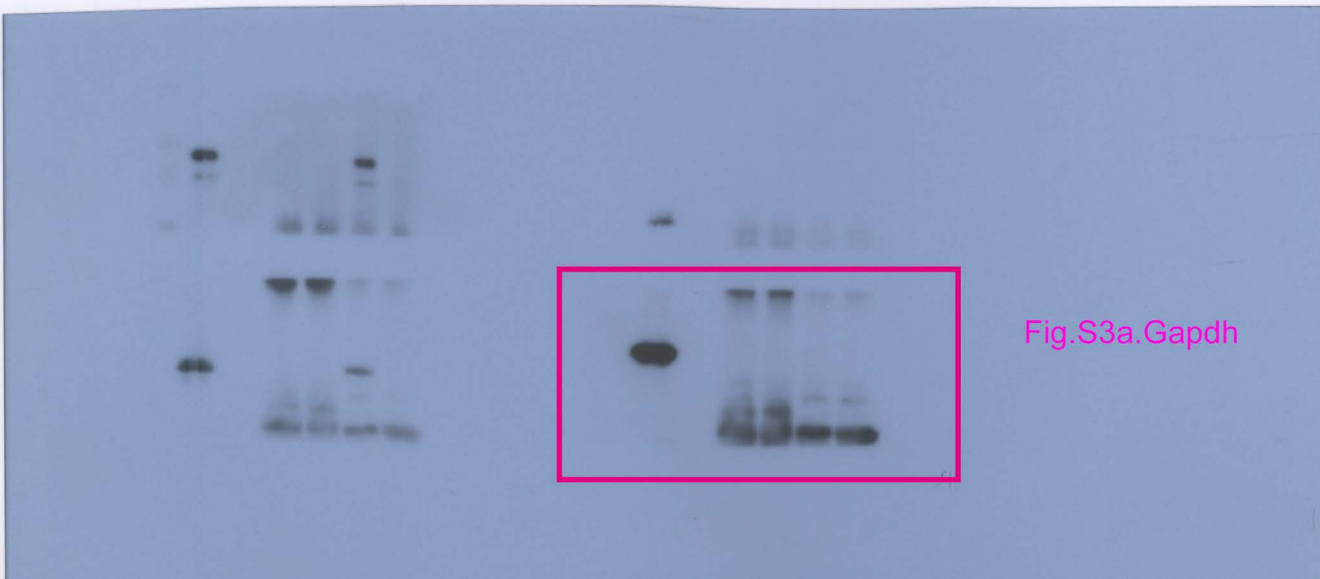

Supplement: Supplementary file 8 — Source Data [file 41467_2021_21529_MOESM8_ESM.zip › Uncropped blot and gel images/FigureS3/FigureS3a/hnRNP R_hnRNP A1_Hexim1_Gapdh.pdf]

Fig.s3a.Larp7

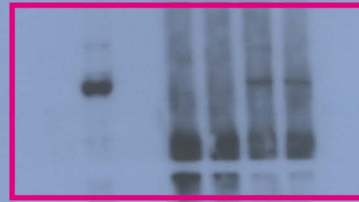

••FUJI•HFC•(SAFETY)••

Supplement: Supplementary file 8 — Source Data [file 41467_2021_21529_MOESM8_ESM.zip › Uncropped blot and gel images/FigureS3/FigureS3a/Larp7.pdf]

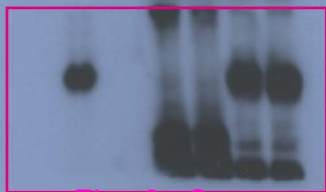

Fig.s3a Smn

U•HRC•(SAFETY)•••

••FUJI•HRC•(SAFETY)•••

Supplement: Supplementary file 8 — Source Data [file 41467_2021_21529_MOESM8_ESM.zip › Uncropped blot and gel images/FigureS3/FigureS3a/Smn.pdf]

Fig.s3b.Mepce

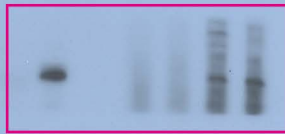

Fig.s3b.Smn

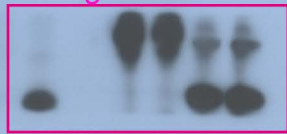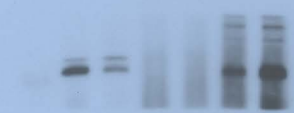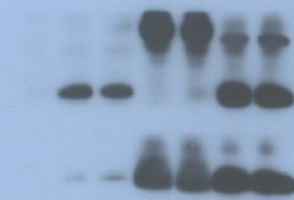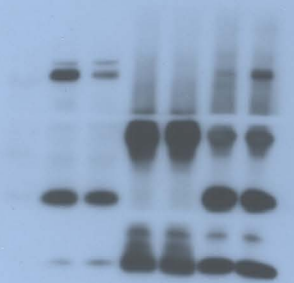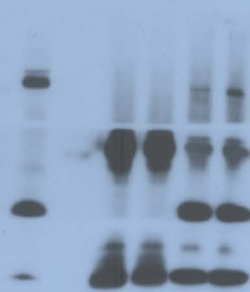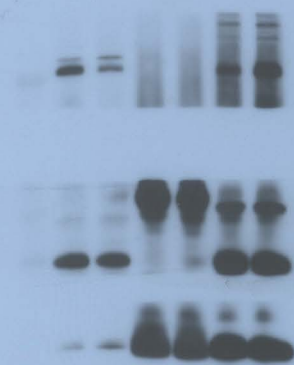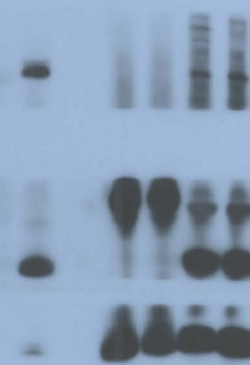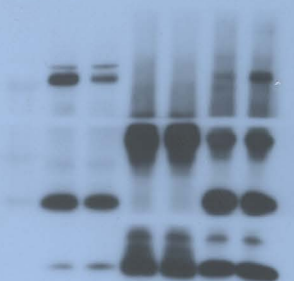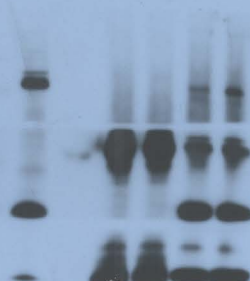

Supplement: Supplementary file 8 — Source Data [file 41467_2021_21529_MOESM8_ESM.zip › Uncropped blot and gel images/FigureS3/FigureS3b/Smn_Mepce.pdf]

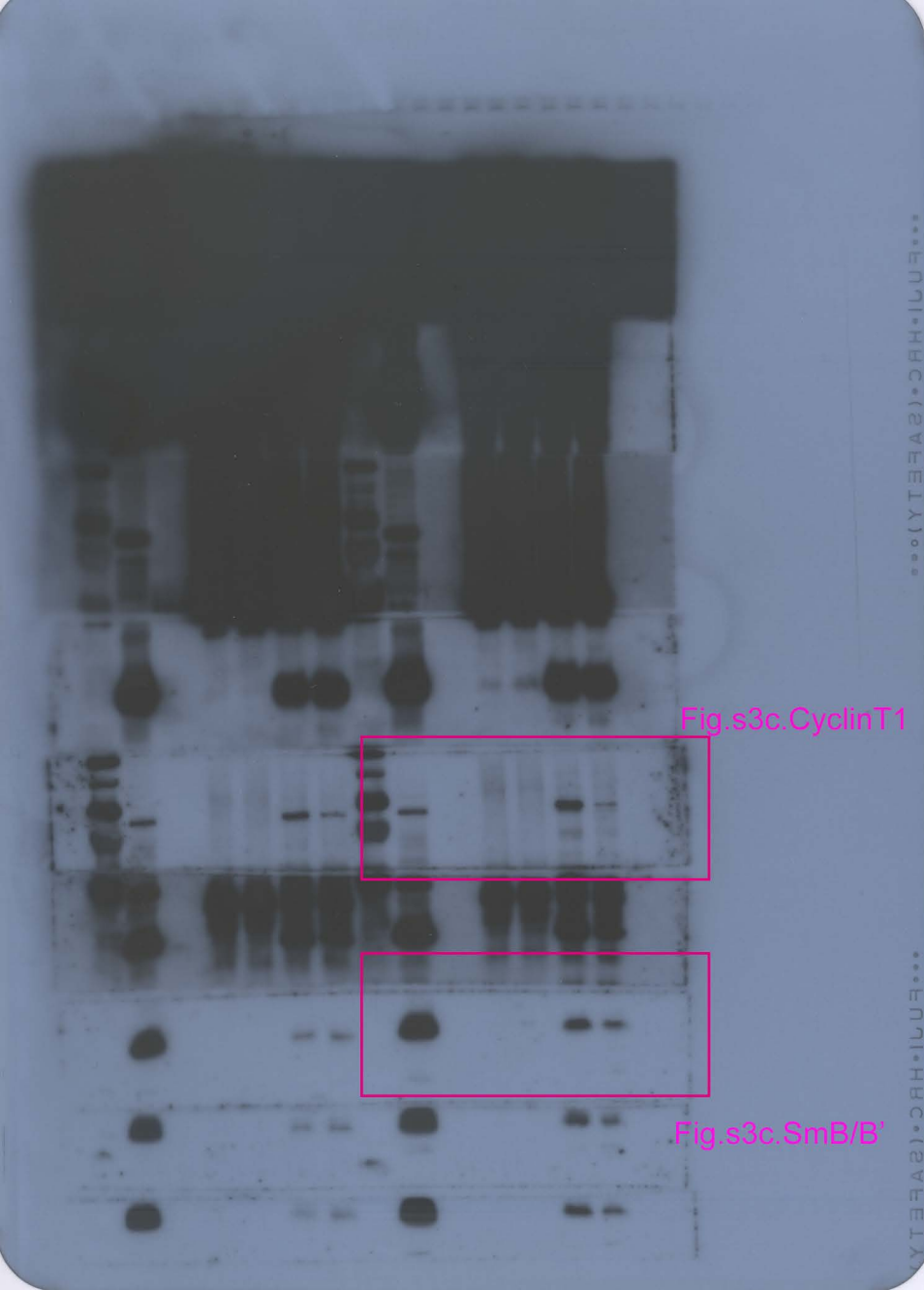

Fig.s3c.CyclinT1

Fig.s3c.SmB/B'

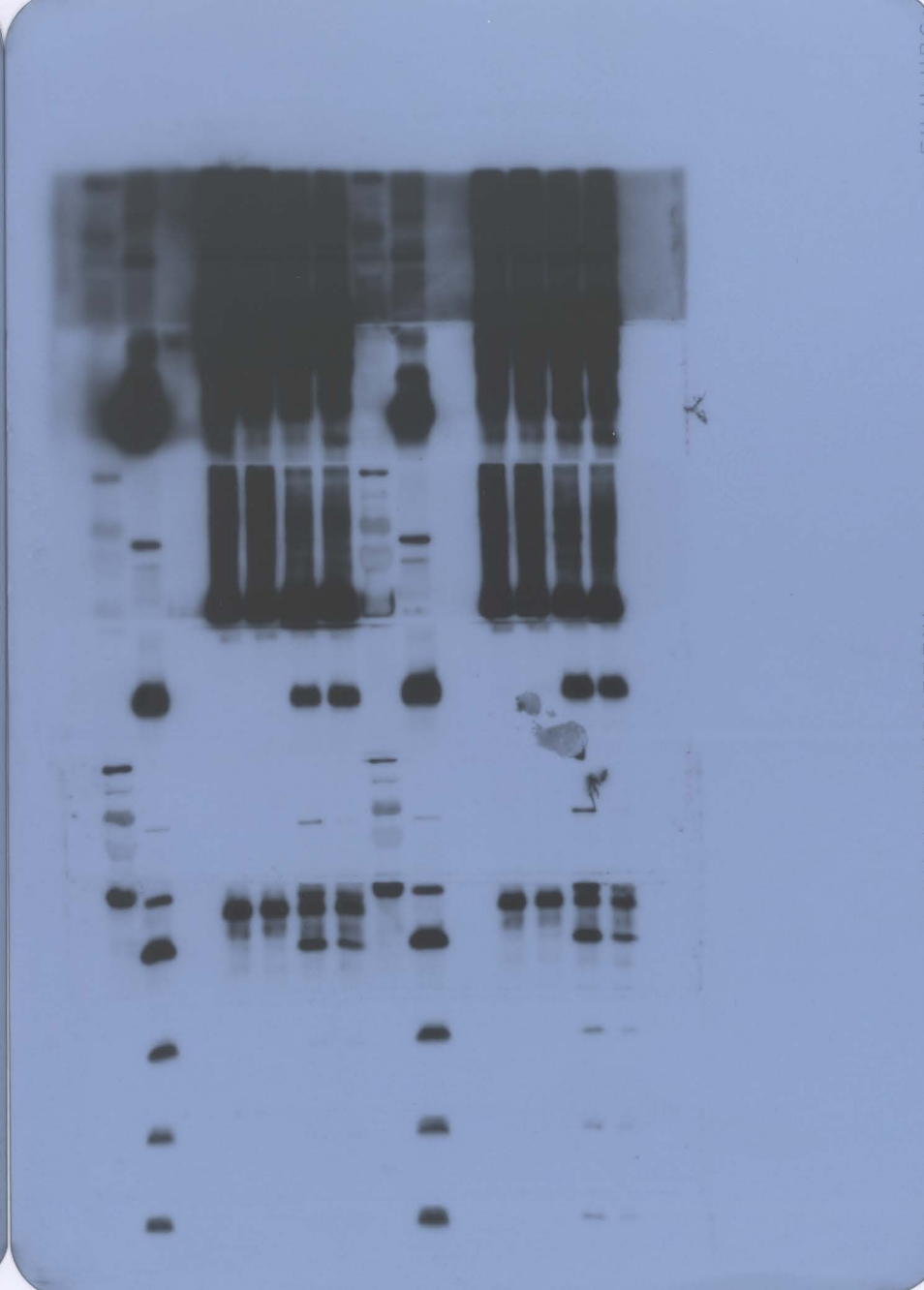

Supplement: Supplementary file 8 — Source Data [file 41467_2021_21529_MOESM8_ESM.zip › Uncropped blot and gel images/FigureS3/FigureS3c/CyclinT1_SmB.pdf]

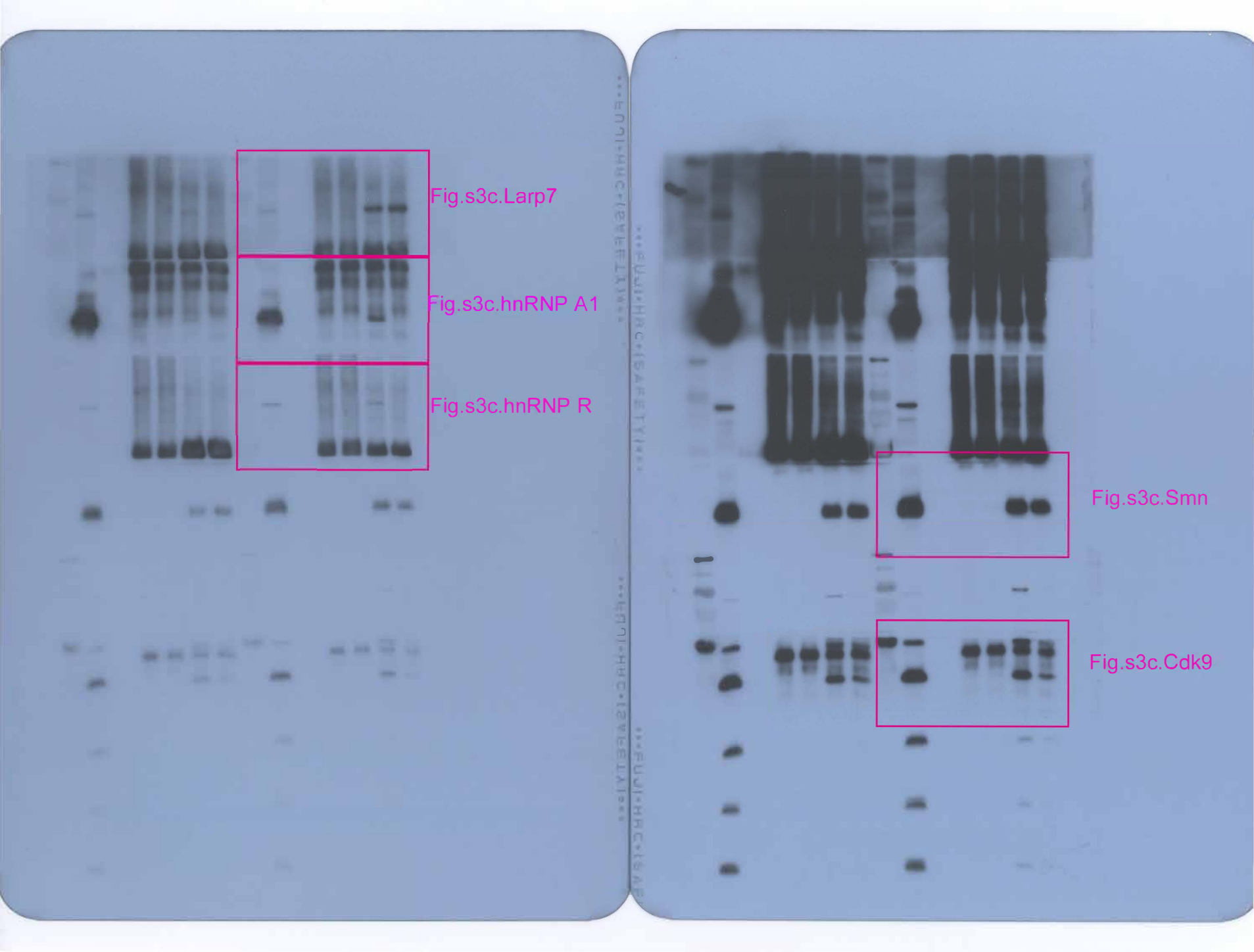

Fig.s3c.Larp7

Fig.s3c.hnRNP A1

Fig.s3c.hnRNP R

Fig.s3c.Smn

Fig.s3c.Cdk9

Supplement: Supplementary file 8 — Source Data [file 41467_2021_21529_MOESM8_ESM.zip › Uncropped blot and gel images/FigureS3/FigureS3c/Larp7_hnRNP A1_hnRNP R_Smn_Cdk9.pdf]

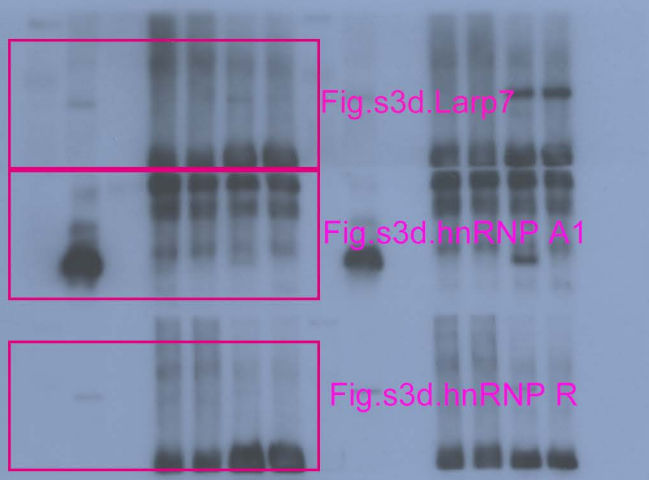

...FNU1•HRC•(SAFEITY)...

...FNU1•HRC•(SAFEITY)...

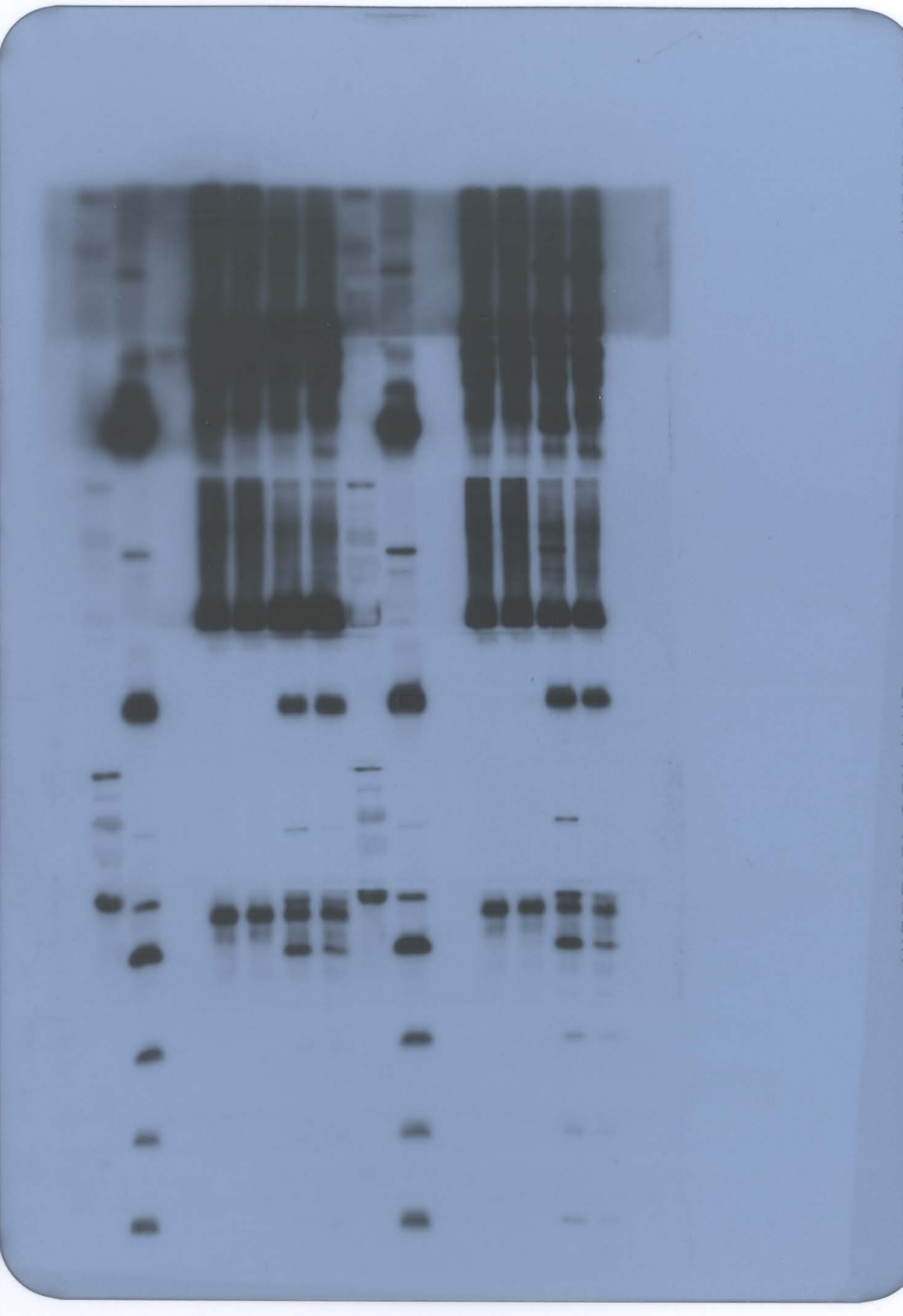

Supplement: Supplementary file 8 — Source Data [file 41467_2021_21529_MOESM8_ESM.zip › Uncropped blot and gel images/FigureS3/FigureS3d/Larp7_hnRNP A1_hnRNP R.pdf]

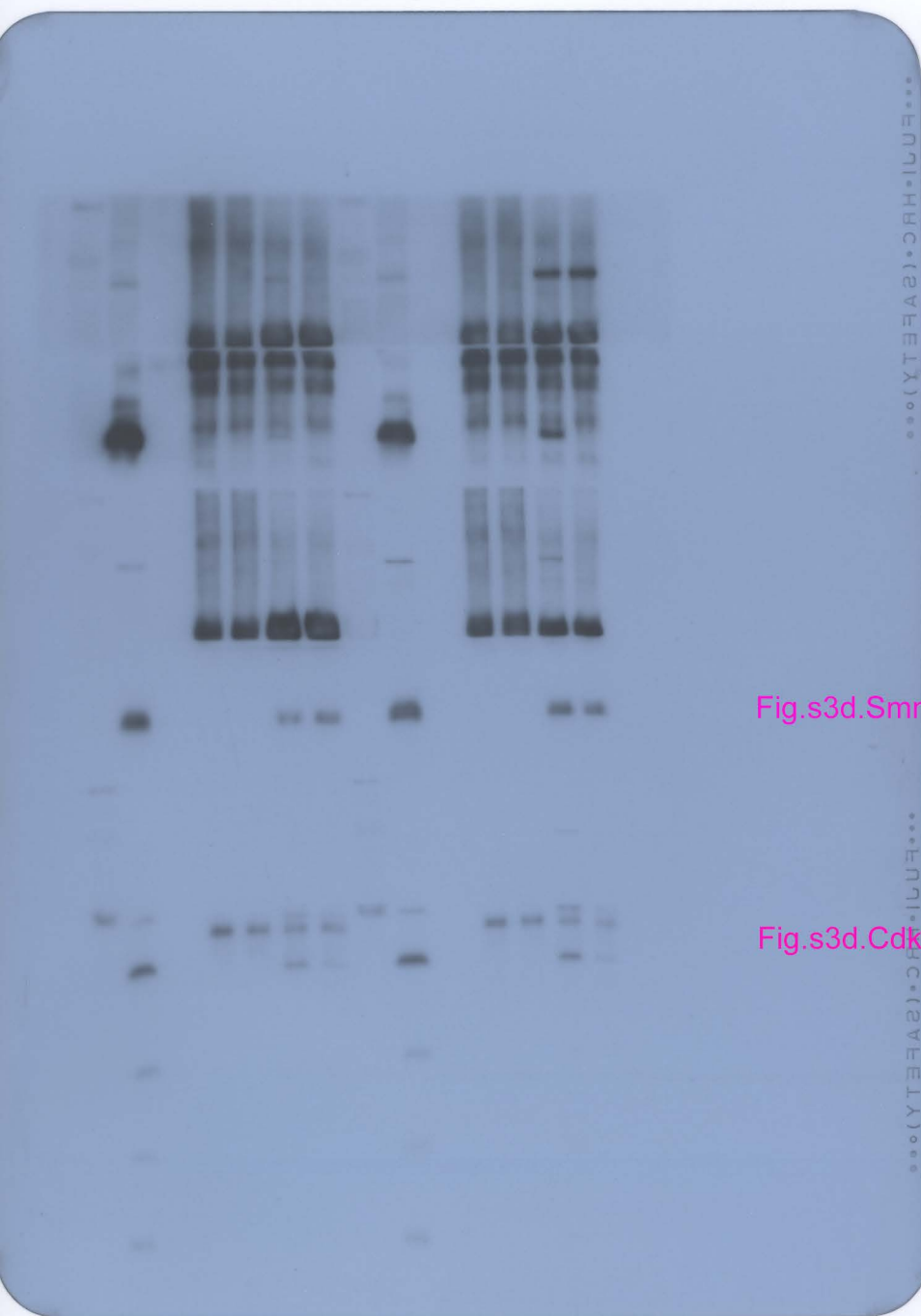

Fig.s3d.Smn

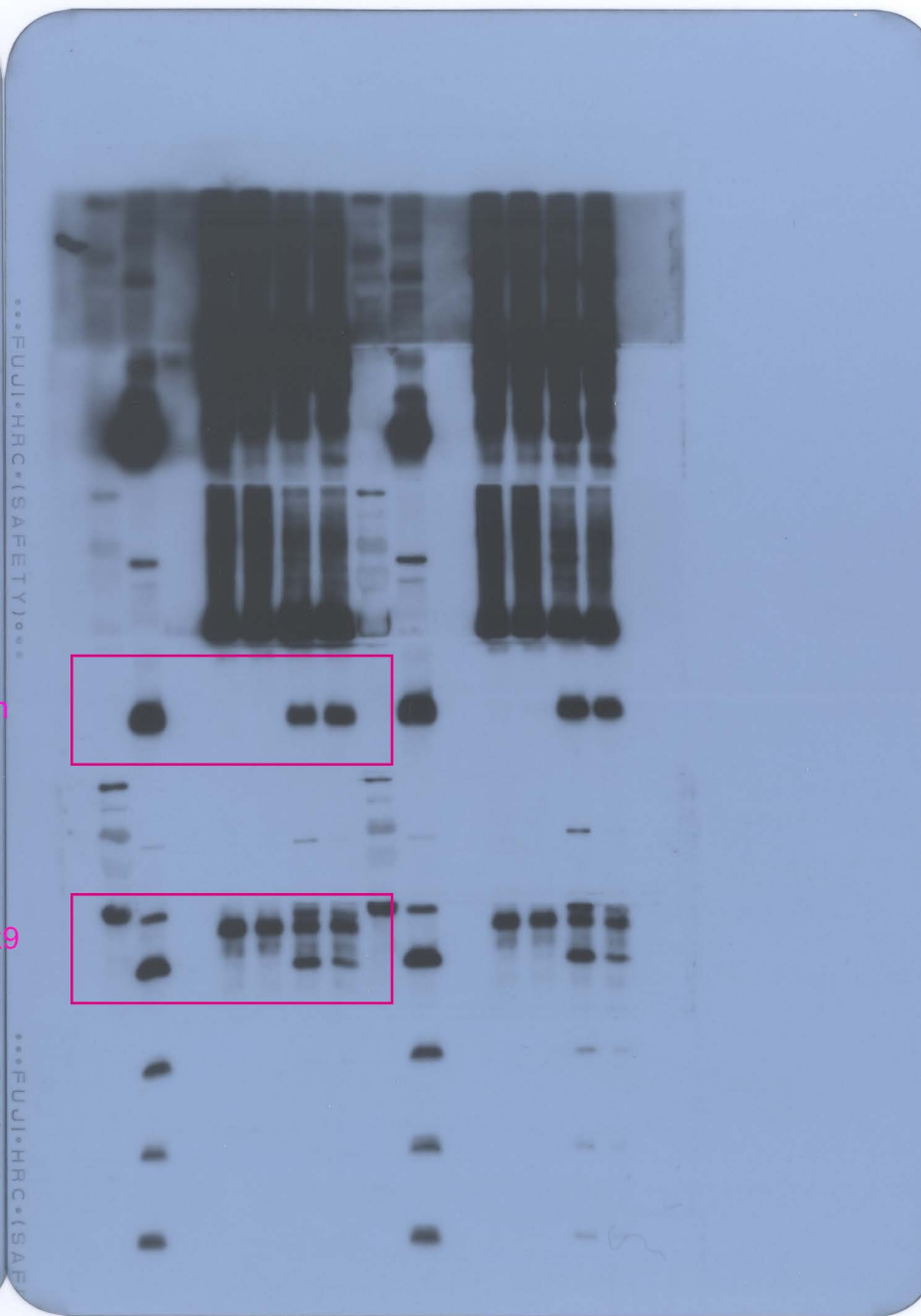

Fig.s3d.Cdk9

Supplement: Supplementary file 8 — Source Data [file 41467_2021_21529_MOESM8_ESM.zip › Uncropped blot and gel images/FigureS3/FigureS3d/Smn_Cdk9.pdf]

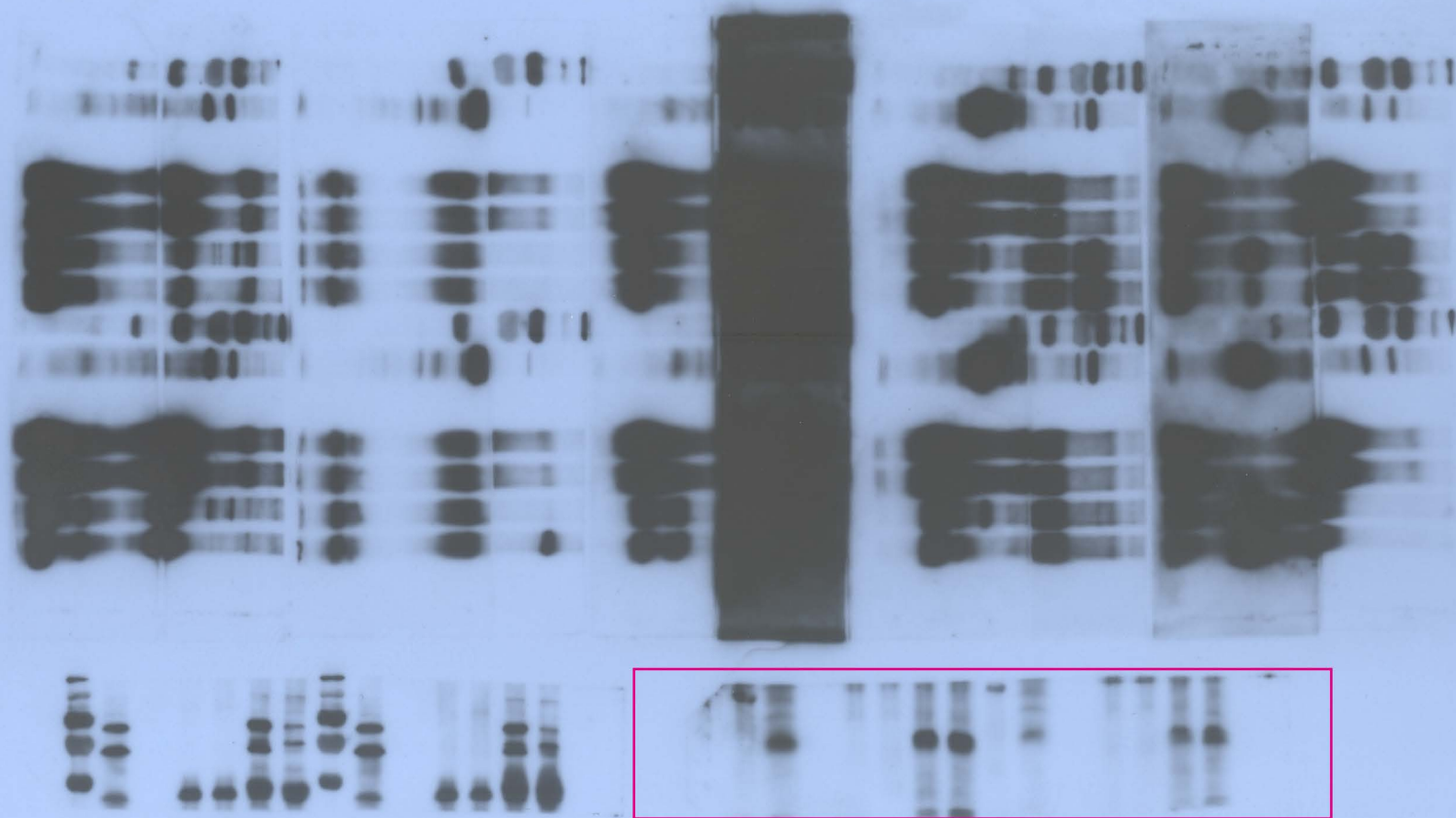

Fig.s3e.Gemin2

Supplement: Supplementary file 8 — Source Data [file 41467_2021_21529_MOESM8_ESM.zip › Uncropped blot and gel images/FigureS3/FigureS3e/Gemin2.pdf]

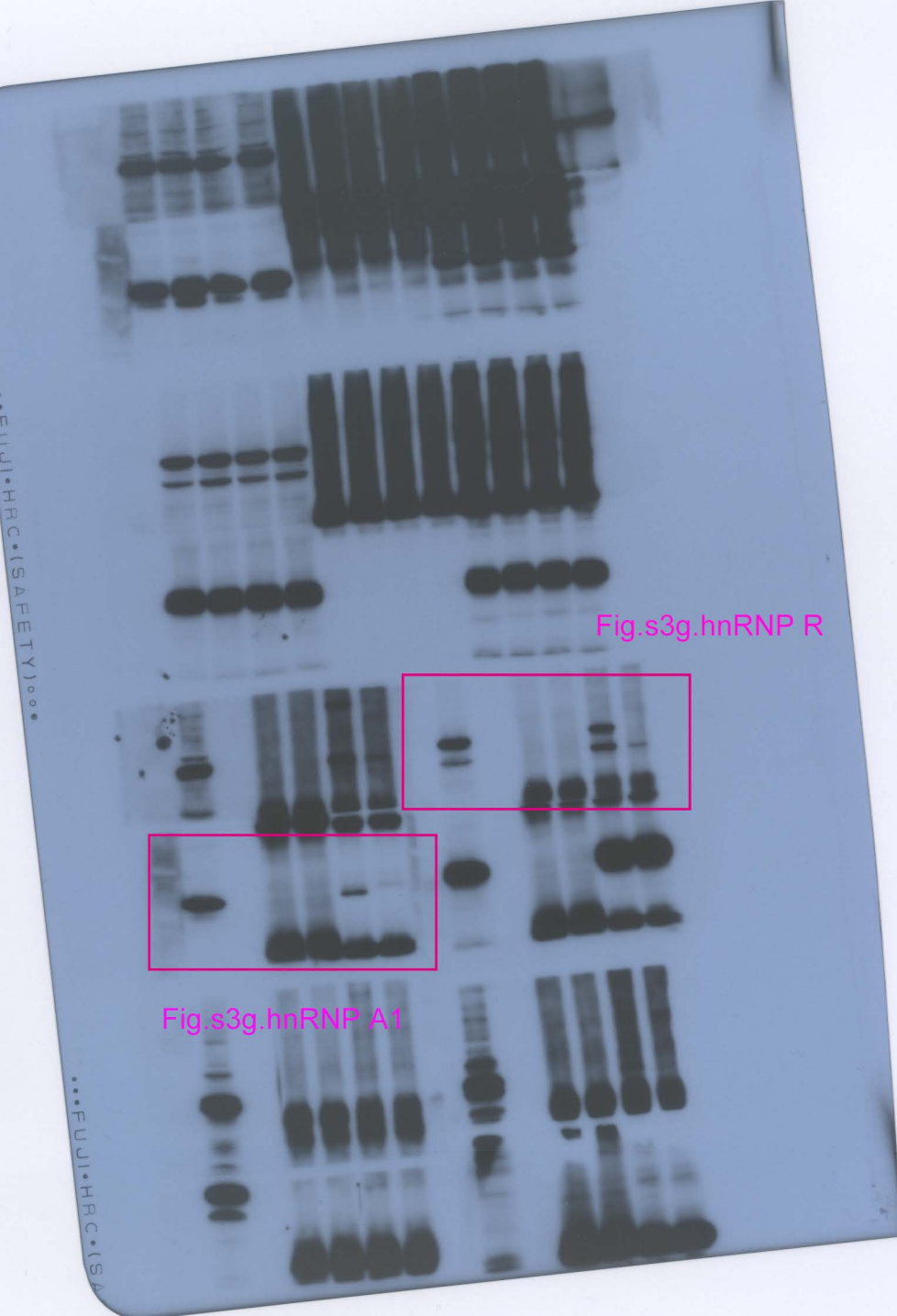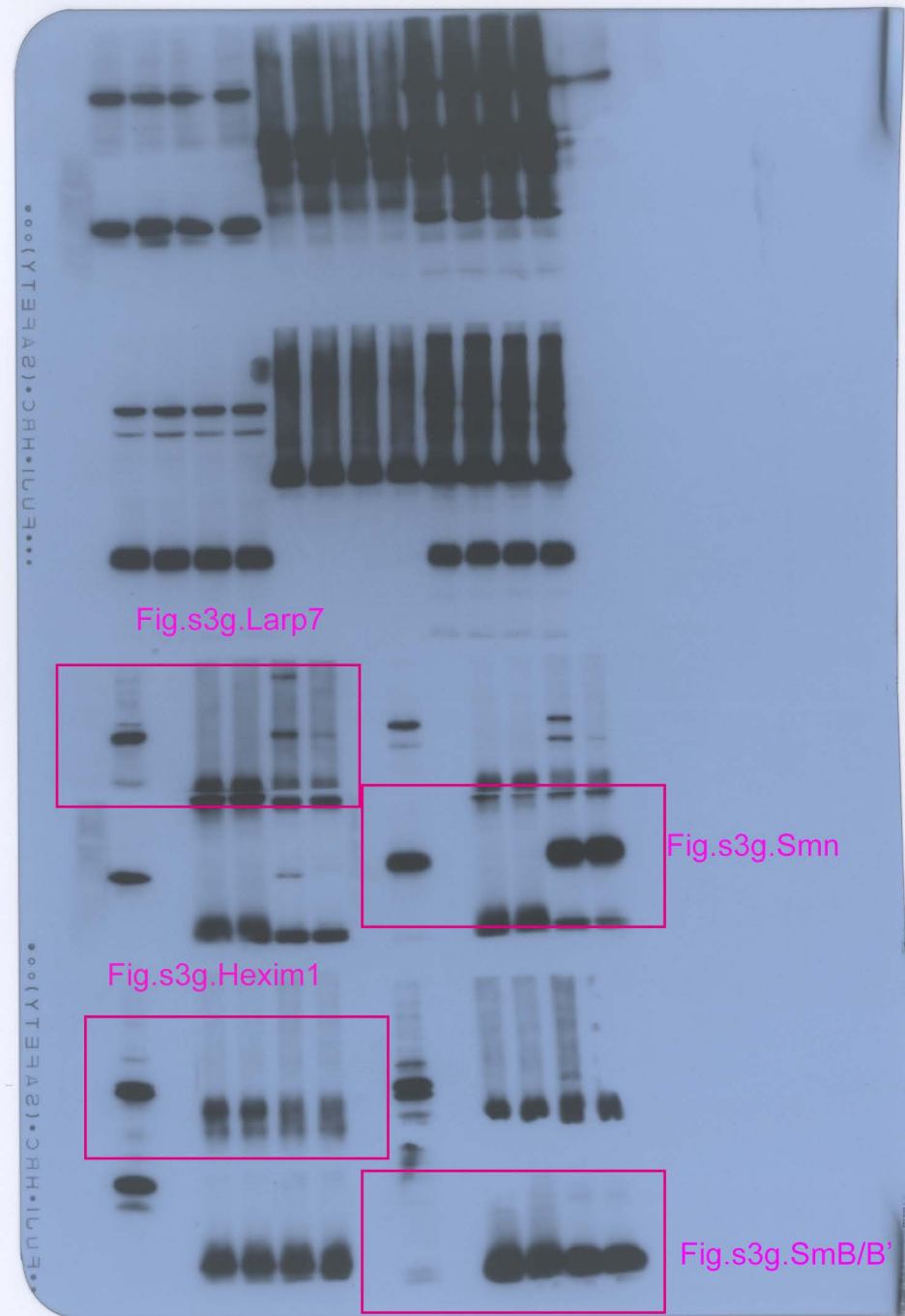

Supplement: Supplementary file 8 — Source Data [file 41467_2021_21529_MOESM8_ESM.zip › Uncropped blot and gel images/FigureS3/FigureS3g/SmB_Smn_Larp7_hnRNP R_hnRNP A1_Hexim1.pdf]

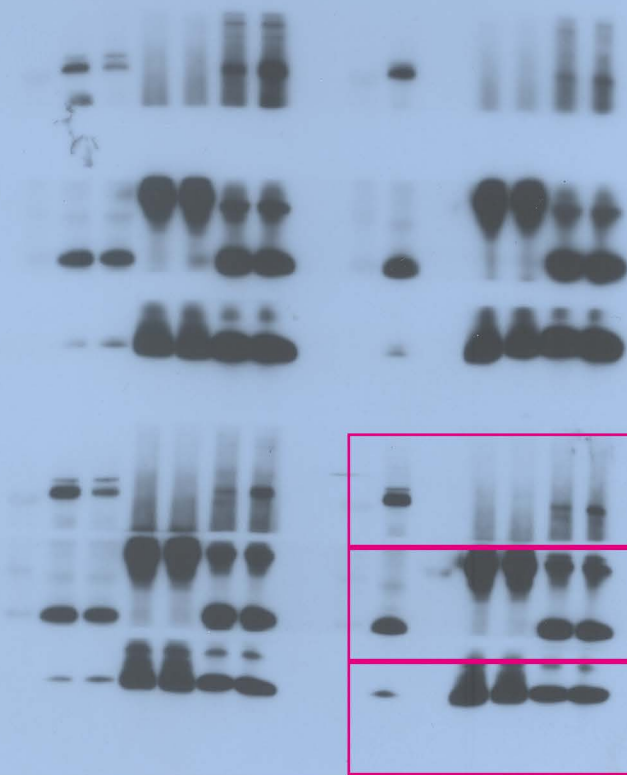

Fig.s3h.Mepce

Fig.s3h.Smn

Fig.s3h.SmB/B'

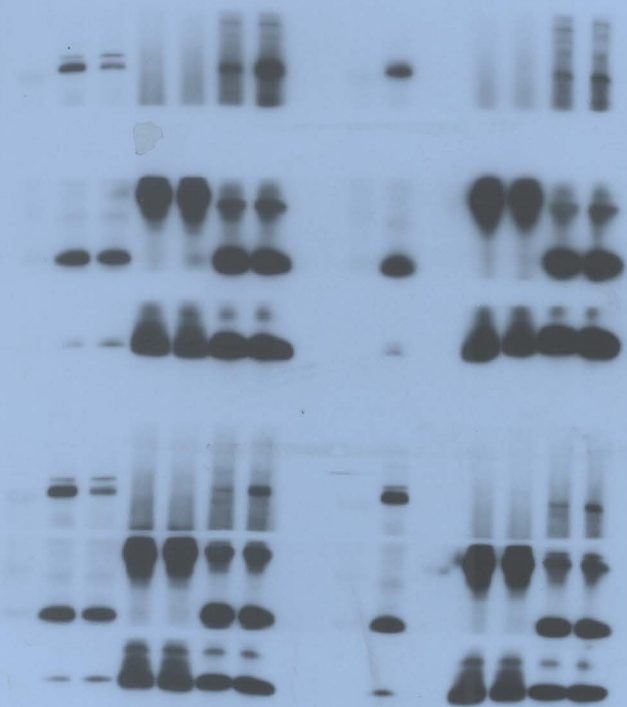

Supplement: Supplementary file 8 — Source Data [file 41467_2021_21529_MOESM8_ESM.zip › Uncropped blot and gel images/FigureS3/FigureS3h/Mepce_Smn_SmB.pdf]

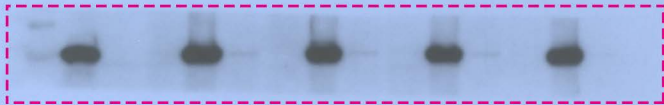

Fig.s4a Calnexin

Supplement: Supplementary file 8 — Source Data [file 41467_2021_21529_MOESM8_ESM.zip › Uncropped blot and gel images/FigureS4/FigureS4a/Calnexin.pdf]

Fig.s4a.Gapdh

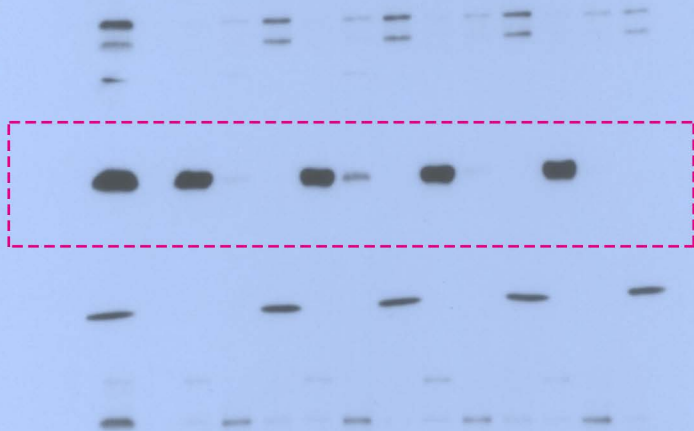

Supplement: Supplementary file 8 — Source Data [file 41467_2021_21529_MOESM8_ESM.zip › Uncropped blot and gel images/FigureS4/FigureS4a/Gapdh.pdf]

Fig.s4a.HistoneH3

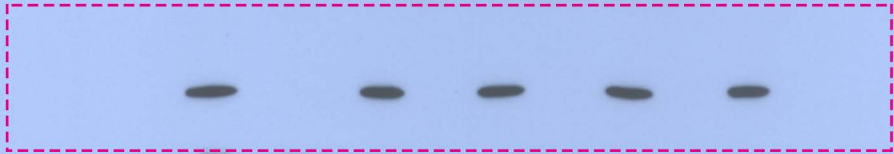

Supplement: Supplementary file 8 — Source Data [file 41467_2021_21529_MOESM8_ESM.zip › Uncropped blot and gel images/FigureS4/FigureS4a/Histone H3.pdf]

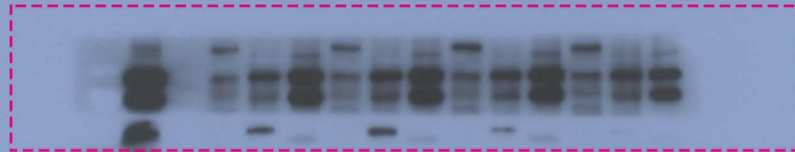

Fig.s4a. hnRNP R

Supplement: Supplementary file 8 — Source Data [file 41467_2021_21529_MOESM8_ESM.zip › Uncropped blot and gel images/FigureS4/FigureS4a/hnRNP R.pdf]

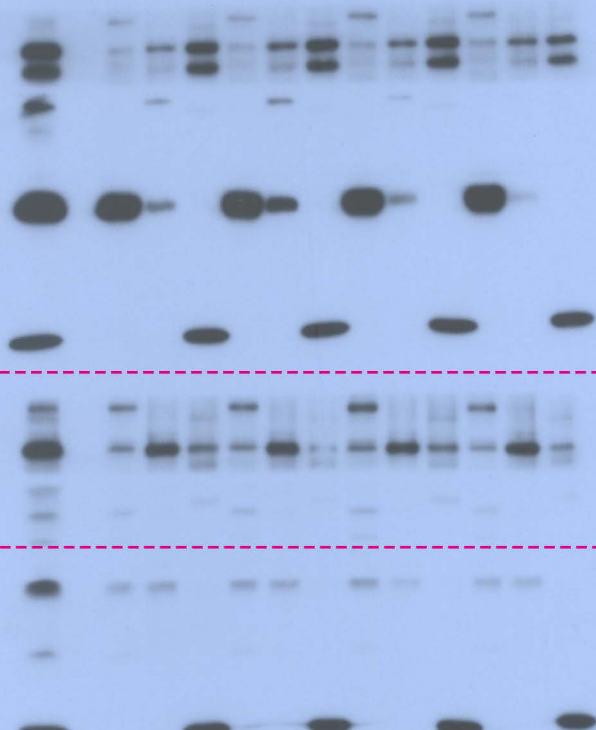

Fig.s4a.Larp7

Supplement: Supplementary file 8 — Source Data [file 41467_2021_21529_MOESM8_ESM.zip › Uncropped blot and gel images/FigureS4/FigureS4a/Larp7.pdf]

Fig.s4a. Smn

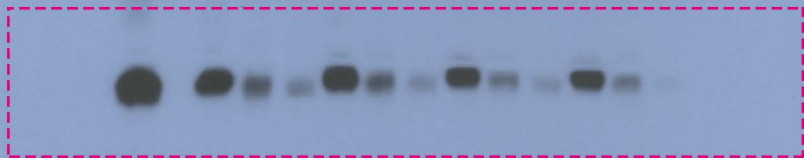

Supplement: Supplementary file 8 — Source Data [file 41467_2021_21529_MOESM8_ESM.zip › Uncropped blot and gel images/FigureS4/FigureS4a/Smn.pdf]

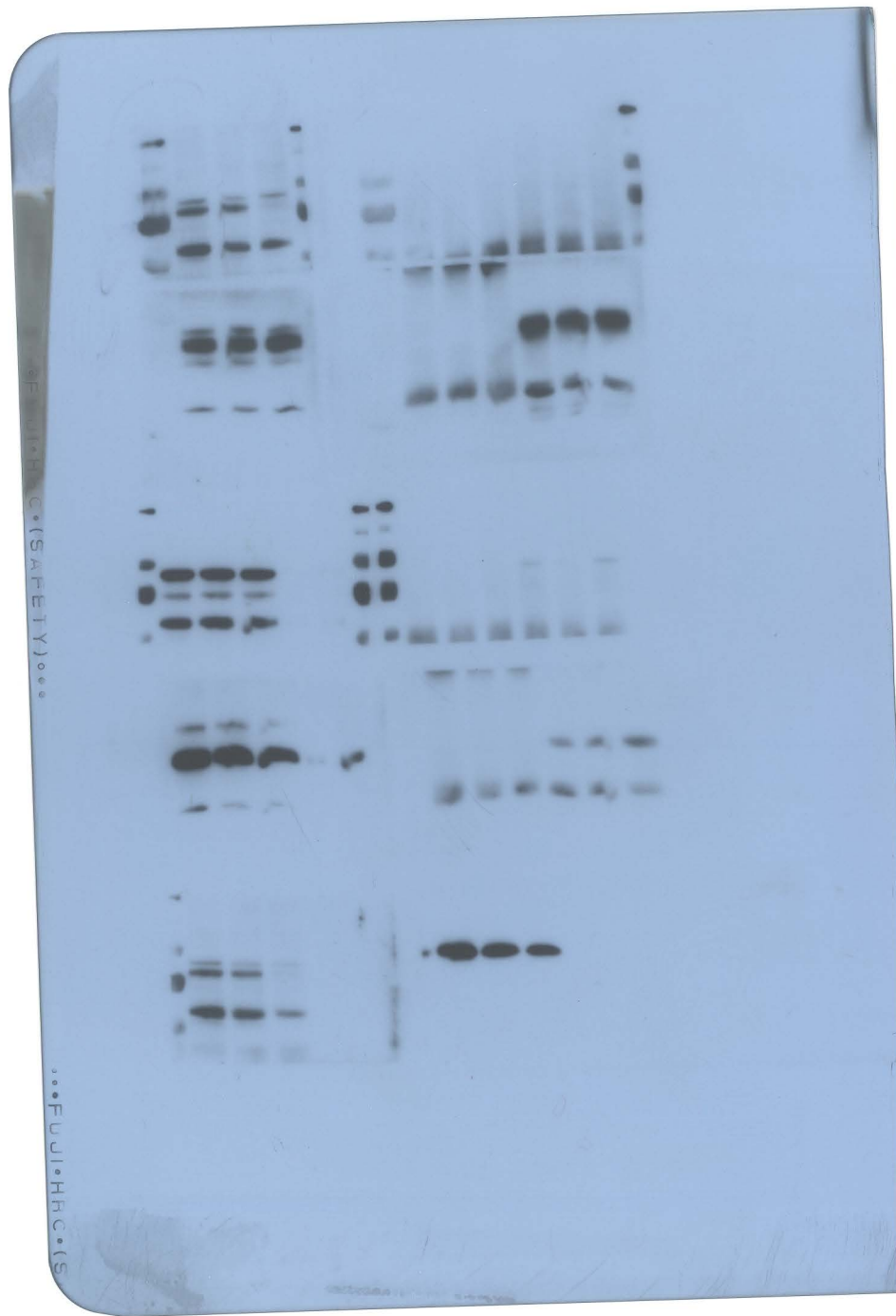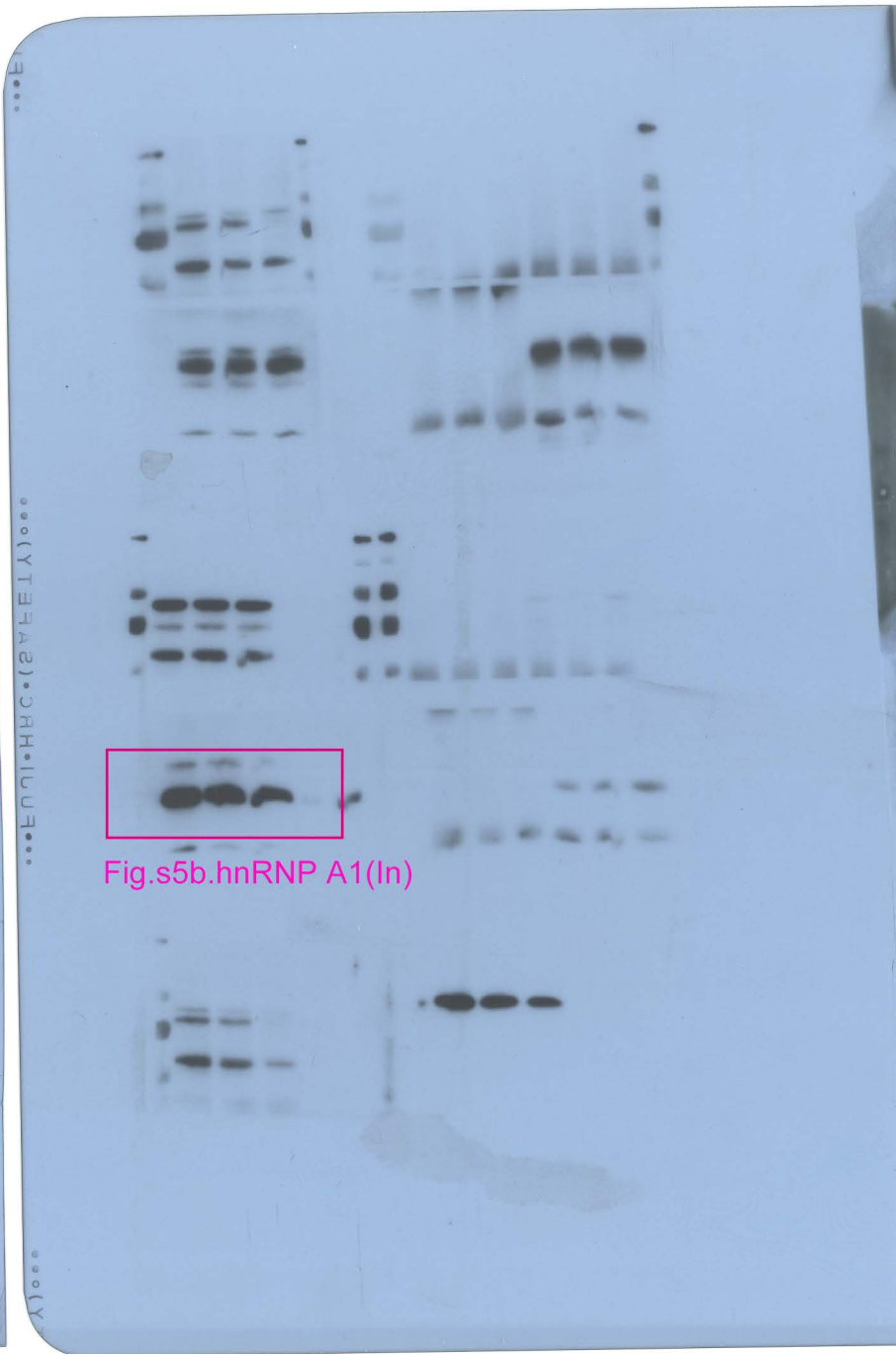

Fig.s5b.hnRNP A1(In)

Supplement: Supplementary file 8 — Source Data [file 41467_2021_21529_MOESM8_ESM.zip › Uncropped blot and gel images/FigureS5/FigureS5b/hnRNP A1.pdf]

Fig.s5b.Larp7(In)

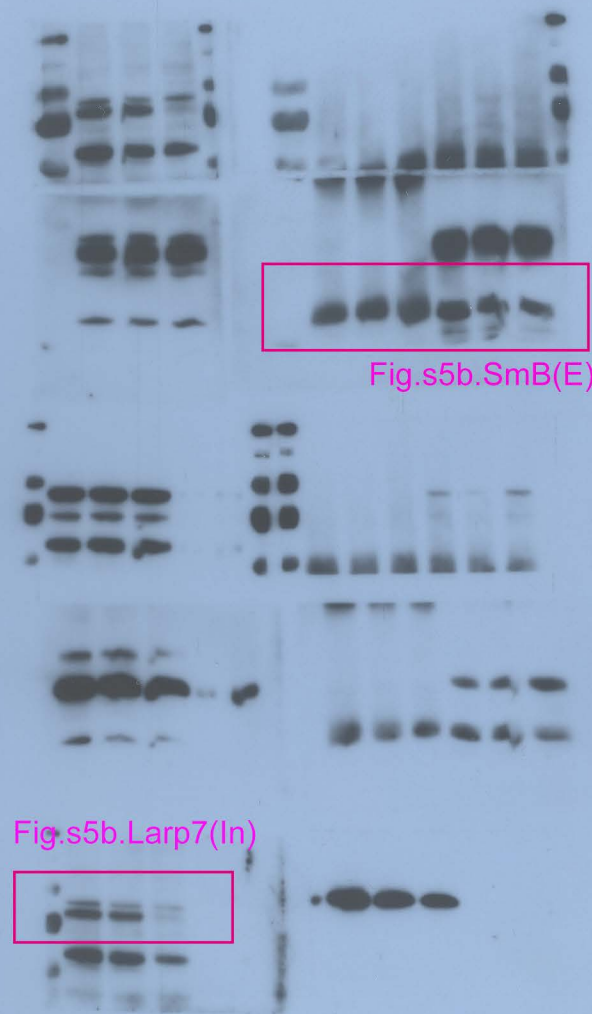

Fig.s5b.SmB(E)

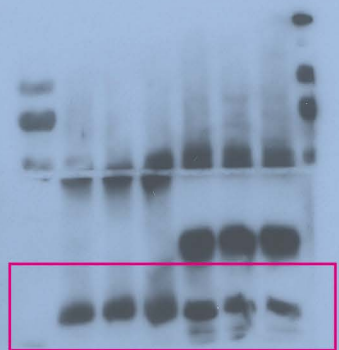

Fig.s5b.hnRNP R(E)

Fig.s5b.Hexim1(E)

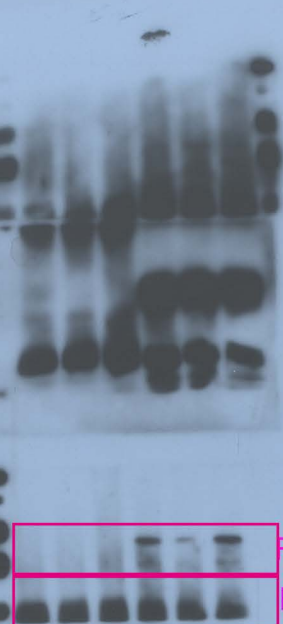

Supplement: Supplementary file 8 — Source Data [file 41467_2021_21529_MOESM8_ESM.zip › Uncropped blot and gel images/FigureS5/FigureS5b/Larp7_SmB_Hexim1_hnRNP R.pdf]

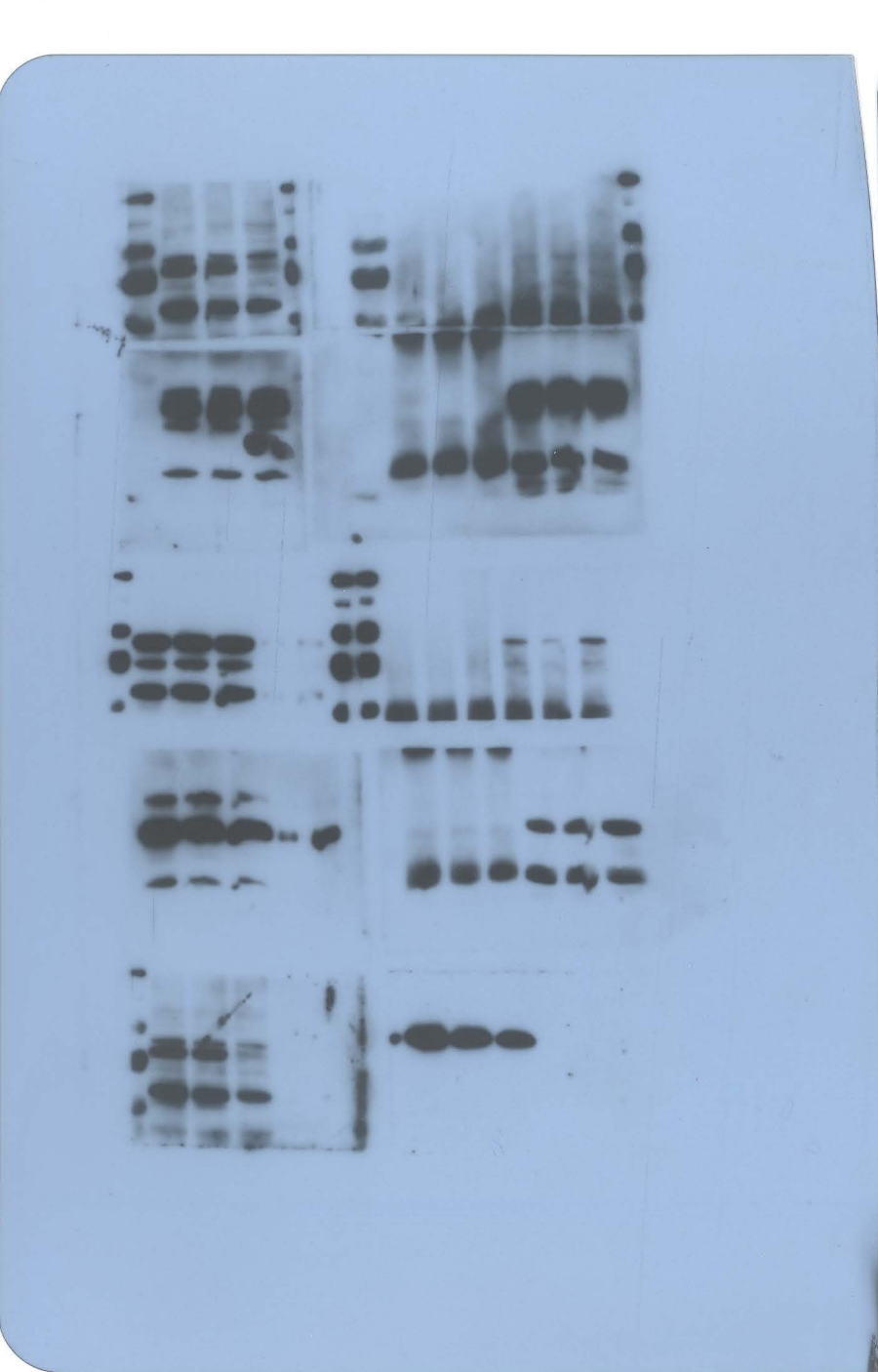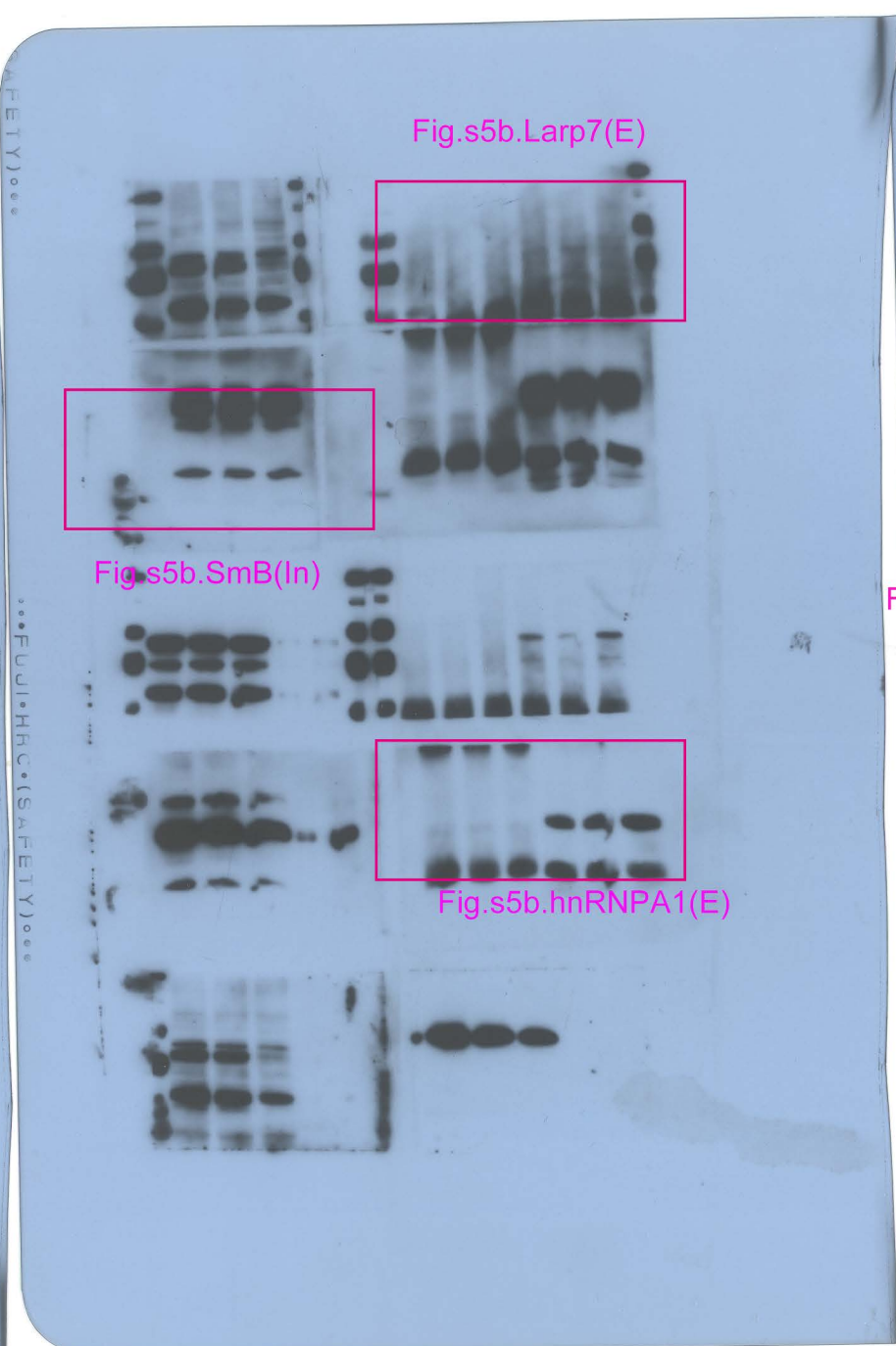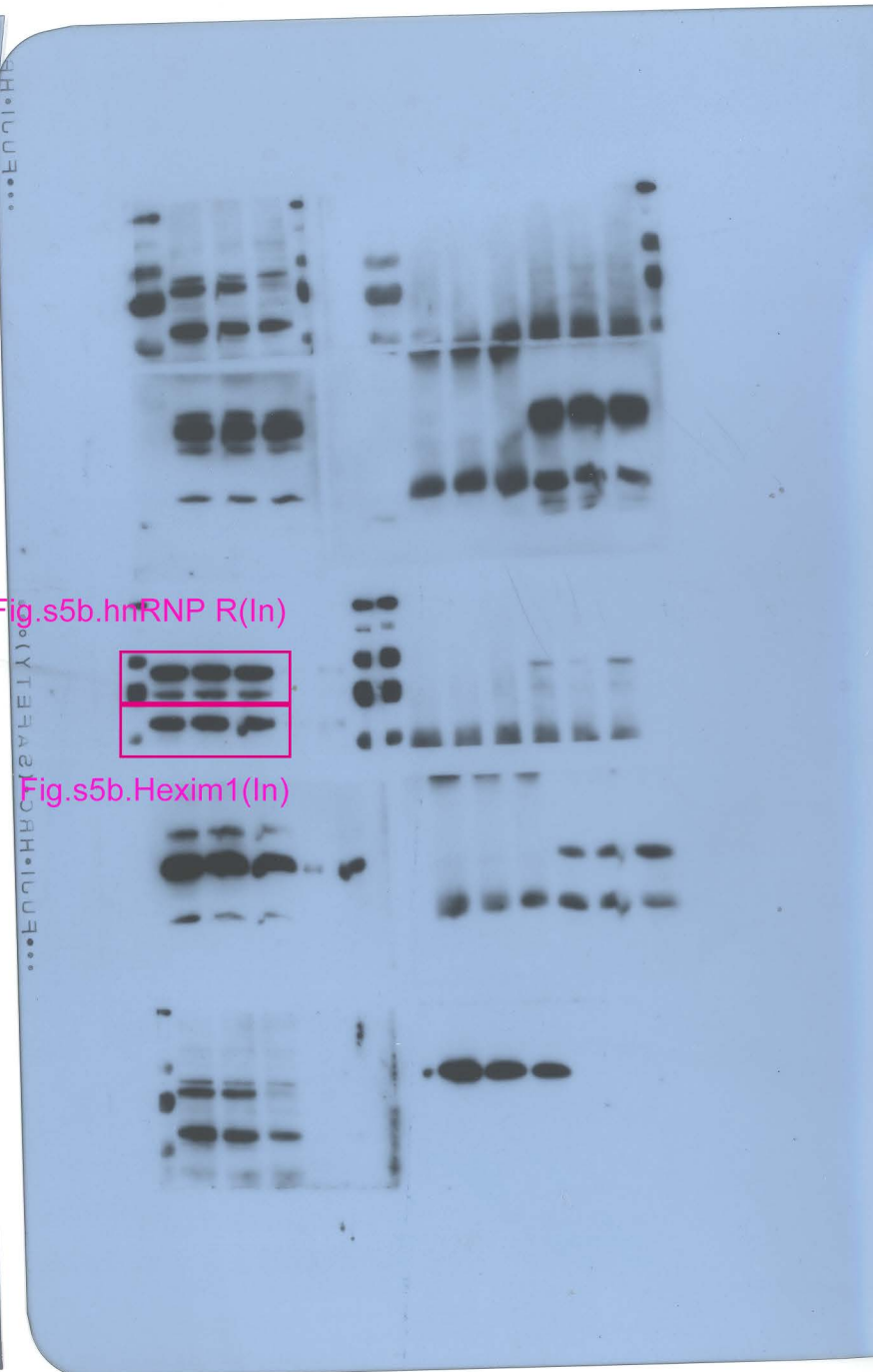

Supplement: Supplementary file 8 — Source Data [file 41467_2021_21529_MOESM8_ESM.zip › Uncropped blot and gel images/FigureS5/FigureS5b/Larp7_SmB_Hexim1_hnRNP R_hnRNP A1.pdf]

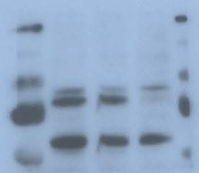

Fig.s5b.Cdk9.

Smn(In)

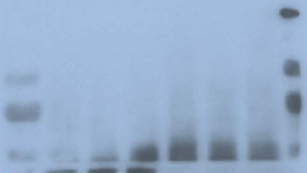

Fig.s5b.Smn(E)

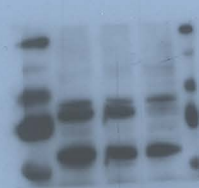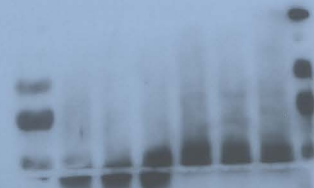

Supplement: Supplementary file 8 — Source Data [file 41467_2021_21529_MOESM8_ESM.zip › Uncropped blot and gel images/FigureS5/FigureS5b/Smn_Cdk9.pdf]

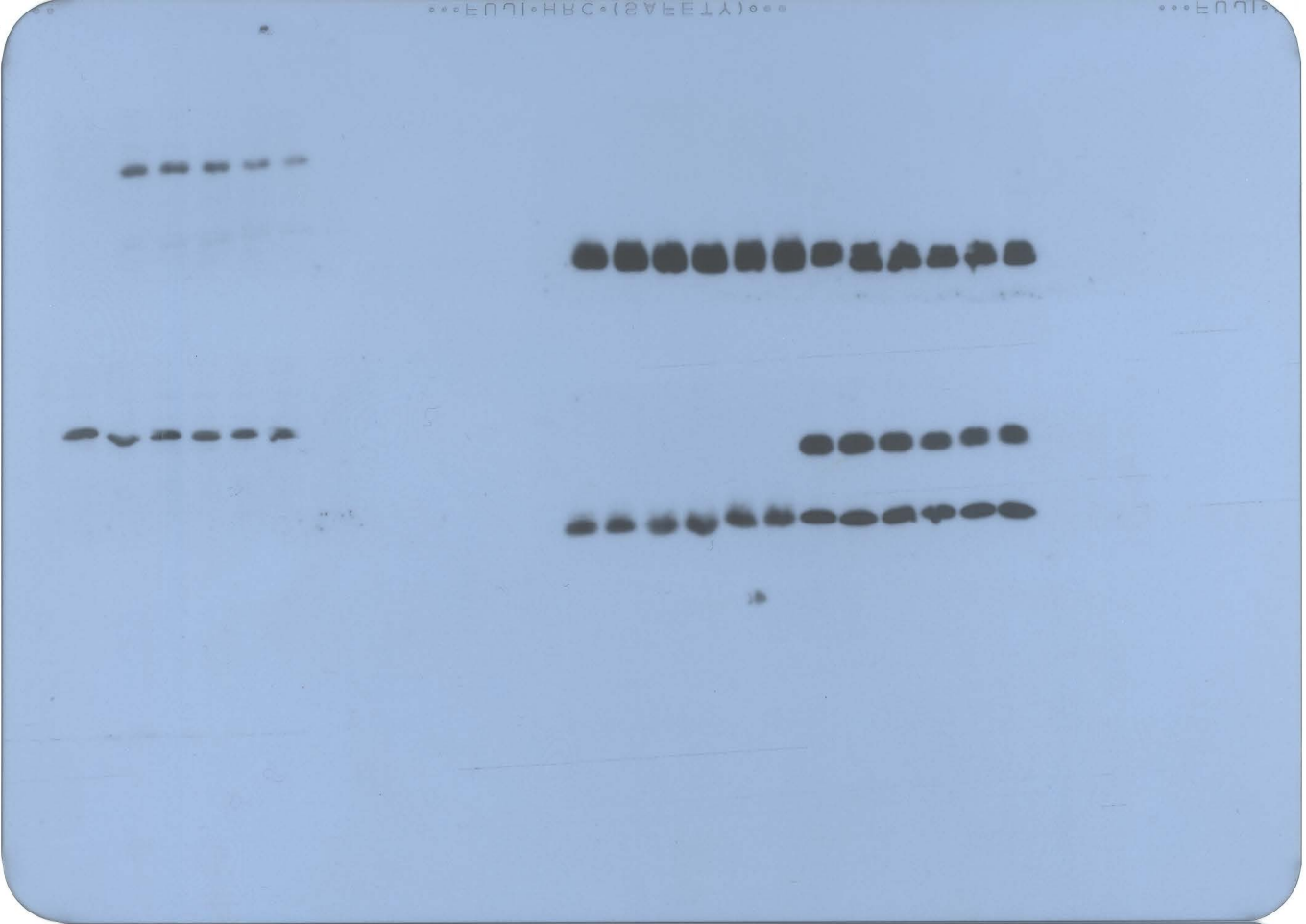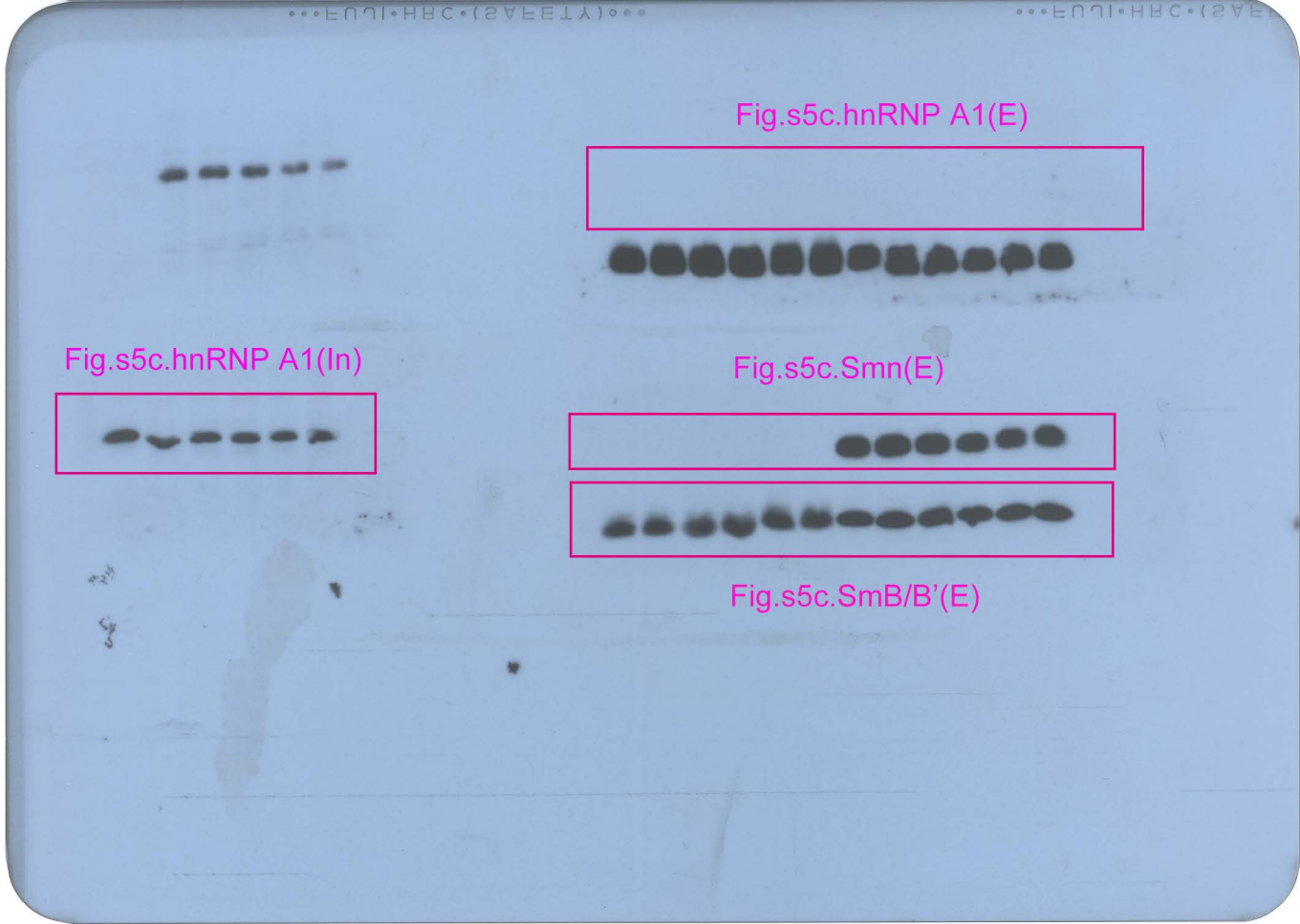

Supplement: Supplementary file 8 — Source Data [file 41467_2021_21529_MOESM8_ESM.zip › Uncropped blot and gel images/FigureS5/FigureS5c/hnRNP A1_Smn_SmB.pdf]

...EULLHHC•(2)AFLA

•••(YIAFA)•••

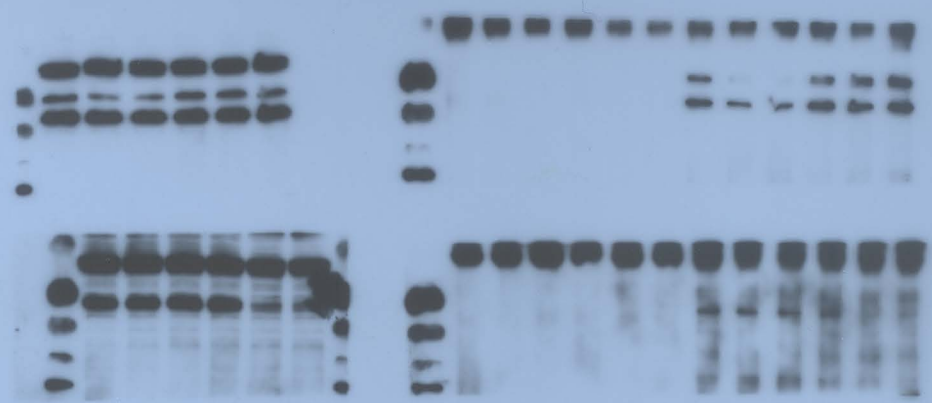

...EULLHHC•(2)AFLA

•••(YIAFA)•••

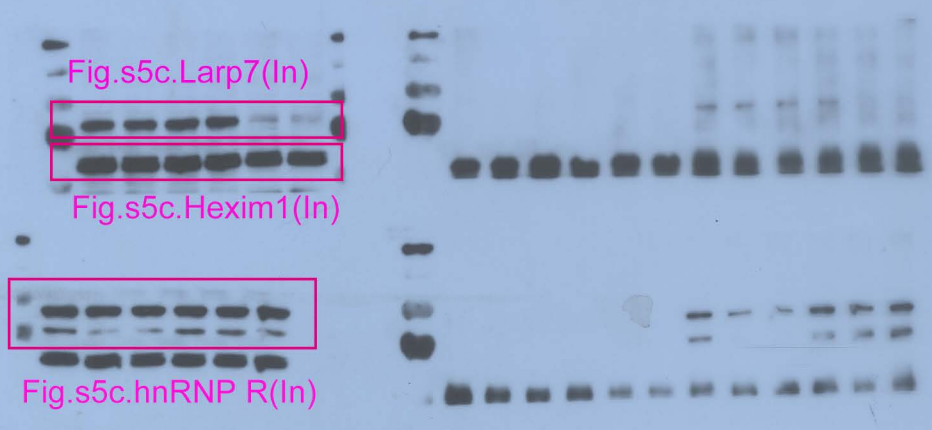

Fig.s5c.Larp7(In)

Fig.s5c.Hexim1(In)

Fig.s5c.hnRNP R(In)

Supplement: Supplementary file 8 — Source Data [file 41467_2021_21529_MOESM8_ESM.zip › Uncropped blot and gel images/FigureS5/FigureS5c/Larp7_hnRNP R_Hexim1.pdf]

Fig.s5c.SmB/B'(In)

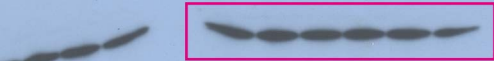

Supplement: Supplementary file 8 — Source Data [file 41467_2021_21529_MOESM8_ESM.zip › Uncropped blot and gel images/FigureS5/FigureS5c/SmB.pdf]

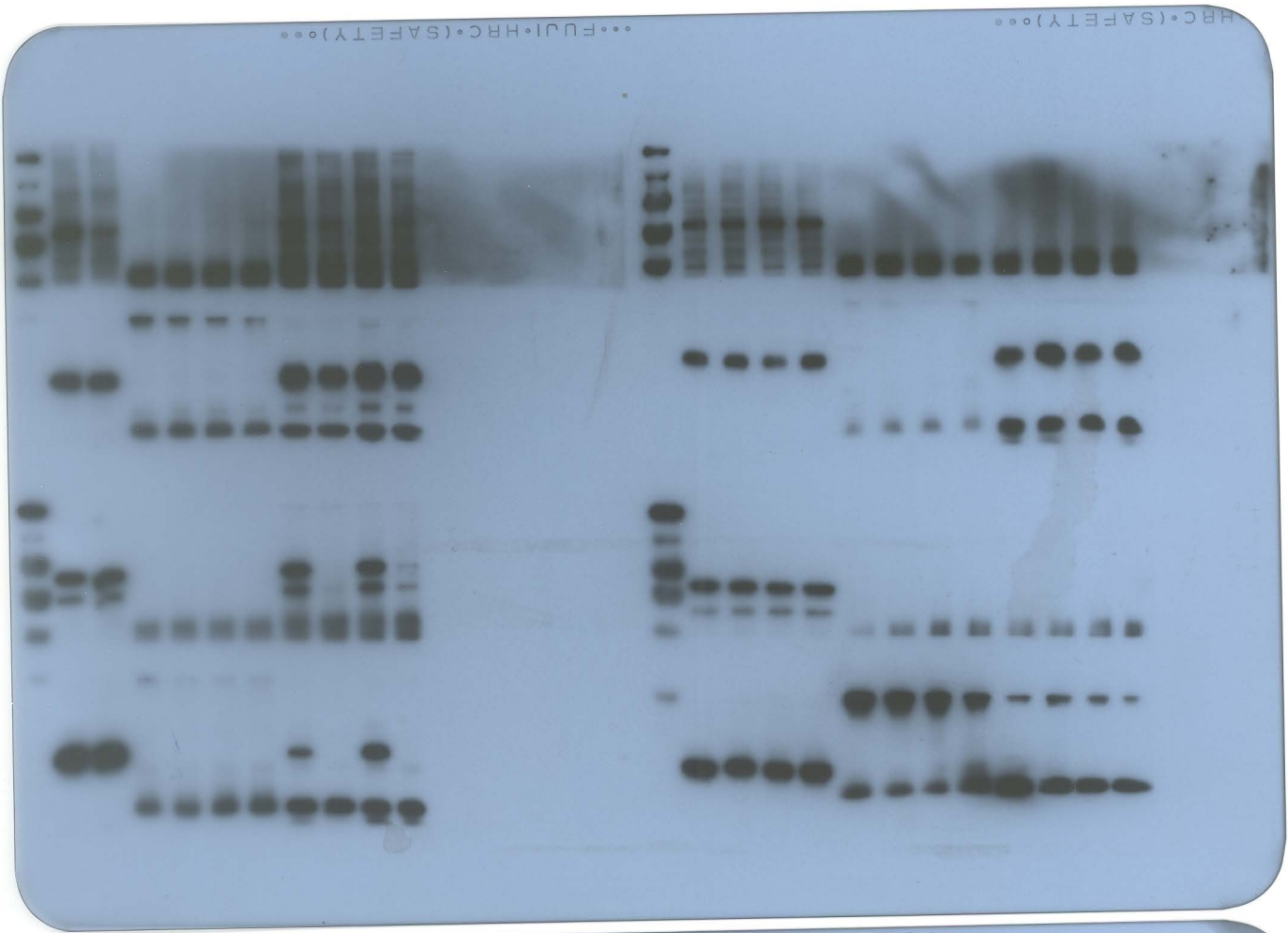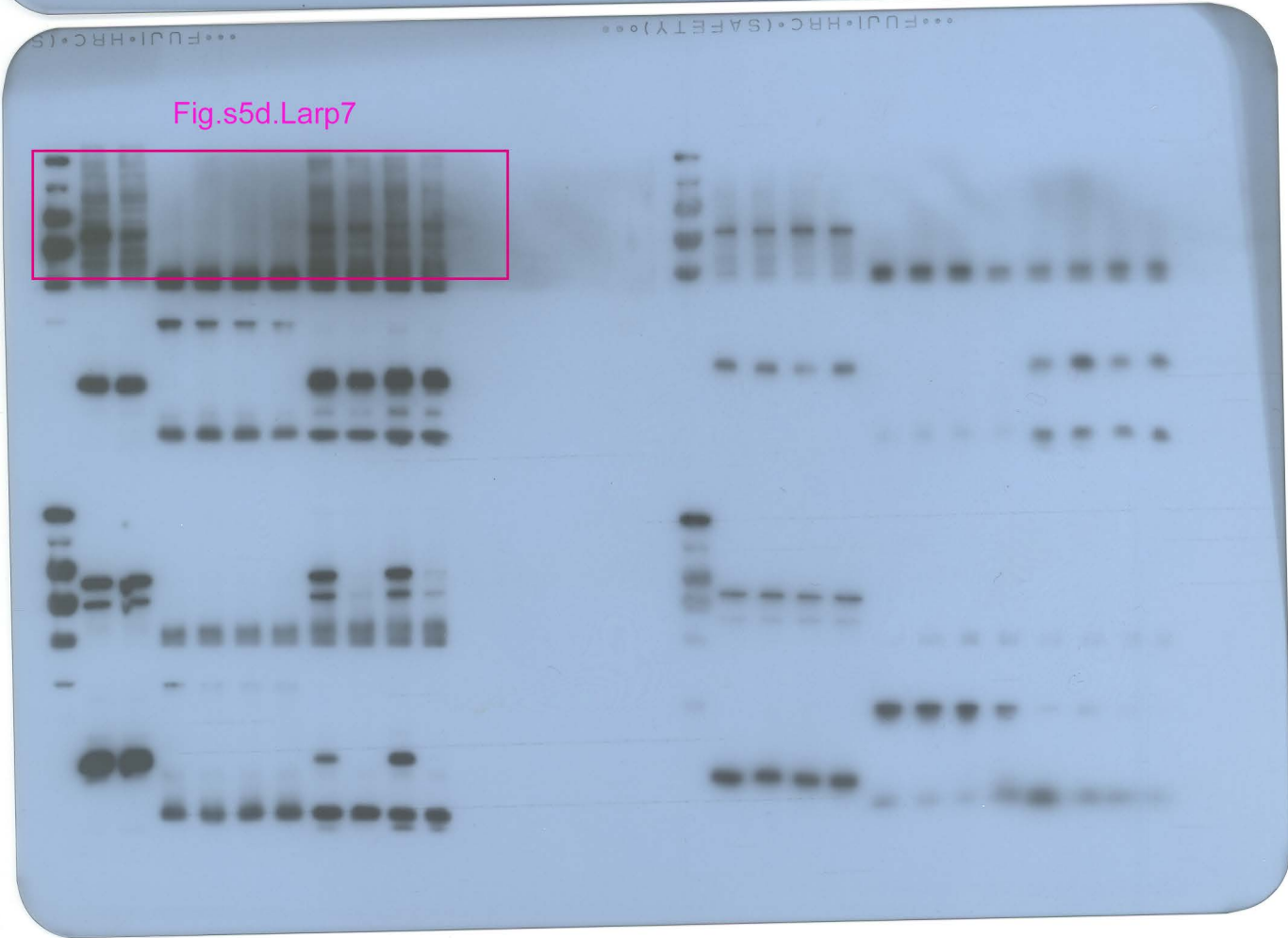

Fig.s5d.Larp7

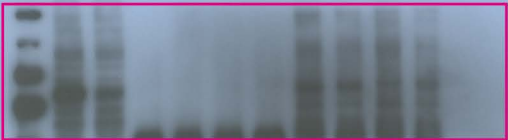

Supplement: Supplementary file 8 — Source Data [file 41467_2021_21529_MOESM8_ESM.zip › Uncropped blot and gel images/FigureS5/FigureS5d/Larp7.pdf]

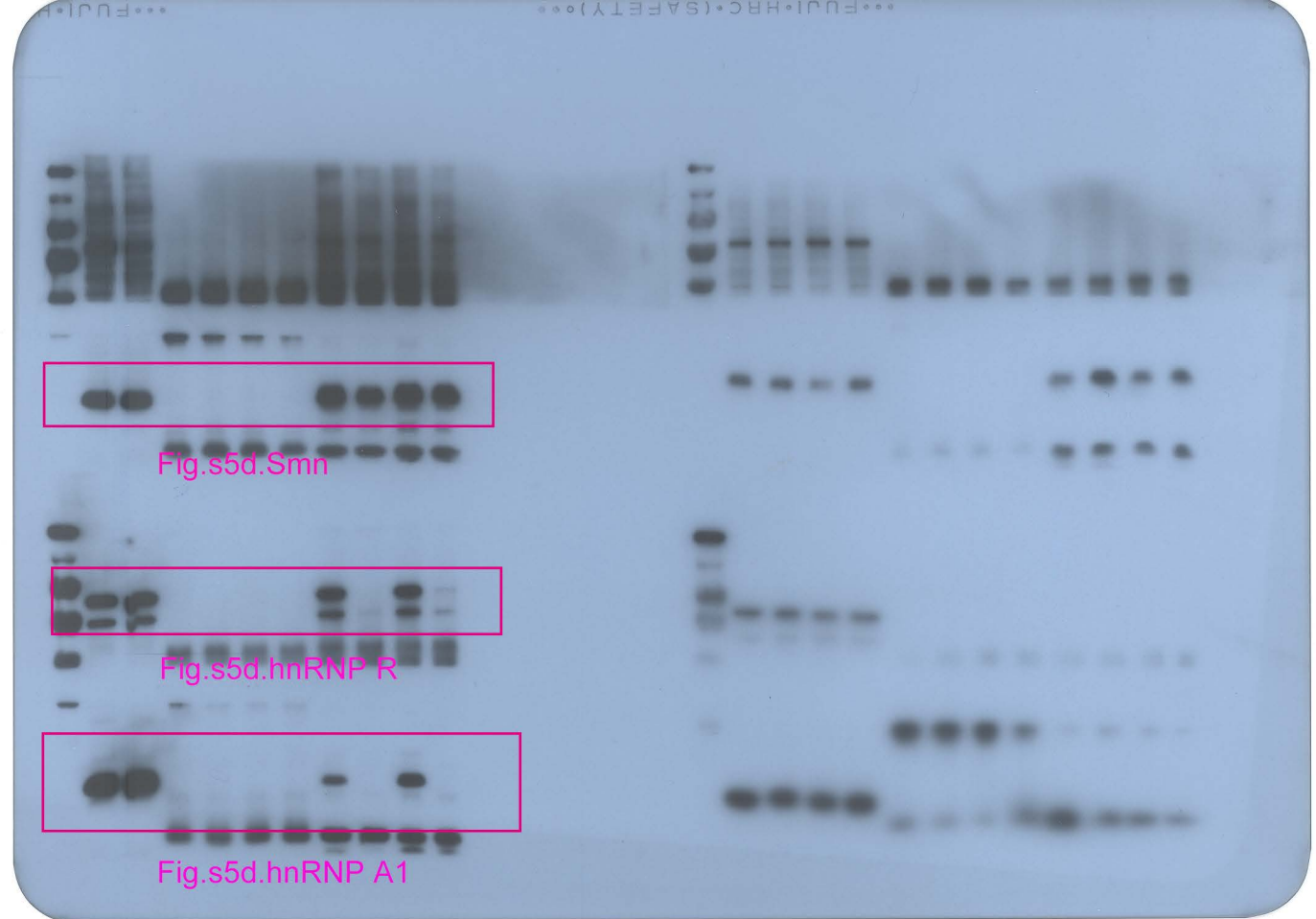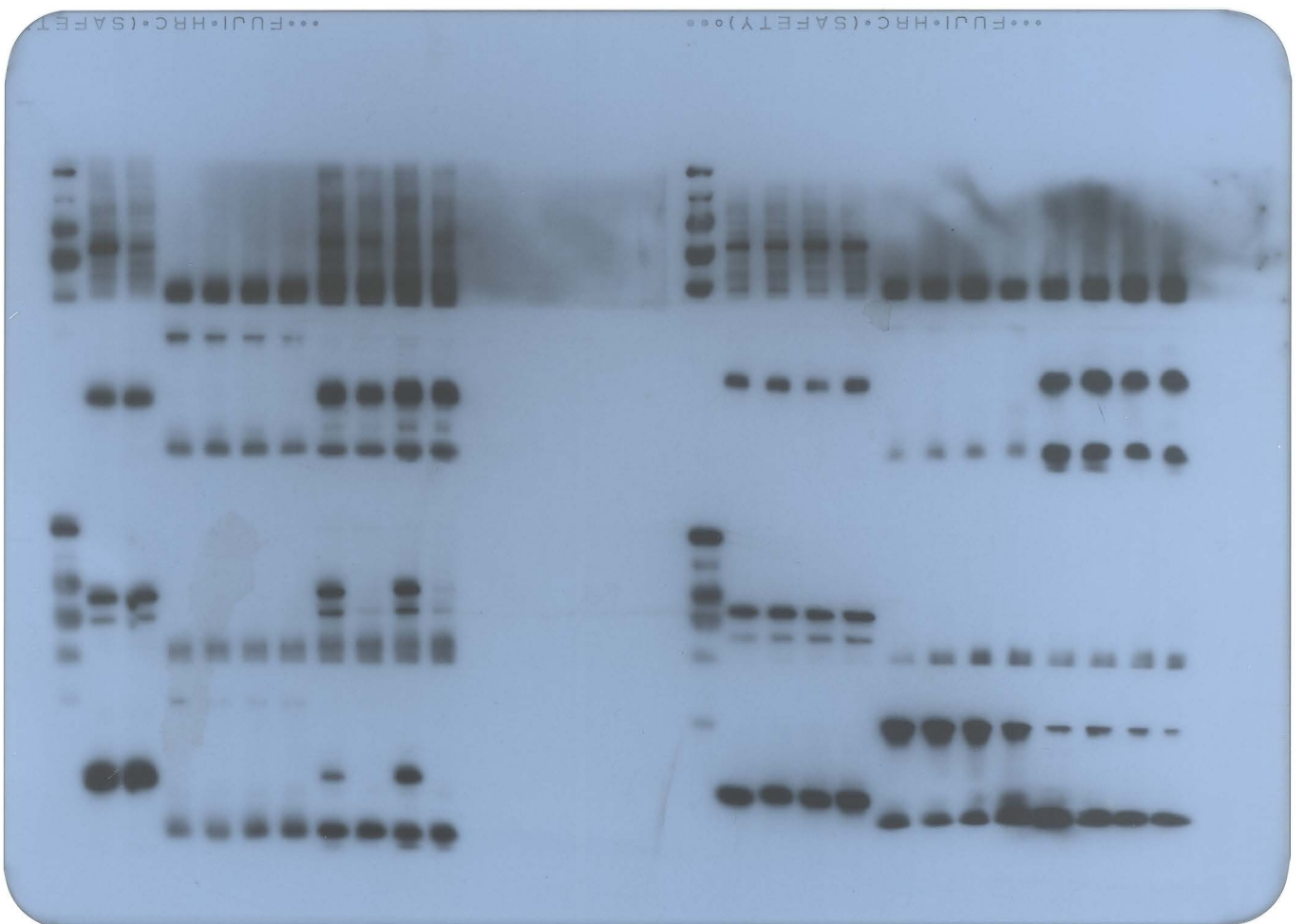

Supplement: Supplementary file 8 — Source Data [file 41467_2021_21529_MOESM8_ESM.zip › Uncropped blot and gel images/FigureS5/FigureS5d/Smn_hnRNP R_hnRNP A1.pdf]

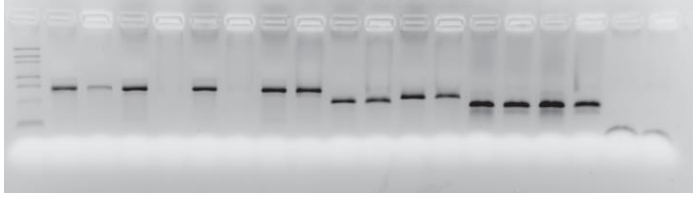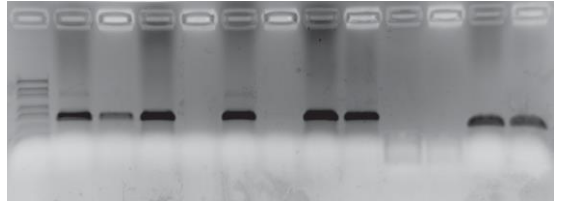

Supplement: Supplementary file 8 — Source Data [file 41467_2021_21529_MOESM8_ESM.zip › Uncropped blot and gel images/FigureS6/FigureS6bd/RNA gels.pdf]

Fig.s6c.Larp7

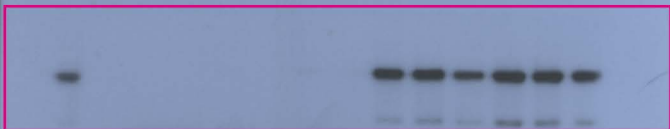

Fig.s6c.Smn

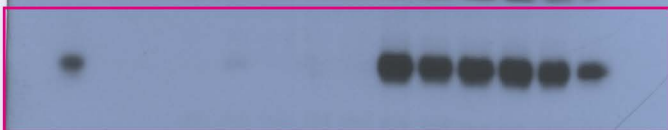

Fig.s6c.hnRNP R

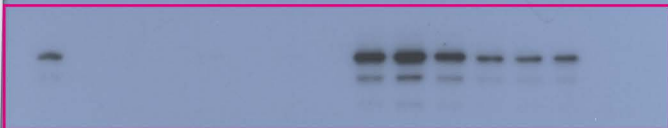

Fig.s6c.hnRNP A1

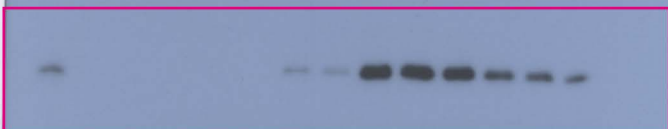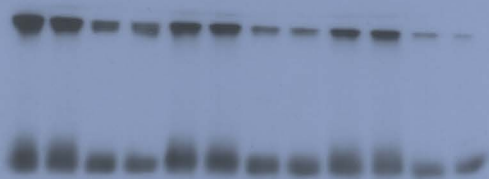

Supplement: Supplementary file 8 — Source Data [file 41467_2021_21529_MOESM8_ESM.zip › Uncropped blot and gel images/FigureS6/FigureS6c/Larp7_Smn_hnRNP R_hnRNP A1.pdf]

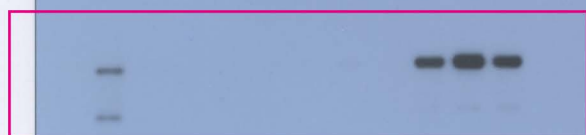

Fig.s6e.Larp7

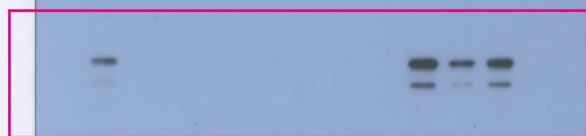

Fig.s6e.hnRNP R

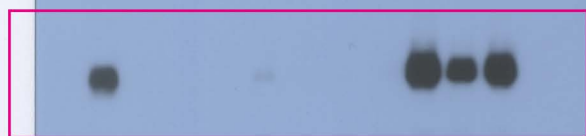

Fig.s6e.Smn

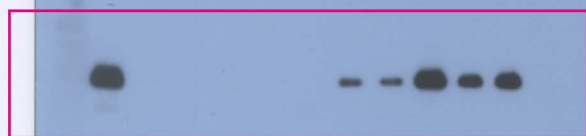

Fig.s6e.hnRNP A1

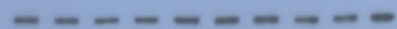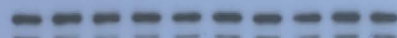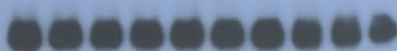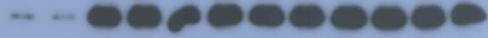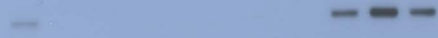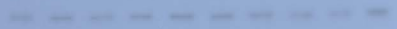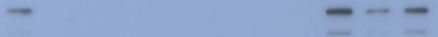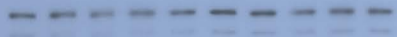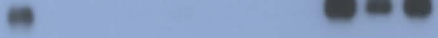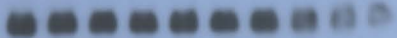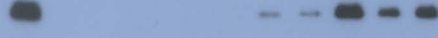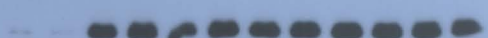

Supplement: Supplementary file 8 — Source Data [file 41467_2021_21529_MOESM8_ESM.zip › Uncropped blot and gel images/FigureS6/FigureS6e/Larp7_Smn_hnRNP R_hnRNP A1.pdf]
