# Supplementary figures and images for: Interaction of 7SK with the Smn complex modulates snRNP production (part 1 of 2)
Source: Nat Commun. 2021 Feb 24;12:1278. doi: 10.1038/s41467-021-21529-1 (PMC7904863; doi:10.1038/s41467-021-21529-1)

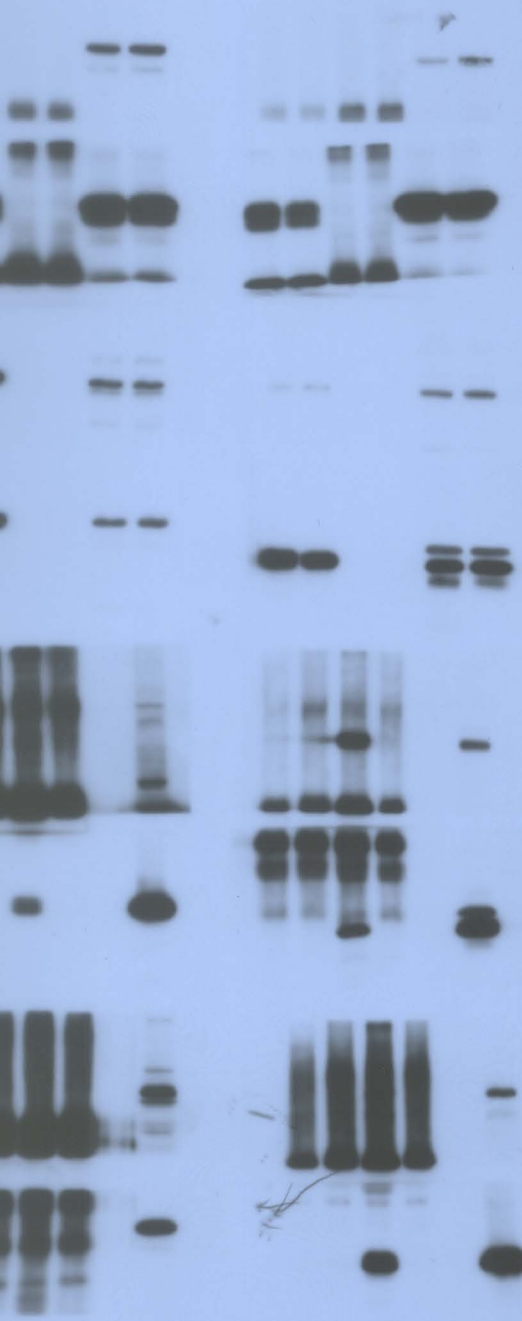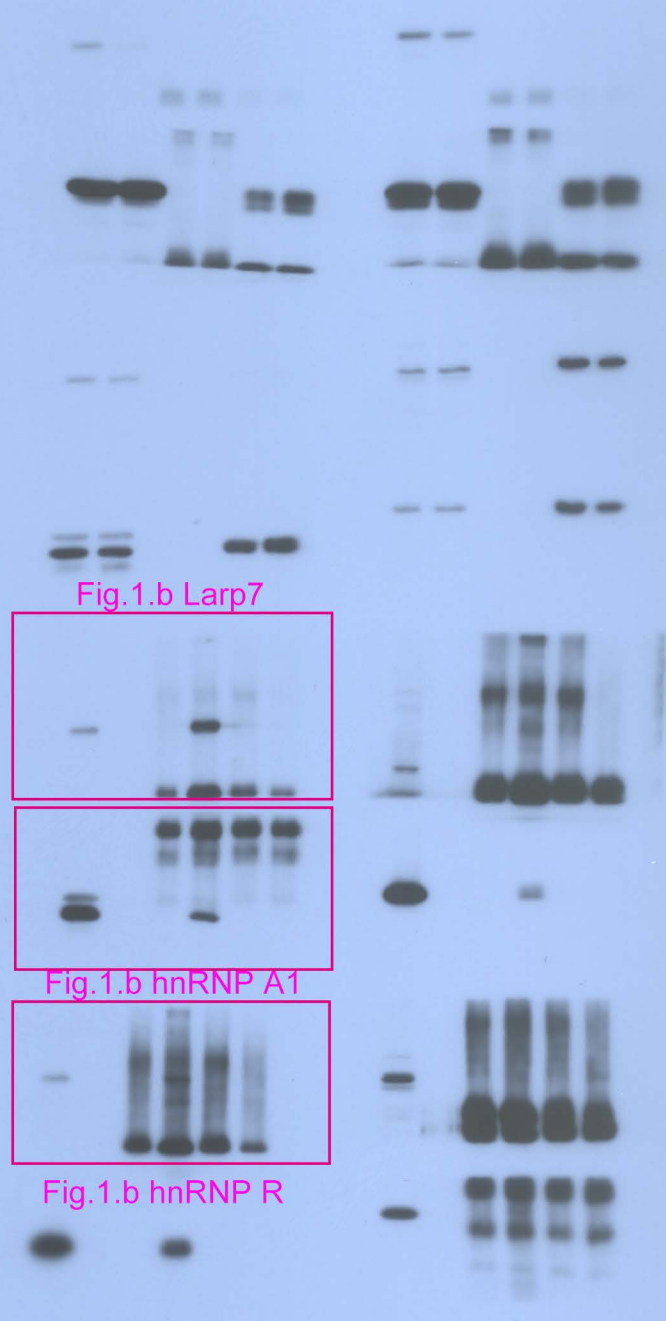

Fig.1.b Larp7

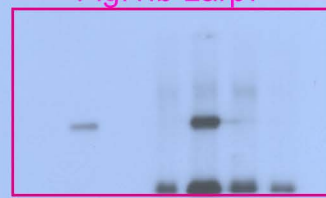

Fig.1.b hnRNP A1

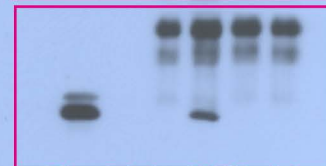

Fig.1.b hnRNP R

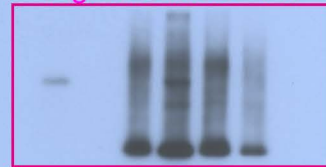

Supplement: Supplementary file 8 — Source Data [file 41467_2021_21529_MOESM8_ESM.zip › Uncropped blot and gel images/Figure1/Figure1b/Larp7_hnRNP R_hnRNP A1.pdf]

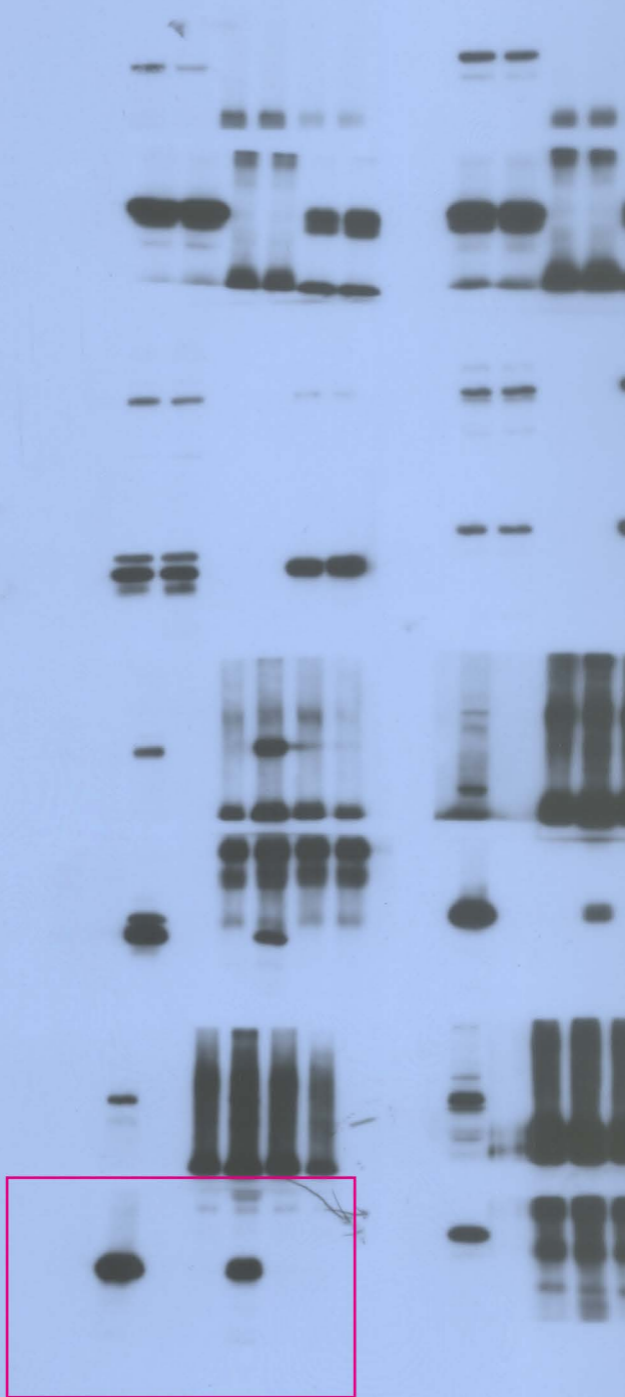

Fig.1b.Smn

Supplement: Supplementary file 8 — Source Data [file 41467_2021_21529_MOESM8_ESM.zip › Uncropped blot and gel images/Figure1/Figure1b/Smn.pdf]

Fig.1c.Larp7

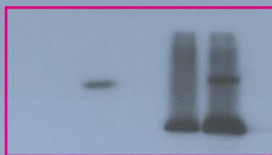

Supplement: Supplementary file 8 — Source Data [file 41467_2021_21529_MOESM8_ESM.zip › Uncropped blot and gel images/Figure1/Figure1c/Larp7.pdf]

Fig.1c.Smn

Fig.1c.SmB/B'

Fig.1c.Gemin2

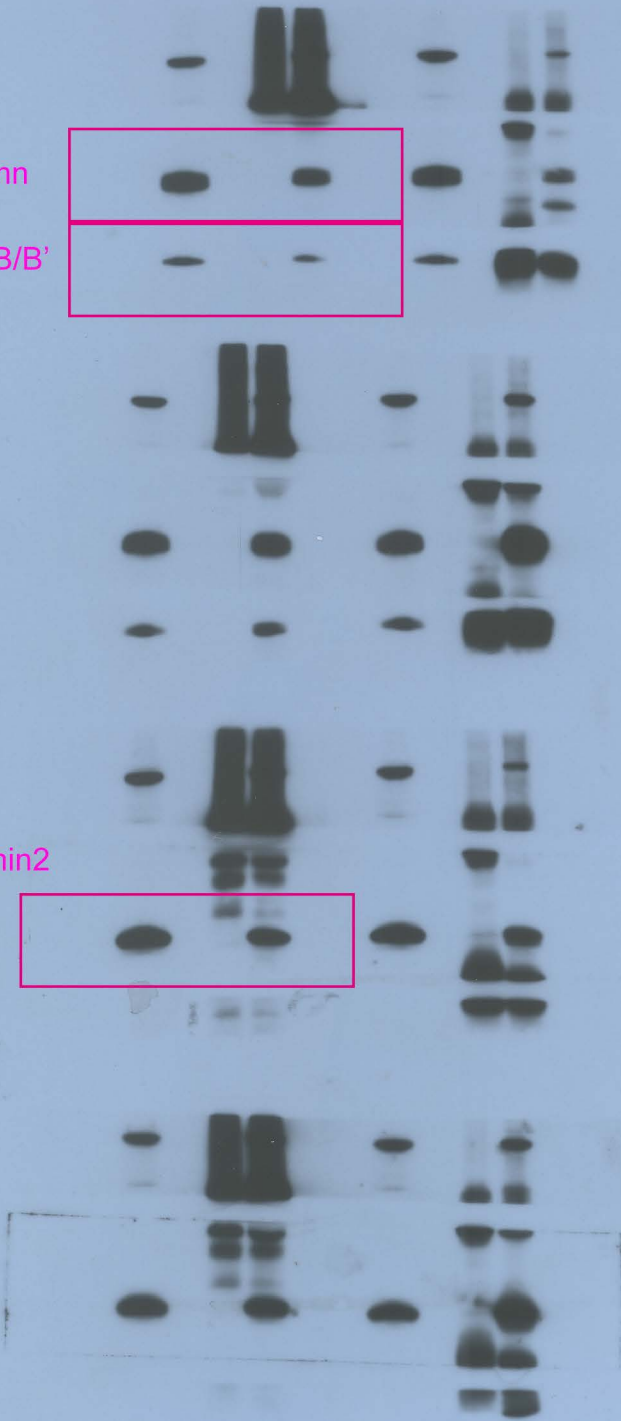

Supplement: Supplementary file 8 — Source Data [file 41467_2021_21529_MOESM8_ESM.zip › Uncropped blot and gel images/Figure1/Figure1c/Smn_SmB_Gemin2.pdf]

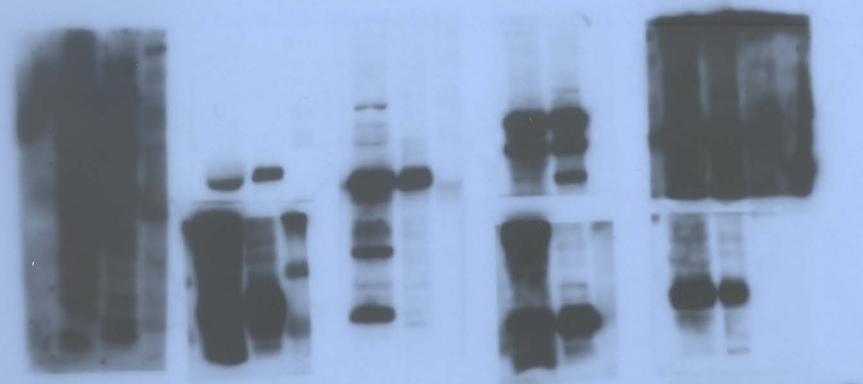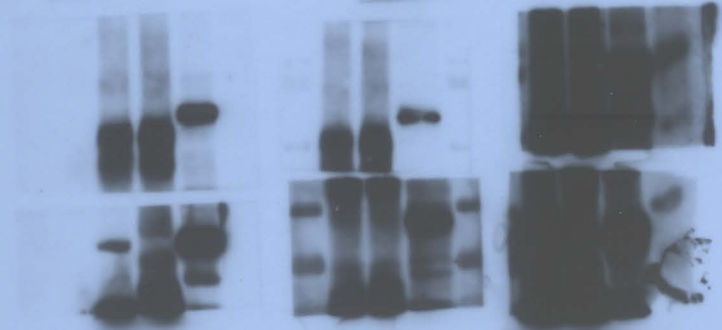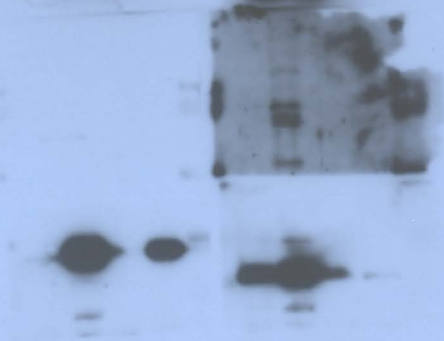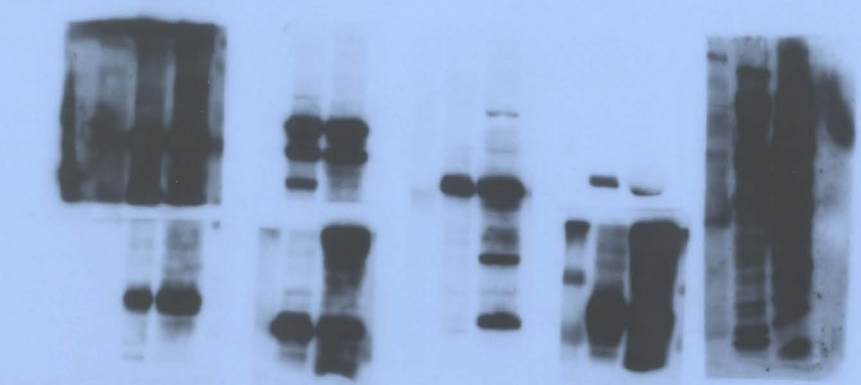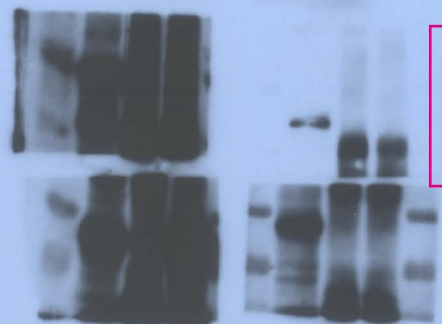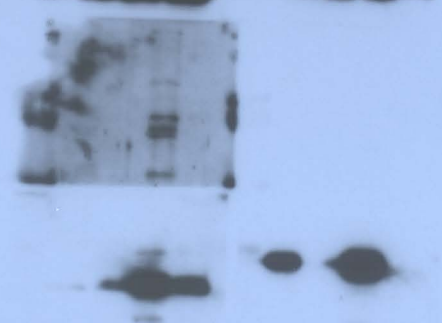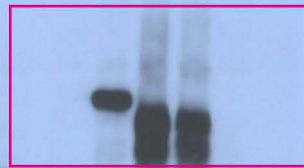

Fig.1d  
Hexim1

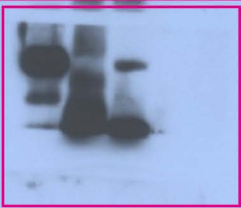

Fig.1d  
hnRNP A1

Supplement: Supplementary file 8 — Source Data [file 41467_2021_21529_MOESM8_ESM.zip › Uncropped blot and gel images/Figure1/Figure1d/Hexim1_hnRNP A1.pdf]

Fig.1d  
hnRNP R

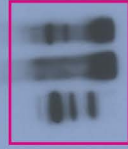

Fig.1d  
Gapdh

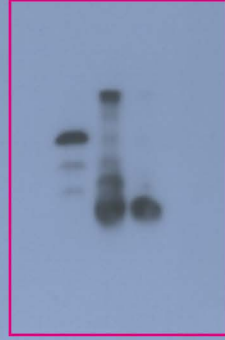

Supplement: Supplementary file 8 — Source Data [file 41467_2021_21529_MOESM8_ESM.zip › Uncropped blot and gel images/Figure1/Figure1d/hnRNP R_Gapdh.pdf]

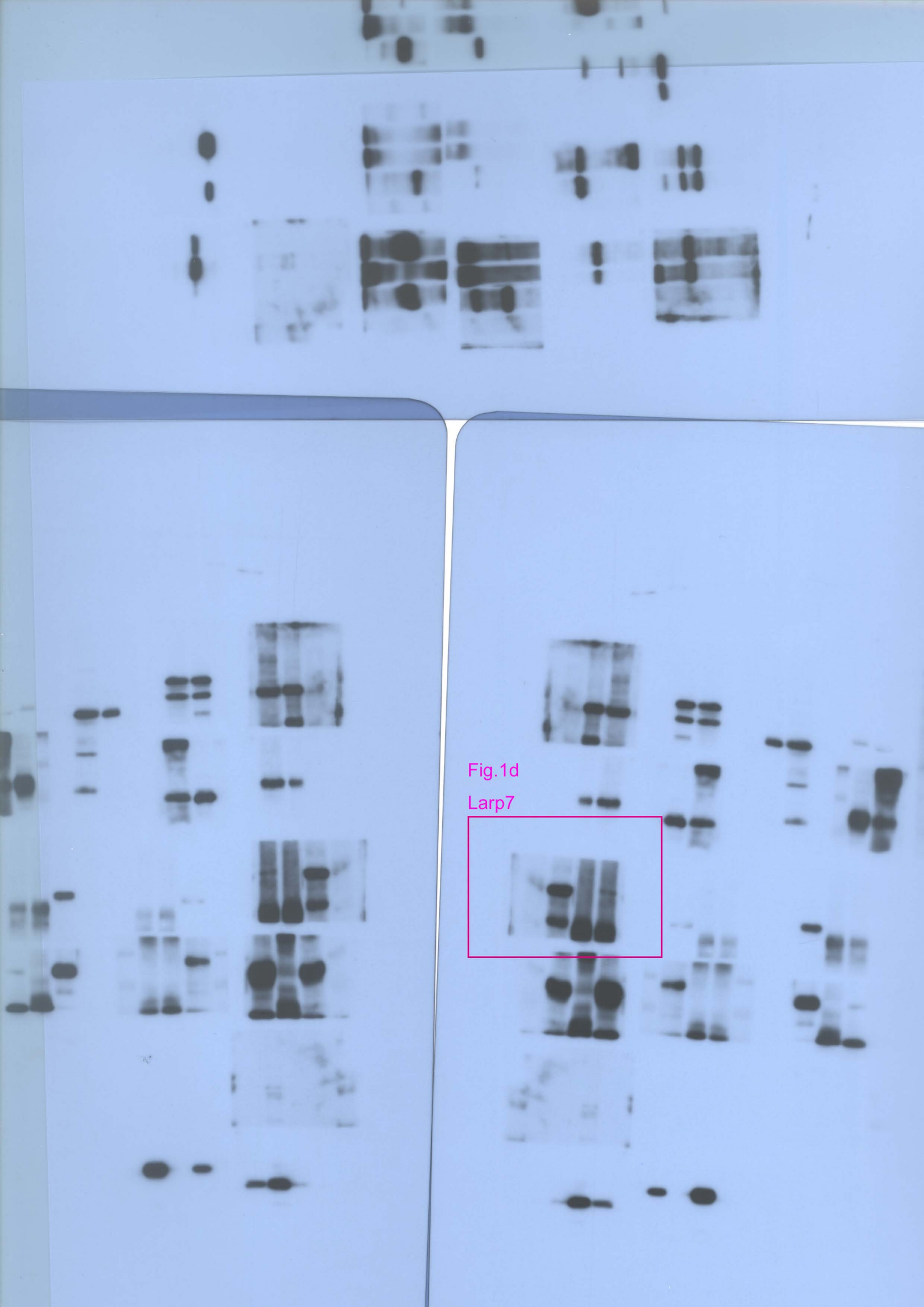

Fig.1d  
Larp7

Supplement: Supplementary file 8 — Source Data [file 41467_2021_21529_MOESM8_ESM.zip › Uncropped blot and gel images/Figure1/Figure1d/Larp7.pdf]

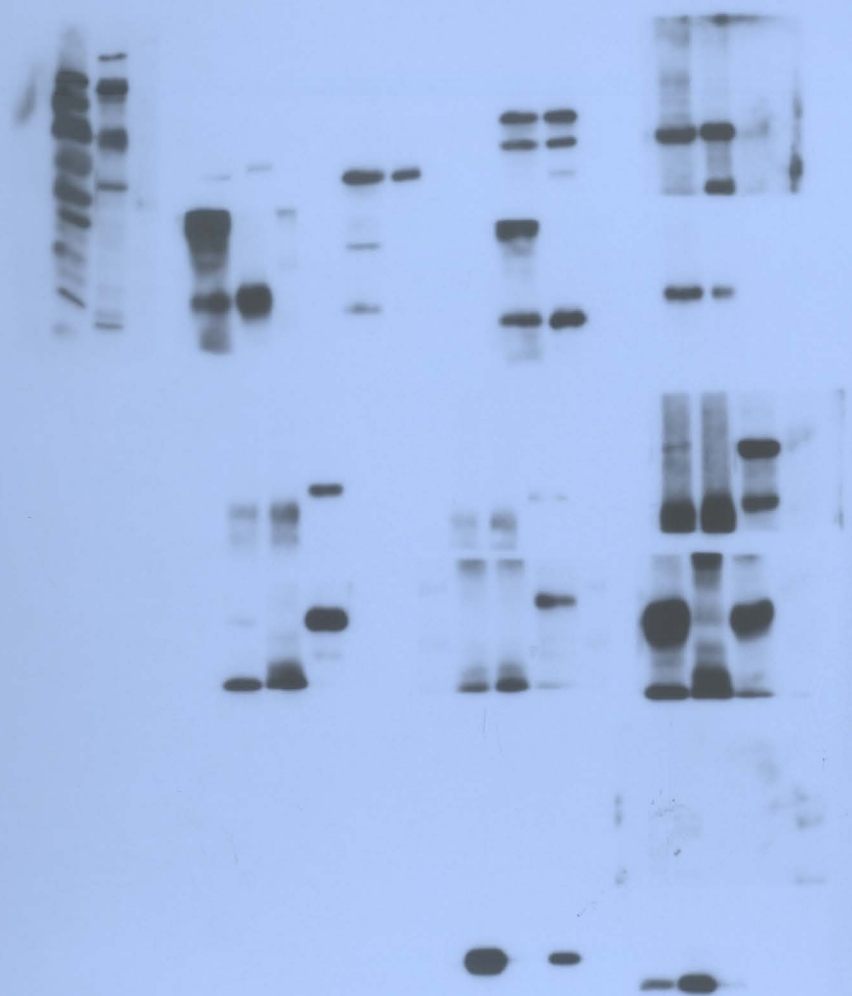

Fig.1d  
Smn

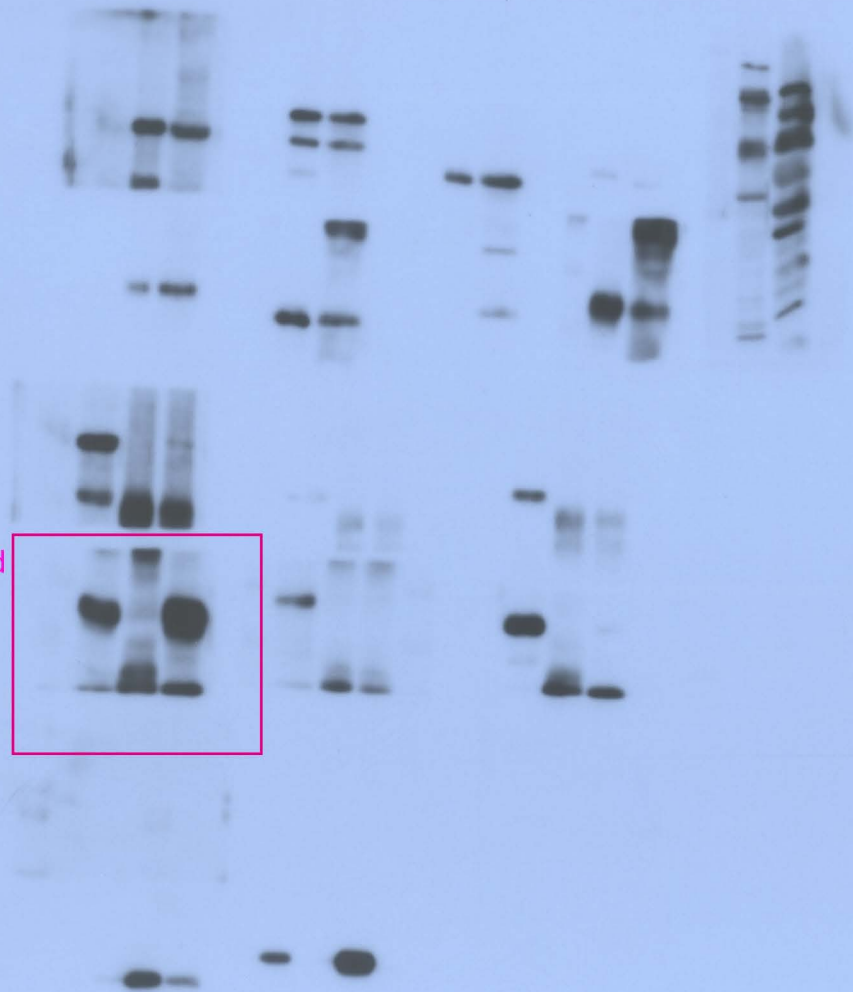

Supplement: Supplementary file 8 — Source Data [file 41467_2021_21529_MOESM8_ESM.zip › Uncropped blot and gel images/Figure1/Figure1d/Smn.pdf]

V-t2 fischer's student

Ji with whole

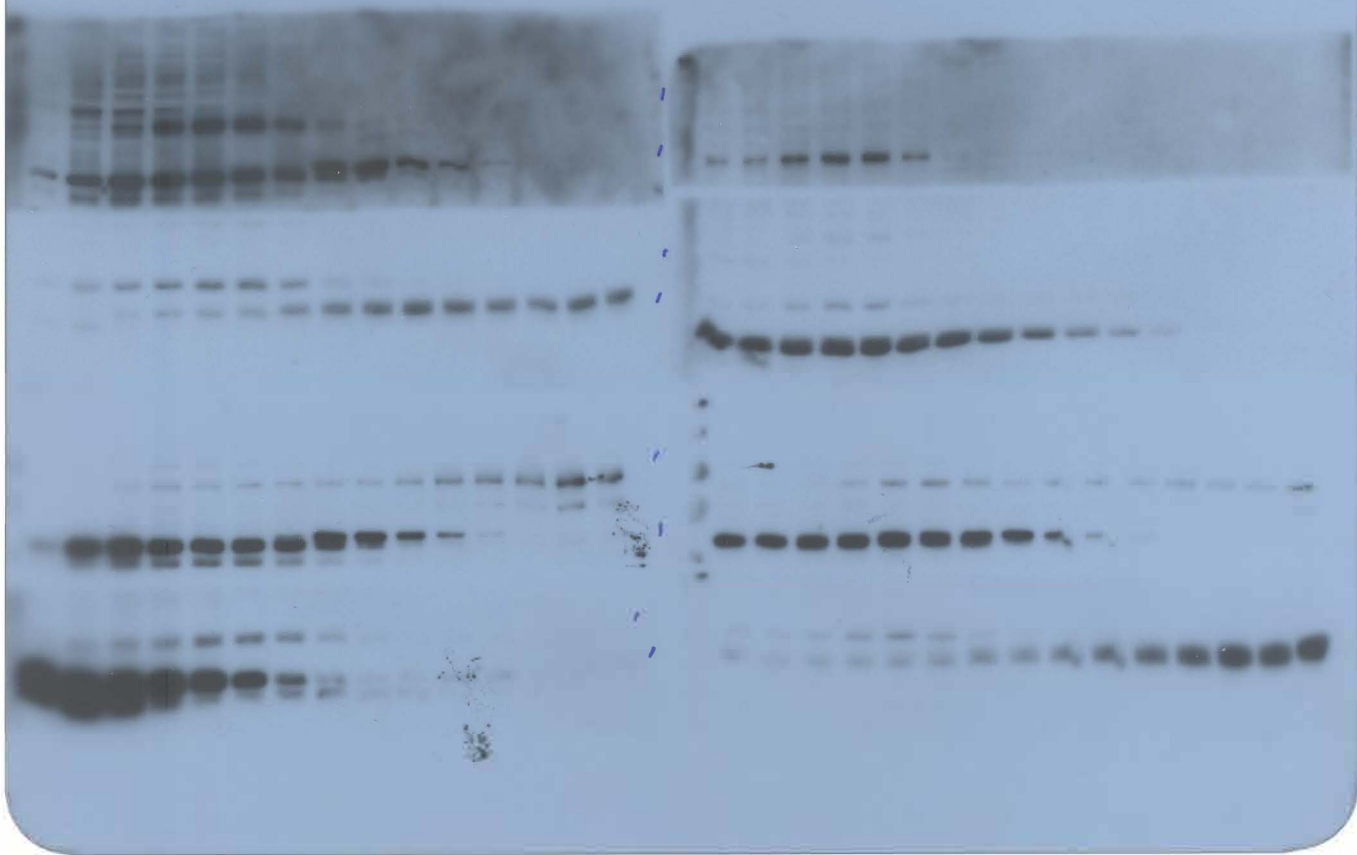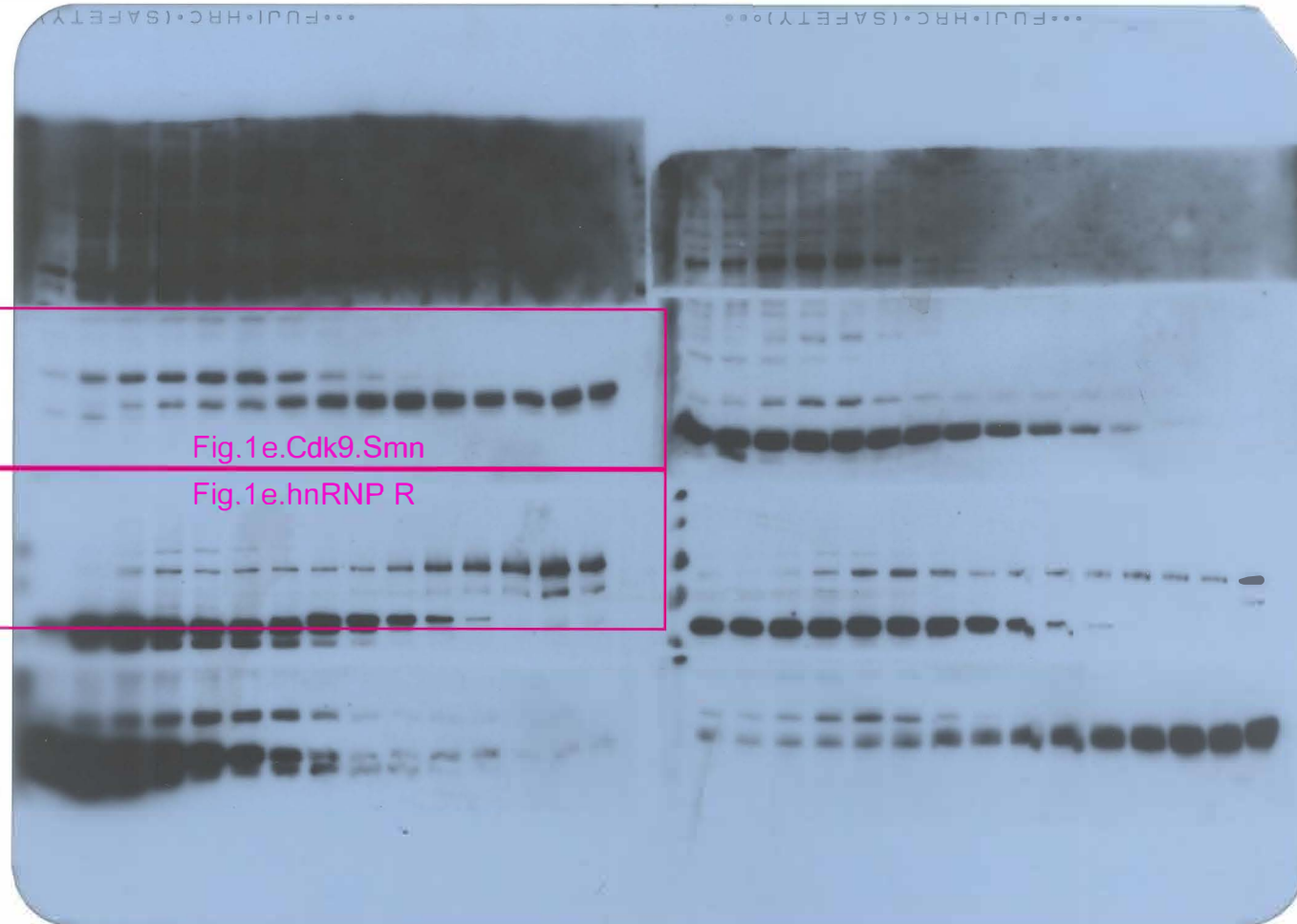

Fig.1e.Cdk9.Smn

Fig.1e.hnRNP R

Supplement: Supplementary file 8 — Source Data [file 41467_2021_21529_MOESM8_ESM.zip › Uncropped blot and gel images/Figure1/Figure1e/Cdk9_Smn_hnRNP R.pdf]

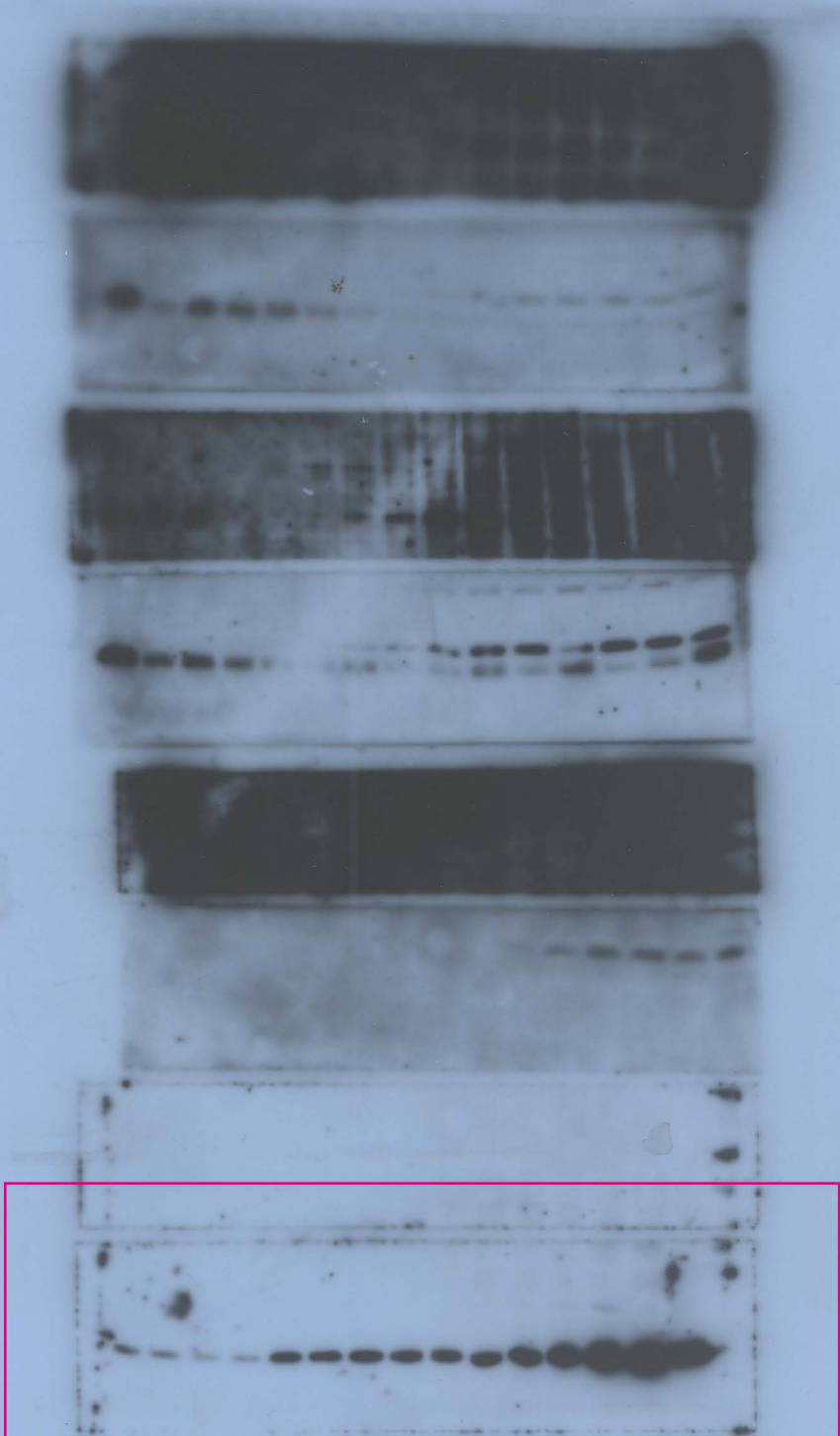

Fig.1e.Gemin2

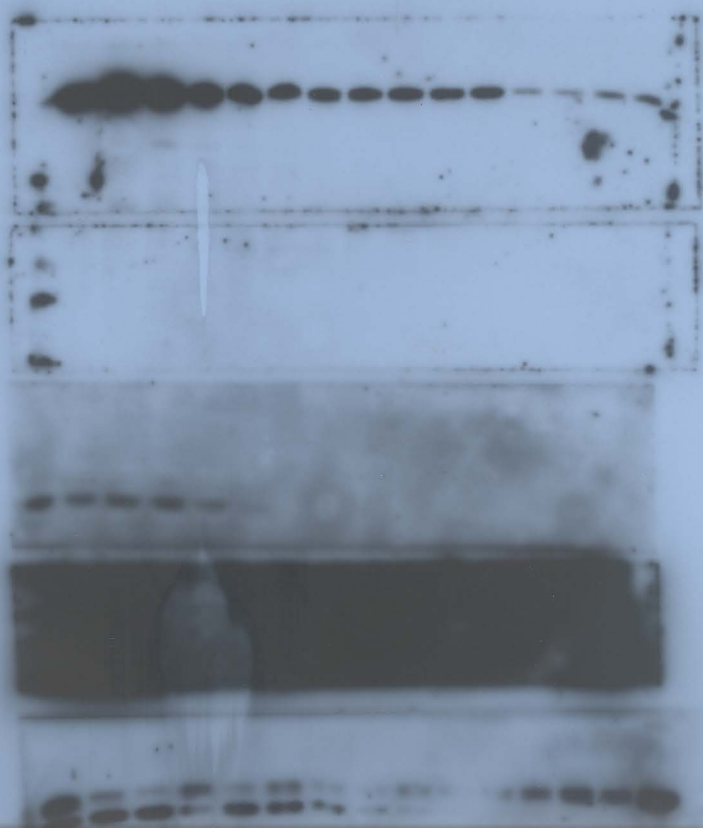

Supplement: Supplementary file 8 — Source Data [file 41467_2021_21529_MOESM8_ESM.zip › Uncropped blot and gel images/Figure1/Figure1e/Gemin2.pdf]

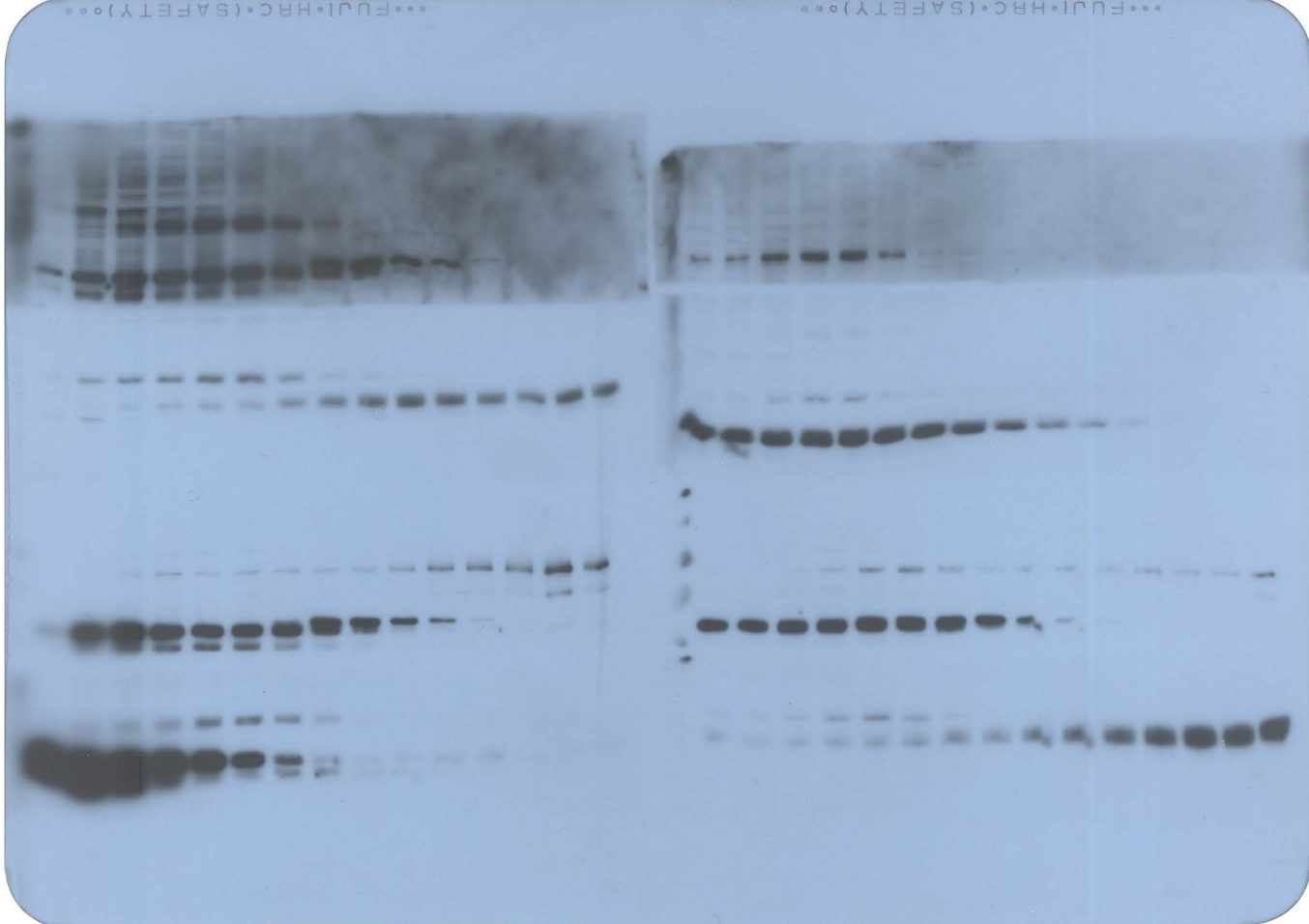

Fig.1e.Hexim1

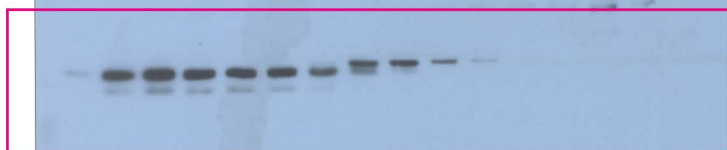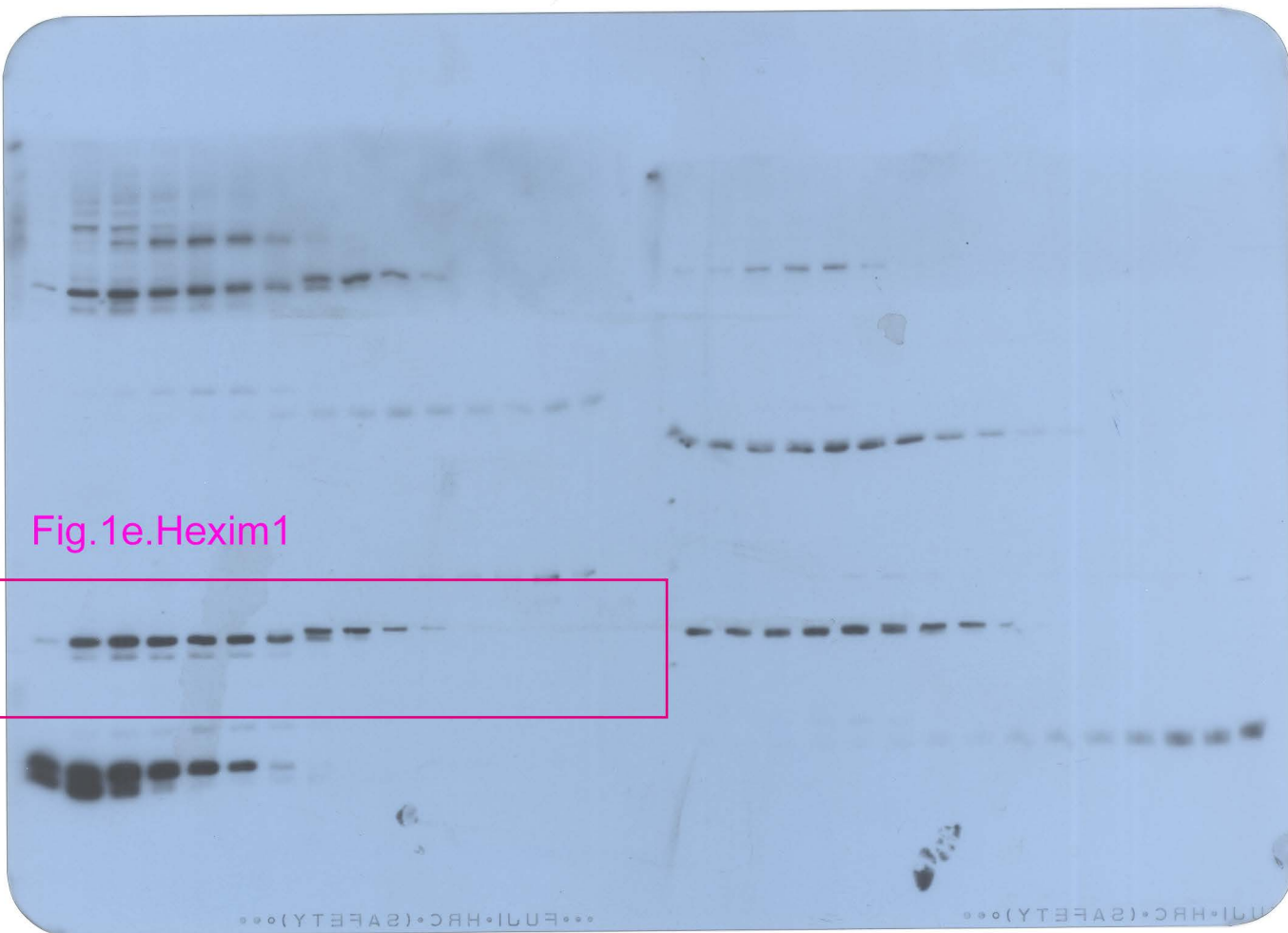

Supplement: Supplementary file 8 — Source Data [file 41467_2021_21529_MOESM8_ESM.zip › Uncropped blot and gel images/Figure1/Figure1e/Hexim1.pdf]

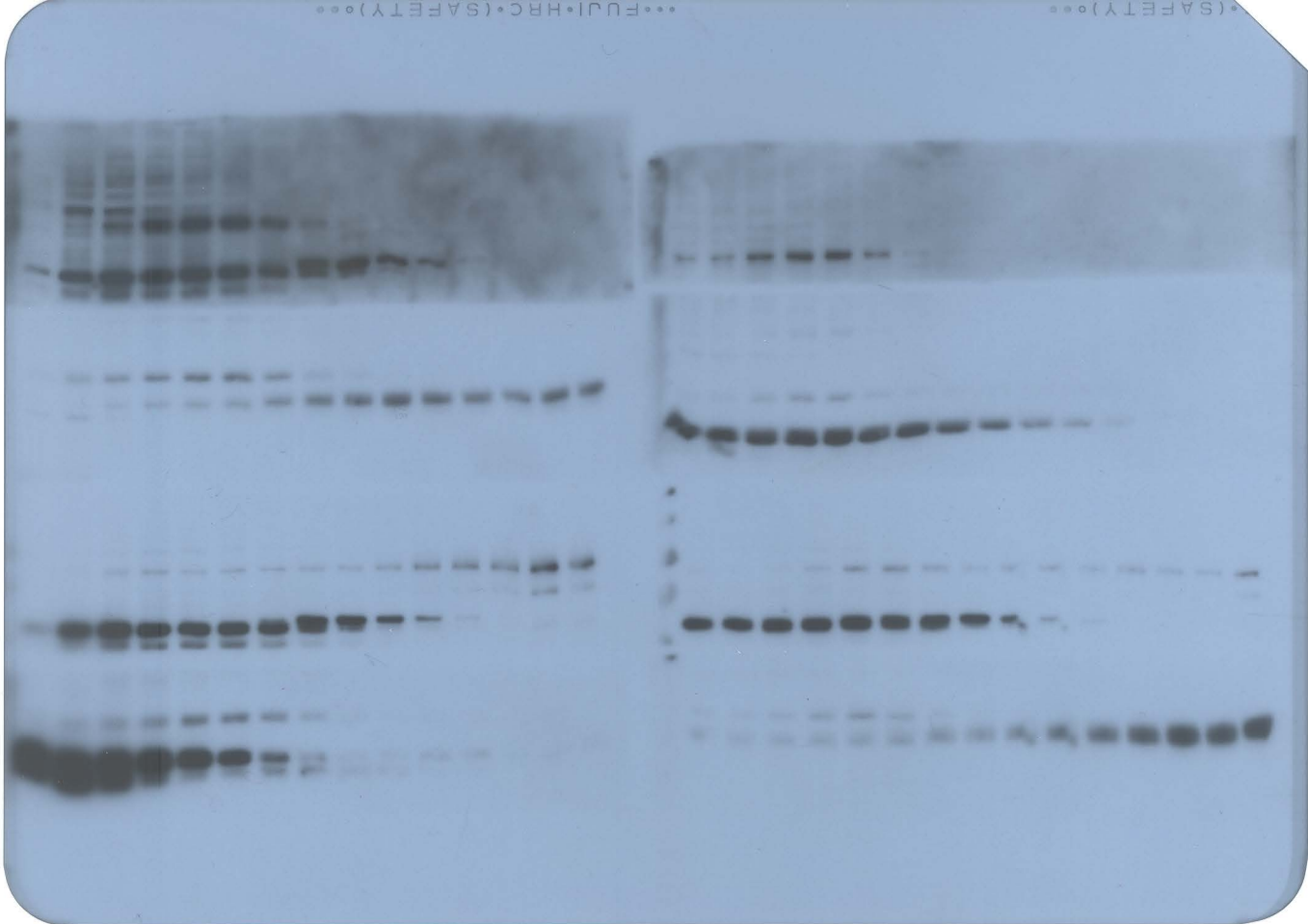

Fig.1e.Larp7

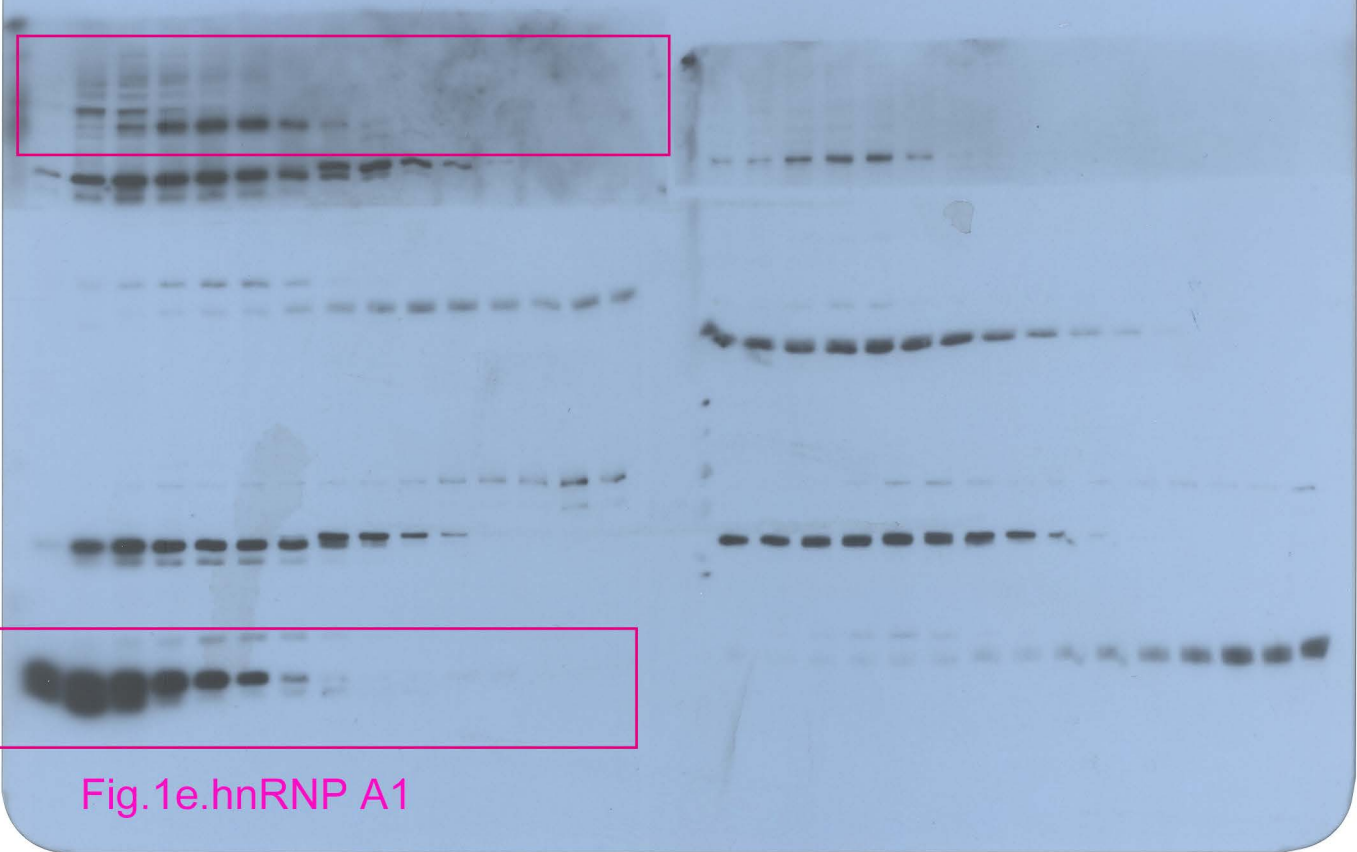

Fig.1e.hnRNP A1

Supplement: Supplementary file 8 — Source Data [file 41467_2021_21529_MOESM8_ESM.zip › Uncropped blot and gel images/Figure1/Figure1e/Larp7_hnRNP A1.pdf]

Fig.1e.SmB/B'

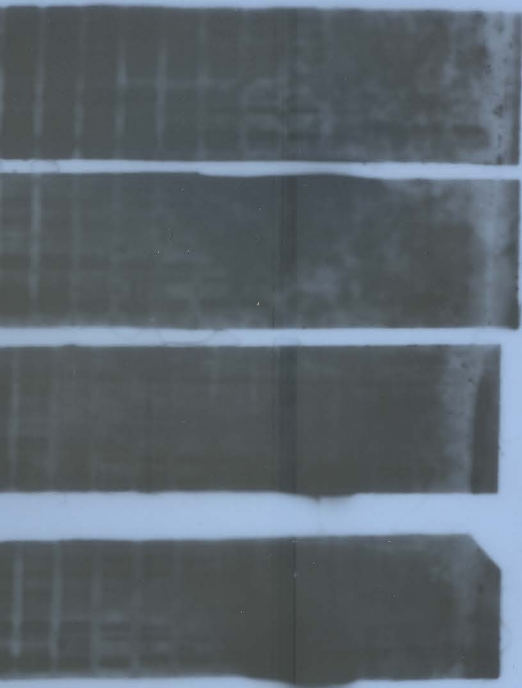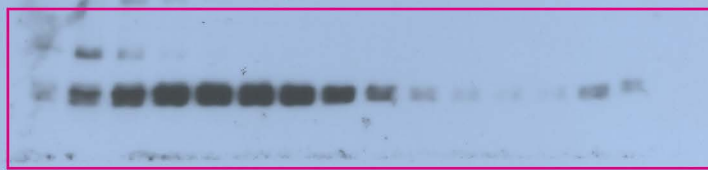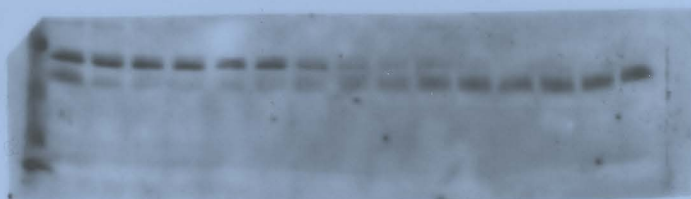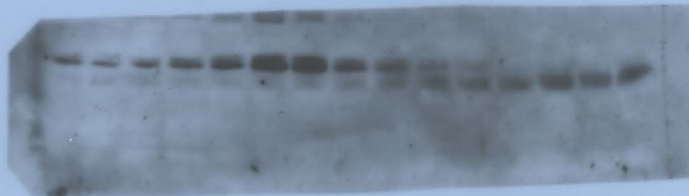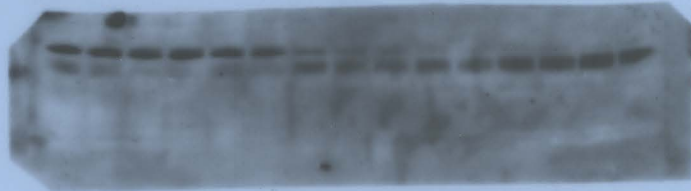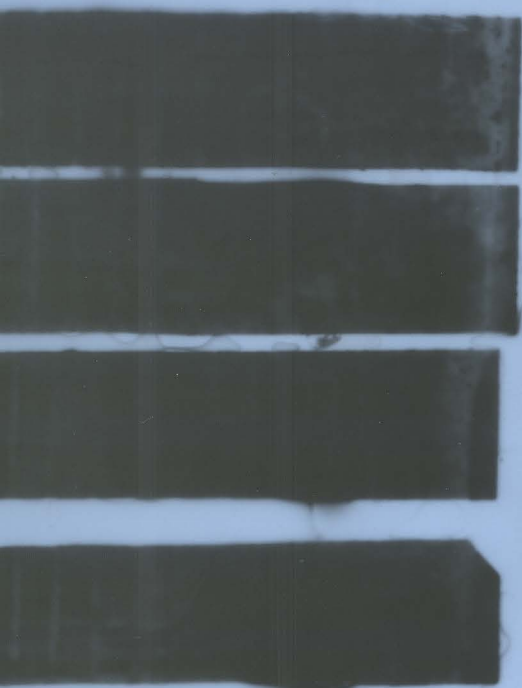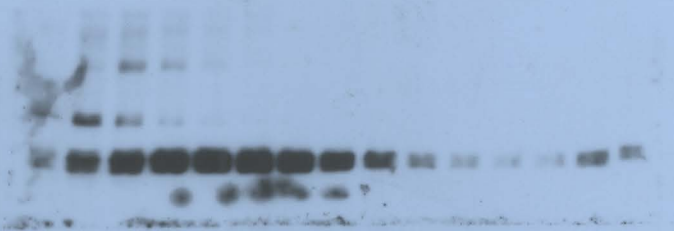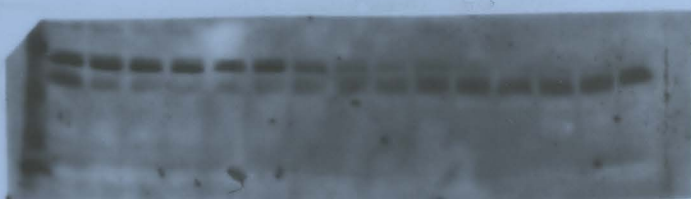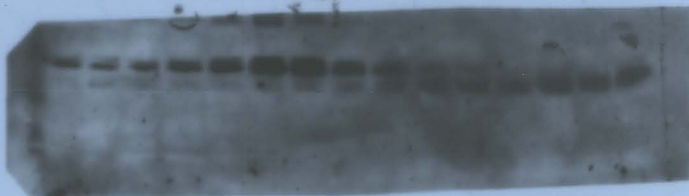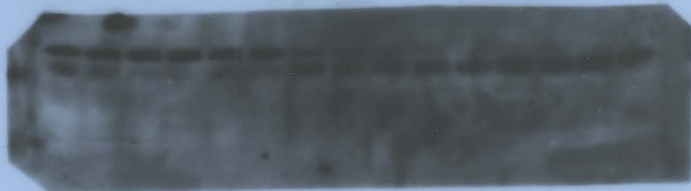

Supplement: Supplementary file 8 — Source Data [file 41467_2021_21529_MOESM8_ESM.zip › Uncropped blot and gel images/Figure1/Figure1e/SmB.pdf]

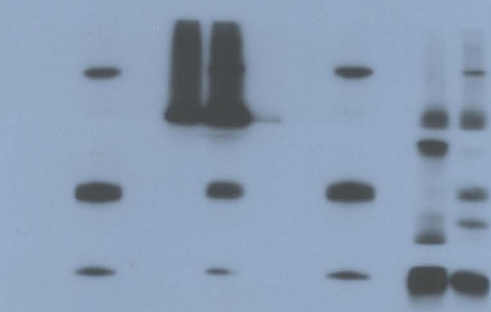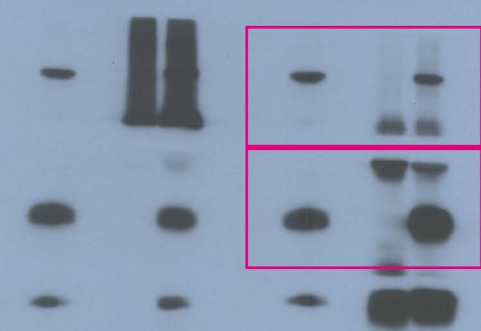

Fig.1f.Larp7

Fig.1f.Smn

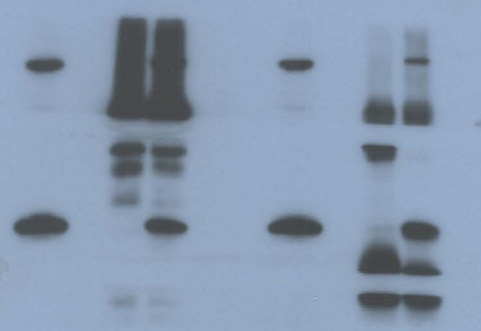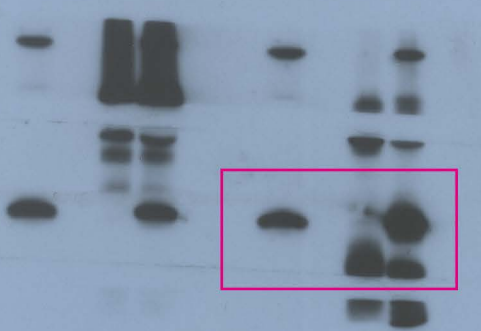

Fig.1f.Gemin2

Supplement: Supplementary file 8 — Source Data [file 41467_2021_21529_MOESM8_ESM.zip › Uncropped blot and gel images/Figure1/Figure1f/Larp7_Smn_Gemin2.pdf]

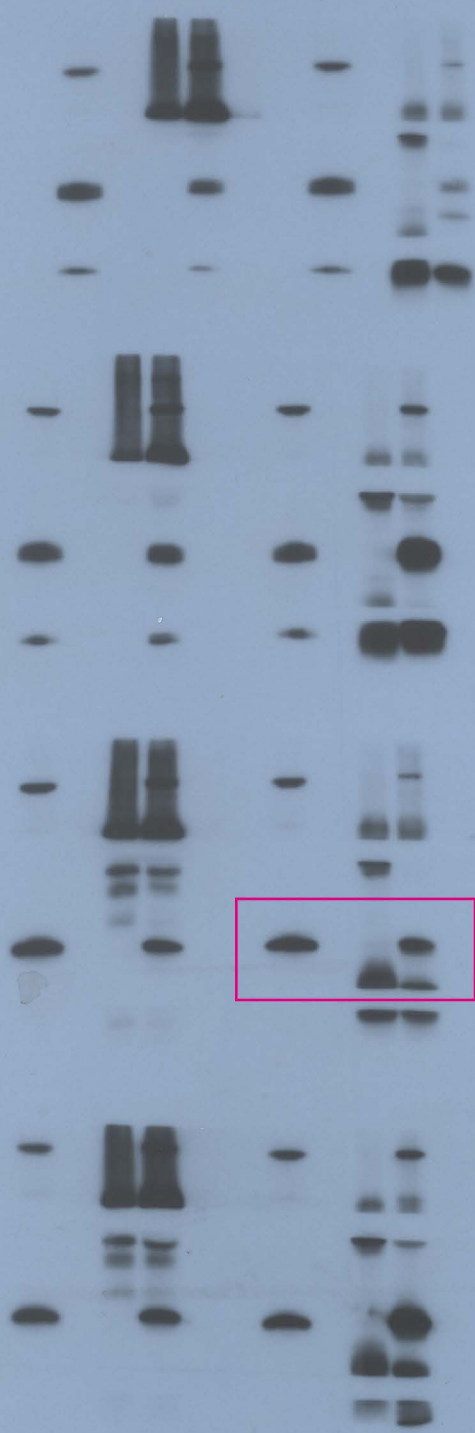

Fig.1h.Gemin2

Supplement: Supplementary file 8 — Source Data [file 41467_2021_21529_MOESM8_ESM.zip › Uncropped blot and gel images/Figure1/Figure1h/Gemin2.pdf]

Fig.1h.Larp7

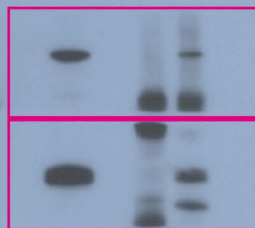

Fig.1h.  
Smn

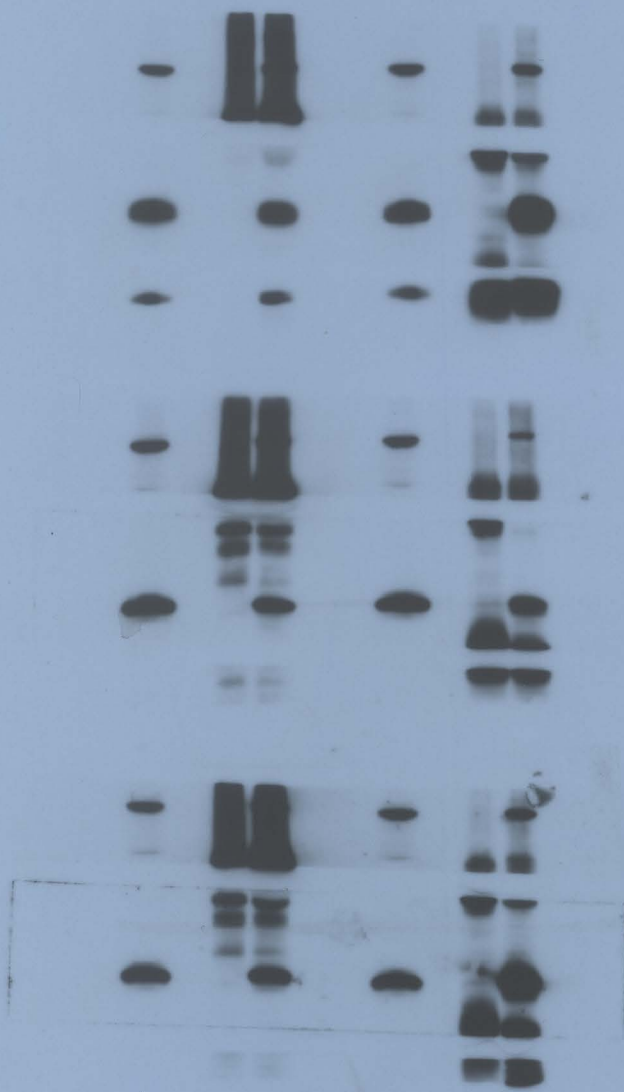

Supplement: Supplementary file 8 — Source Data [file 41467_2021_21529_MOESM8_ESM.zip › Uncropped blot and gel images/Figure1/Figure1h/Larp7_Smn.pdf]

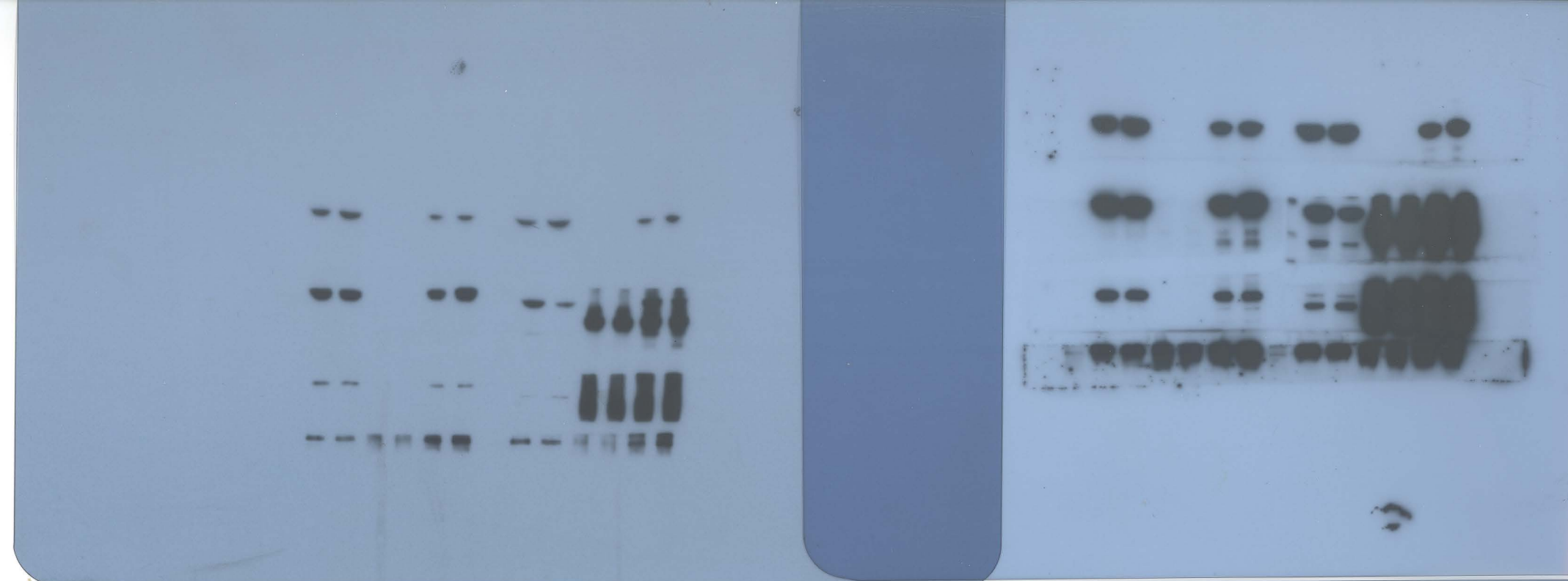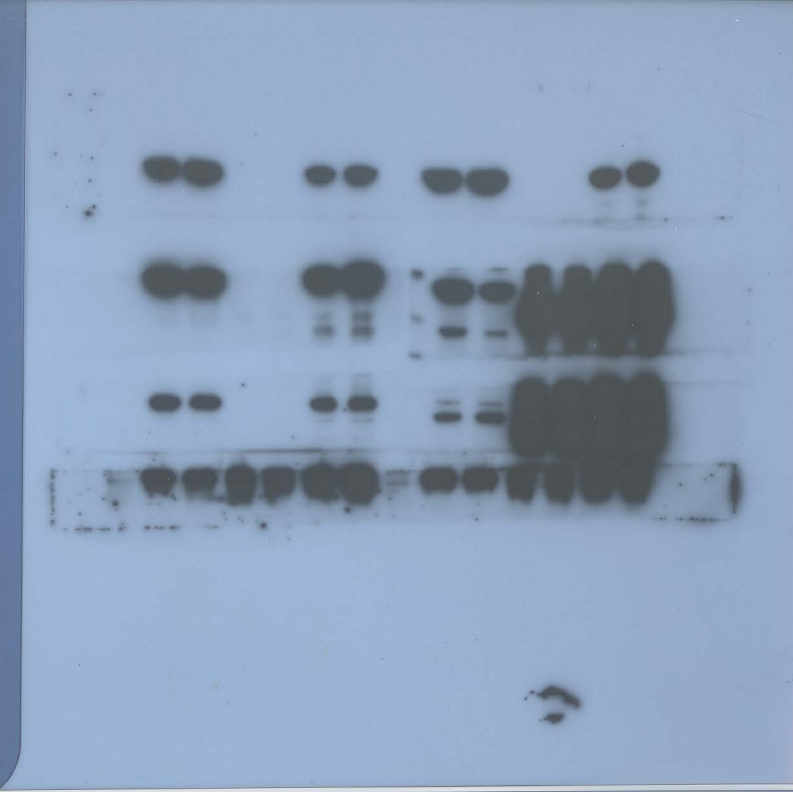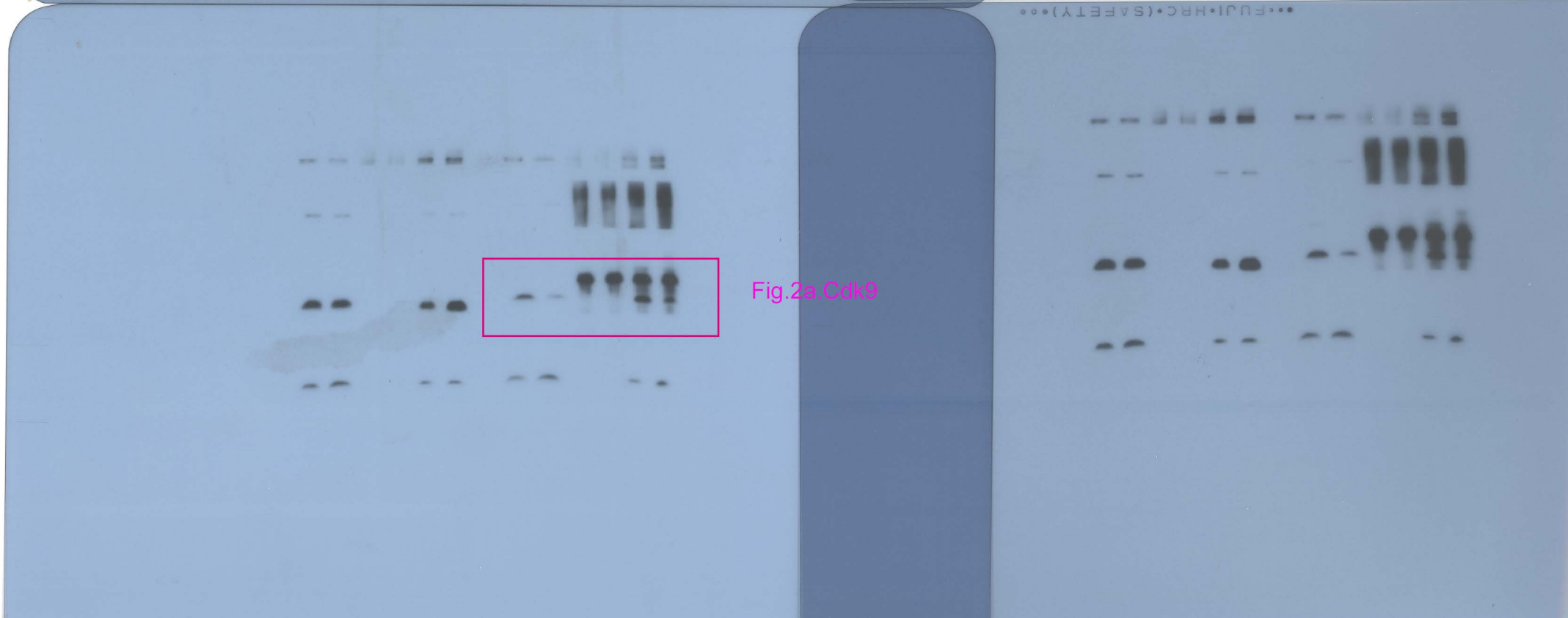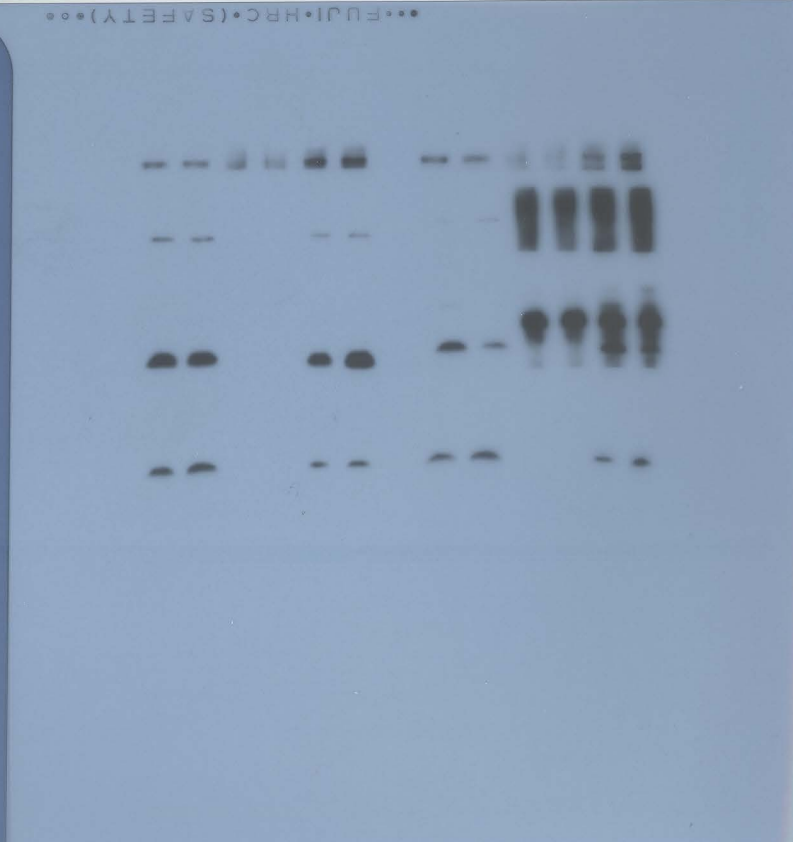

Fig.2a.Cdk9

Supplement: Supplementary file 8 — Source Data [file 41467_2021_21529_MOESM8_ESM.zip › Uncropped blot and gel images/Figure2/Figure2a/Cdk9.pdf]

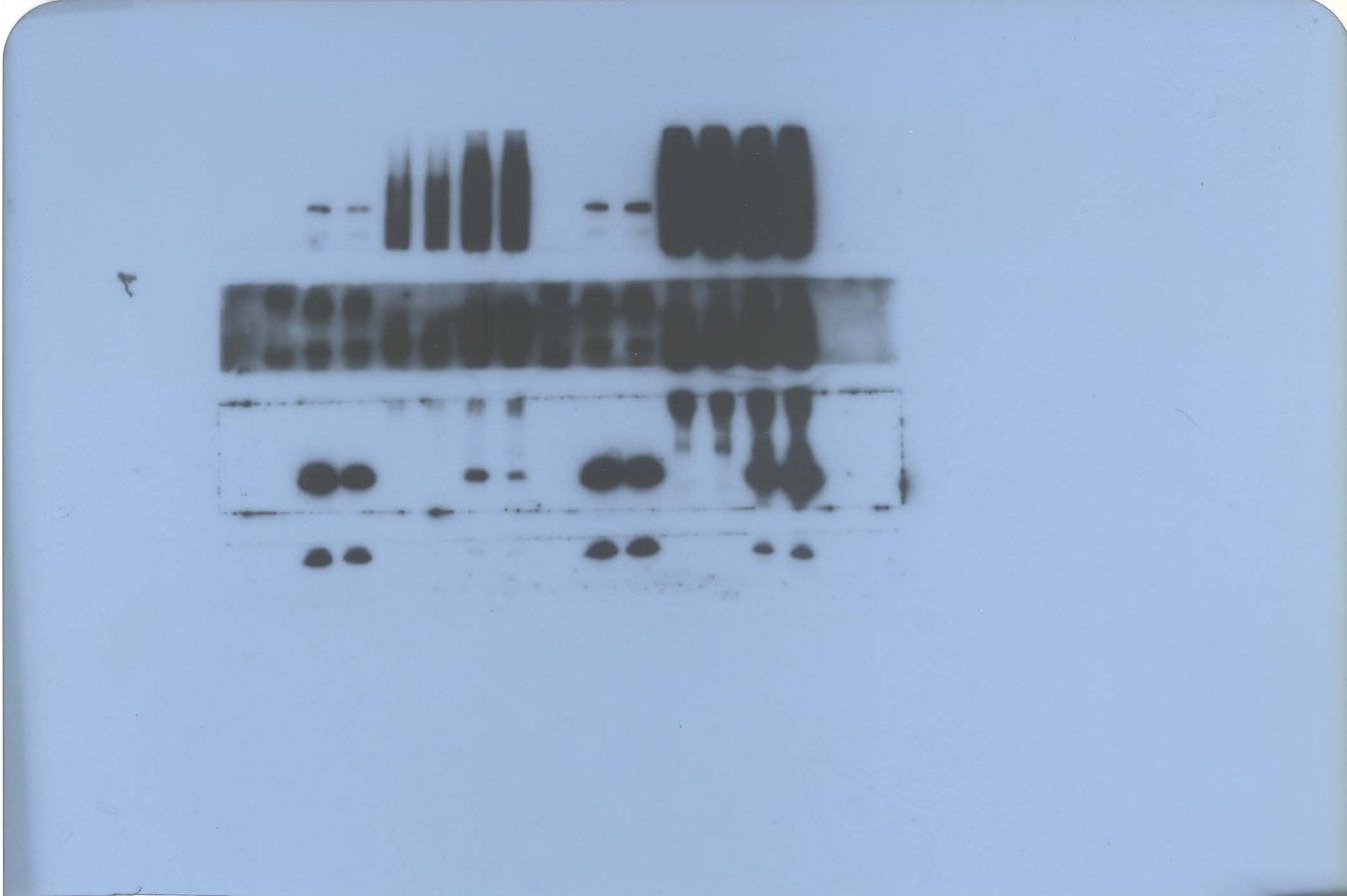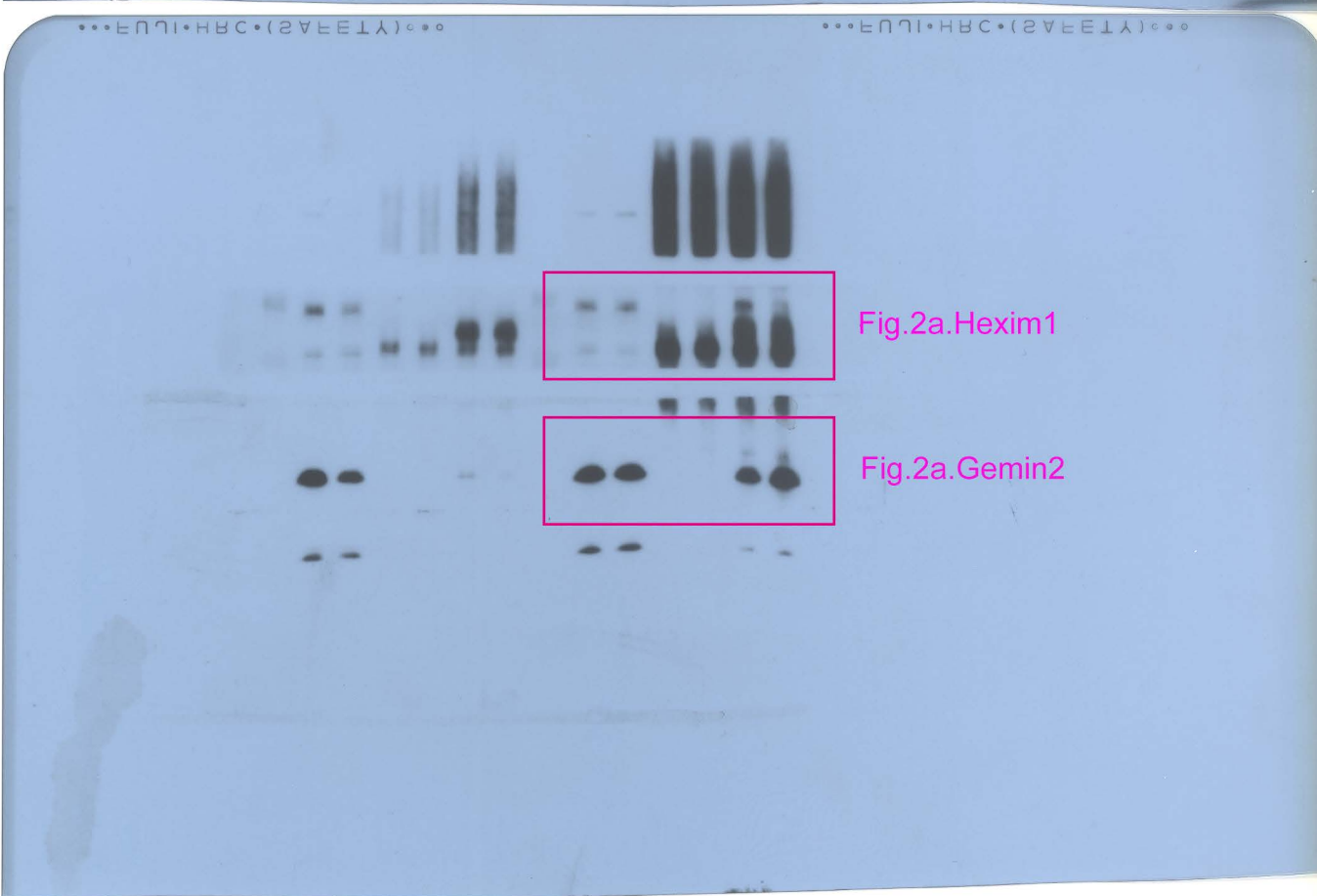

Supplement: Supplementary file 8 — Source Data [file 41467_2021_21529_MOESM8_ESM.zip › Uncropped blot and gel images/Figure2/Figure2a/Hexim1_Gemin2.pdf]

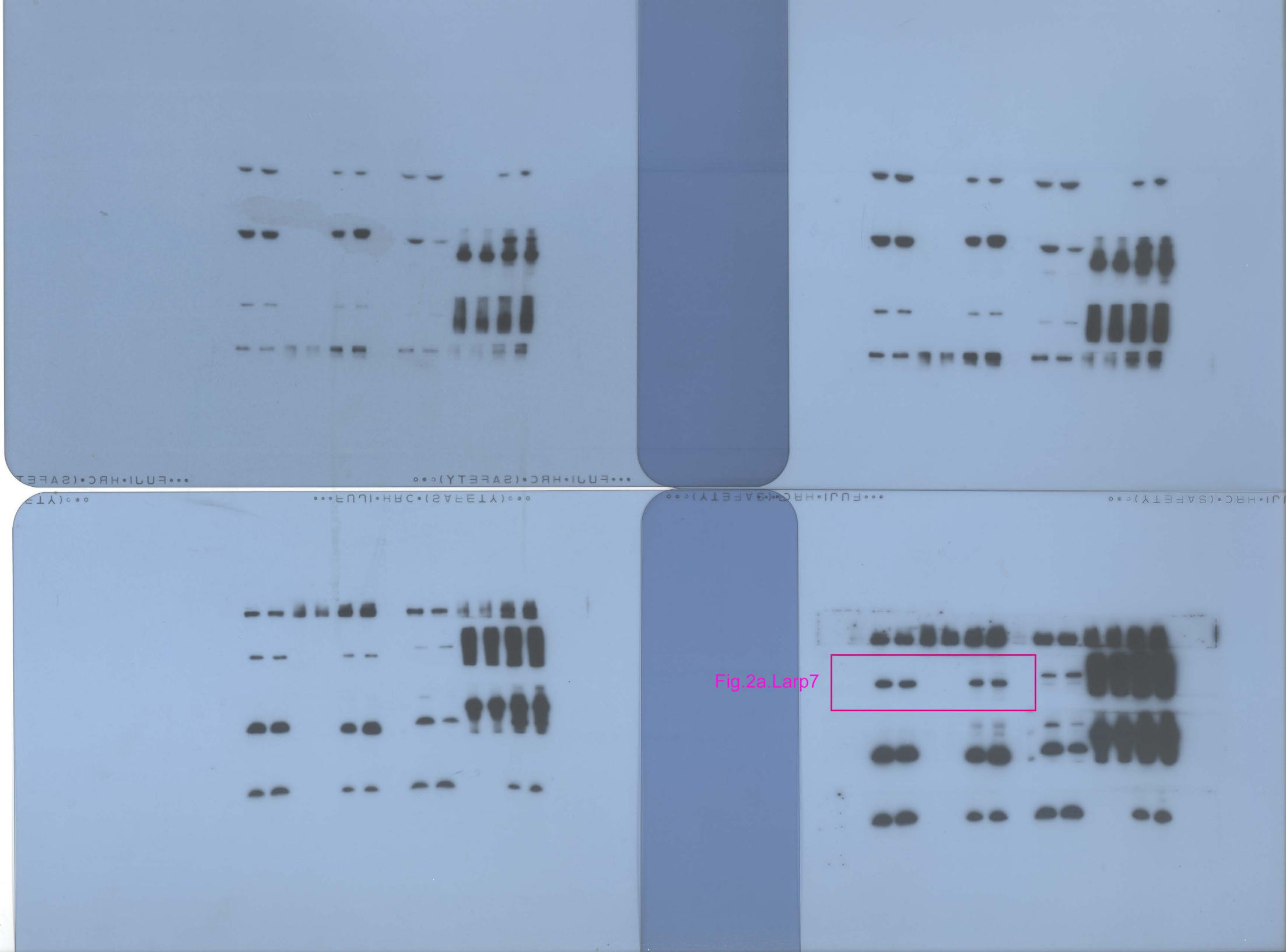

Supplement: Supplementary file 8 — Source Data [file 41467_2021_21529_MOESM8_ESM.zip › Uncropped blot and gel images/Figure2/Figure2a/Larp7.pdf]

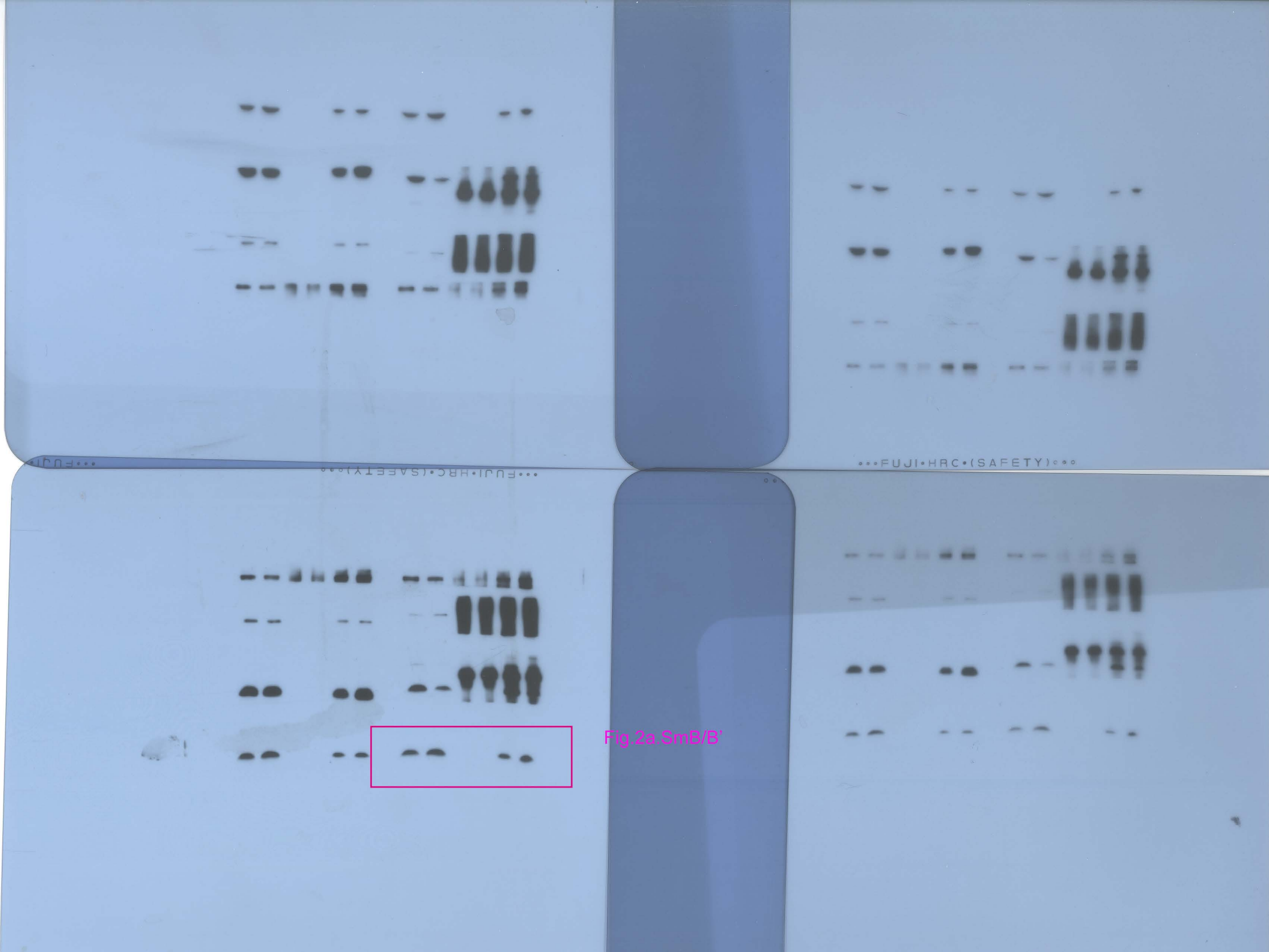

Supplement: Supplementary file 8 — Source Data [file 41467_2021_21529_MOESM8_ESM.zip › Uncropped blot and gel images/Figure2/Figure2a/SmB.pdf]

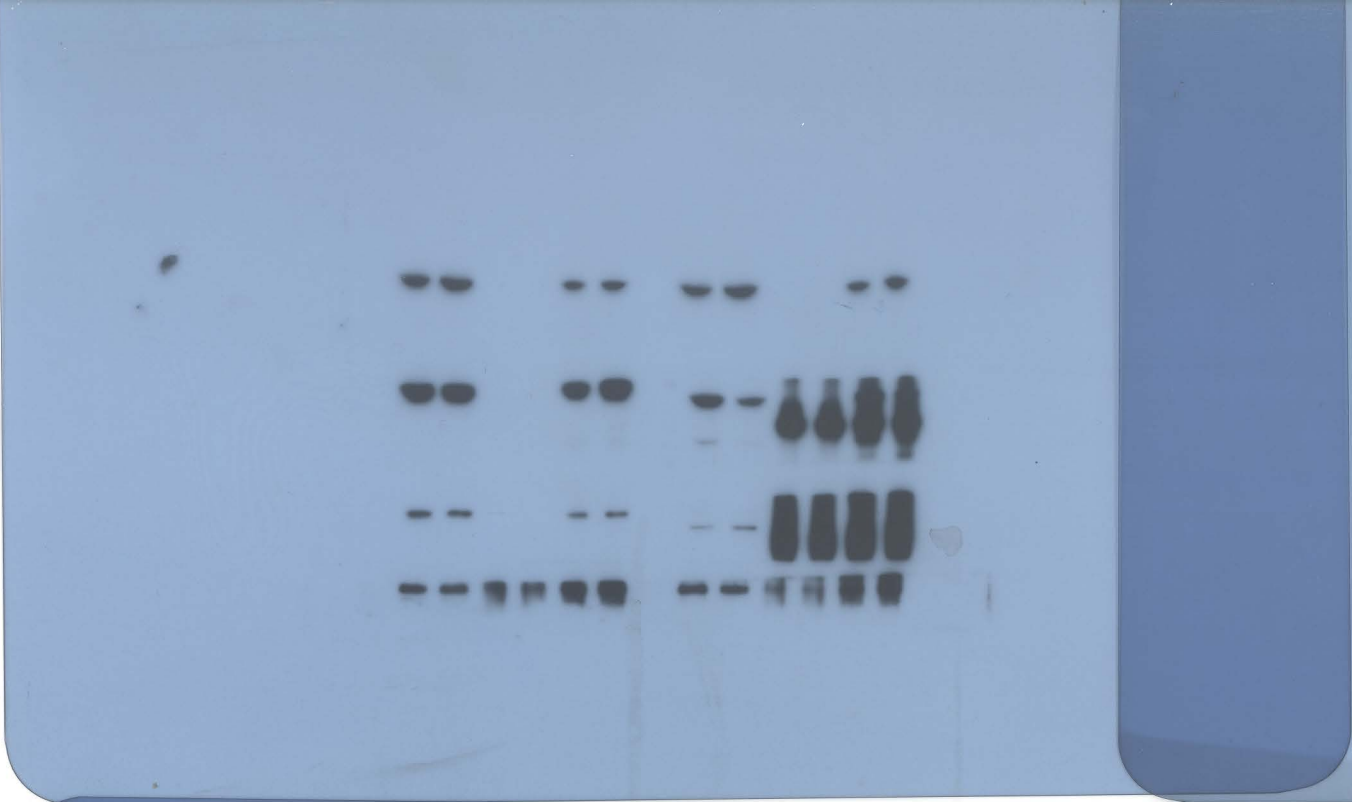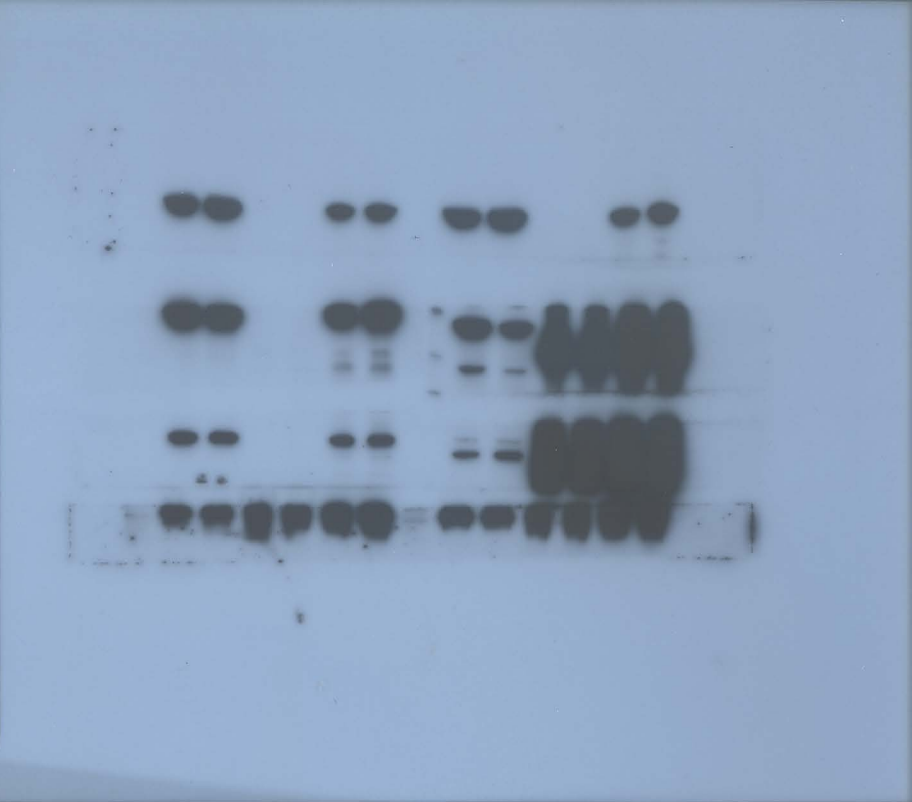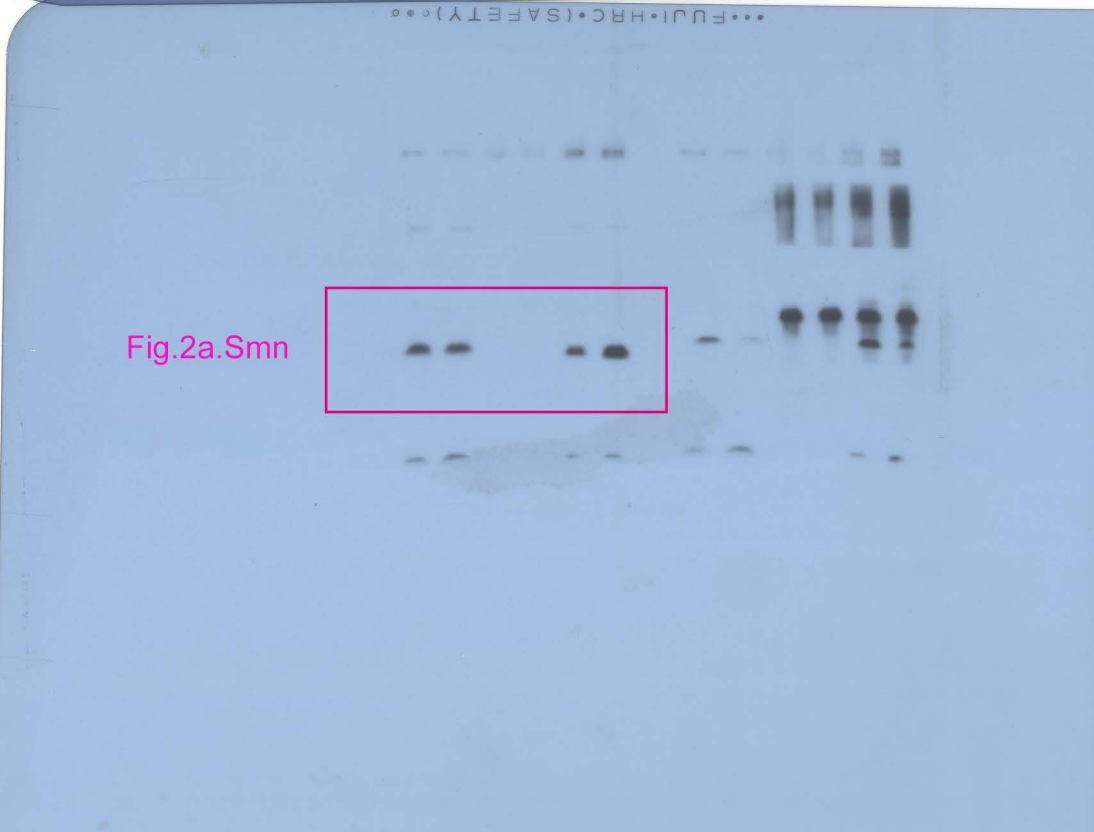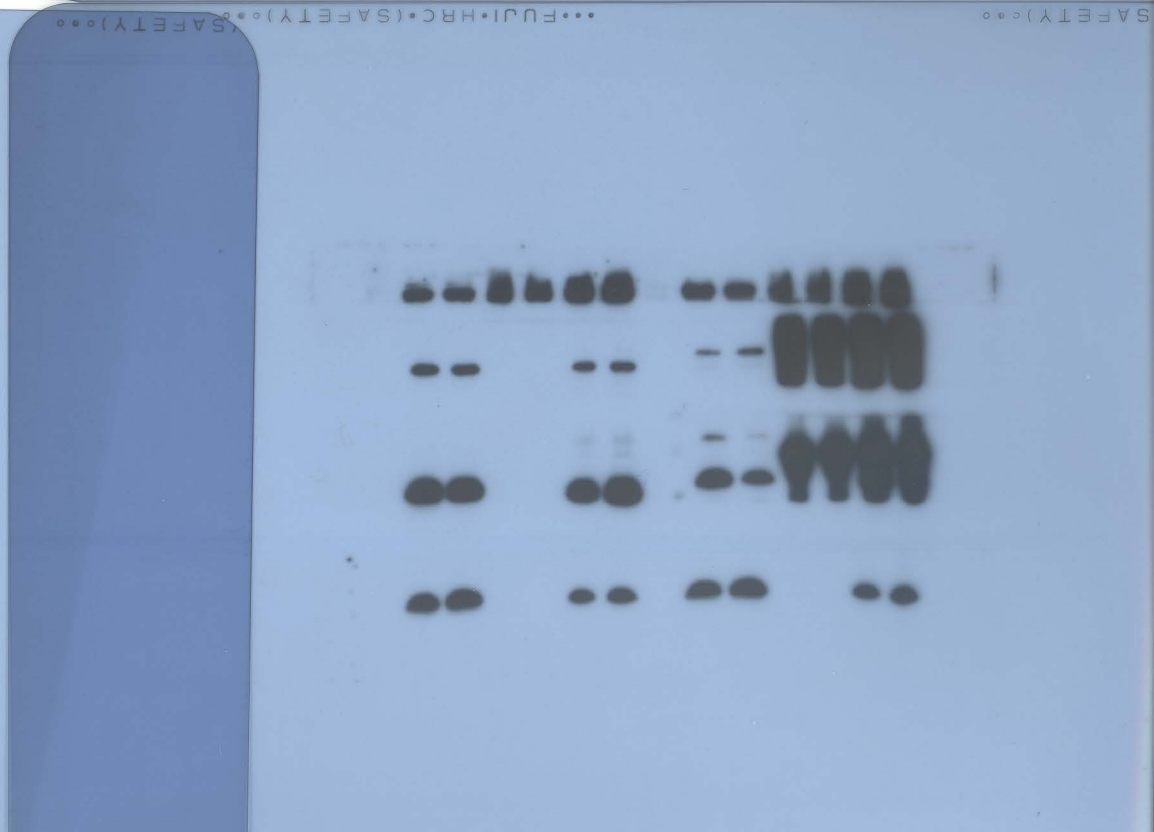

Fig.2a.Smn

Supplement: Supplementary file 8 — Source Data [file 41467_2021_21529_MOESM8_ESM.zip › Uncropped blot and gel images/Figure2/Figure2a/Smn.pdf]

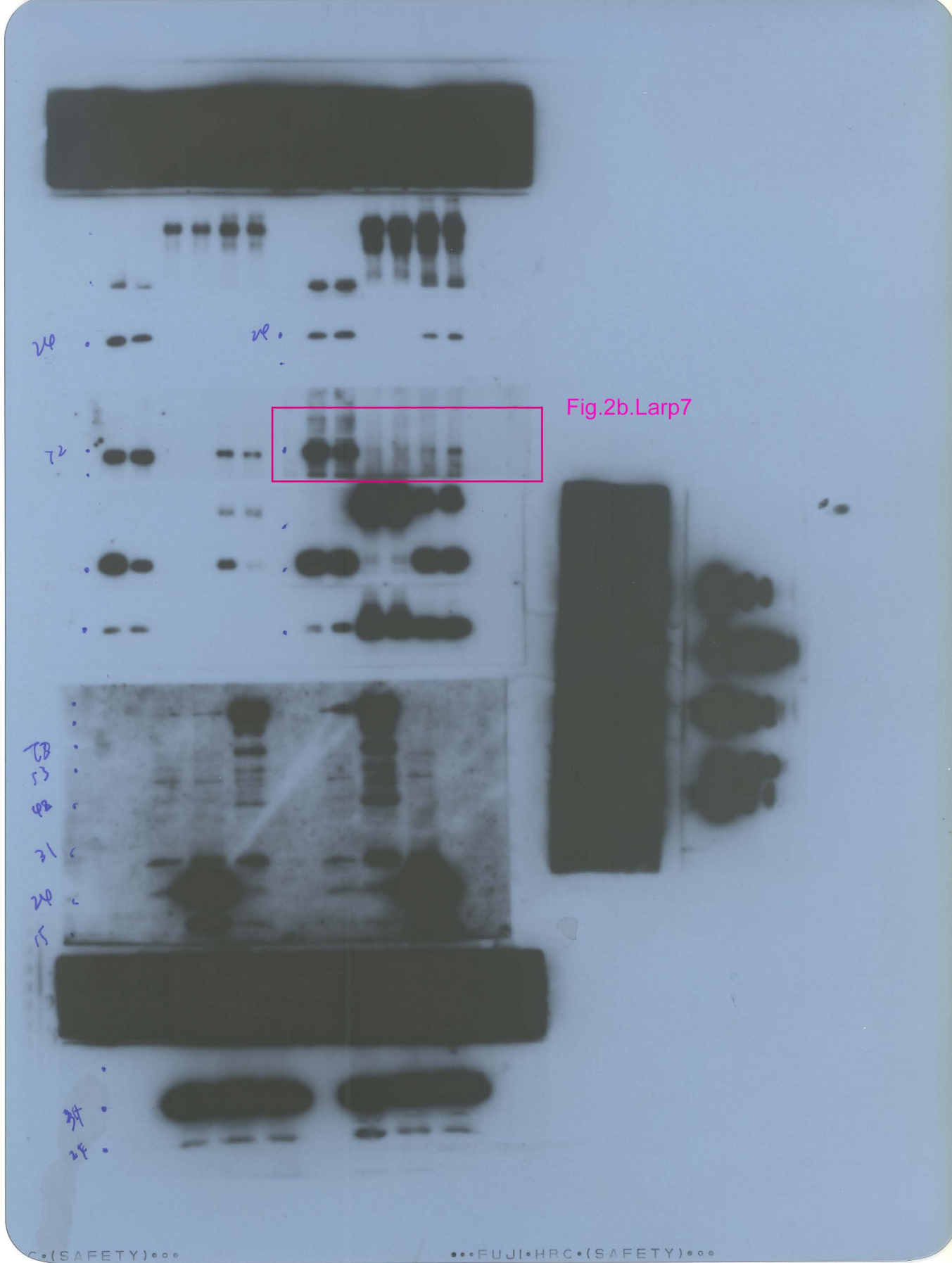

Supplement: Supplementary file 8 — Source Data [file 41467_2021_21529_MOESM8_ESM.zip › Uncropped blot and gel images/Figure2/Figure2b/Larp7.pdf]

Fig.2b.Smn

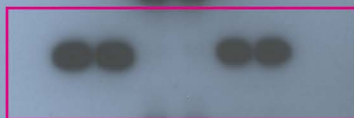

Supplement: Supplementary file 8 — Source Data [file 41467_2021_21529_MOESM8_ESM.zip › Uncropped blot and gel images/Figure2/Figure2b/Smn.pdf]

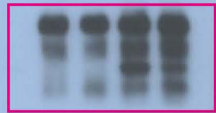

Fig.2c.Cdk9

Supplement: Supplementary file 8 — Source Data [file 41467_2021_21529_MOESM8_ESM.zip › Uncropped blot and gel images/Figure2/Figure2c/Cdk9_1.pdf]

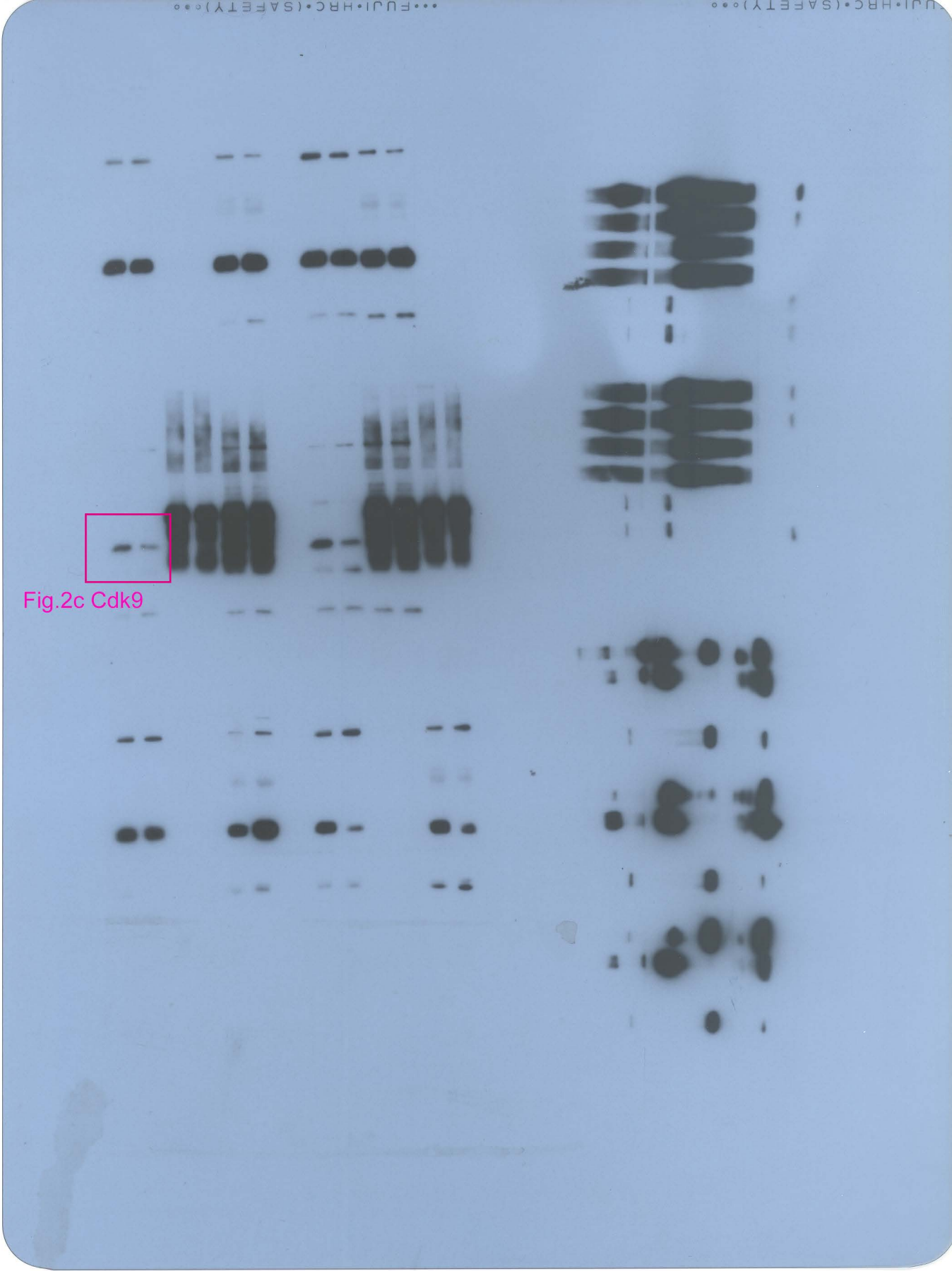

Fig.2c Cdk9

Supplement: Supplementary file 8 — Source Data [file 41467_2021_21529_MOESM8_ESM.zip › Uncropped blot and gel images/Figure2/Figure2c/Cdk9_2.pdf]

Fig.2c.hnRNP R

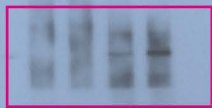

Supplement: Supplementary file 8 — Source Data [file 41467_2021_21529_MOESM8_ESM.zip › Uncropped blot and gel images/Figure2/Figure2c/hnRNP R.pdf]

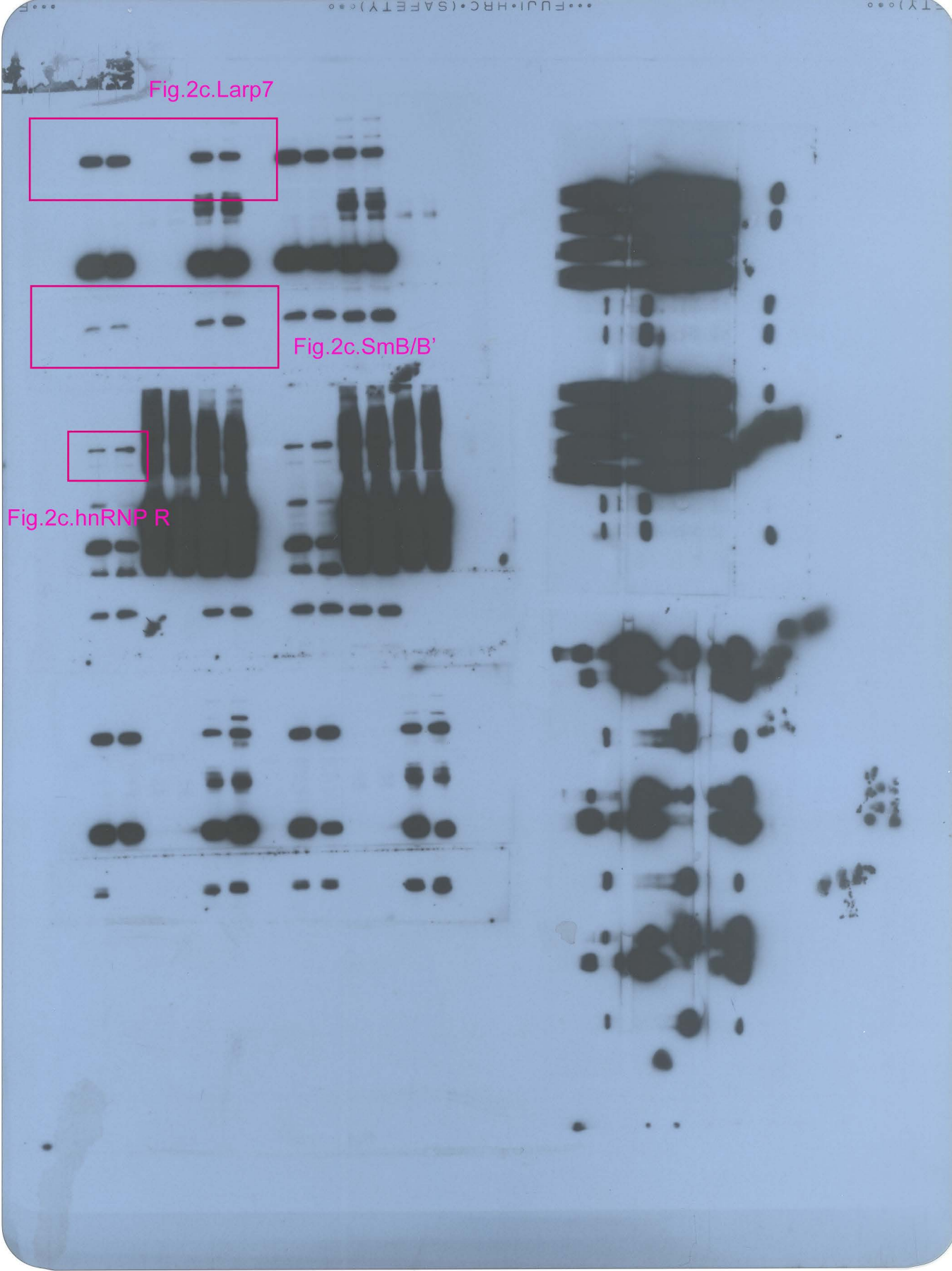

Fig.2c.Larp7

Fig.2c.SmB/B'

Fig.2c.hnRNP R

Supplement: Supplementary file 8 — Source Data [file 41467_2021_21529_MOESM8_ESM.zip › Uncropped blot and gel images/Figure2/Figure2c/Larp7_SmB_hnRNP R.pdf]

Fig.2c.Smn

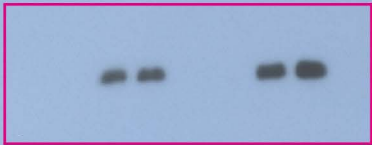

Supplement: Supplementary file 8 — Source Data [file 41467_2021_21529_MOESM8_ESM.zip › Uncropped blot and gel images/Figure2/Figure2c/Smn.pdf]

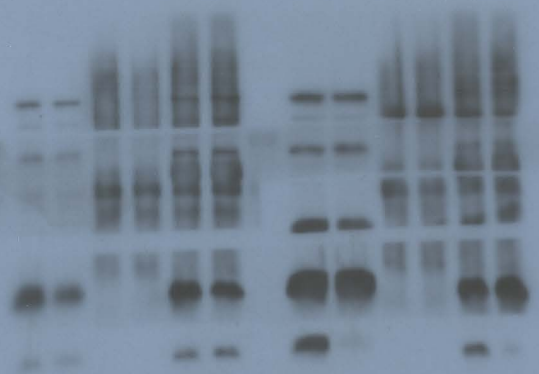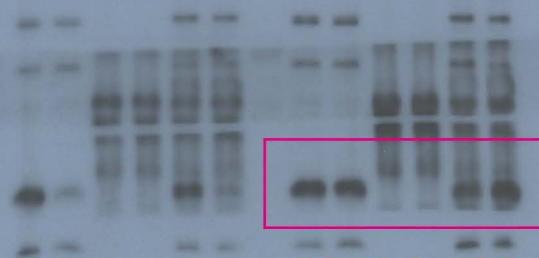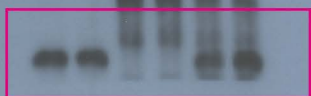

Fig.2d.Gemin2

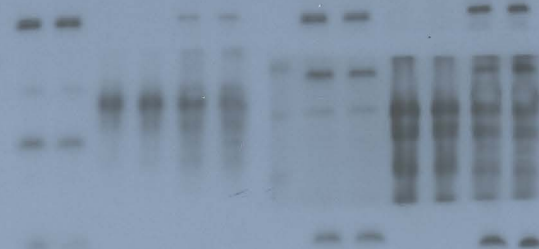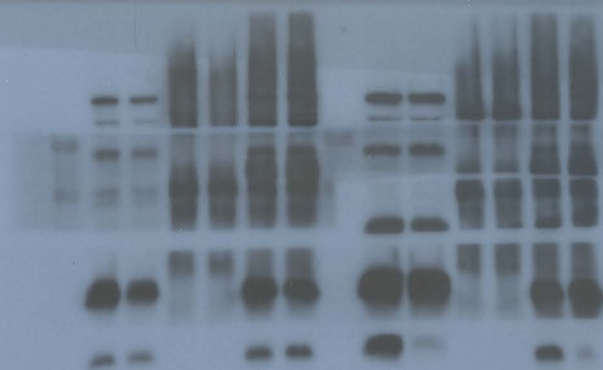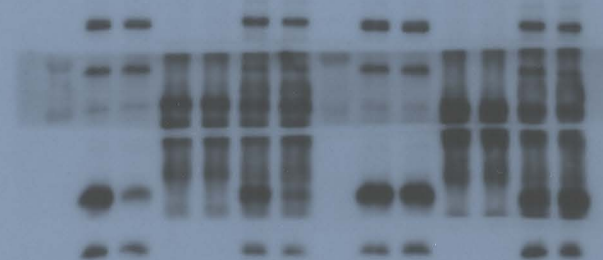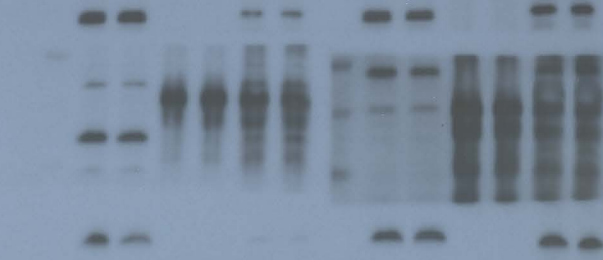

Supplement: Supplementary file 8 — Source Data [file 41467_2021_21529_MOESM8_ESM.zip › Uncropped blot and gel images/Figure2/Figure2d/Gemin2.pdf]

Fig.2d.Larp7

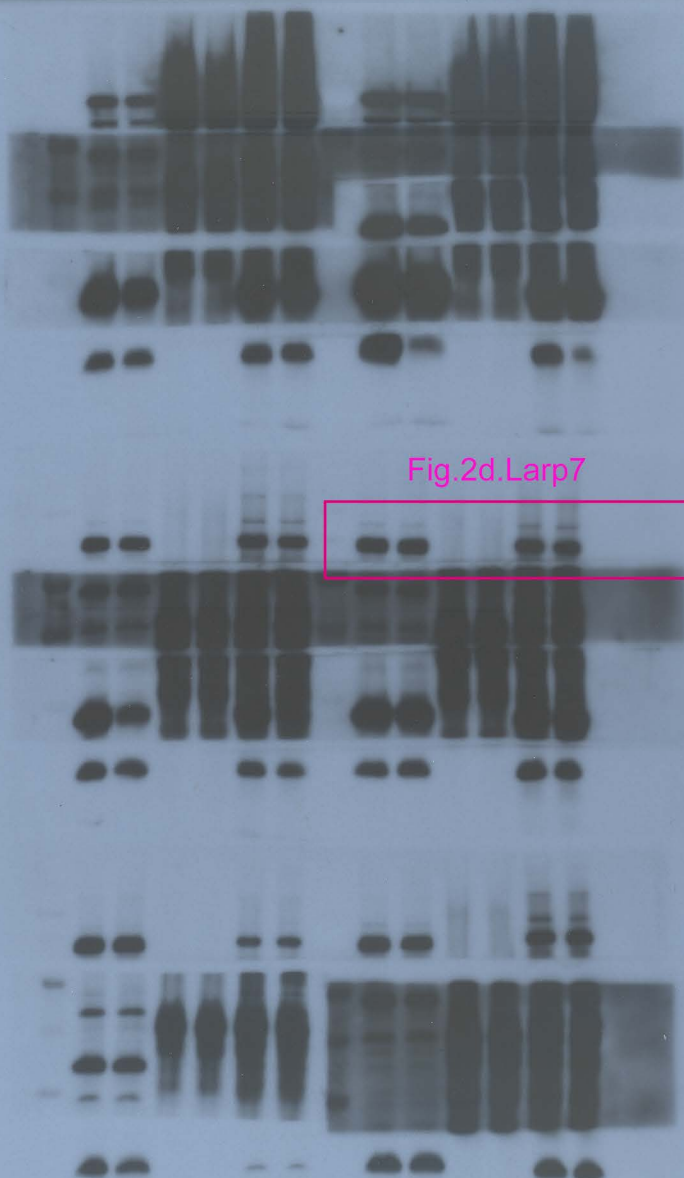

Supplement: Supplementary file 8 — Source Data [file 41467_2021_21529_MOESM8_ESM.zip › Uncropped blot and gel images/Figure2/Figure2d/Larp7.pdf]

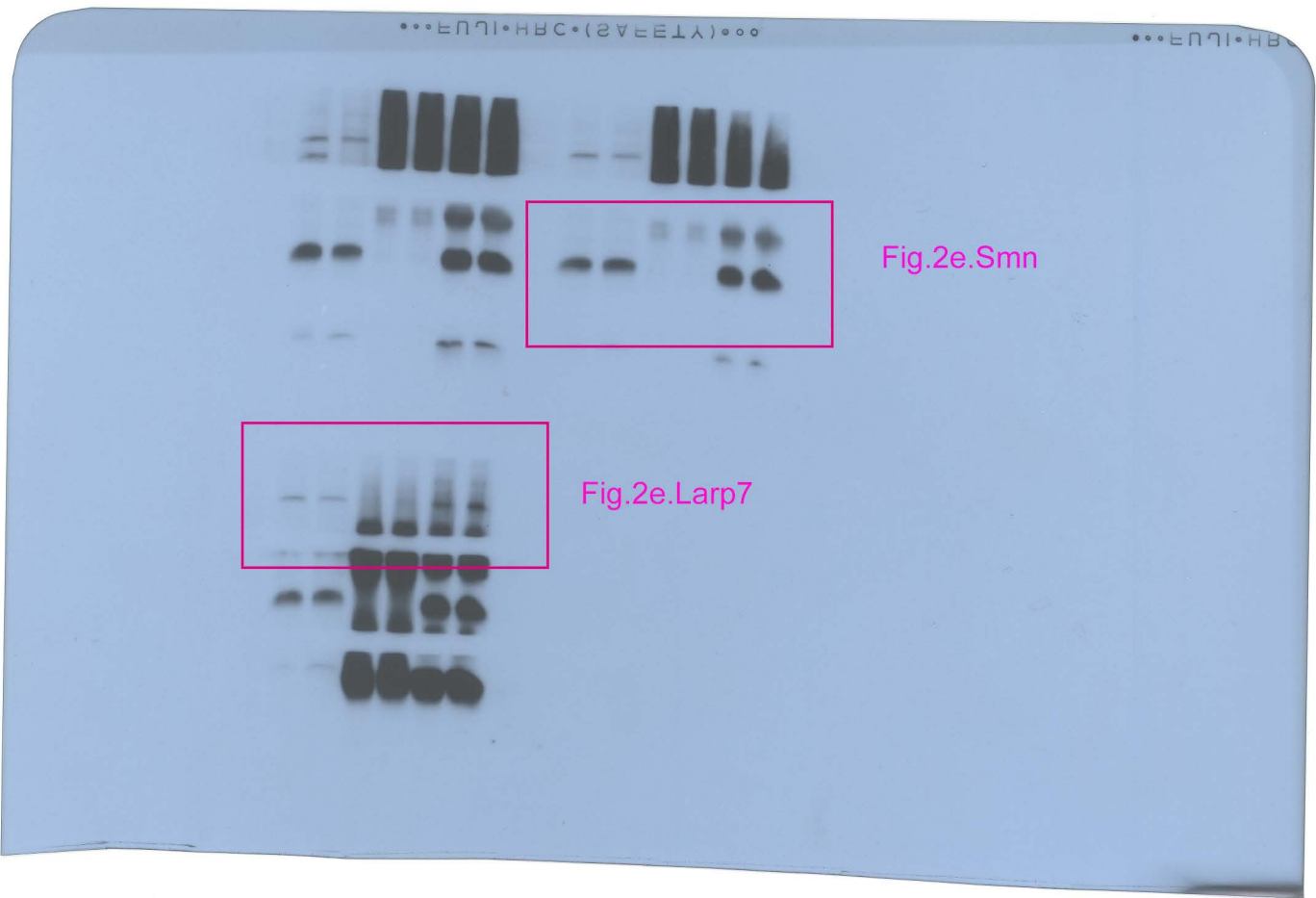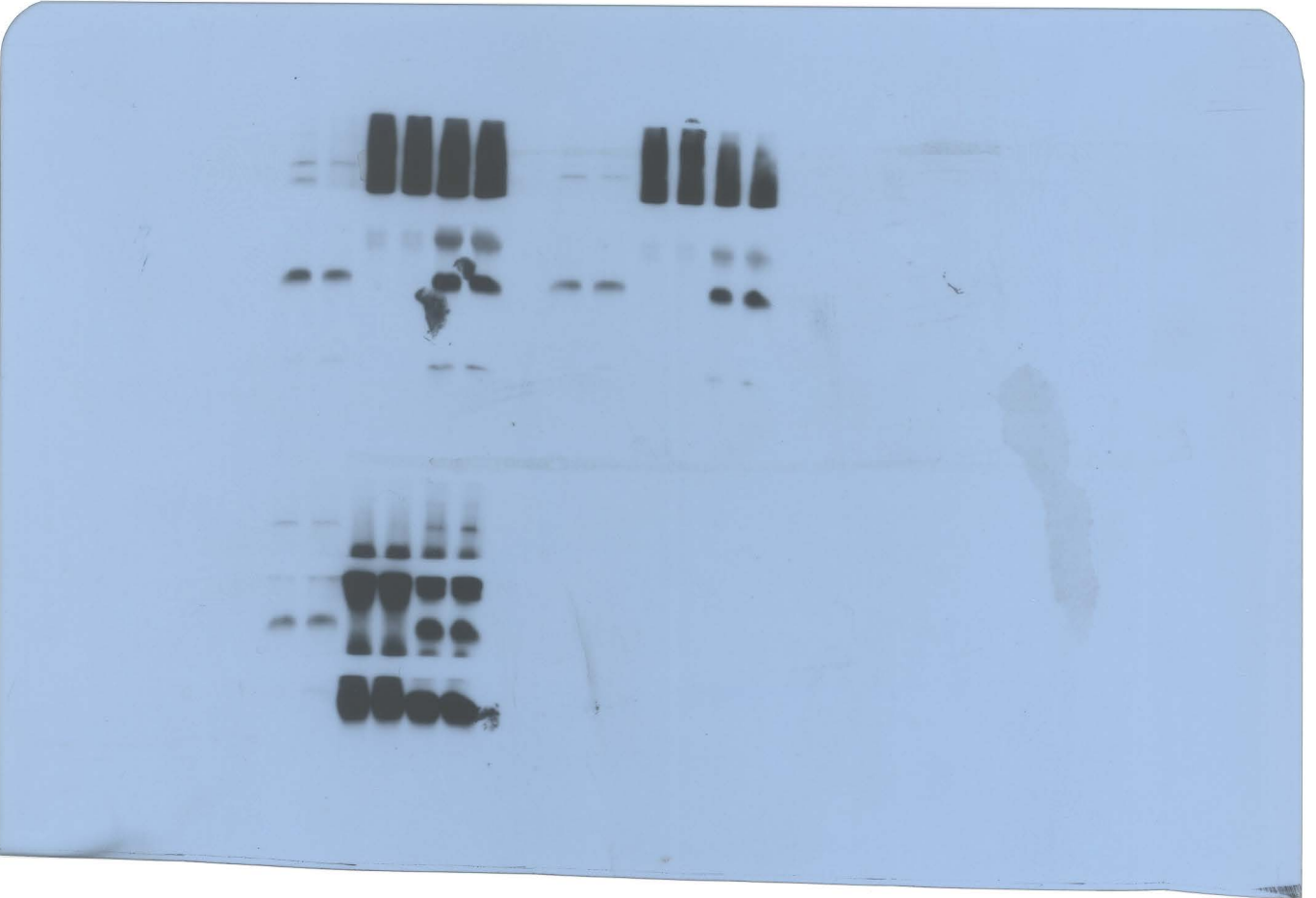

Supplement: Supplementary file 8 — Source Data [file 41467_2021_21529_MOESM8_ESM.zip › Uncropped blot and gel images/Figure2/Figure2e/Smn_Larp7.pdf]

Fig.2f.GEMIN2

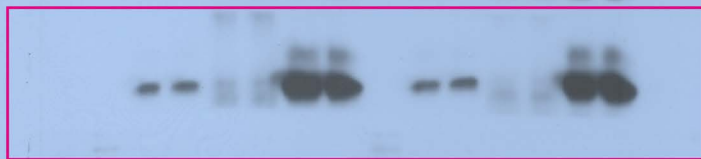

Supplement: Supplementary file 8 — Source Data [file 41467_2021_21529_MOESM8_ESM.zip › Uncropped blot and gel images/Figure2/Figure2f/GEMIN2.pdf]

Fig.2f.LARP7

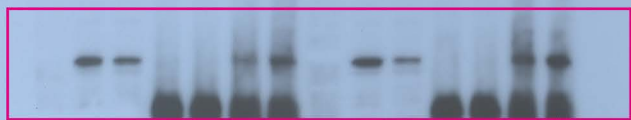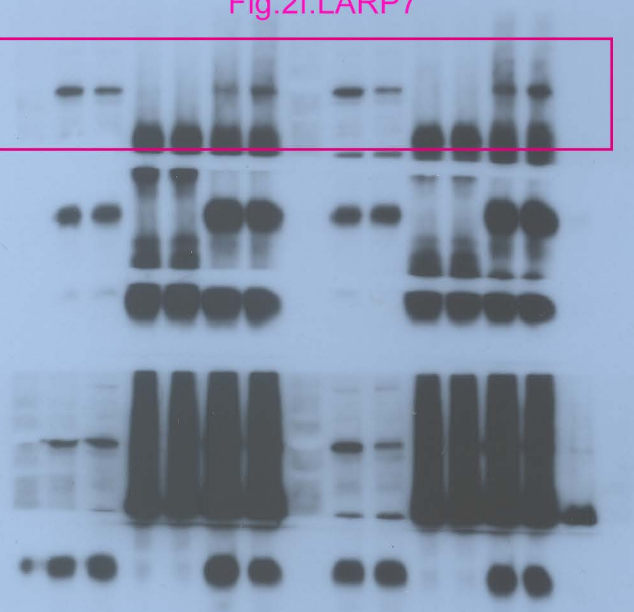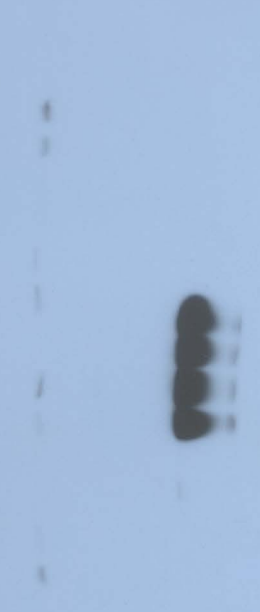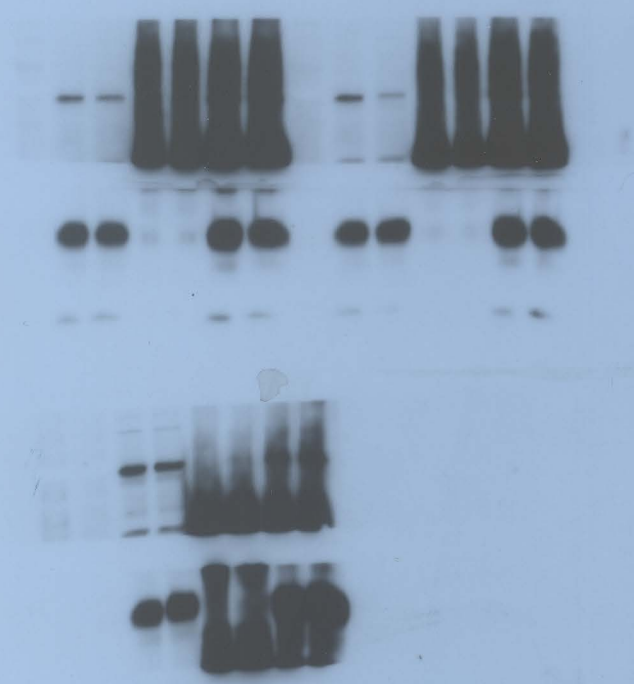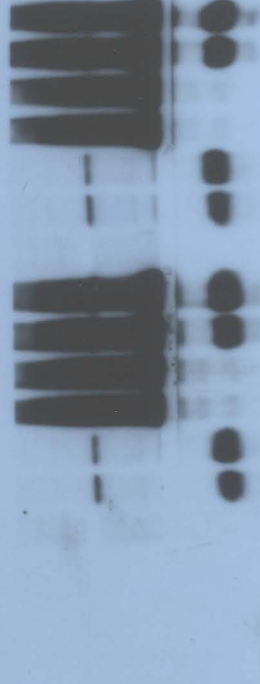

Supplement: Supplementary file 8 — Source Data [file 41467_2021_21529_MOESM8_ESM.zip › Uncropped blot and gel images/Figure2/Figure2f/LARP7.pdf]

Fig.2f.MePCE

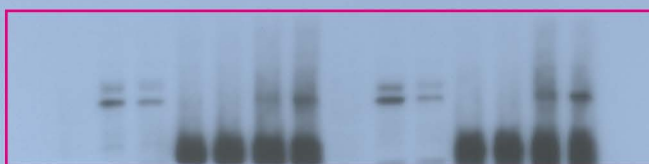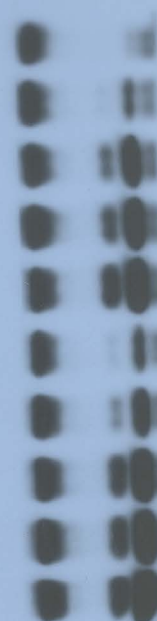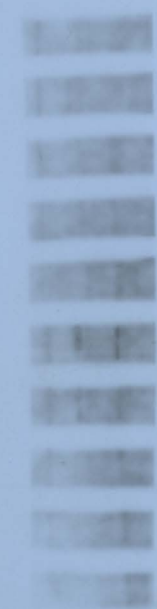

Supplement: Supplementary file 8 — Source Data [file 41467_2021_21529_MOESM8_ESM.zip › Uncropped blot and gel images/Figure2/Figure2f/MePCE.pdf]

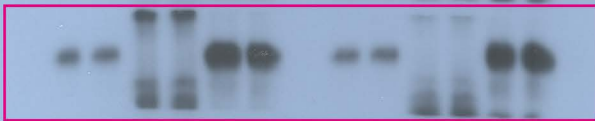

Fig.2f.SMN

Supplement: Supplementary file 8 — Source Data [file 41467_2021_21529_MOESM8_ESM.zip › Uncropped blot and gel images/Figure2/Figure2f/SMN.pdf]

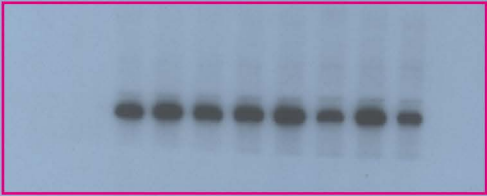

Fig.3a.Larp7

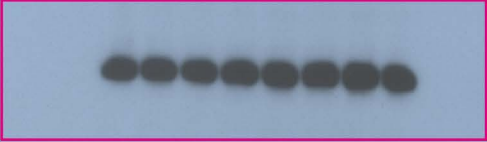

Fig.3a.Smn

Supplement: Supplementary file 8 — Source Data [file 41467_2021_21529_MOESM8_ESM.zip › Uncropped blot and gel images/Figure3/Figure3a/Larp7_Smn.pdf]

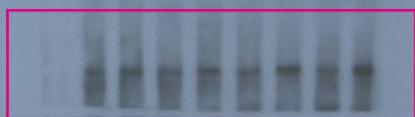

Fig.3c.Larp7

Supplement: Supplementary file 8 — Source Data [file 41467_2021_21529_MOESM8_ESM.zip › Uncropped blot and gel images/Figure3/Figure3c/Larp7.pdf]

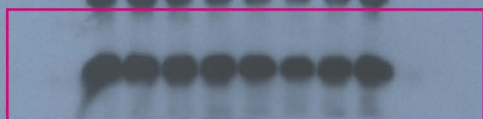

Fig.3c.Smn

Supplement: Supplementary file 8 — Source Data [file 41467_2021_21529_MOESM8_ESM.zip › Uncropped blot and gel images/Figure3/Figure3c/Smn.pdf]

Fig.3e.Mepce

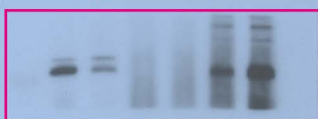

Fig.3e.Smn

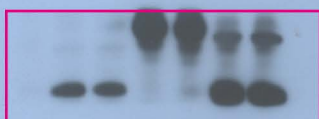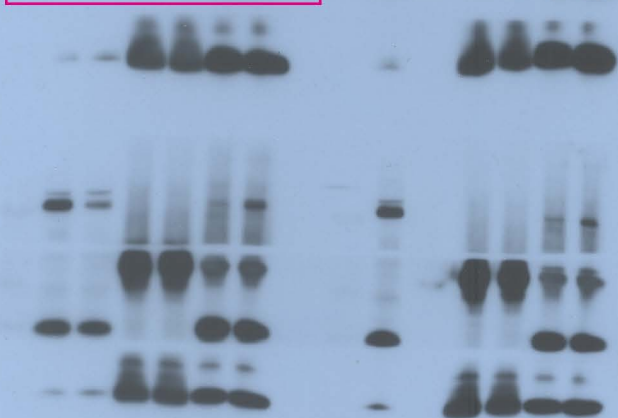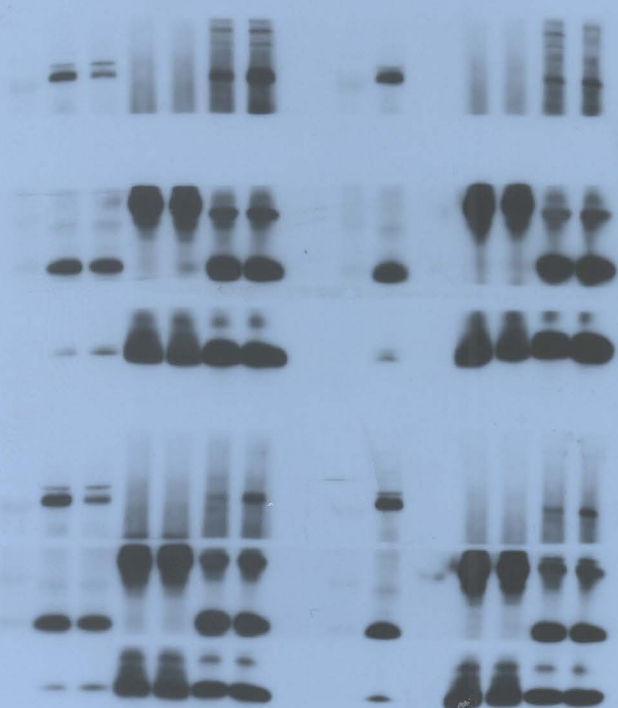

Supplement: Supplementary file 8 — Source Data [file 41467_2021_21529_MOESM8_ESM.zip › Uncropped blot and gel images/Figure3/Figure3e/Mepce_Smn.pdf]

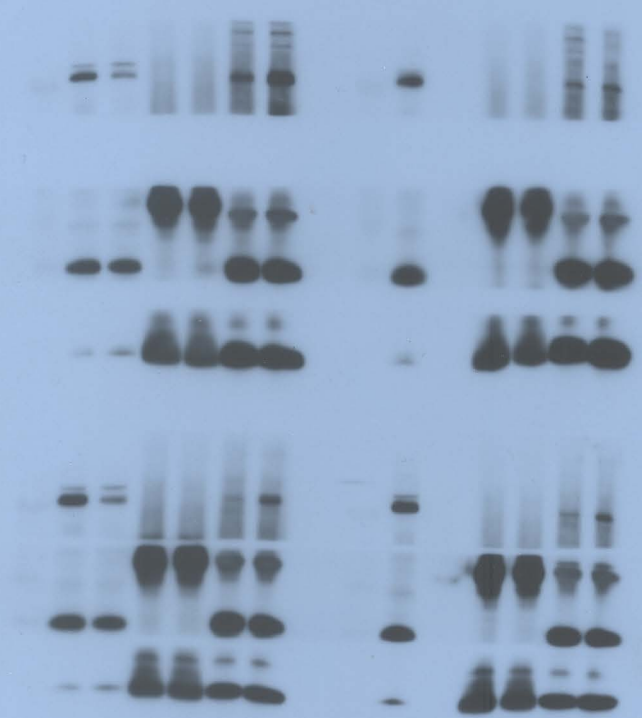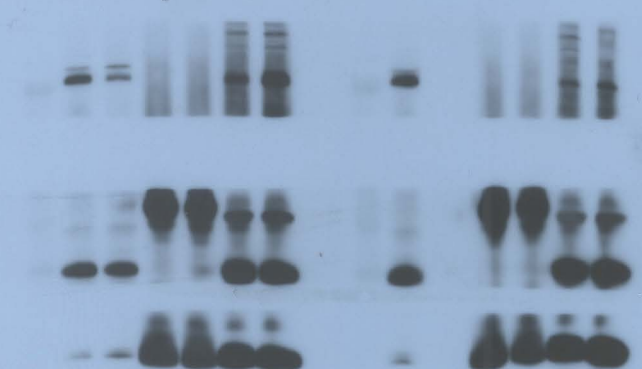

Fig.3f.Mepce

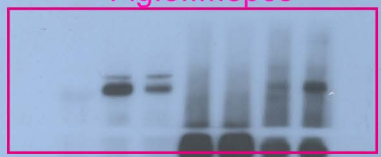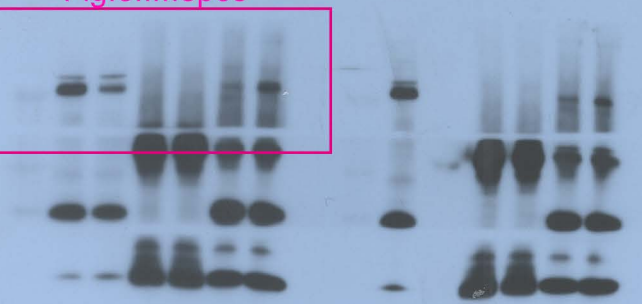

Supplement: Supplementary file 8 — Source Data [file 41467_2021_21529_MOESM8_ESM.zip › Uncropped blot and gel images/Figure3/Figure3f/Mepce.pdf]

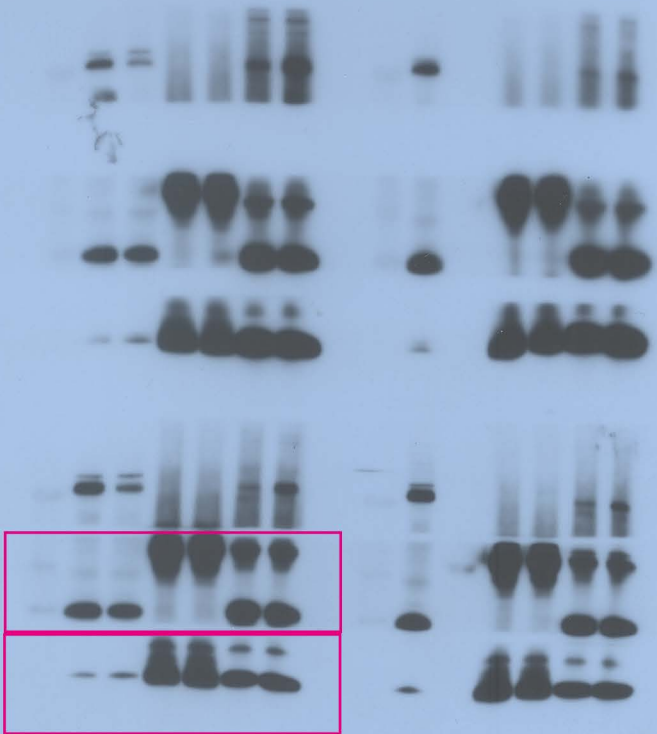

Fig.3f.Smn

Fig.3f.SmB/B'

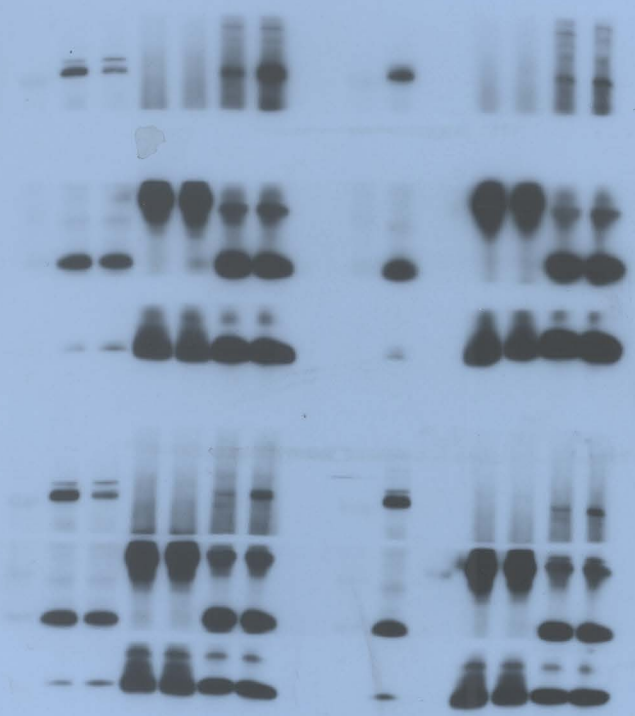

Supplement: Supplementary file 8 — Source Data [file 41467_2021_21529_MOESM8_ESM.zip › Uncropped blot and gel images/Figure3/Figure3f/Smn_SmB.pdf]

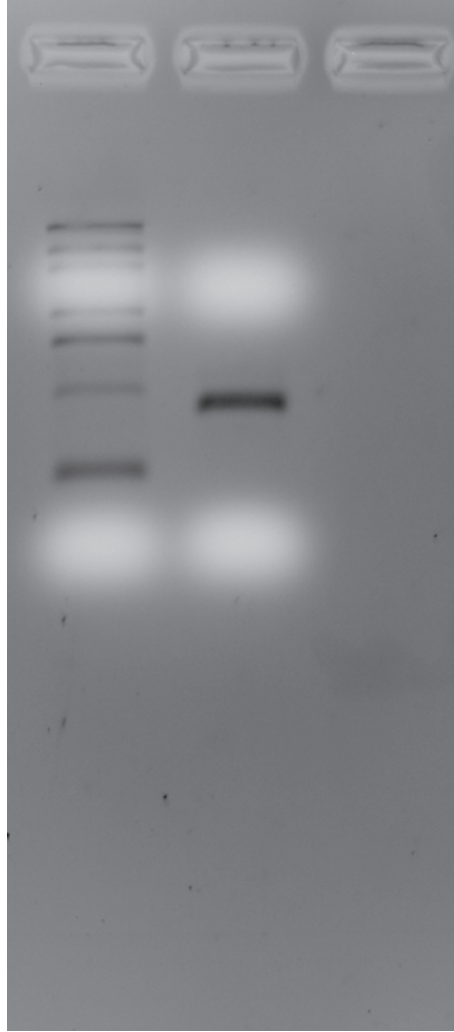

Supplement: Supplementary file 8 — Source Data [file 41467_2021_21529_MOESM8_ESM.zip › Uncropped blot and gel images/Figure3/Figure3i/Biotinylated_U2 snRNA.pdf]

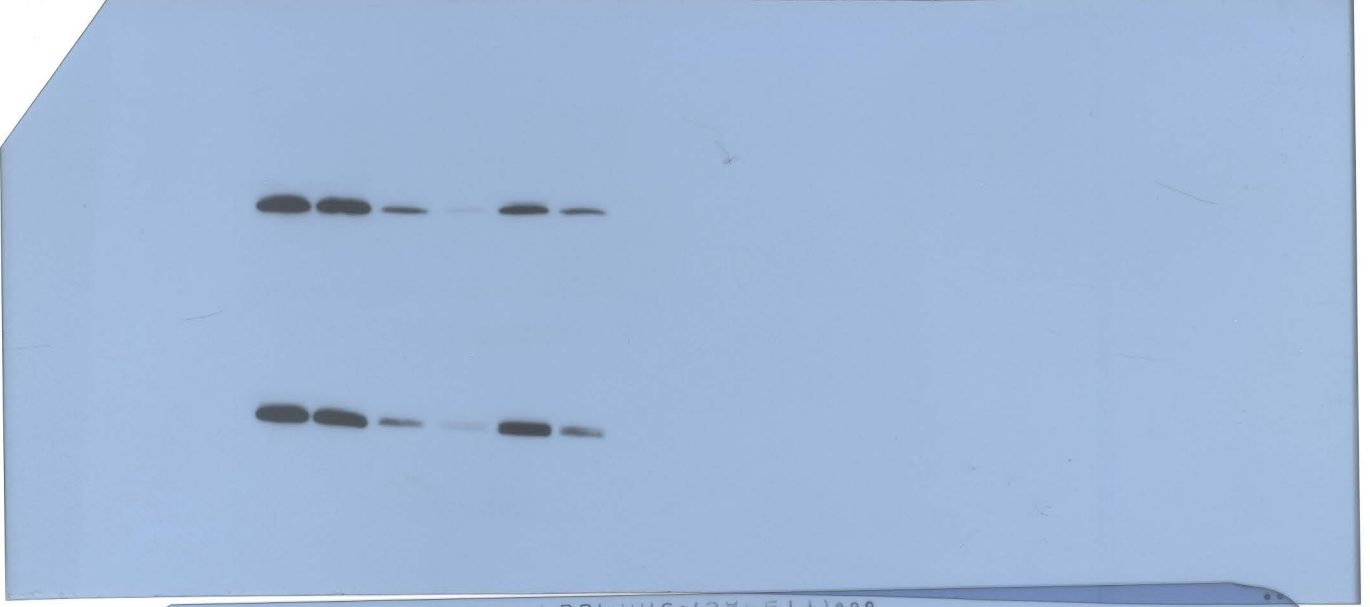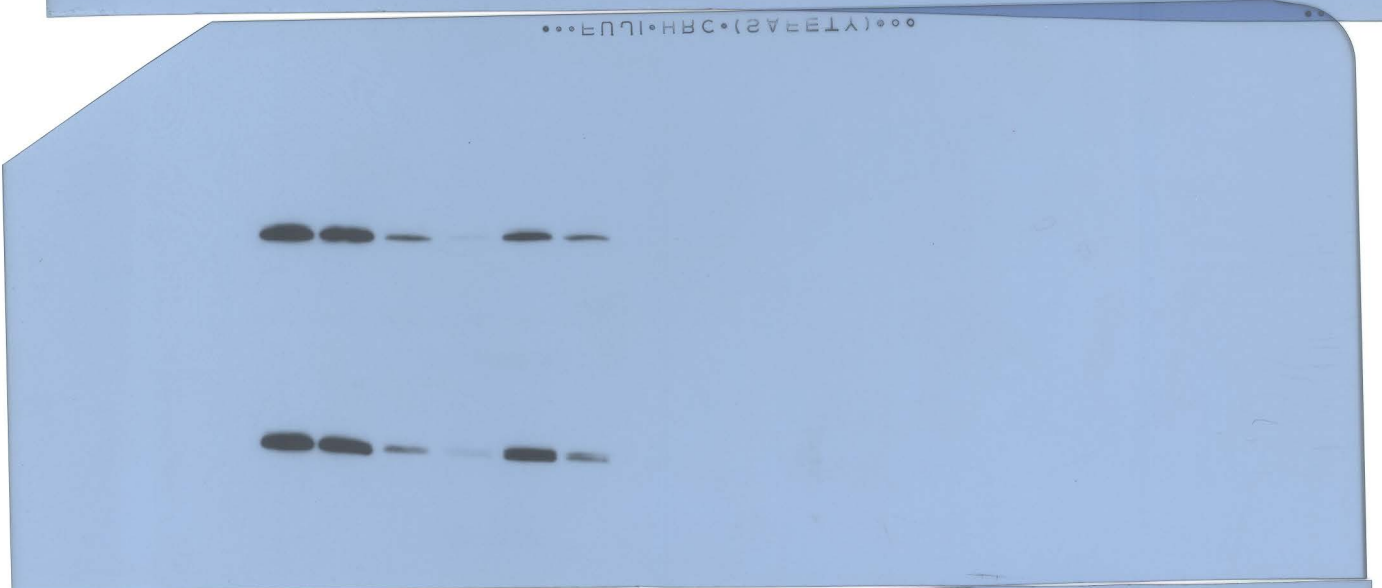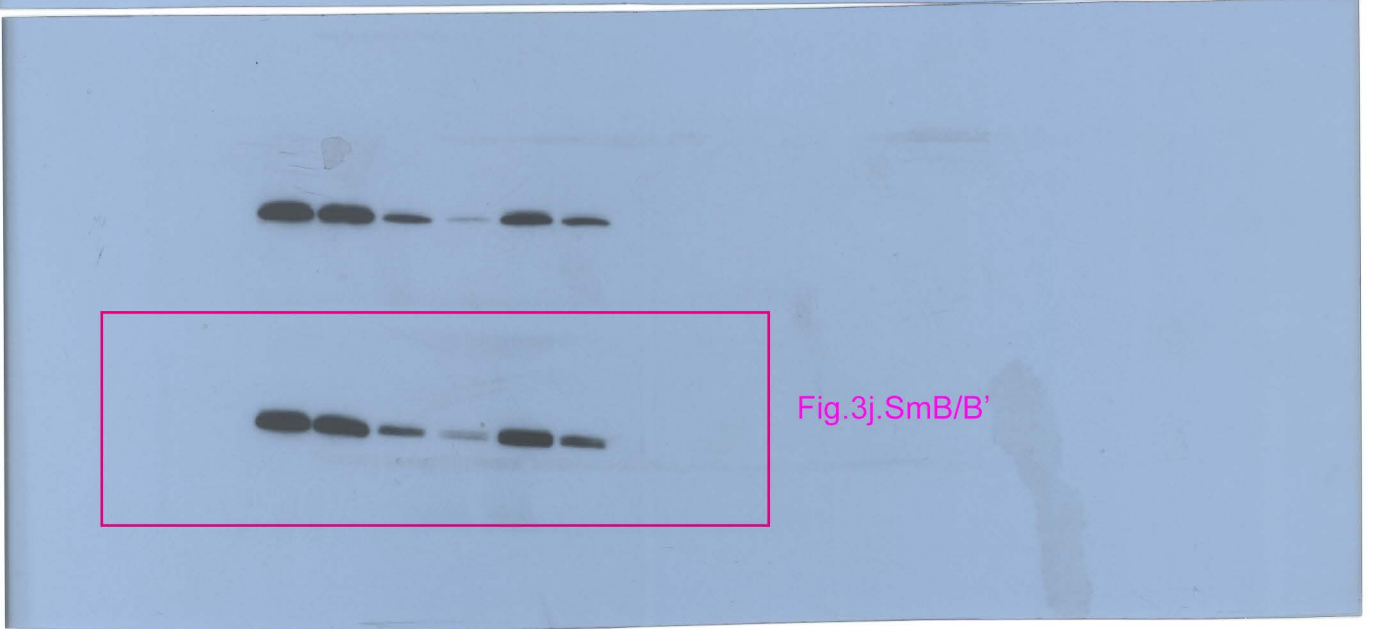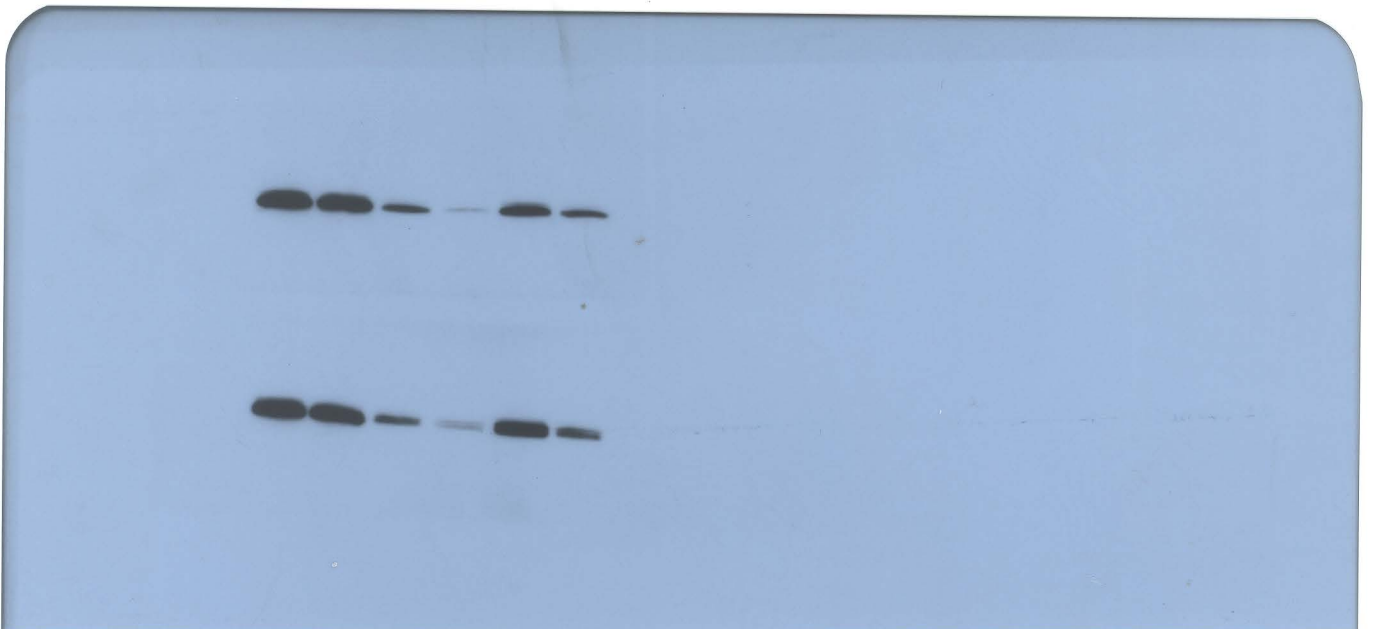

Supplement: Supplementary file 8 — Source Data [file 41467_2021_21529_MOESM8_ESM.zip › Uncropped blot and gel images/Figure3/Figure3j/SmB.pdf]

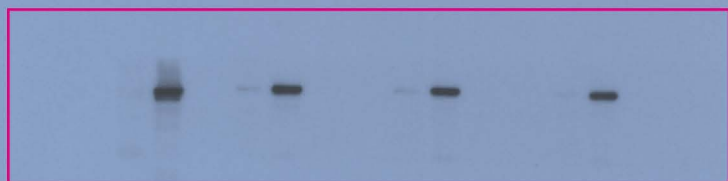

Fig.4a. Calnexin

Supplement: Supplementary file 8 — Source Data [file 41467_2021_21529_MOESM8_ESM.zip › Uncropped blot and gel images/Figure4/Figure4a/Calnexin.pdf]

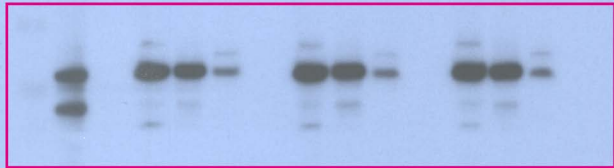

Fig.4a.Hexim1

Supplement: Supplementary file 8 — Source Data [file 41467_2021_21529_MOESM8_ESM.zip › Uncropped blot and gel images/Figure4/Figure4a/Hexim1.pdf]

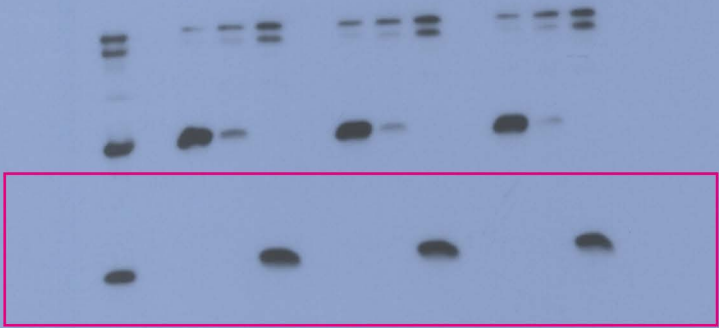

Fig.4a.Histone H3

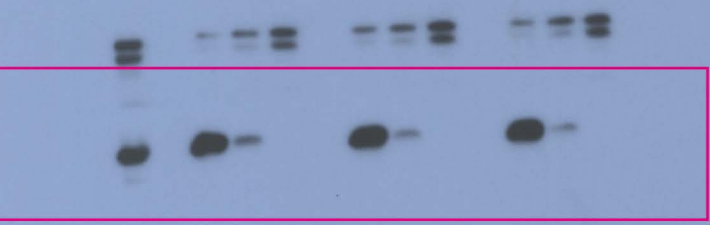

Fig.4a.Gapdh

Supplement: Supplementary file 8 — Source Data [file 41467_2021_21529_MOESM8_ESM.zip › Uncropped blot and gel images/Figure4/Figure4a/Histone H3_Gapdh.pdf]

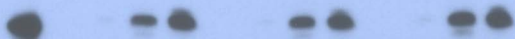

Fig.4a.hnRNP A1

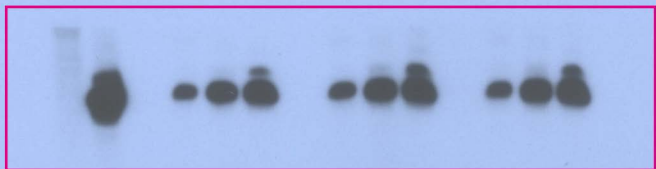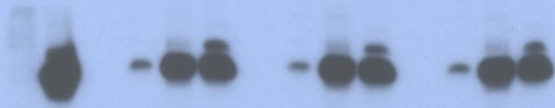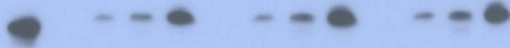

Supplement: Supplementary file 8 — Source Data [file 41467_2021_21529_MOESM8_ESM.zip › Uncropped blot and gel images/Figure4/Figure4a/hnRNP A1.pdf]

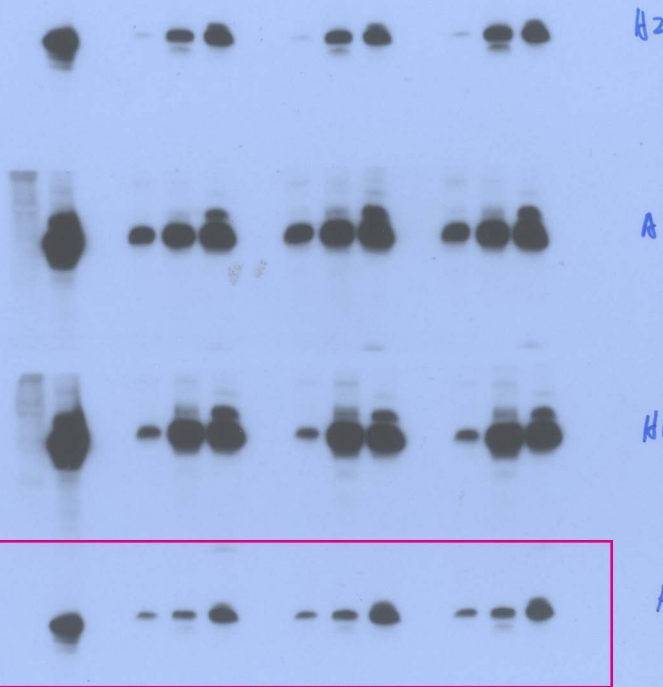

Fig.4a.hnRNP A2/B1

Supplement: Supplementary file 8 — Source Data [file 41467_2021_21529_MOESM8_ESM.zip › Uncropped blot and gel images/Figure4/Figure4a/hnRNP A2B1.pdf]

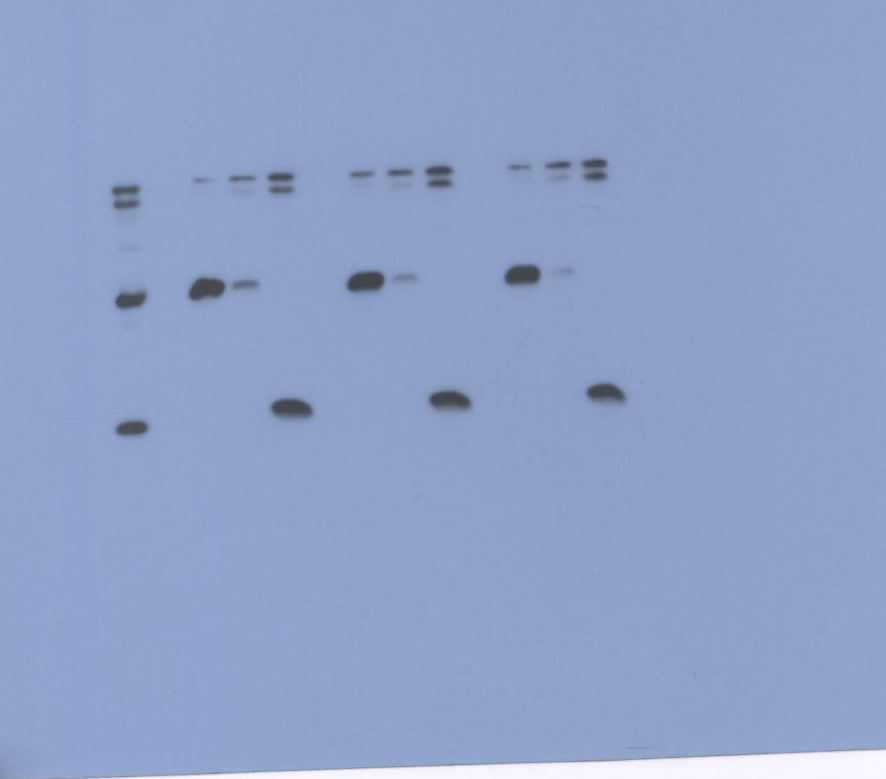

•••(SAFETY)•••

Fig.4a.hnRNP R

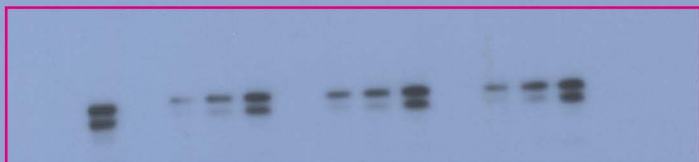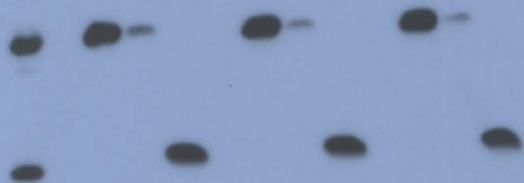

Supplement: Supplementary file 8 — Source Data [file 41467_2021_21529_MOESM8_ESM.zip › Uncropped blot and gel images/Figure4/Figure4a/hnRNP R.pdf]

Fig.4a Larp7

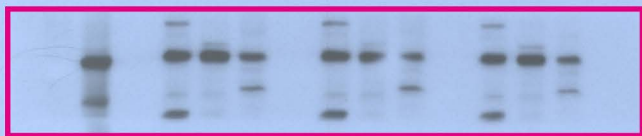

Supplement: Supplementary file 8 — Source Data [file 41467_2021_21529_MOESM8_ESM.zip › Uncropped blot and gel images/Figure4/Figure4a/Larp7.pdf]

...FUJI.HRC.(SA  
I.HRC.(SAFETY)...

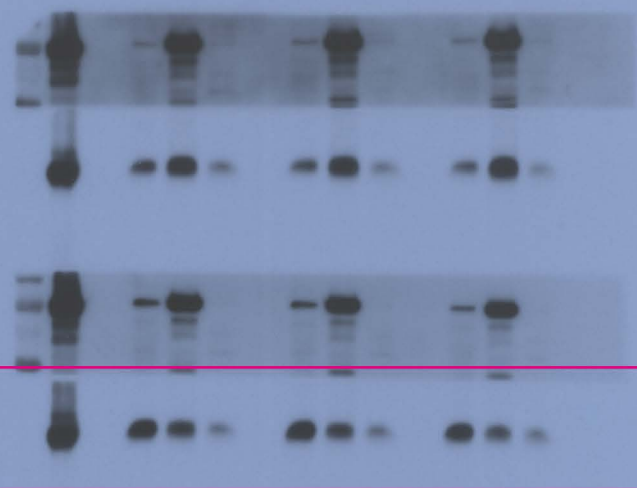

Fig.4a.Smn

Supplement: Supplementary file 8 — Source Data [file 41467_2021_21529_MOESM8_ESM.zip › Uncropped blot and gel images/Figure4/Figure4a/Smn.pdf]

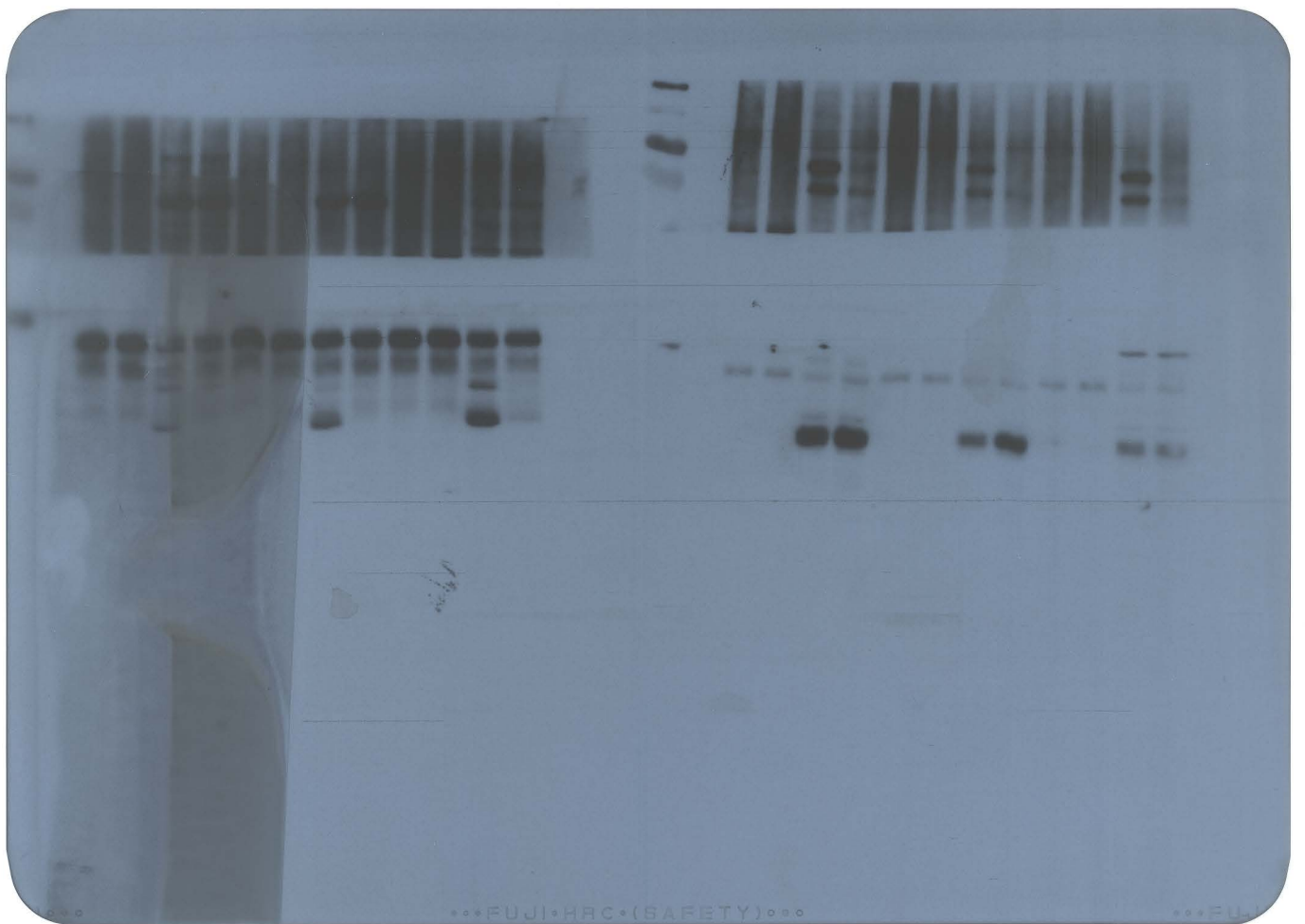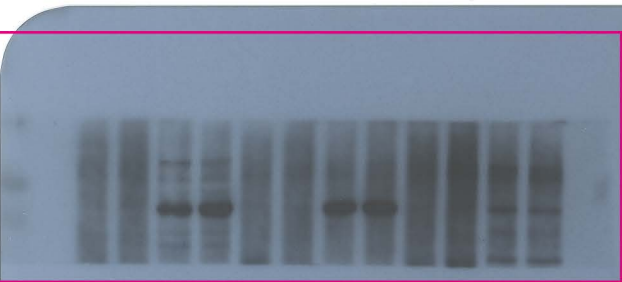

Fig.4d.Larp7

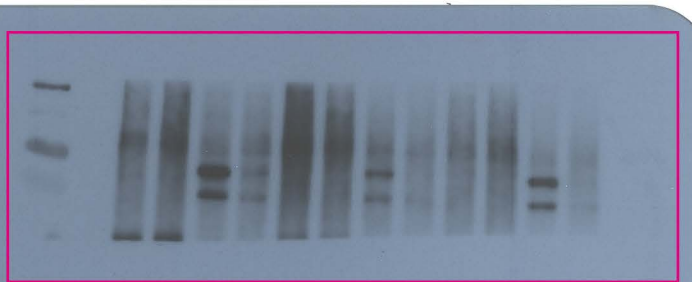

Fig.4d.hnRNP R

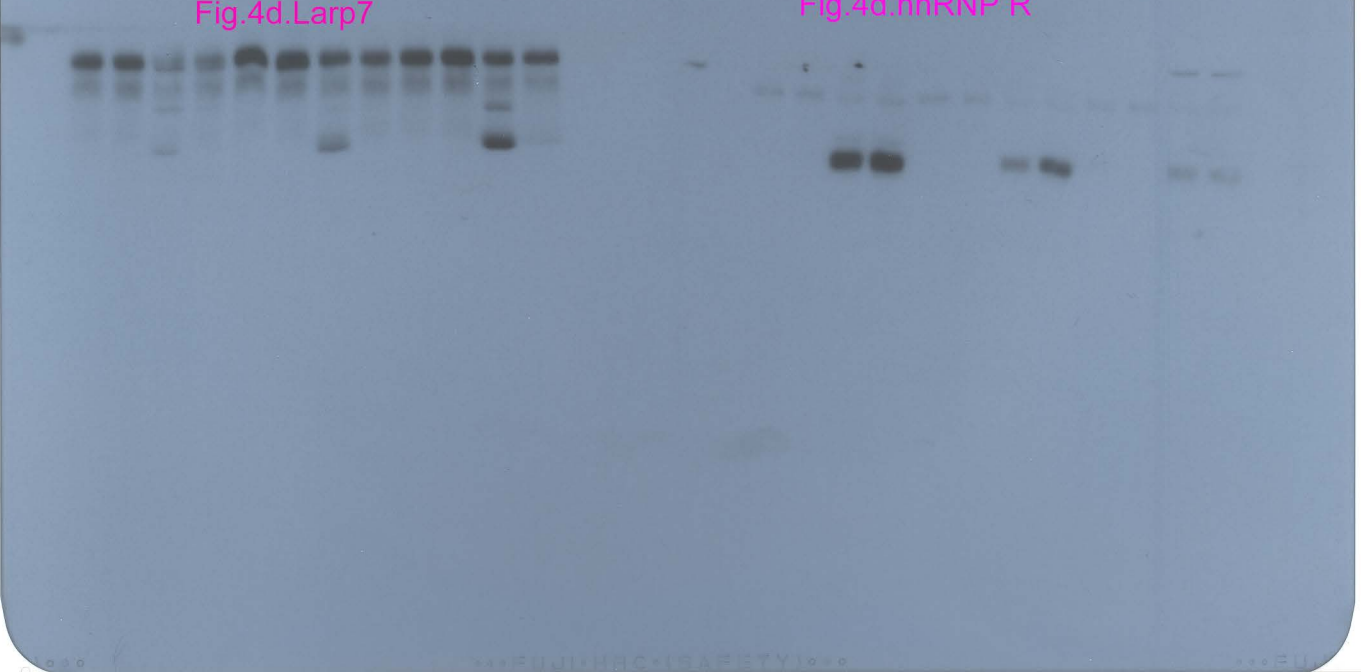

Supplement: Supplementary file 8 — Source Data [file 41467_2021_21529_MOESM8_ESM.zip › Uncropped blot and gel images/Figure4/Figure4d/Larp7_hnRNP R.pdf]

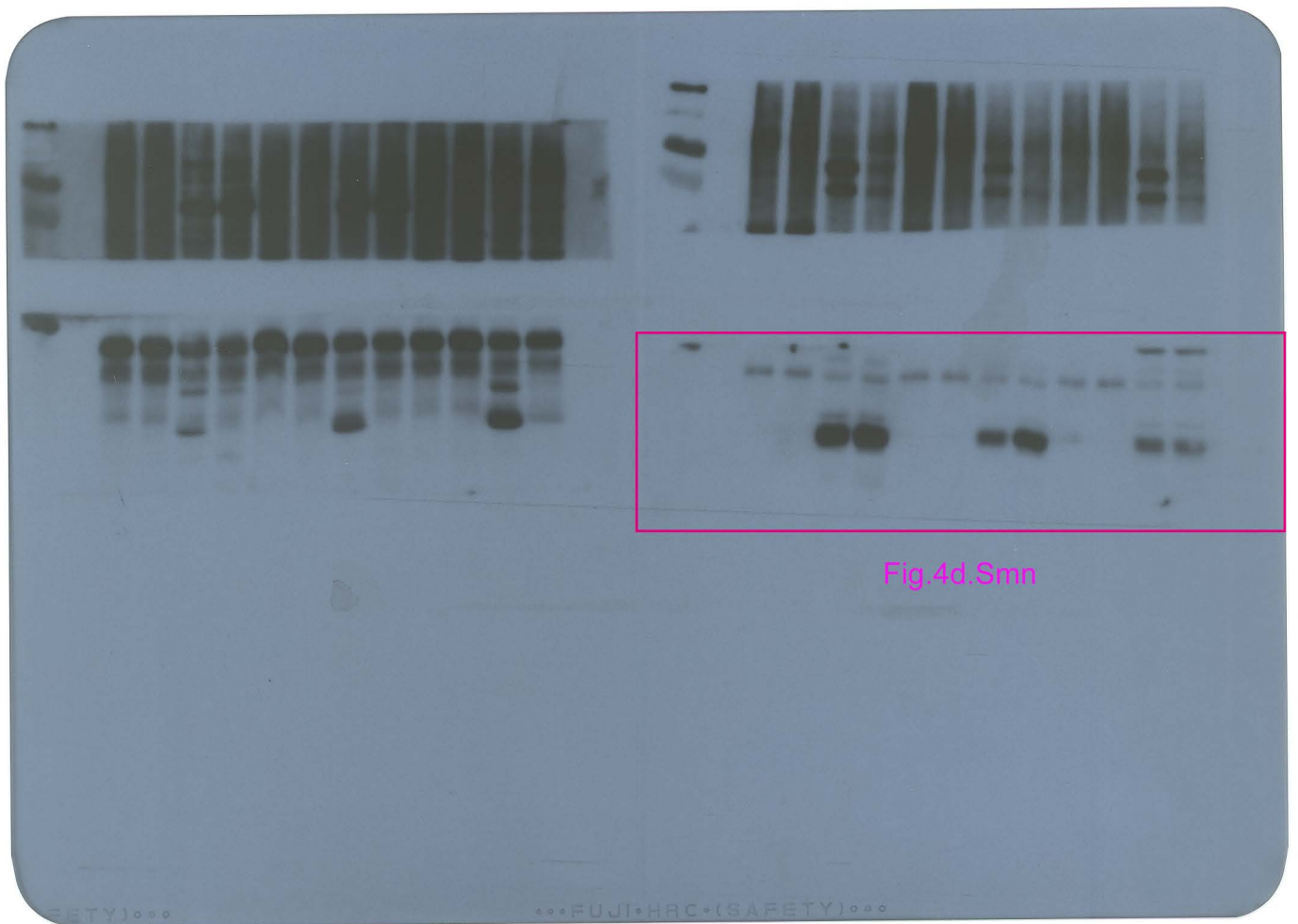

Fig.4d.Smn

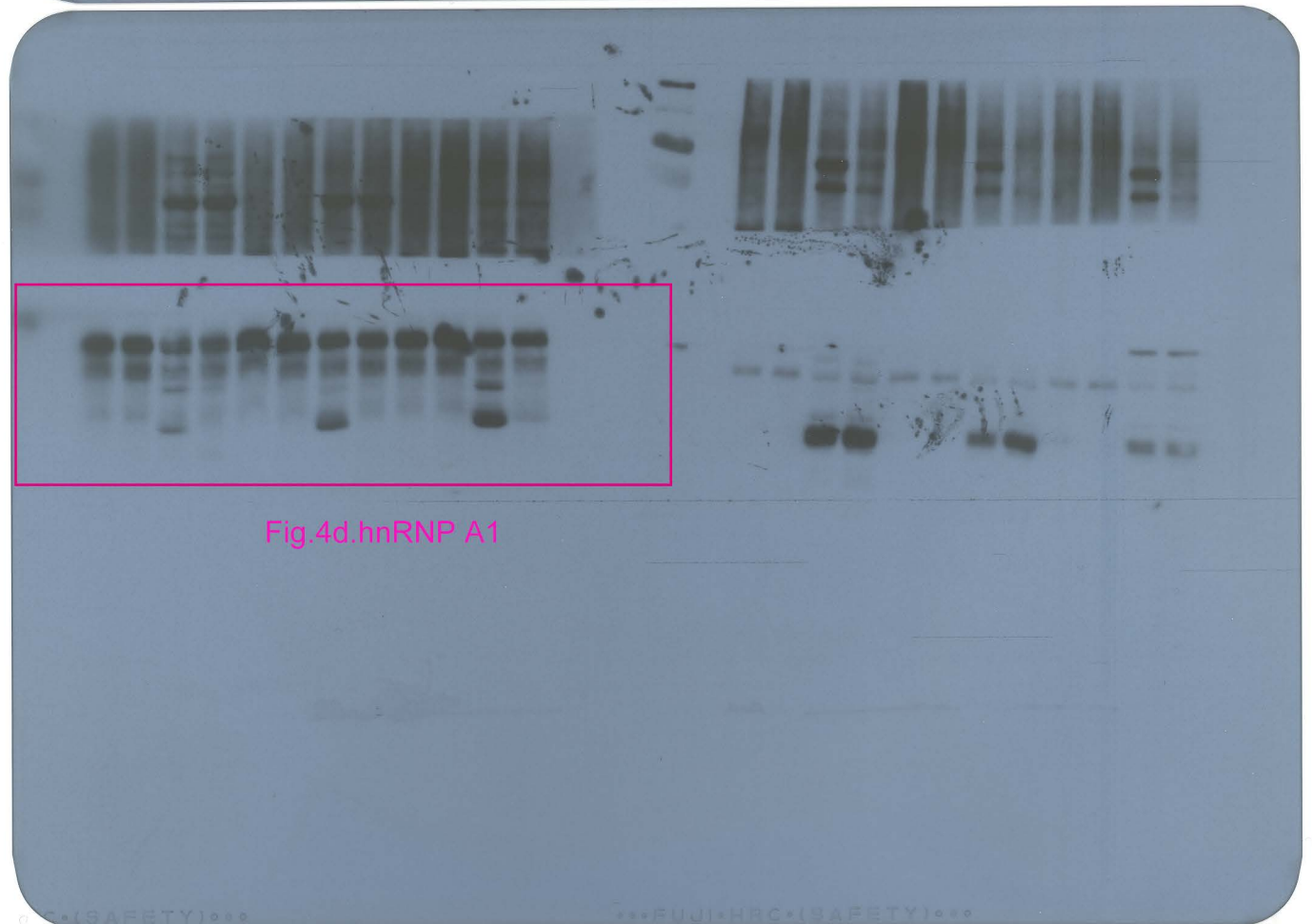

Fig.4d.hnRNP A1

Supplement: Supplementary file 8 — Source Data [file 41467_2021_21529_MOESM8_ESM.zip › Uncropped blot and gel images/Figure4/Figure4d/Smn_hnRNP A1.pdf]

SAFETY

SAFETY

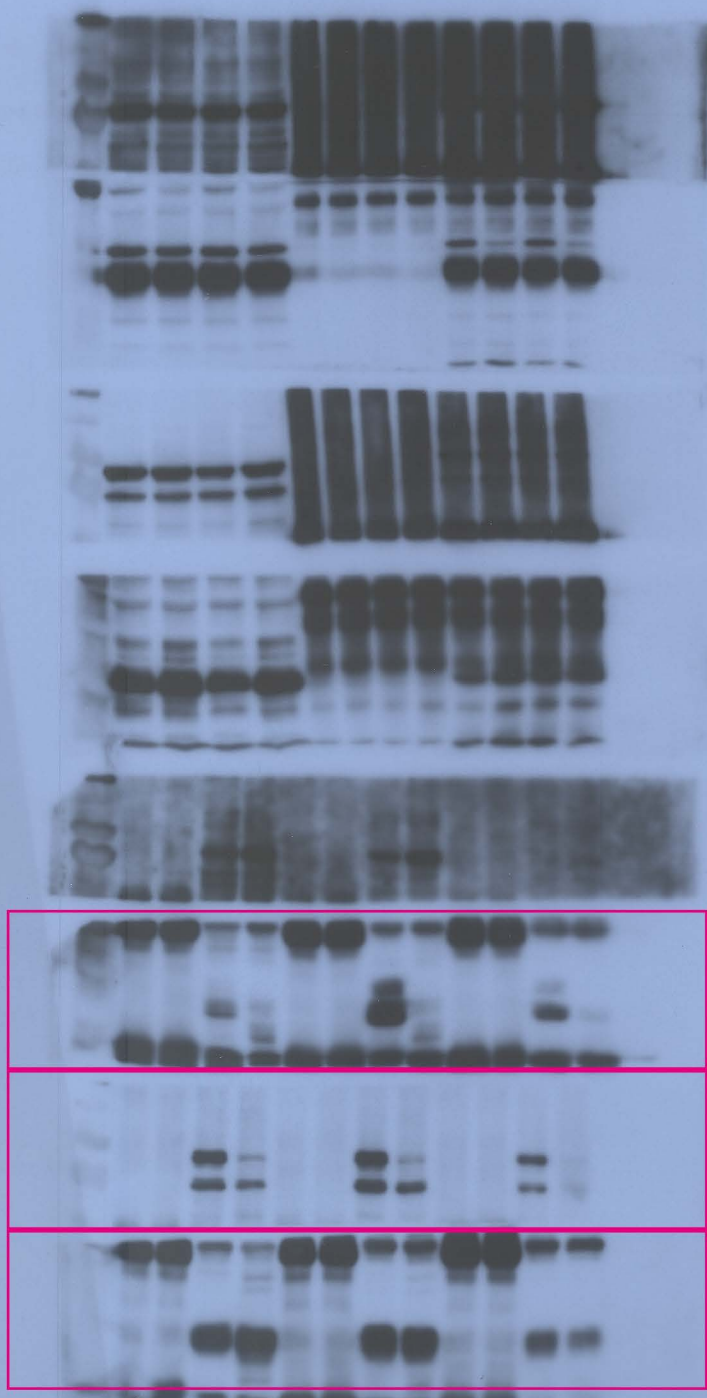

Fig.4e.hnRNP A1

Fig.4e.hnRNP R

Fig.4e.Smn

Supplement: Supplementary file 8 — Source Data [file 41467_2021_21529_MOESM8_ESM.zip › Uncropped blot and gel images/Figure4/Figure4e/hnRNP A1_hnRNP R_Smn.pdf]

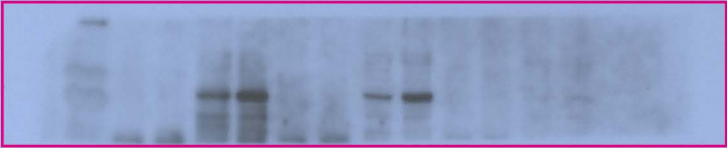

Fig.4e.Larp7

Supplement: Supplementary file 8 — Source Data [file 41467_2021_21529_MOESM8_ESM.zip › Uncropped blot and gel images/Figure4/Figure4e/Larp7.pdf]

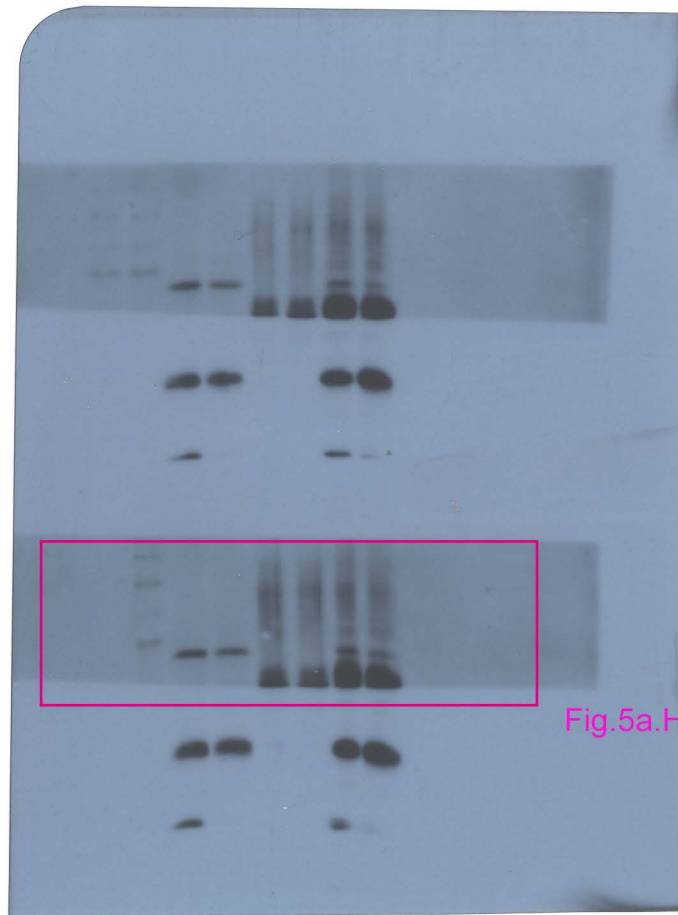

Fig.5a.Hexim1

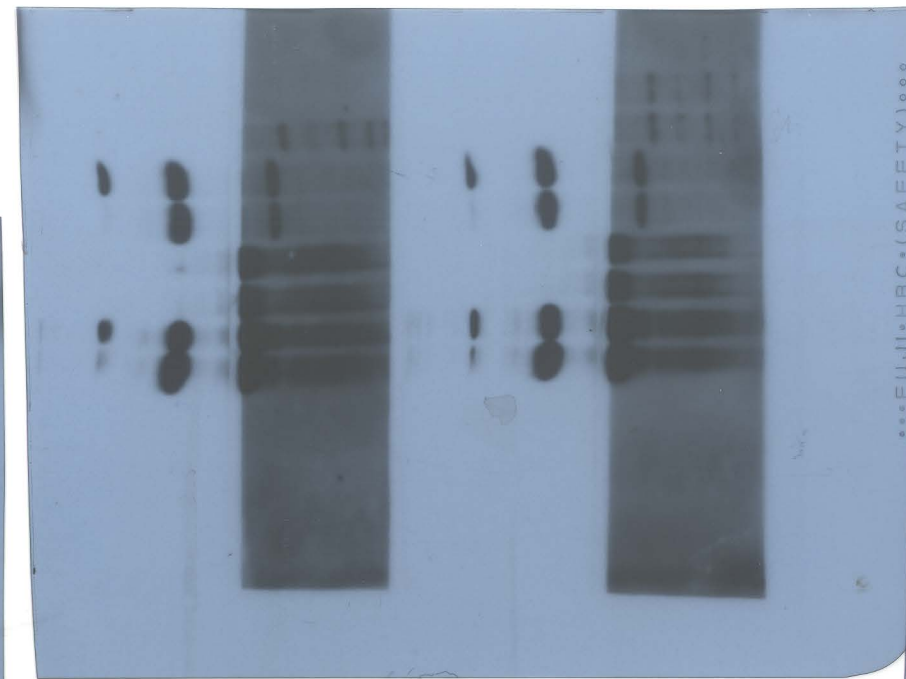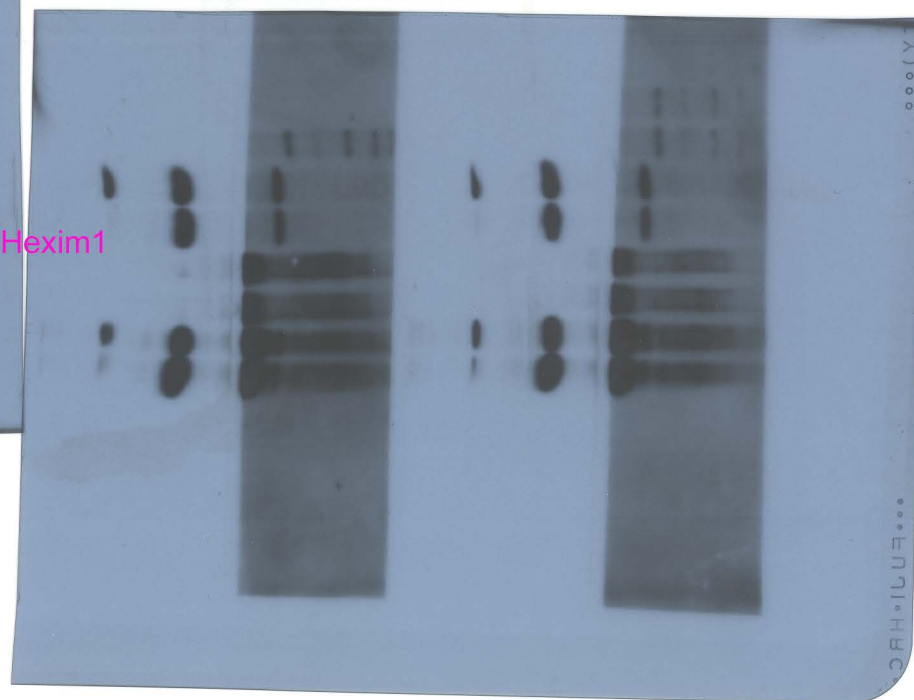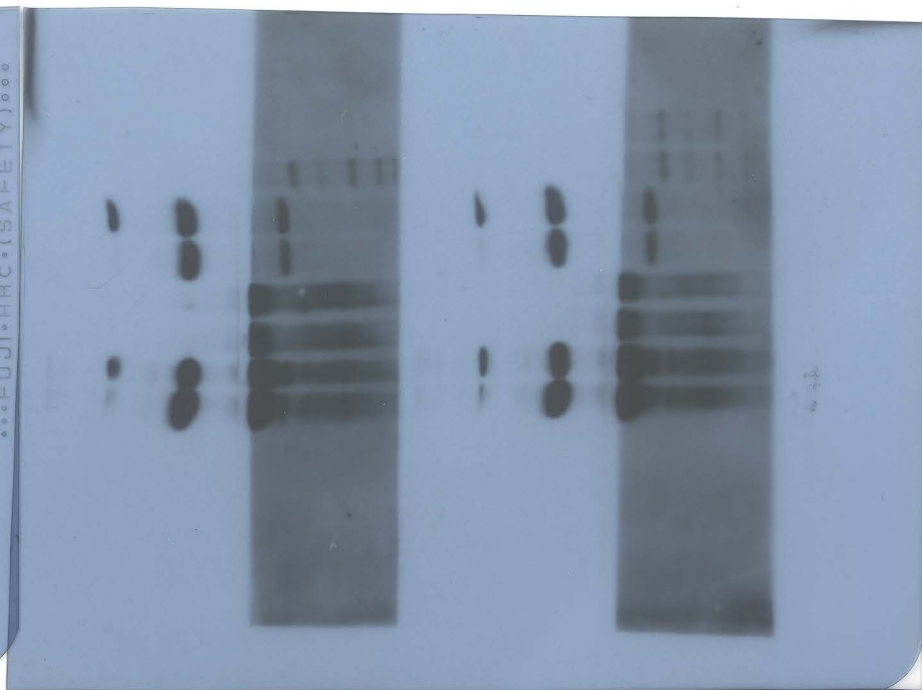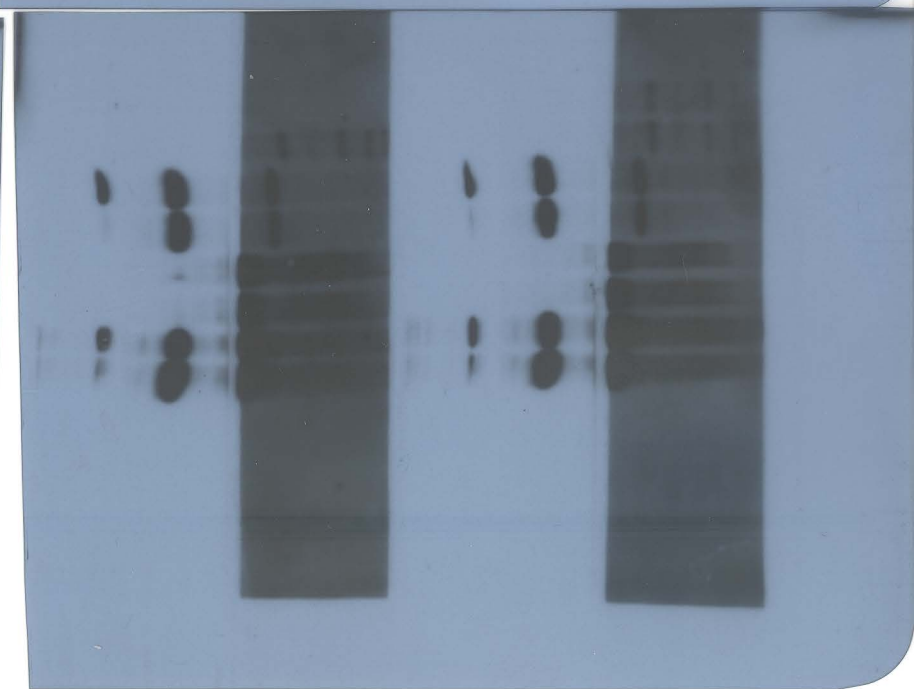

Supplement: Supplementary file 8 — Source Data [file 41467_2021_21529_MOESM8_ESM.zip › Uncropped blot and gel images/Figure5/Figure5a/Hexim1.pdf]

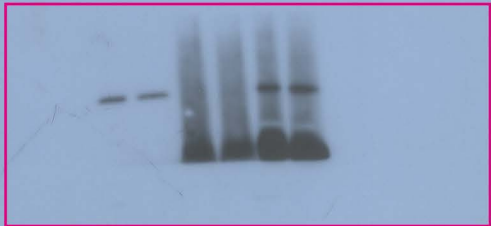

Fig.5a.Larp7

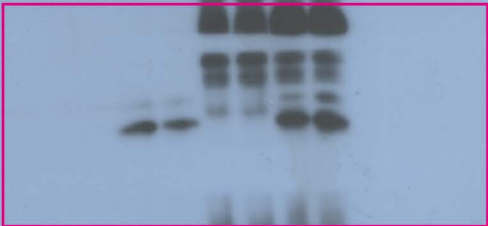

Fig.5a.Gemin2

Supplement: Supplementary file 8 — Source Data [file 41467_2021_21529_MOESM8_ESM.zip › Uncropped blot and gel images/Figure5/Figure5a/Larp7_Gemin2.pdf]

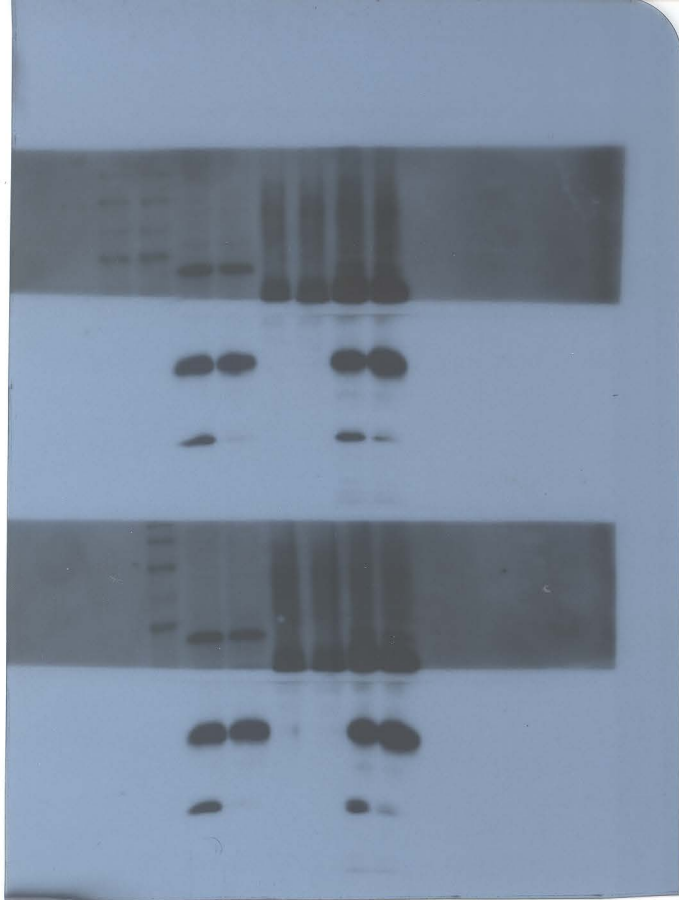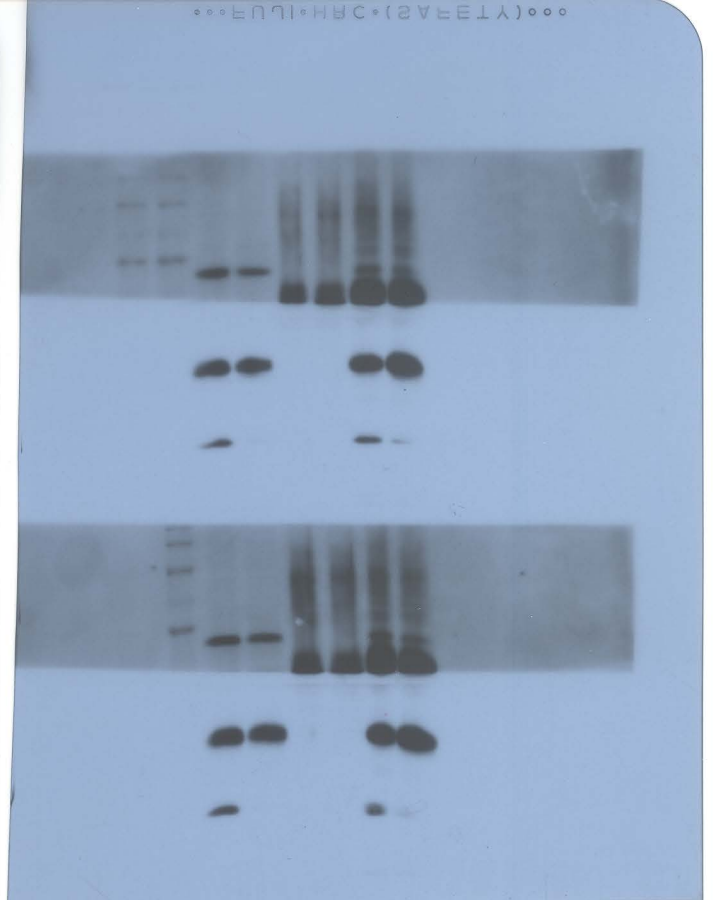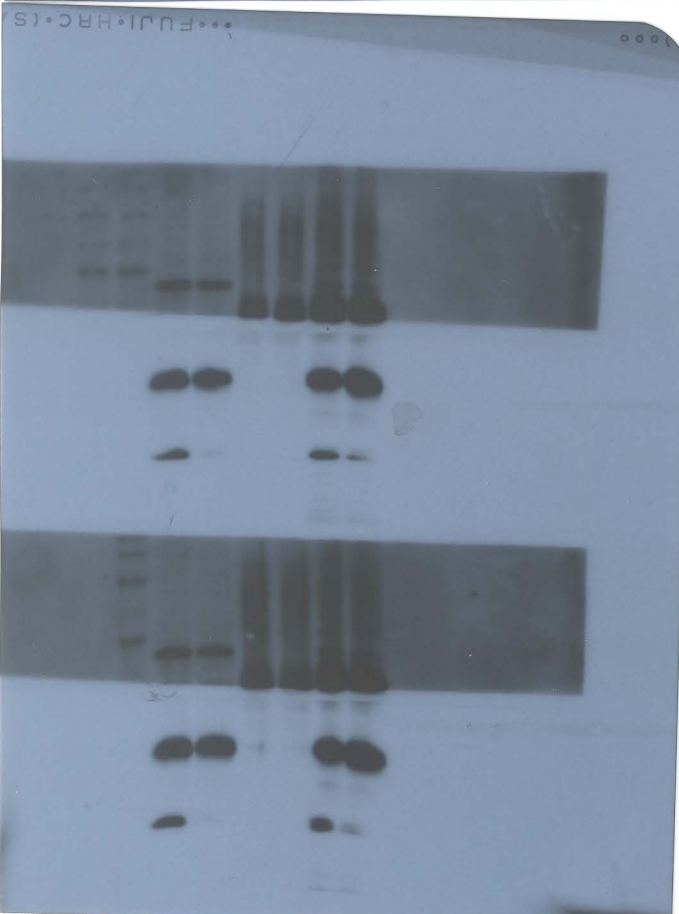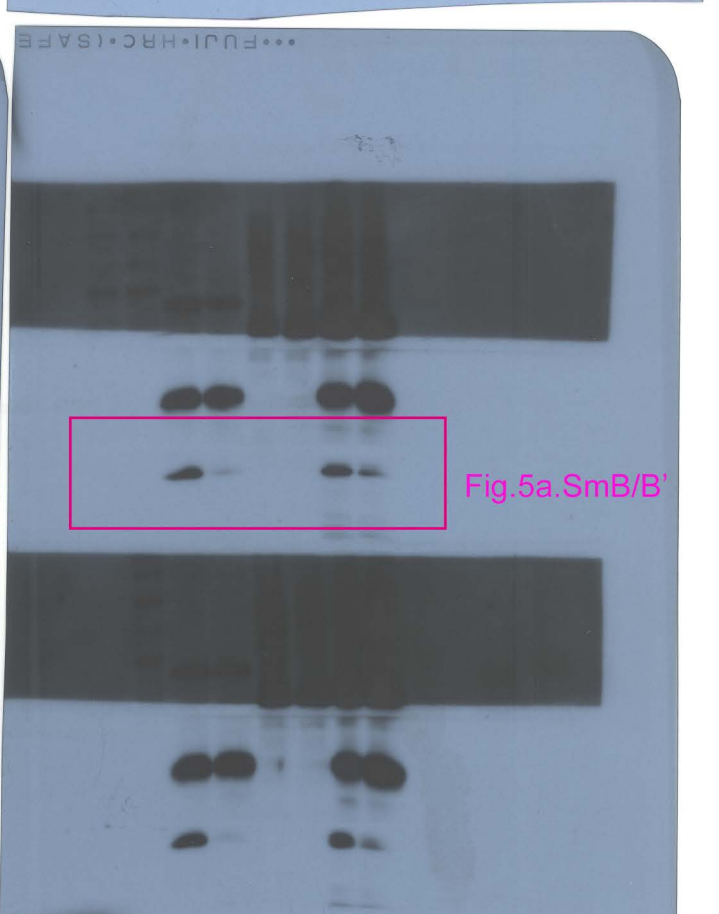

Fig.5a.SmB/B'

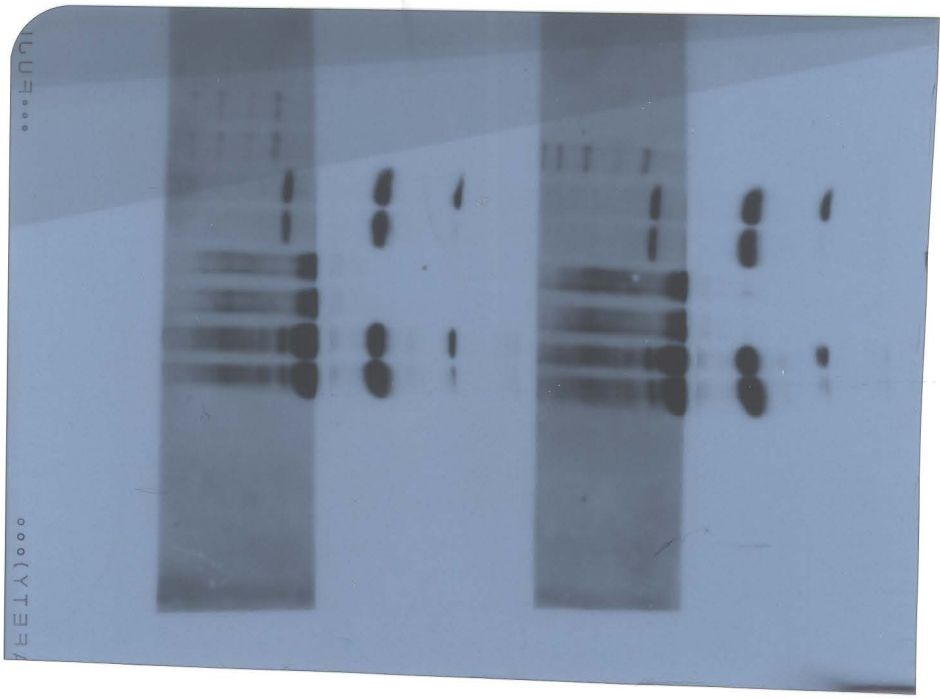

Supplement: Supplementary file 8 — Source Data [file 41467_2021_21529_MOESM8_ESM.zip › Uncropped blot and gel images/Figure5/Figure5a/SmB.pdf]

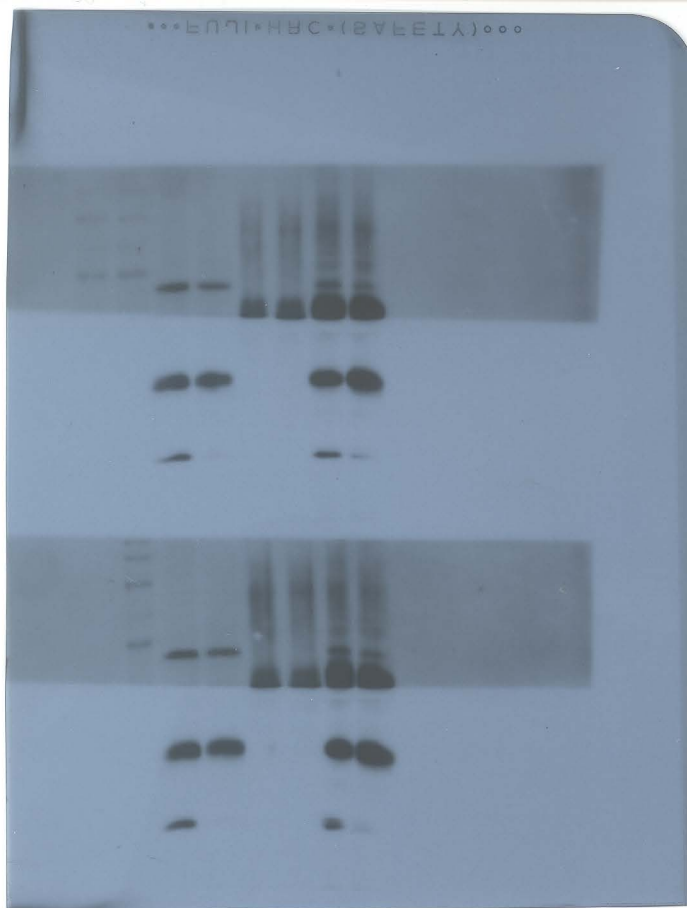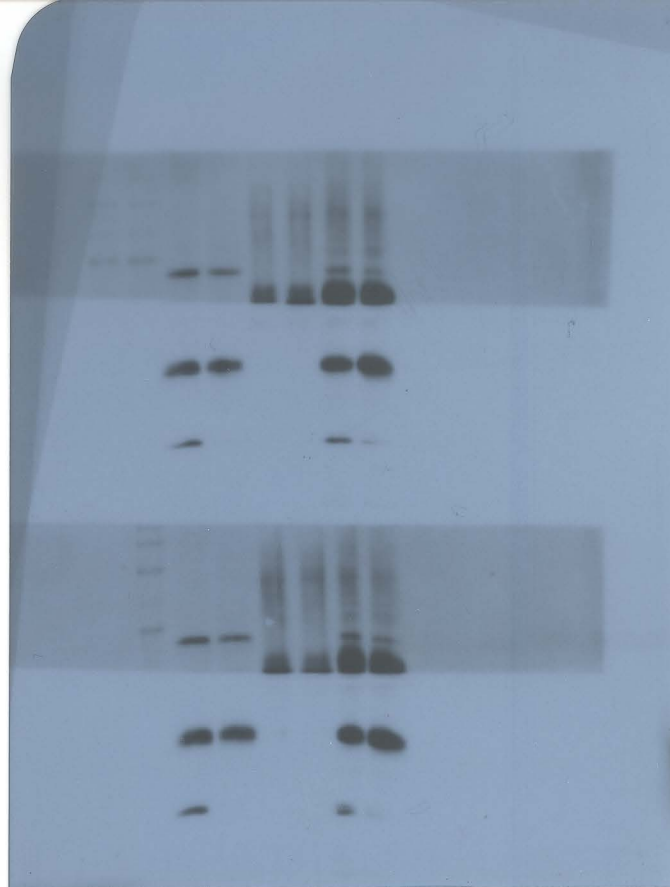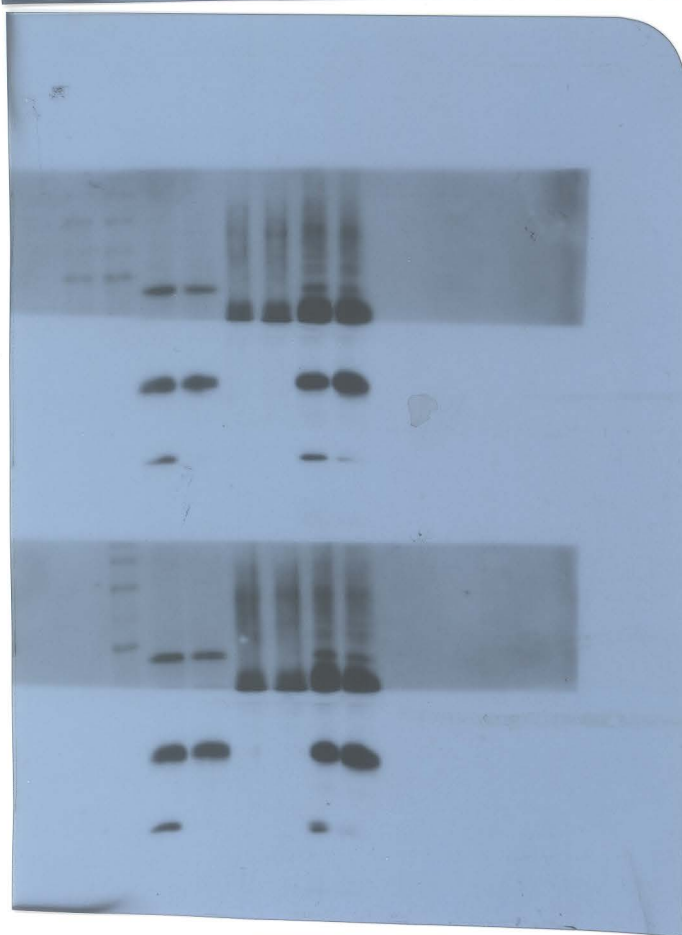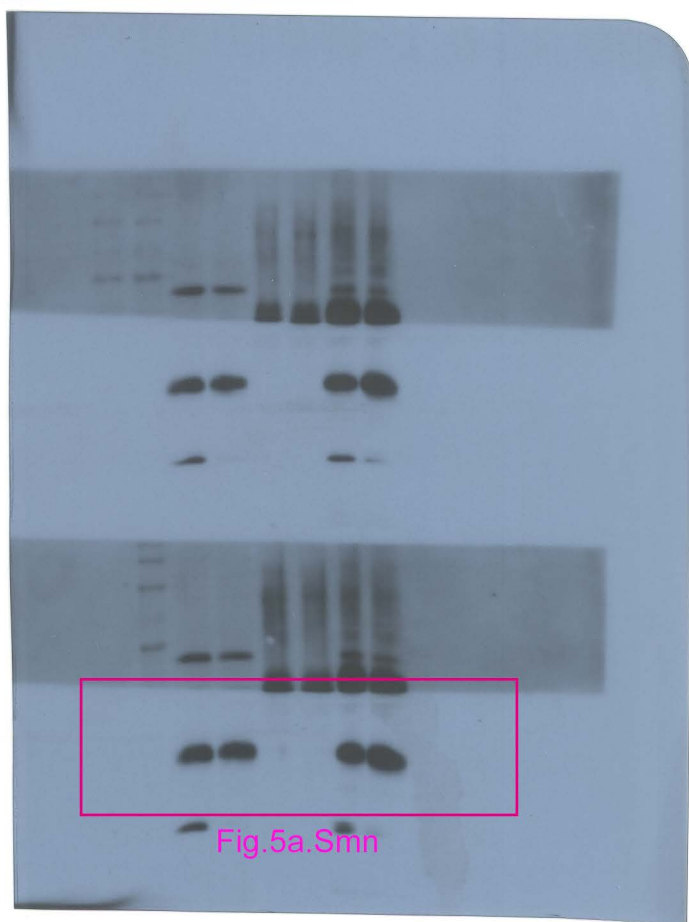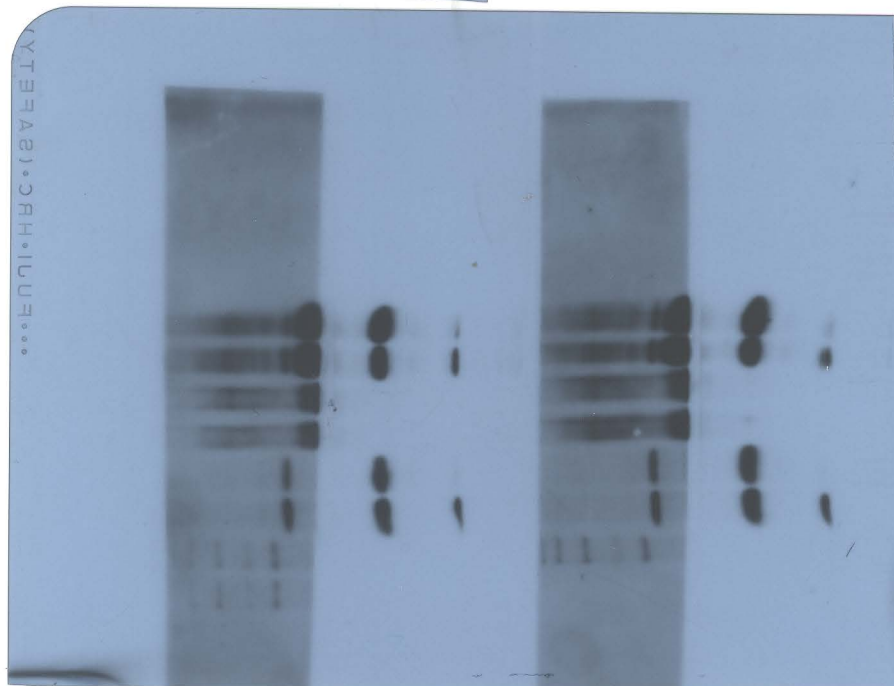

Supplement: Supplementary file 8 — Source Data [file 41467_2021_21529_MOESM8_ESM.zip › Uncropped blot and gel images/Figure5/Figure5a/Smn.pdf]

000(YT37A2)•3BH•LUN•000

000(YT37A2)•3BH•LUN•000

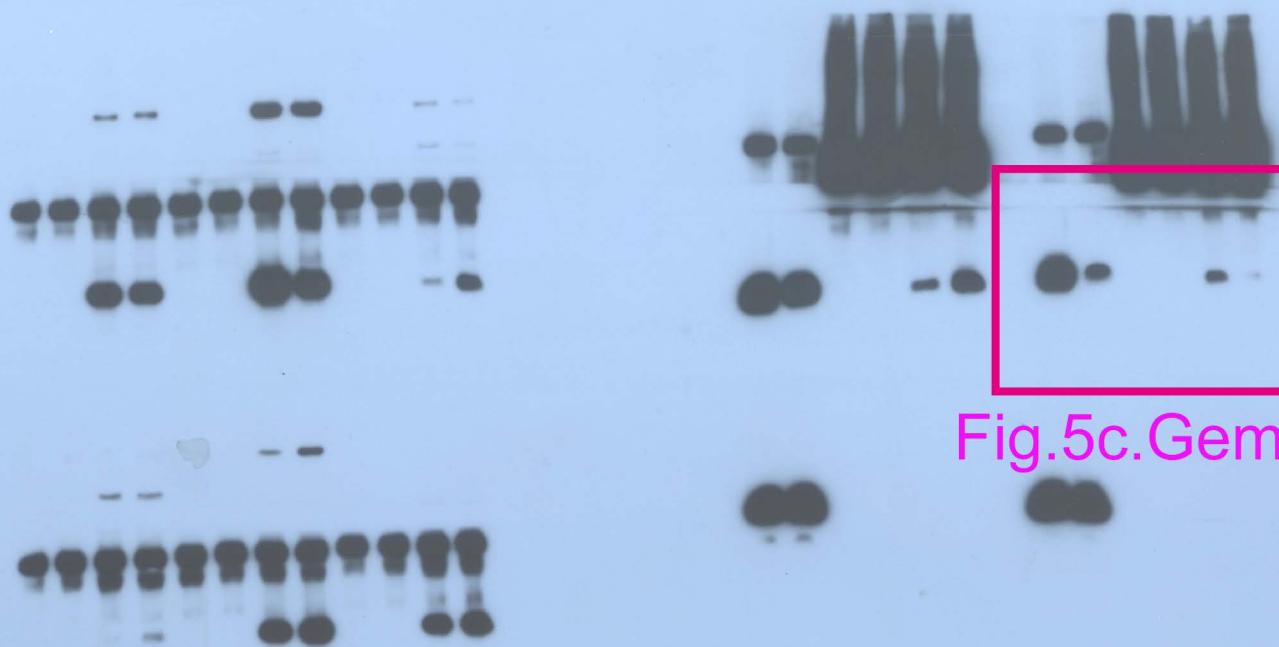

Fig.5c.Gemin2

Supplement: Supplementary file 8 — Source Data [file 41467_2021_21529_MOESM8_ESM.zip › Uncropped blot and gel images/Figure5/Figure5c/Gemin2.pdf]

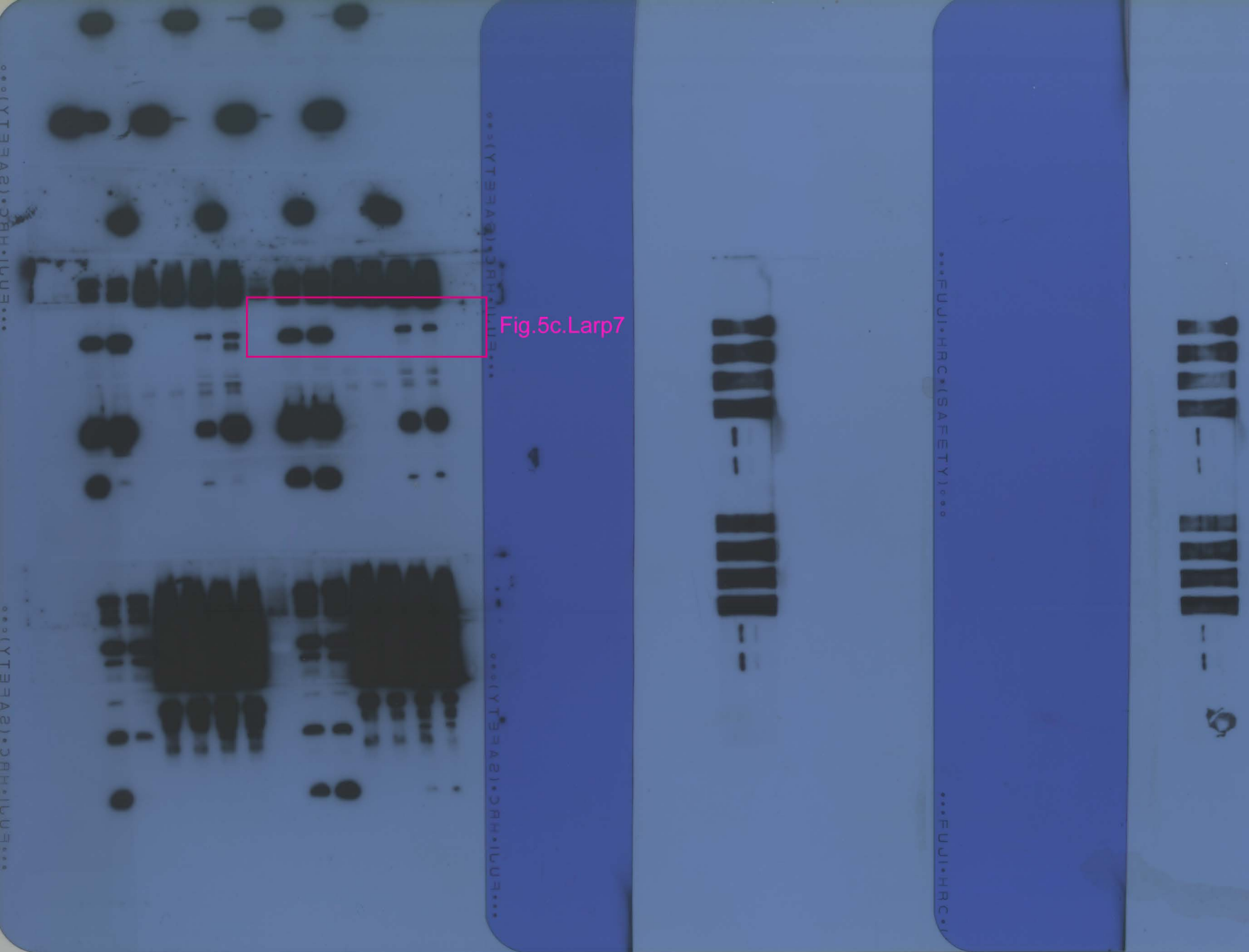

Supplement: Supplementary file 8 — Source Data [file 41467_2021_21529_MOESM8_ESM.zip › Uncropped blot and gel images/Figure5/Figure5c/Larp7.pdf]

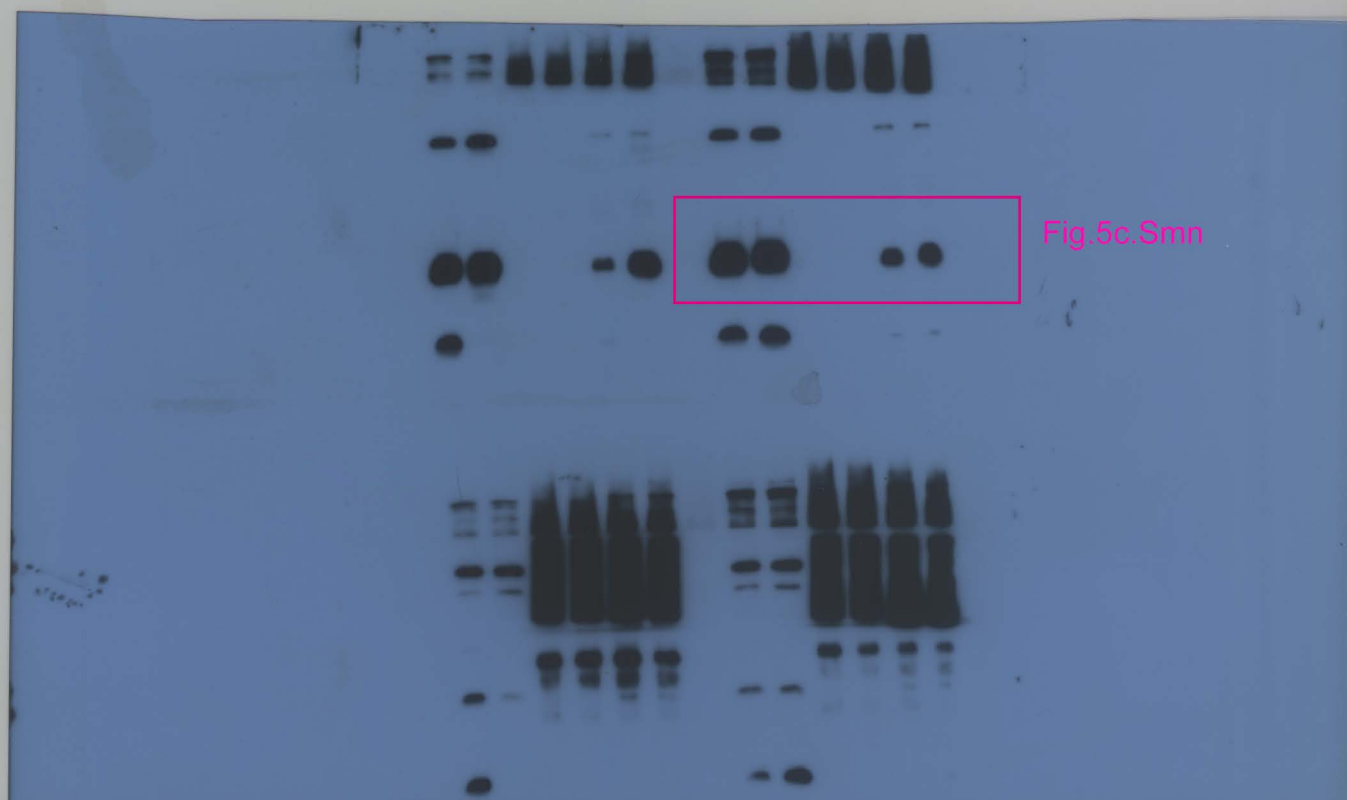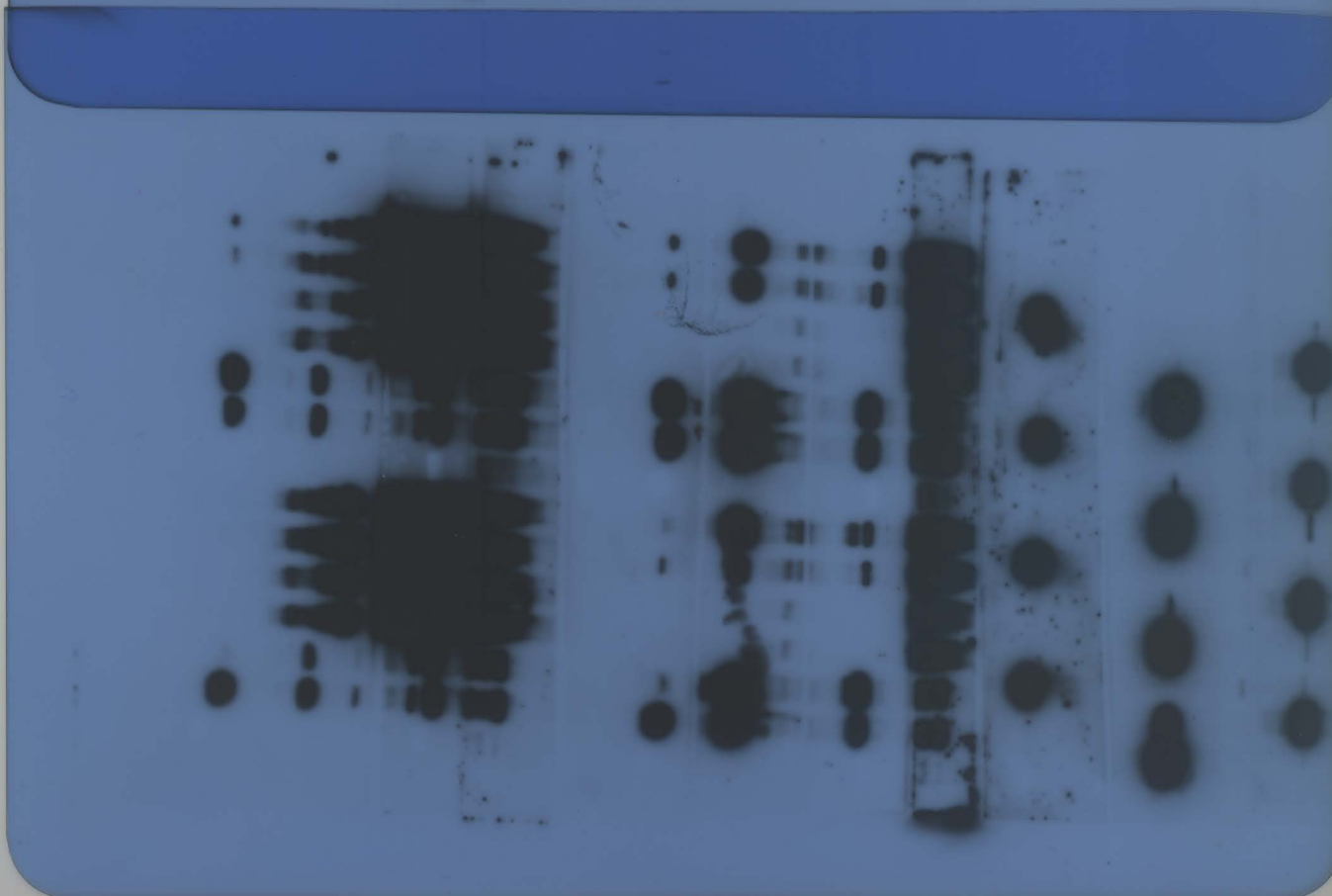

Supplement: Supplementary file 8 — Source Data [file 41467_2021_21529_MOESM8_ESM.zip › Uncropped blot and gel images/Figure5/Figure5c/Smn.pdf]

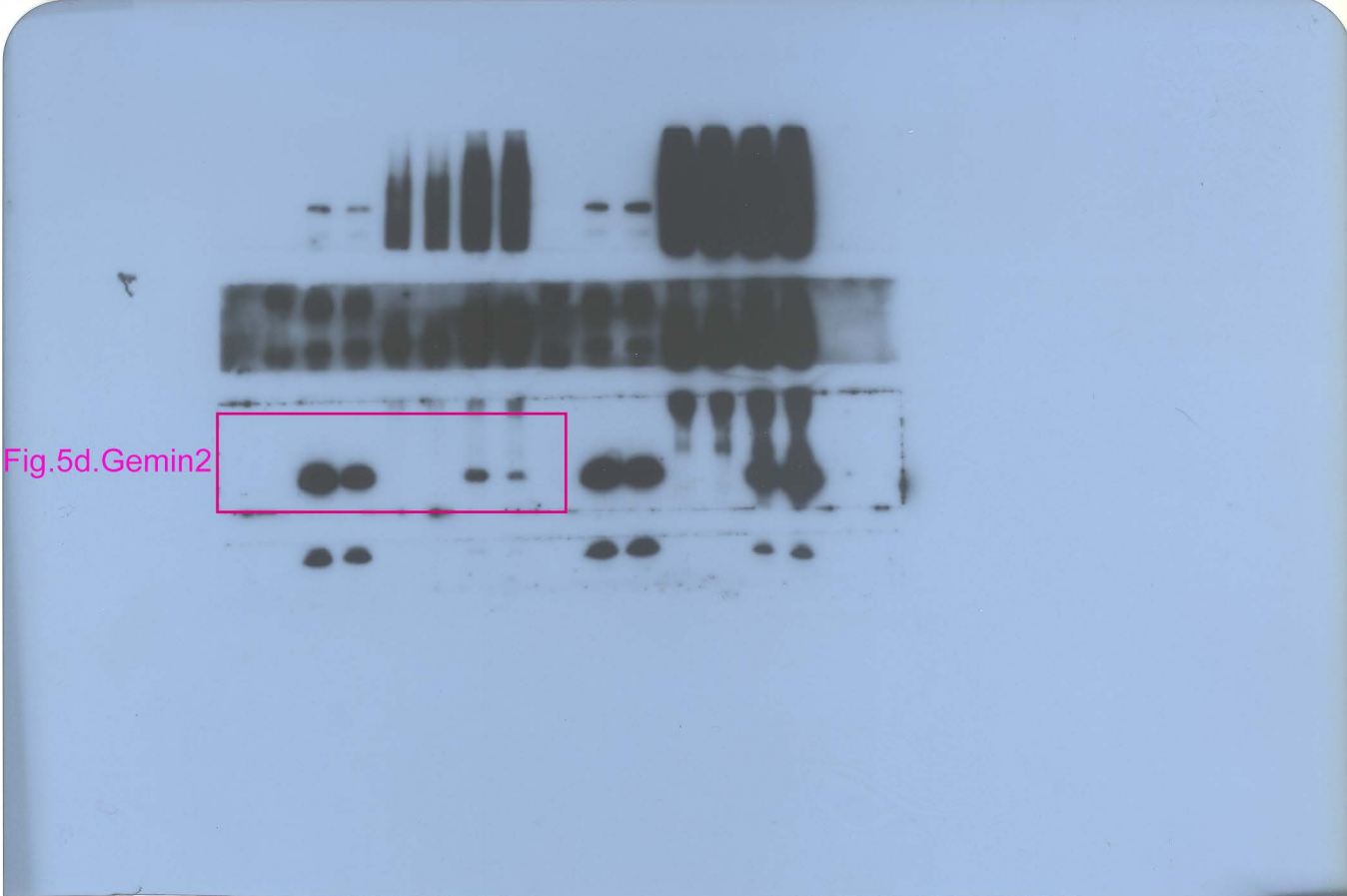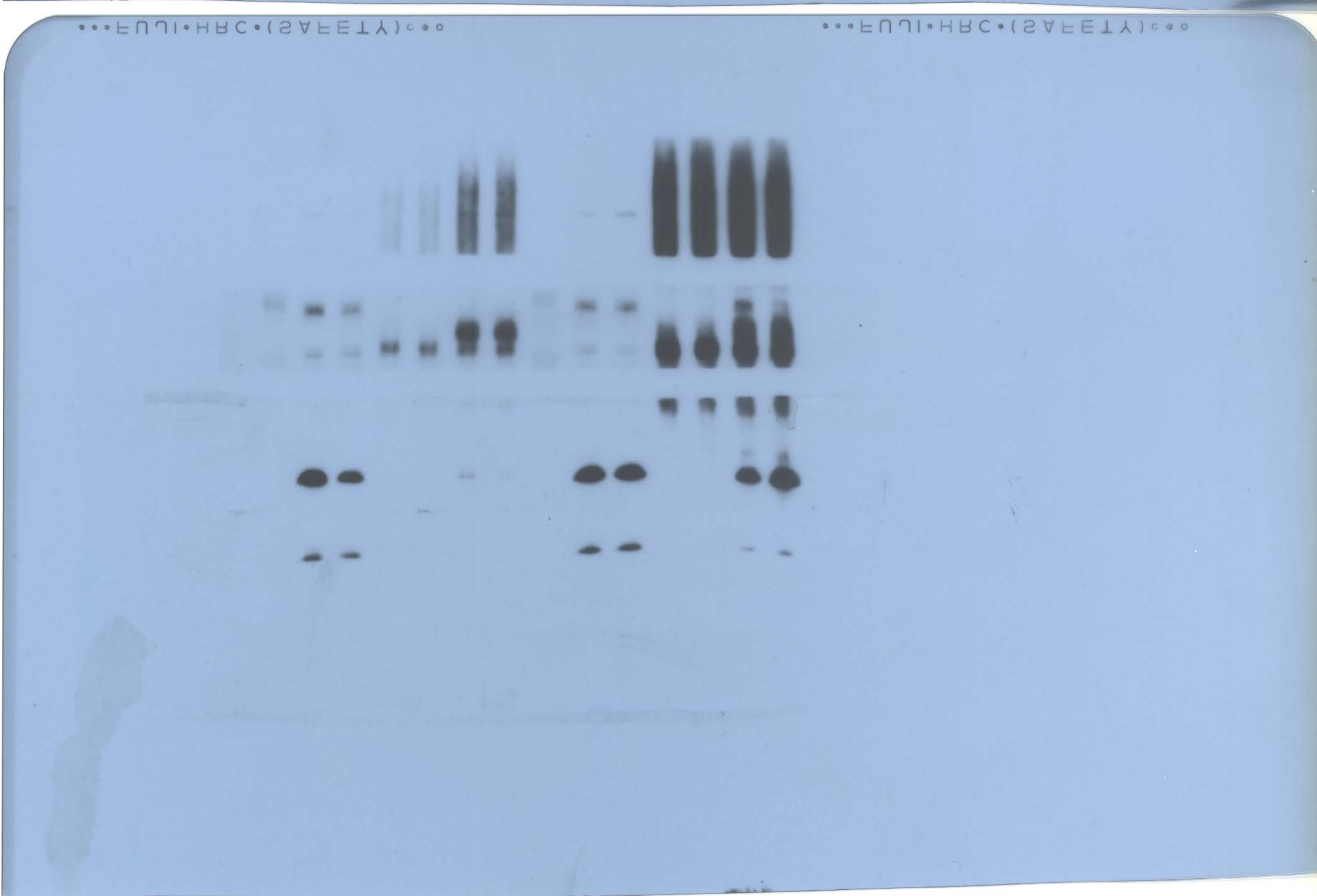

Supplement: Supplementary file 8 — Source Data [file 41467_2021_21529_MOESM8_ESM.zip › Uncropped blot and gel images/Figure5/Figure5d/Gemin2.pdf]

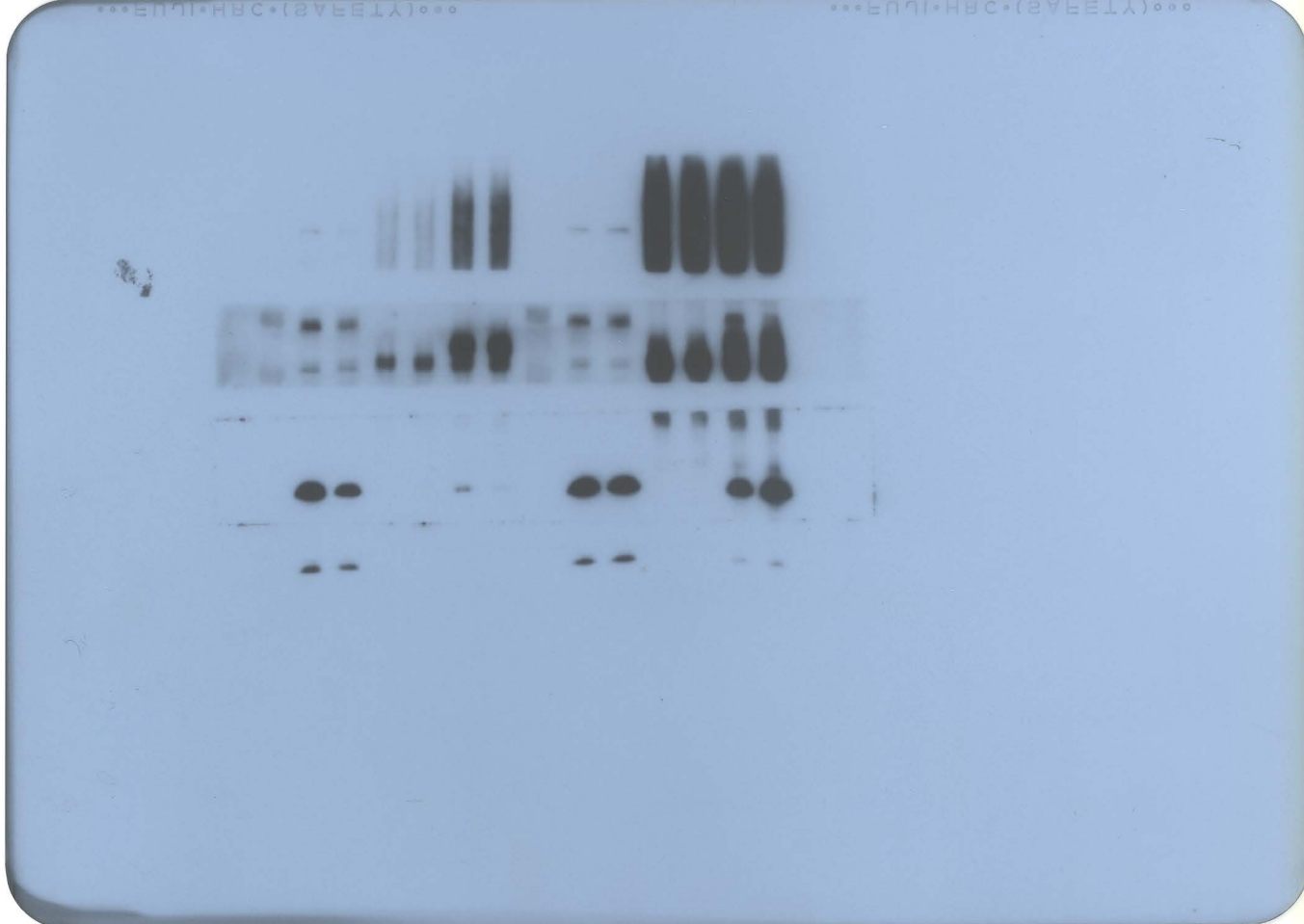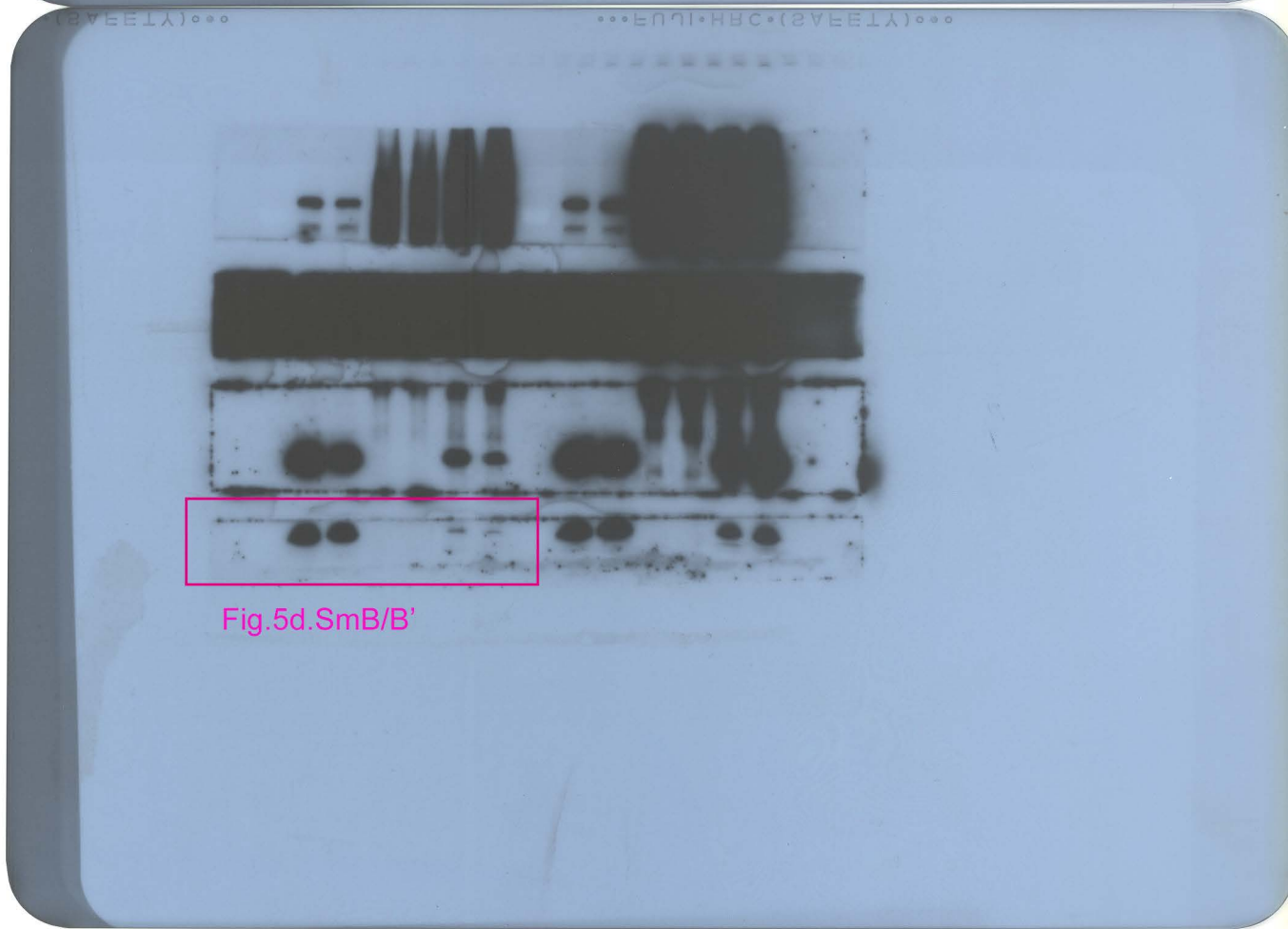

Fig.5d.SmB/B'

Supplement: Supplementary file 8 — Source Data [file 41467_2021_21529_MOESM8_ESM.zip › Uncropped blot and gel images/Figure5/Figure5d/SmB.pdf]

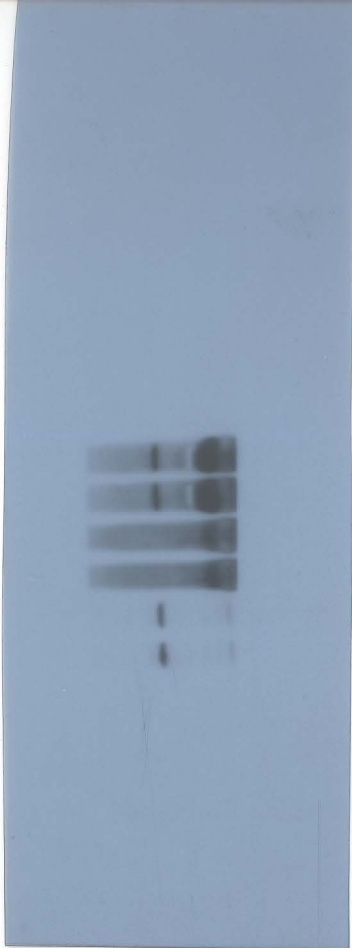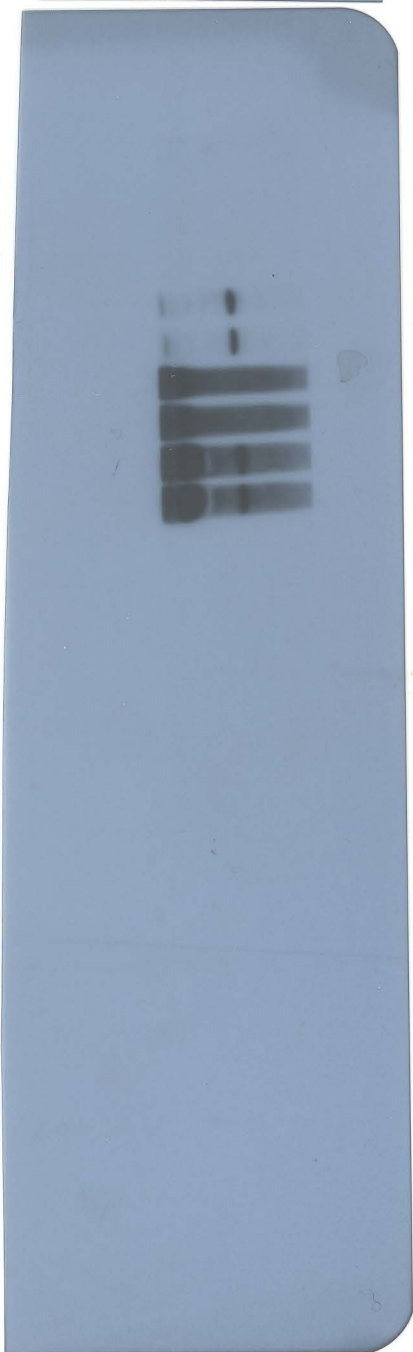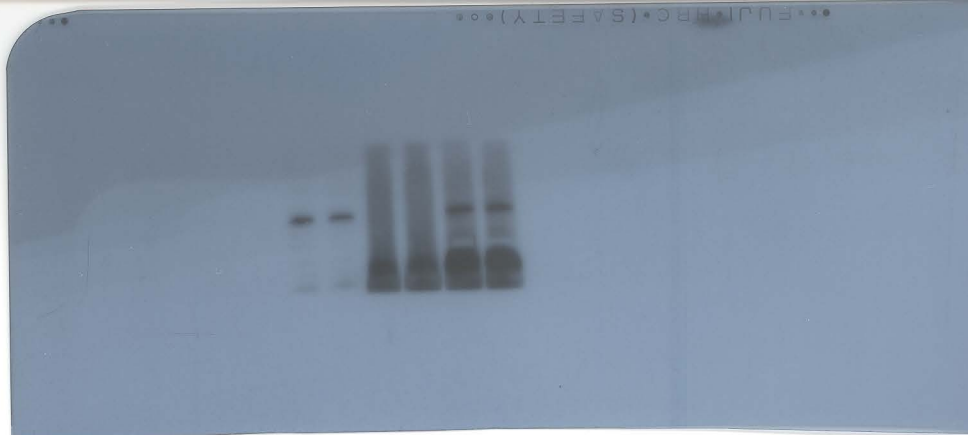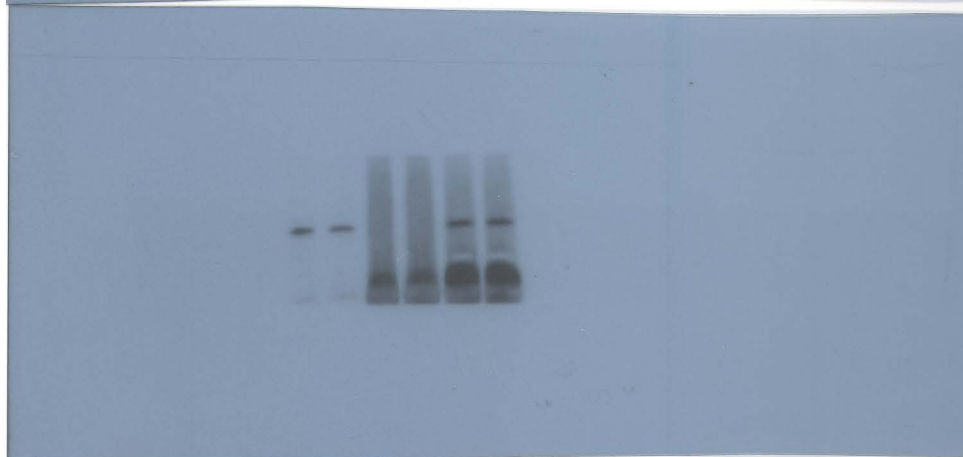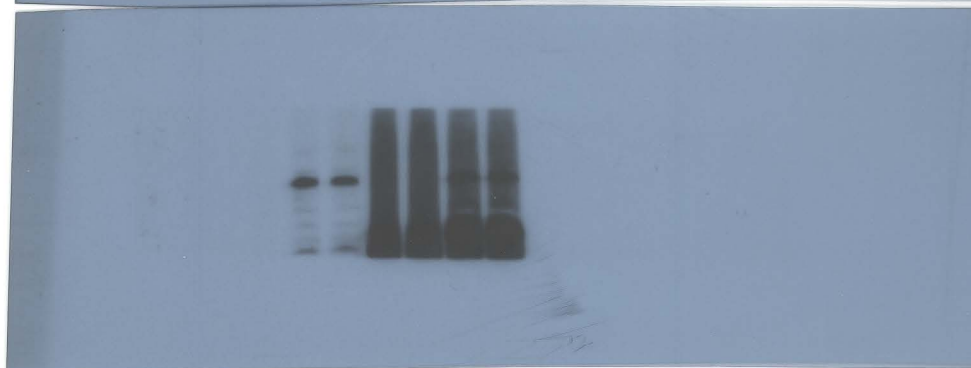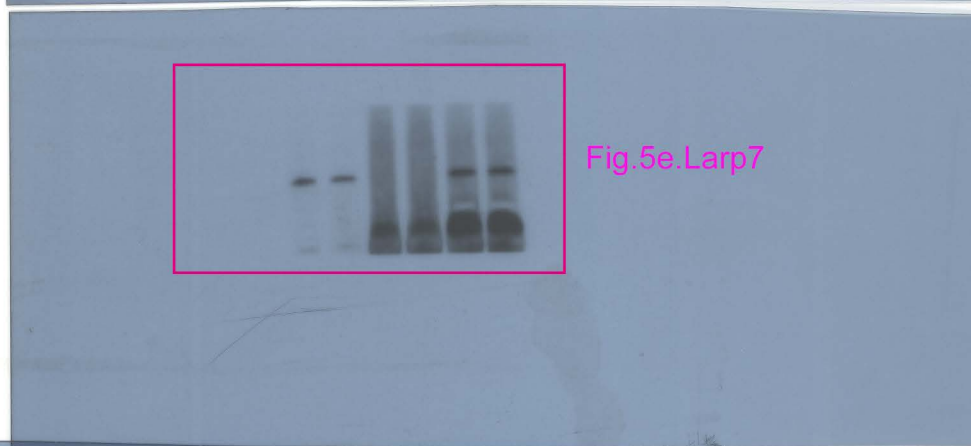

Fig. 5e.Larp7

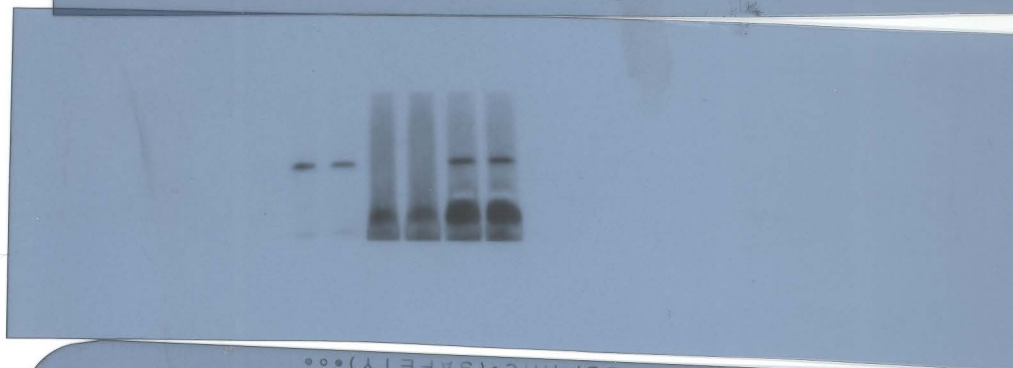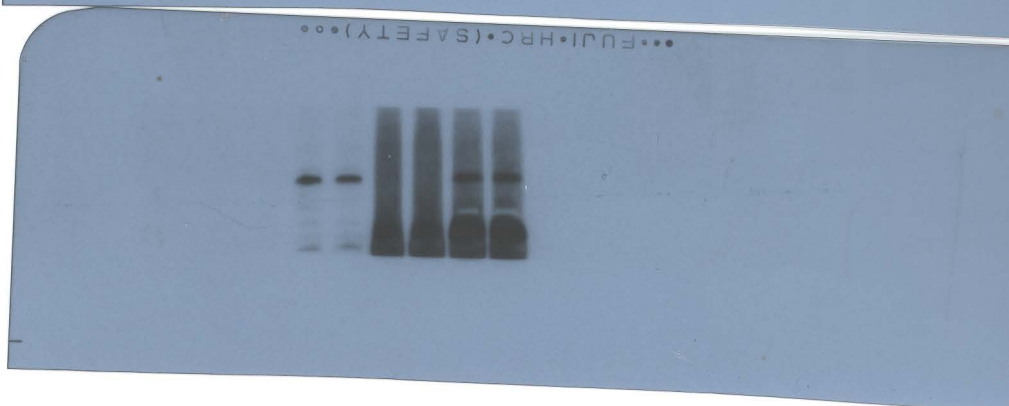

Supplement: Supplementary file 8 — Source Data [file 41467_2021_21529_MOESM8_ESM.zip › Uncropped blot and gel images/Figure5/Figure5e/Larp7.pdf]

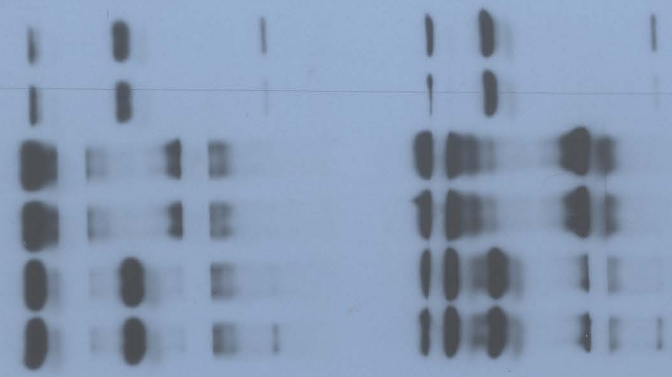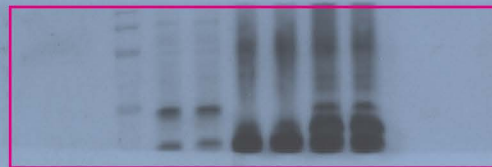

Fig.5e.Hexim1

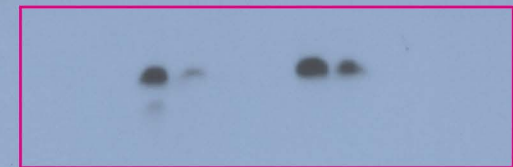

Fig.5e.Smn

...ENH•HRC•(SVEELY)•••  
...ENH•HRC•(SVEELY)•••

Supplement: Supplementary file 8 — Source Data [file 41467_2021_21529_MOESM8_ESM.zip › Uncropped blot and gel images/Figure5/Figure5e/Smn_Hexim1.pdf]

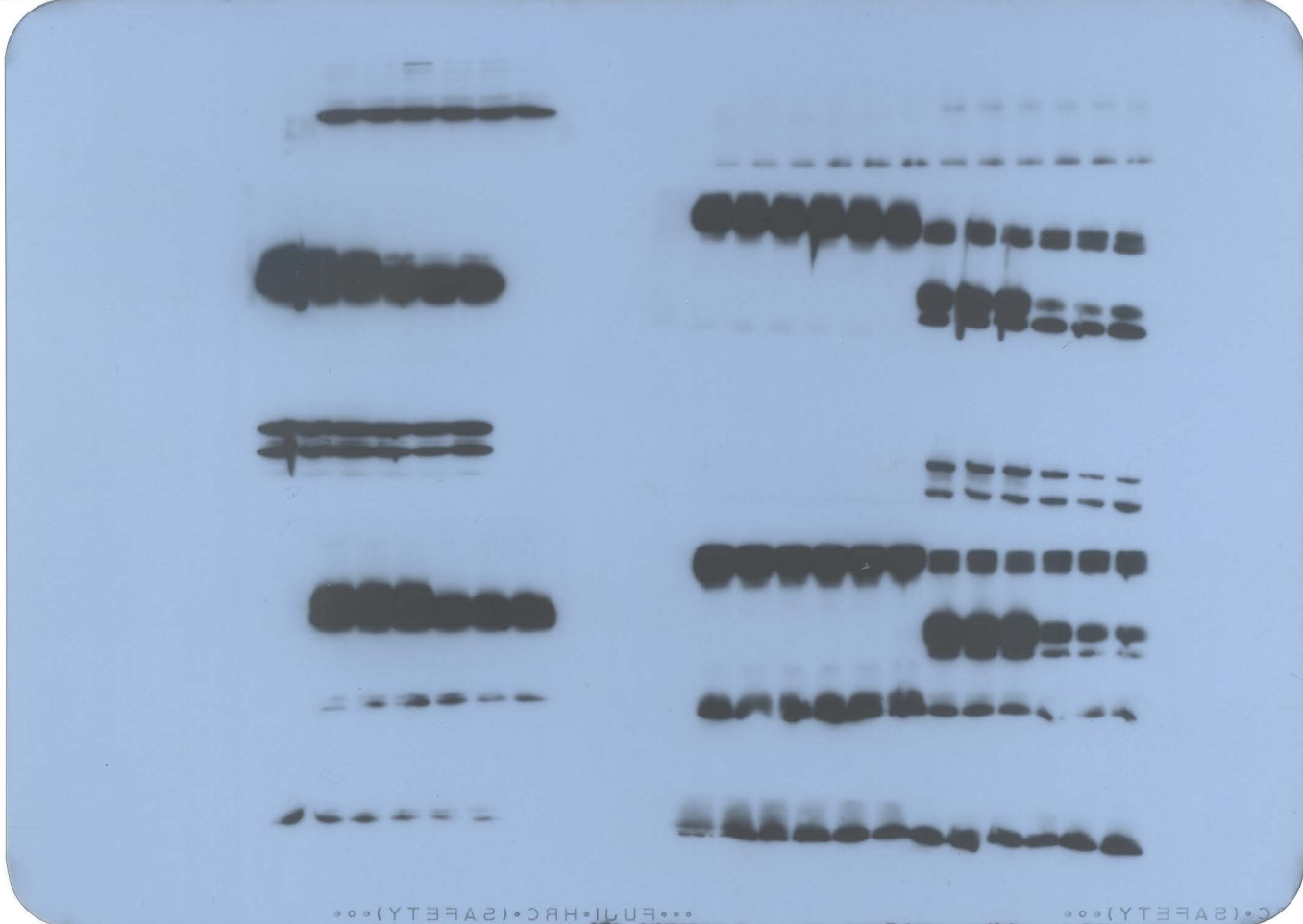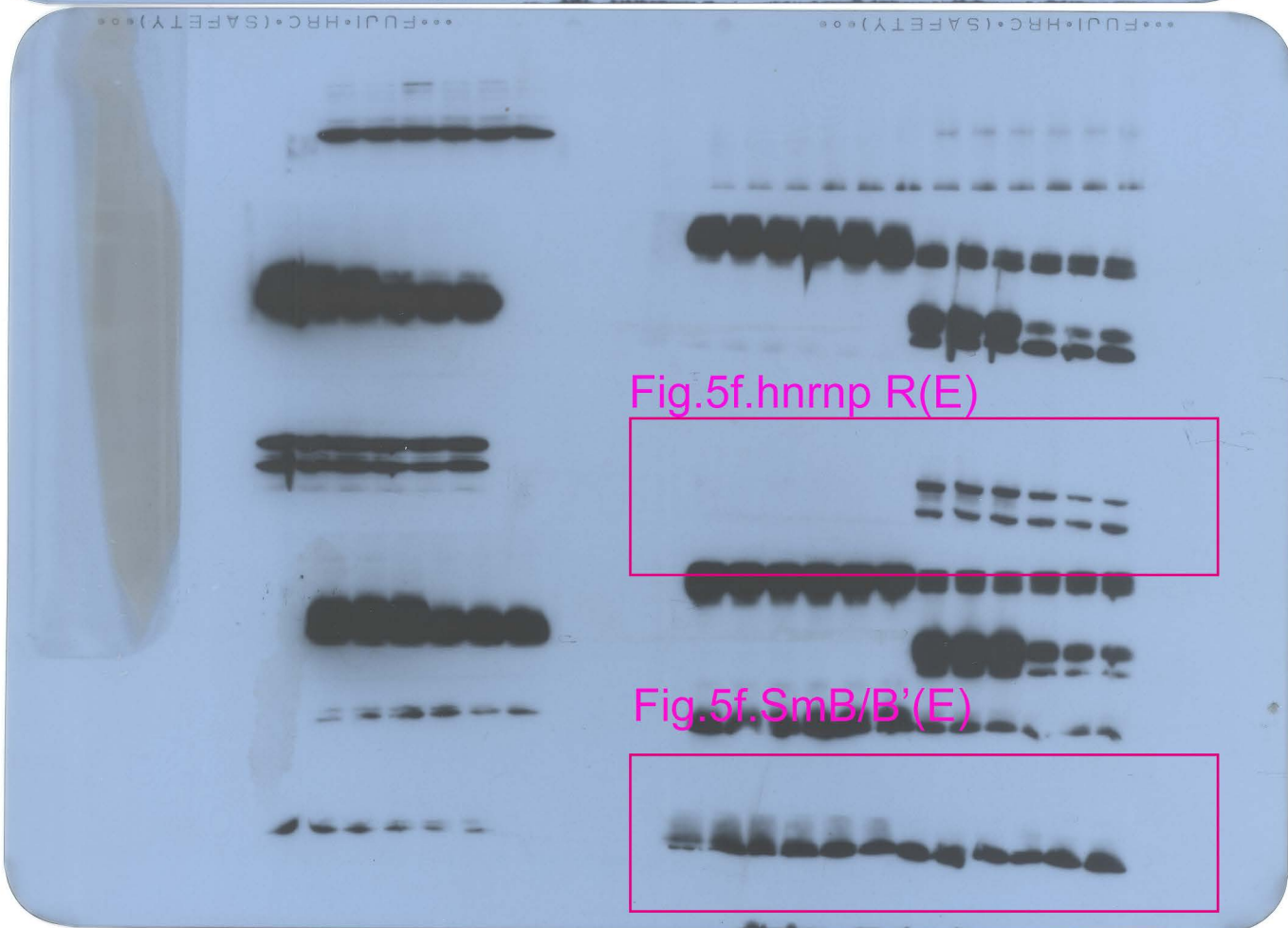

Fig.5f.hnrnp R(E)

Fig.5f.SmB/B'(E)

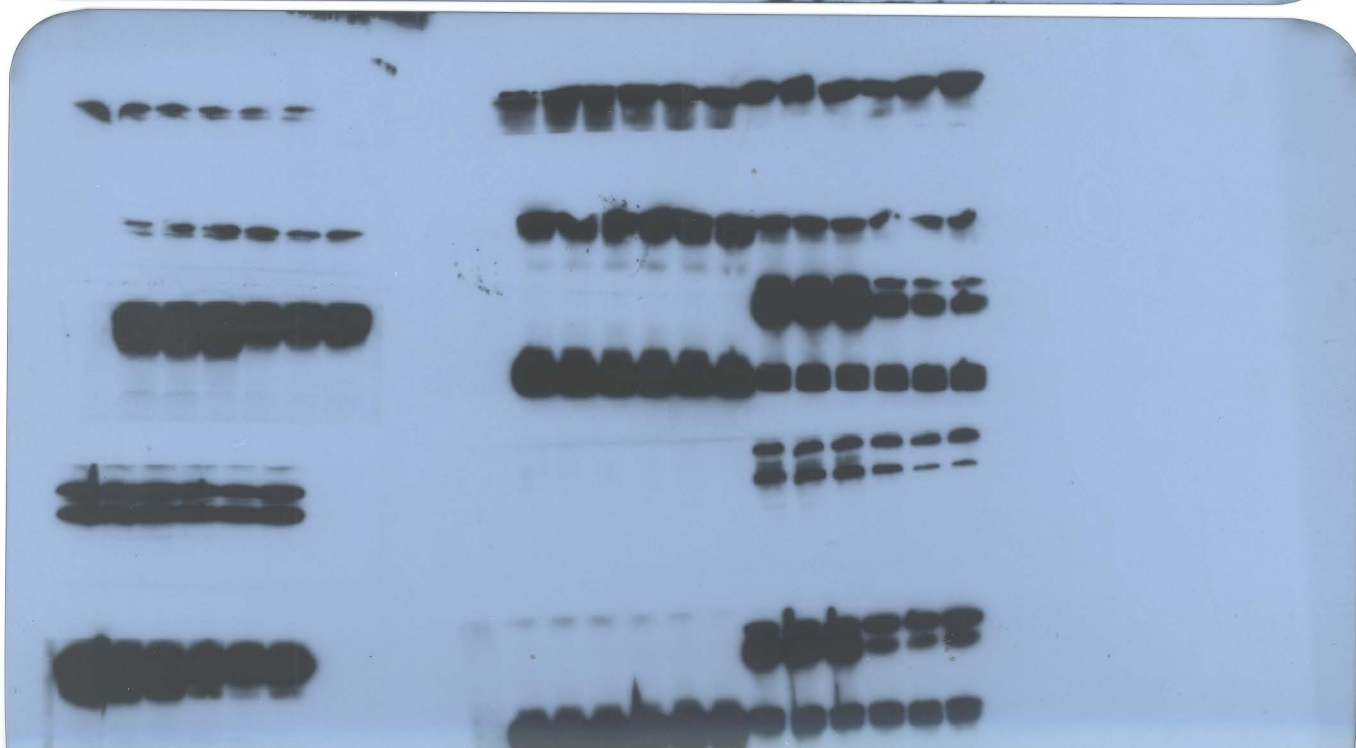

Supplement: Supplementary file 8 — Source Data [file 41467_2021_21529_MOESM8_ESM.zip › Uncropped blot and gel images/Figure5/Figure5f/hnRNP R_SmB.pdf]

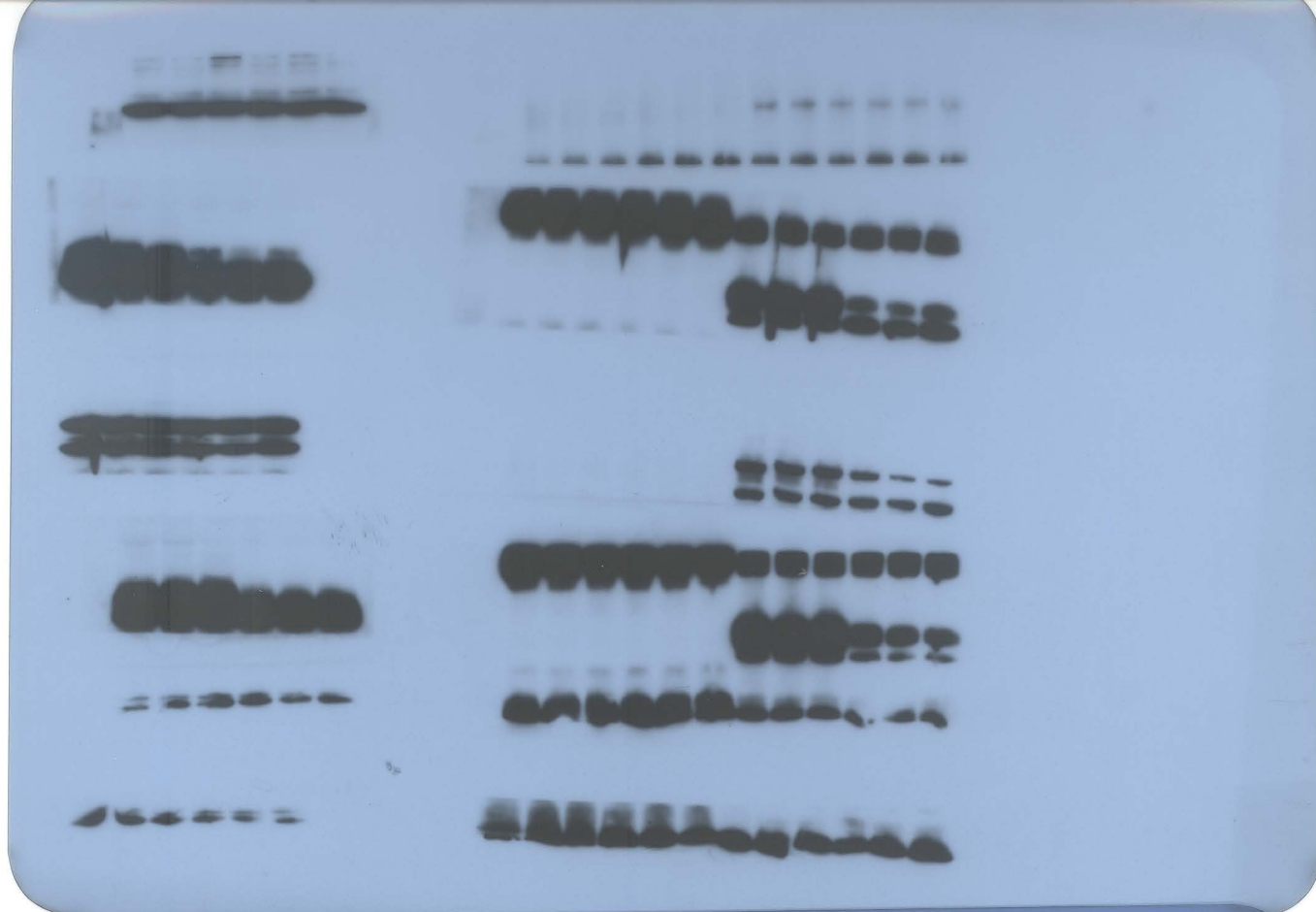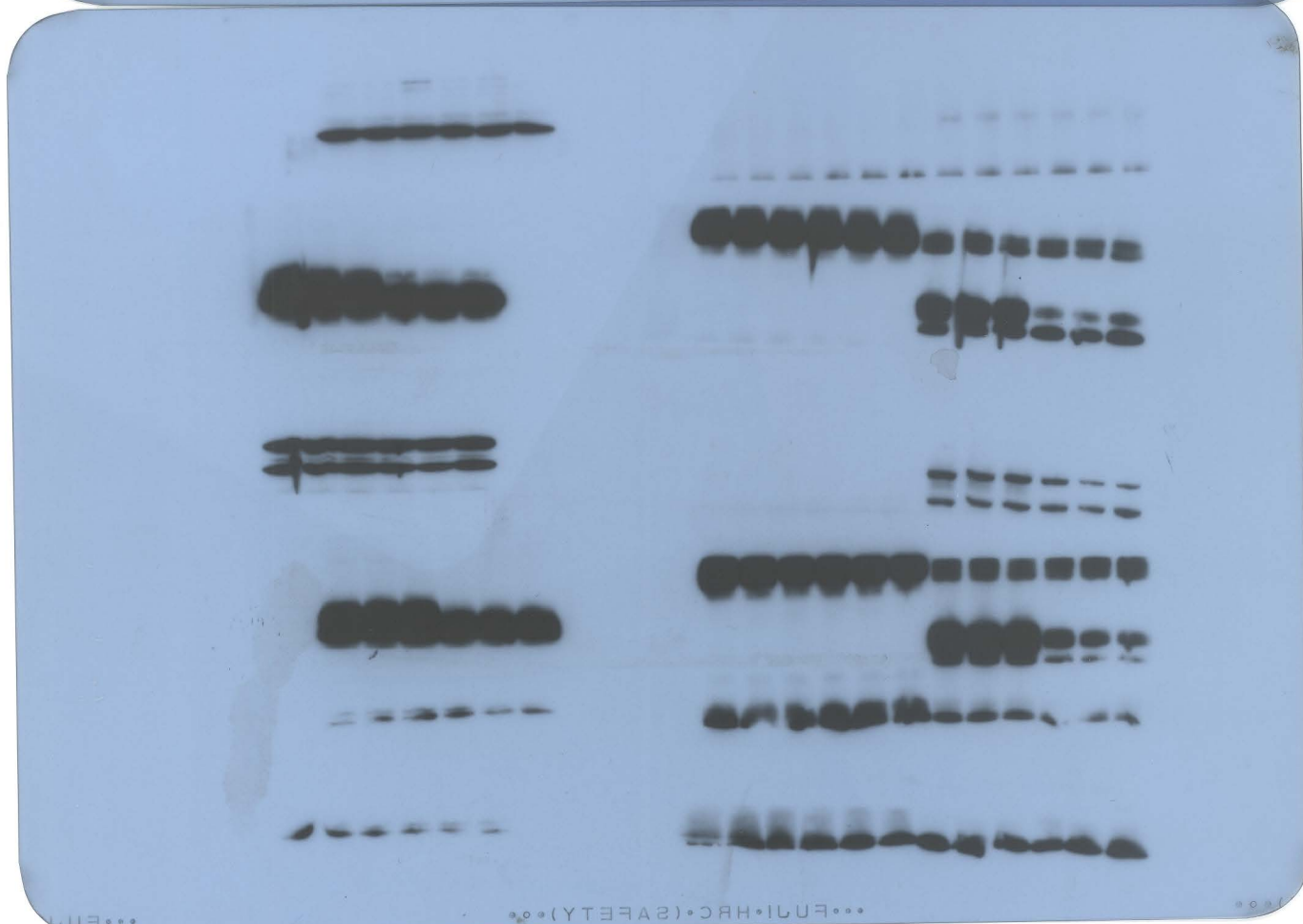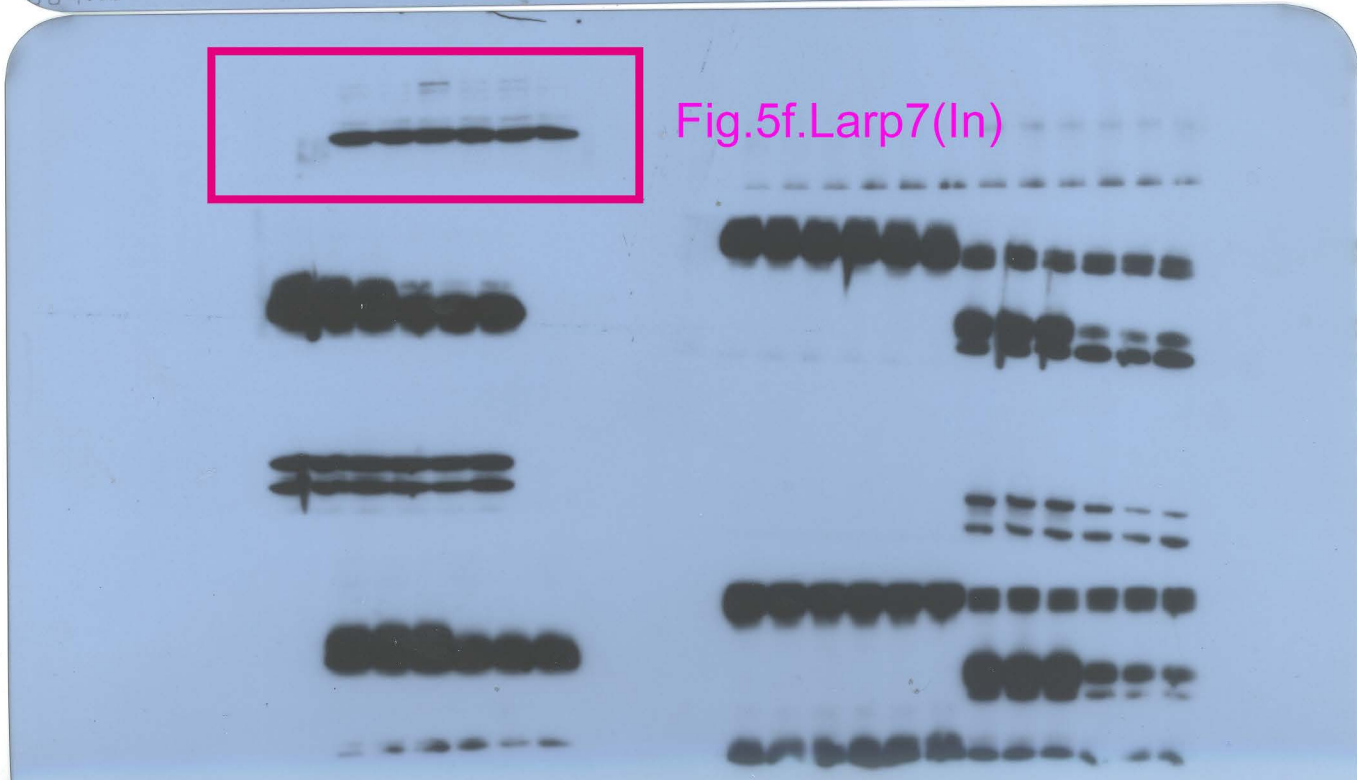

Fig.5f.Larp7(In)

Supplement: Supplementary file 8 — Source Data [file 41467_2021_21529_MOESM8_ESM.zip › Uncropped blot and gel images/Figure5/Figure5f/Larp7.pdf]

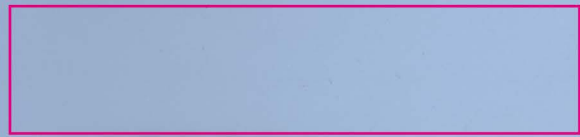

Fig.5f.  
hnRNP A1(E)

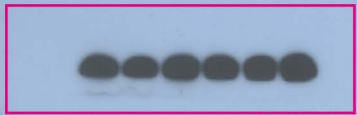

Fig.5f.  
hnRNP A1(In)

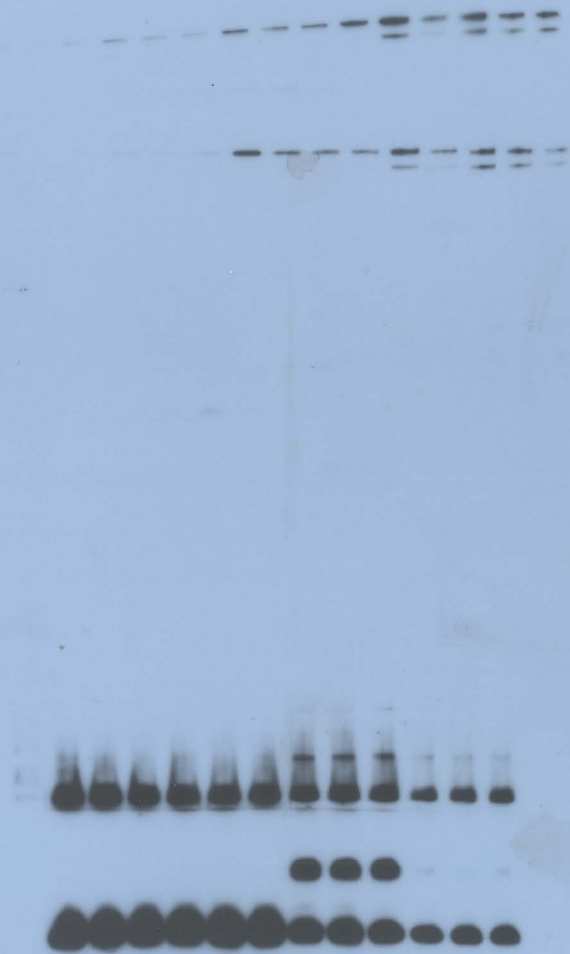

Fig.5f.  
hnRNP R(In)

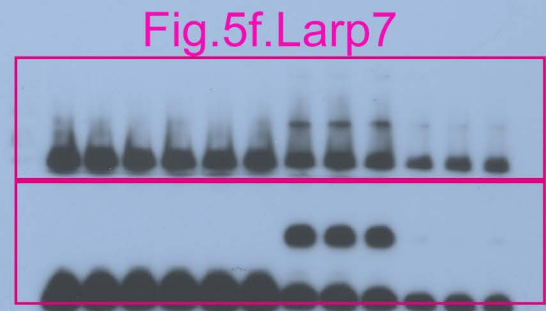

Fig.5f.Larp7

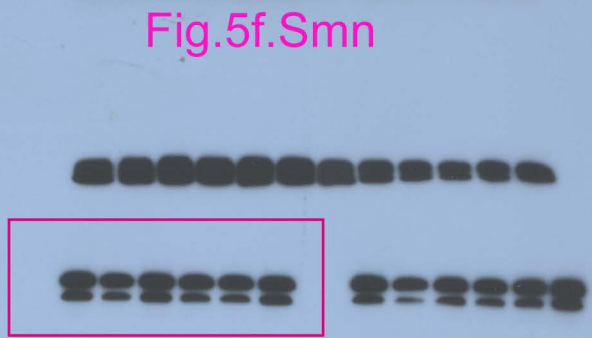

Fig.5f.Smn

Supplement: Supplementary file 8 — Source Data [file 41467_2021_21529_MOESM8_ESM.zip › Uncropped blot and gel images/Figure5/Figure5f/Larp7_Smn_hnRNP R_hnRNP A1.pdf]

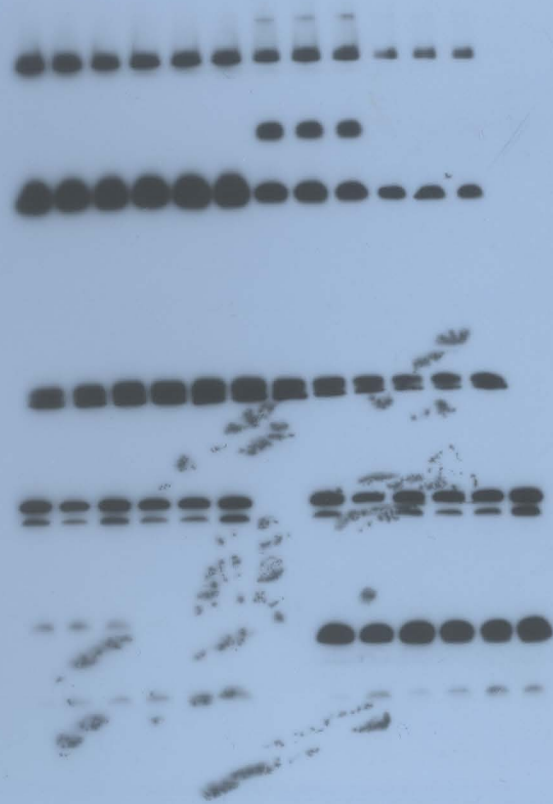

...FUJI·HRC·(SAFETY)...

SAFETY)...

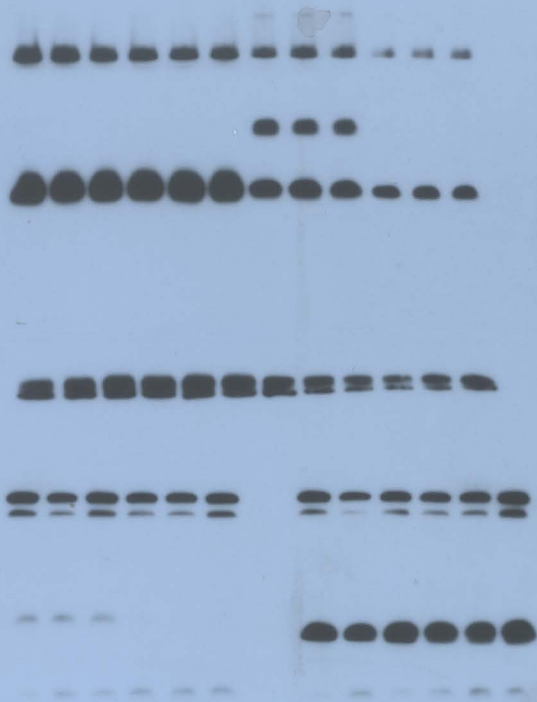

...FUJI·HRC·(SAFETY)...

TY)...

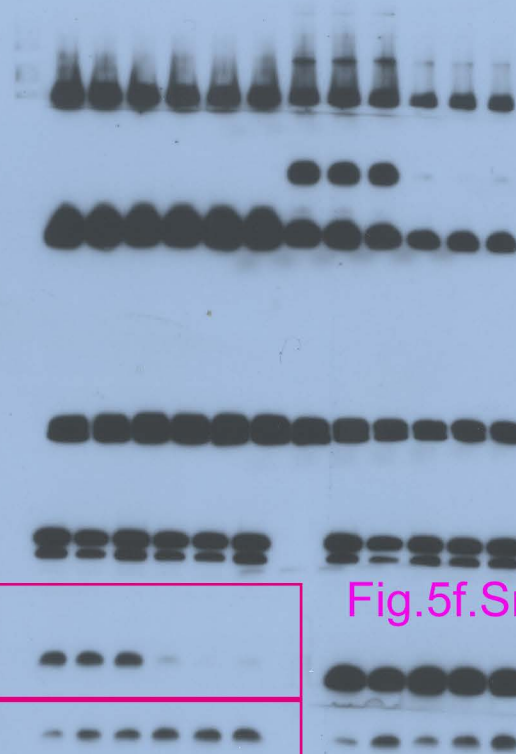

Fig.5f.Smn(In)

Fig.5f.SmB/B'(In)

Supplement: Supplementary file 8 — Source Data [file 41467_2021_21529_MOESM8_ESM.zip › Uncropped blot and gel images/Figure5/Figure5f/Smn_SmB.pdf]

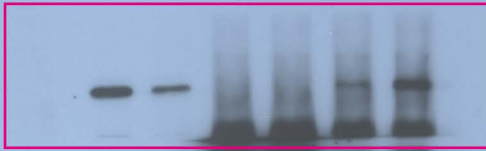

Fig.6a.Larp7

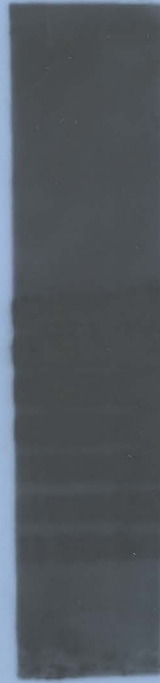

Supplement: Supplementary file 8 — Source Data [file 41467_2021_21529_MOESM8_ESM.zip › Uncropped blot and gel images/Figure6/Figure6a/Larp7.pdf]

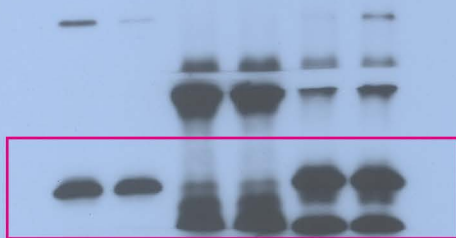

Fig.6a.Smn

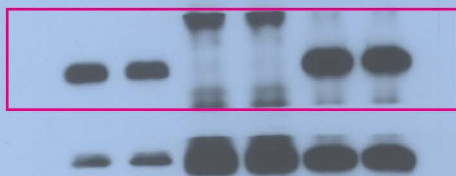

Fig.6a.Gemin2

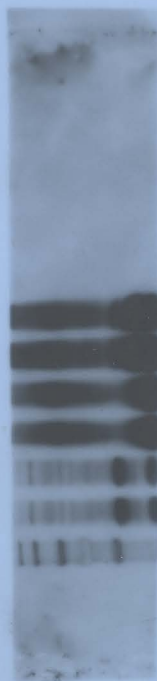

Supplement: Supplementary file 8 — Source Data [file 41467_2021_21529_MOESM8_ESM.zip › Uncropped blot and gel images/Figure6/Figure6a/Smn_Gemin2.pdf]

••FUJILHRC•(SAR

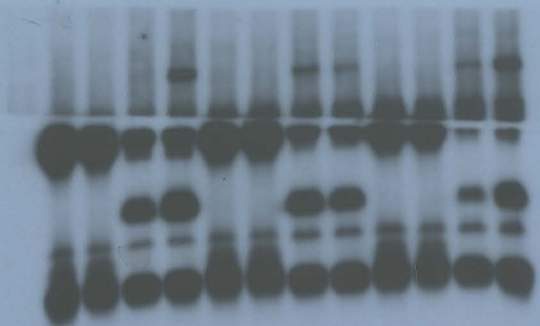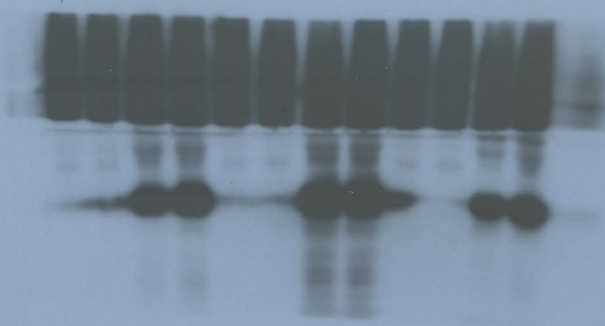

Fig.6b.Larp7

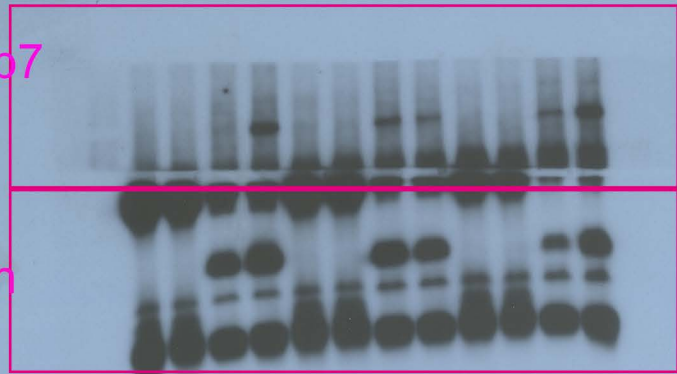

Fig.6b.Smr

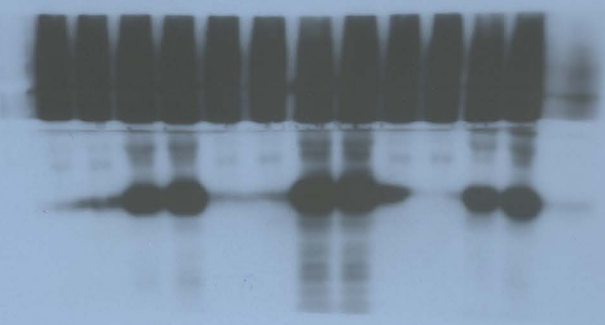

•••(YTBAA2)•CHH•II

Supplement: Supplementary file 8 — Source Data [file 41467_2021_21529_MOESM8_ESM.zip › Uncropped blot and gel images/Figure6/Figure6b/Larp7_Smn.pdf]

Fig.6e.Larp7

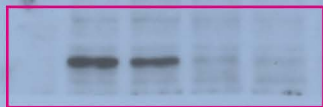

Fig.6e.Smn

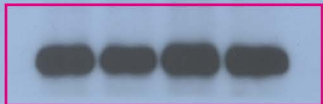

C•(2AFET1)••••

••••F0071•HBC•(2AFET1)••••

Supplement: Supplementary file 8 — Source Data [file 41467_2021_21529_MOESM8_ESM.zip › Uncropped blot and gel images/Figure6/Figure6e/Larp7_Smn.pdf]

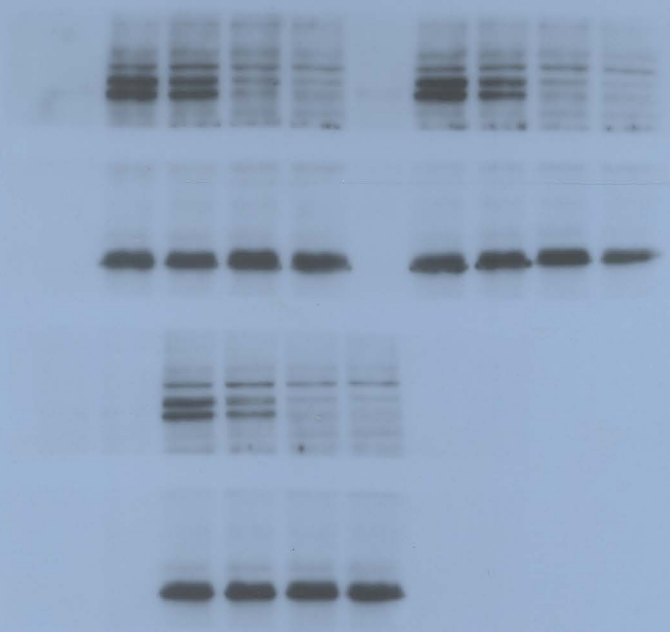

Fig.6e.Mepce

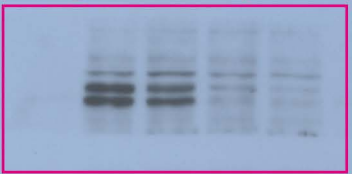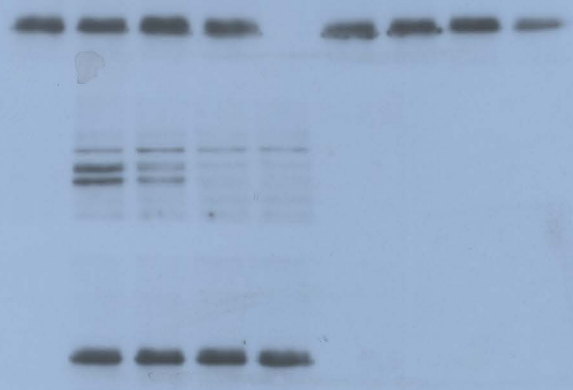

Fig.6e.Gapdh

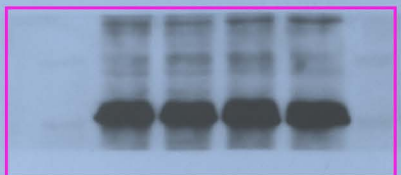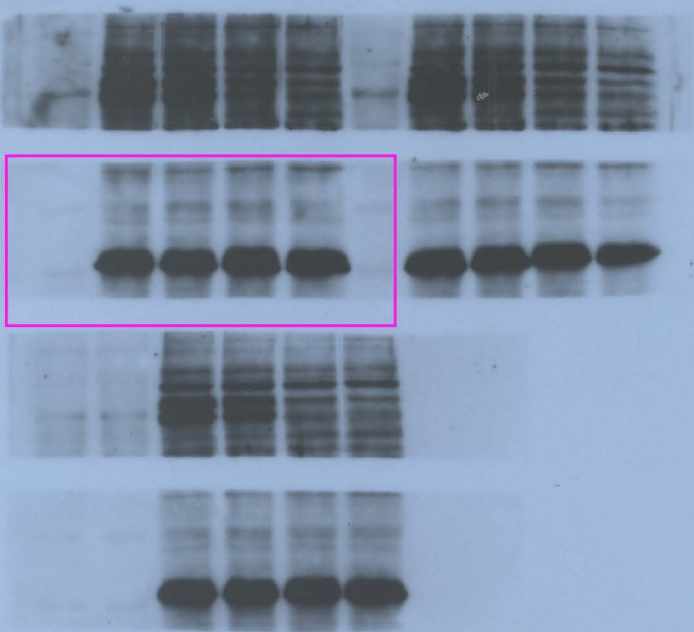

Supplement: Supplementary file 8 — Source Data [file 41467_2021_21529_MOESM8_ESM.zip › Uncropped blot and gel images/Figure6/Figure6e/Mepce.pdf]

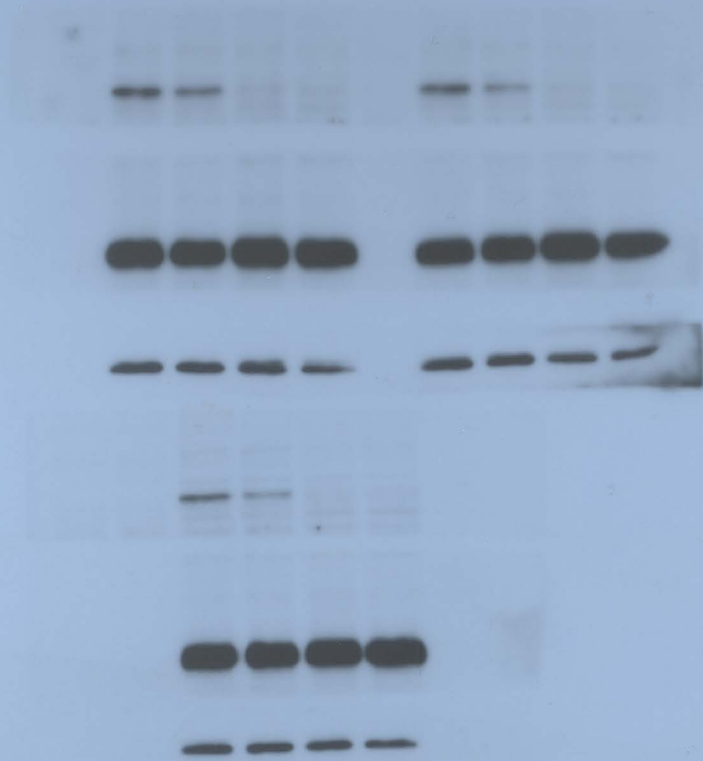

Fig.6e.  
SmB/B'

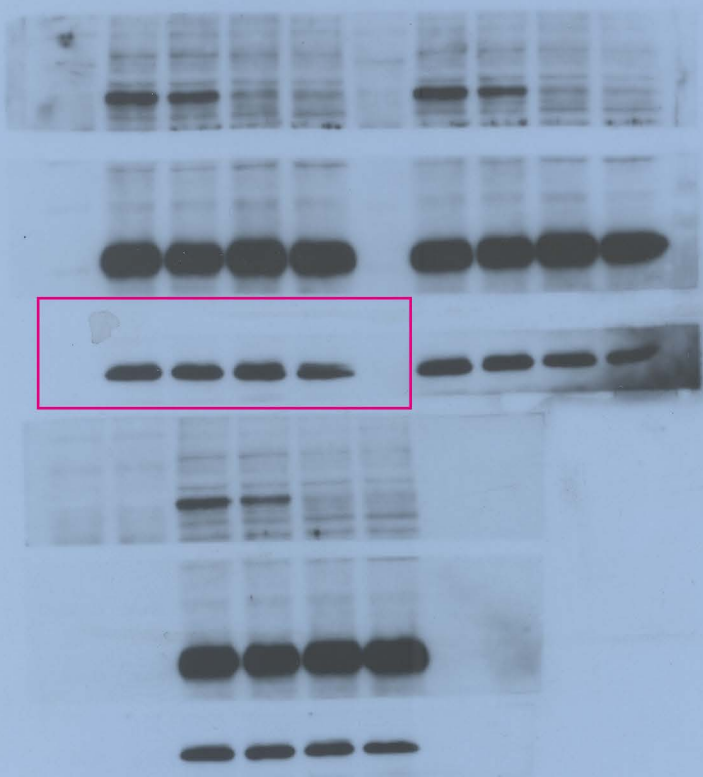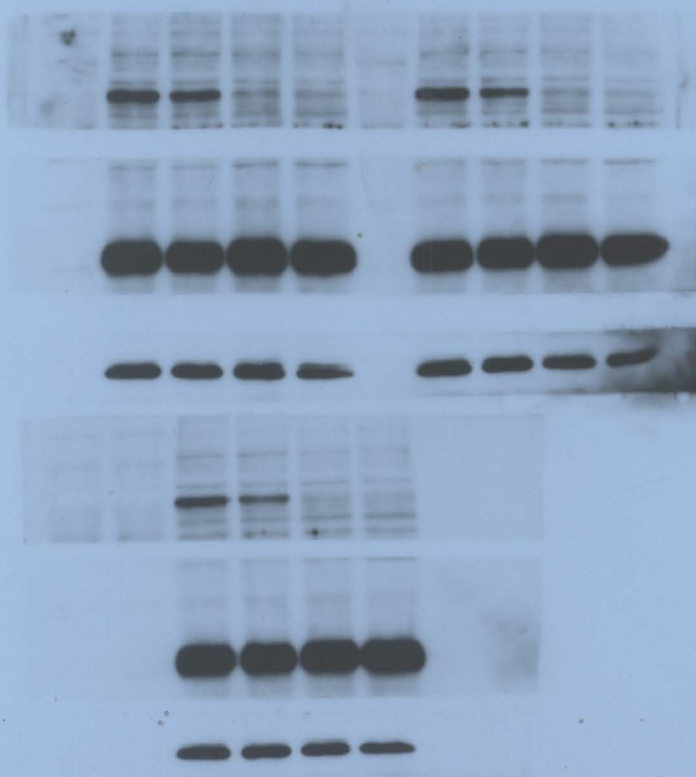

Supplement: Supplementary file 8 — Source Data [file 41467_2021_21529_MOESM8_ESM.zip › Uncropped blot and gel images/Figure6/Figure6e/SmB.pdf]

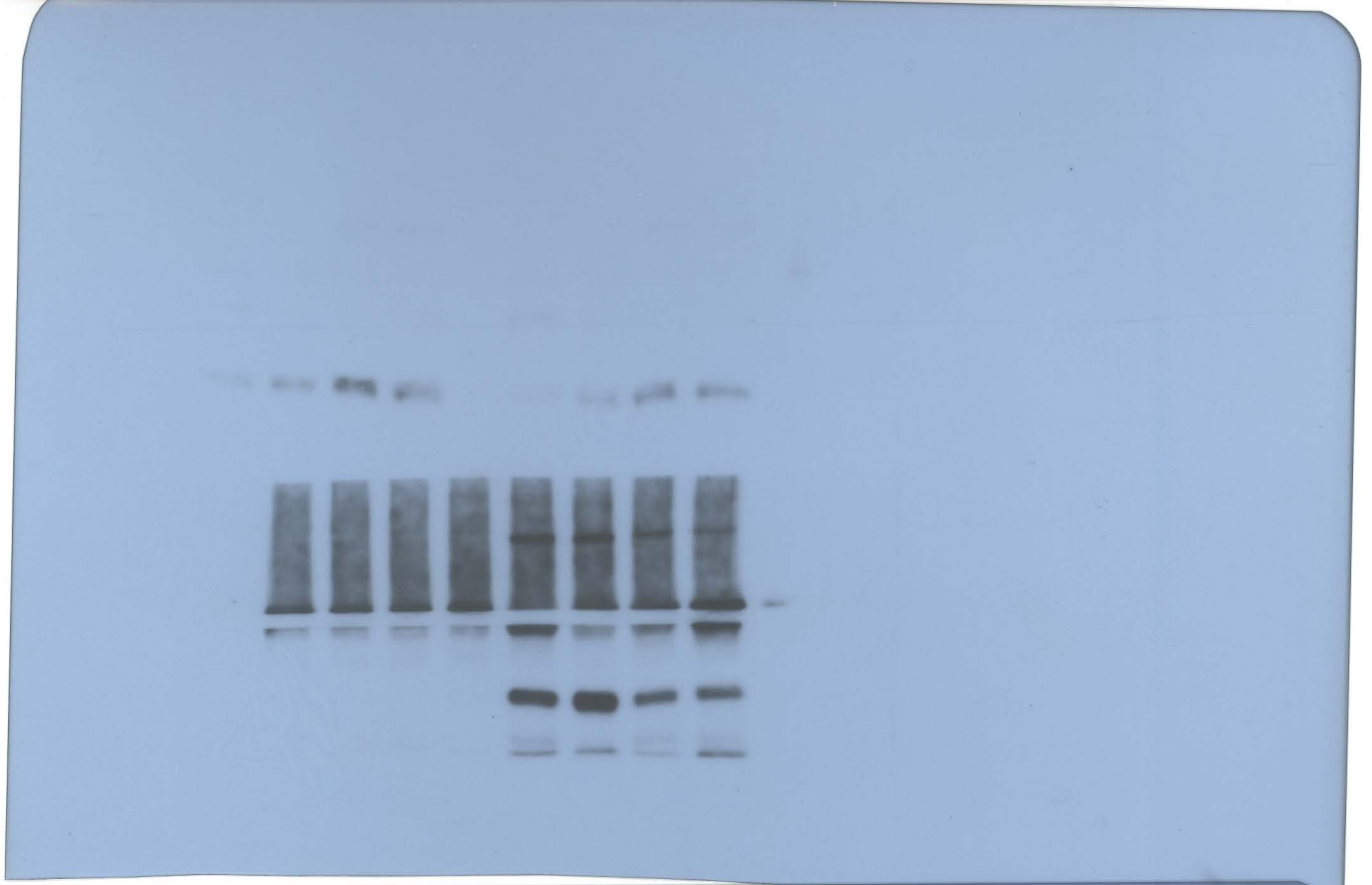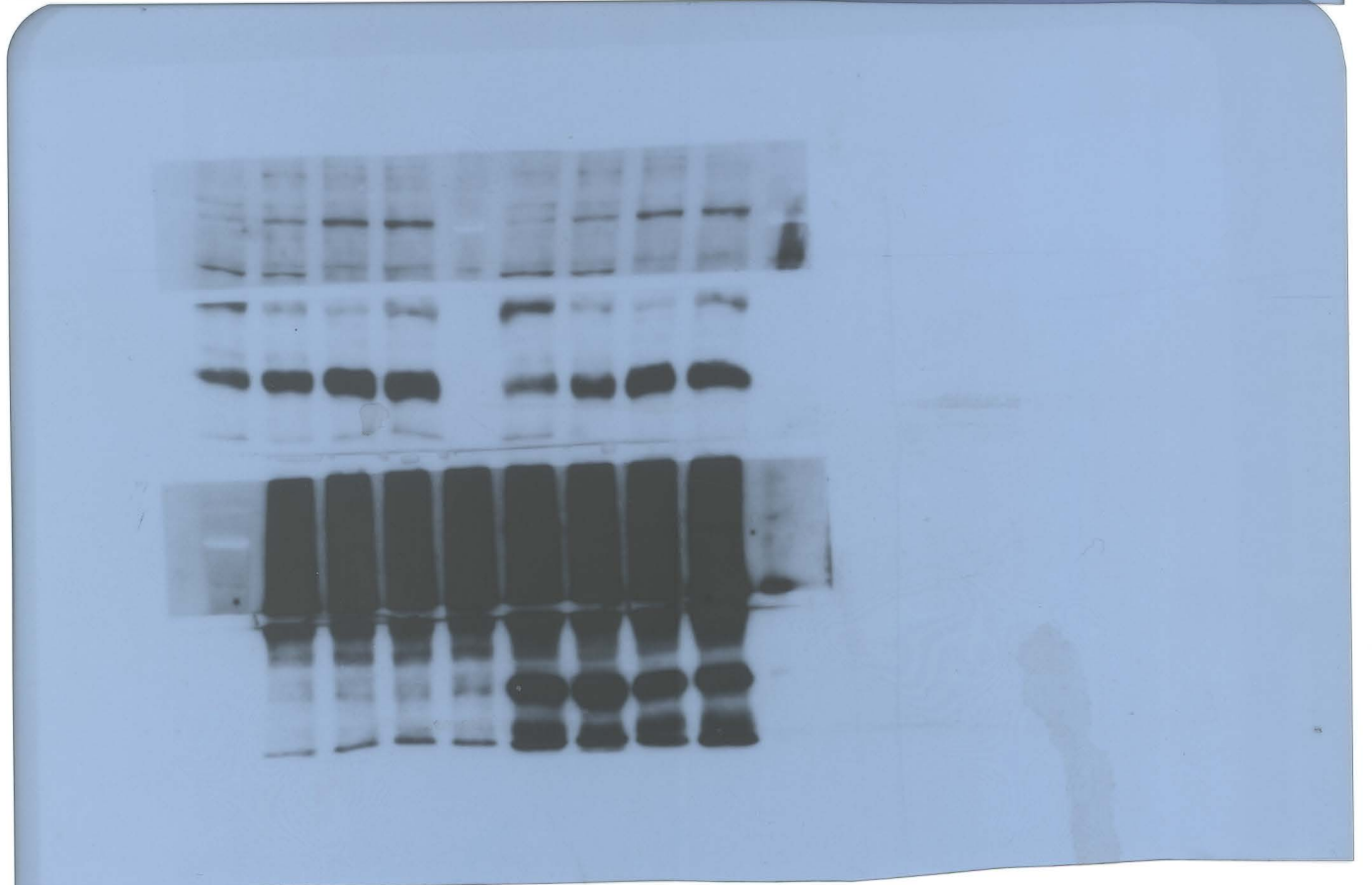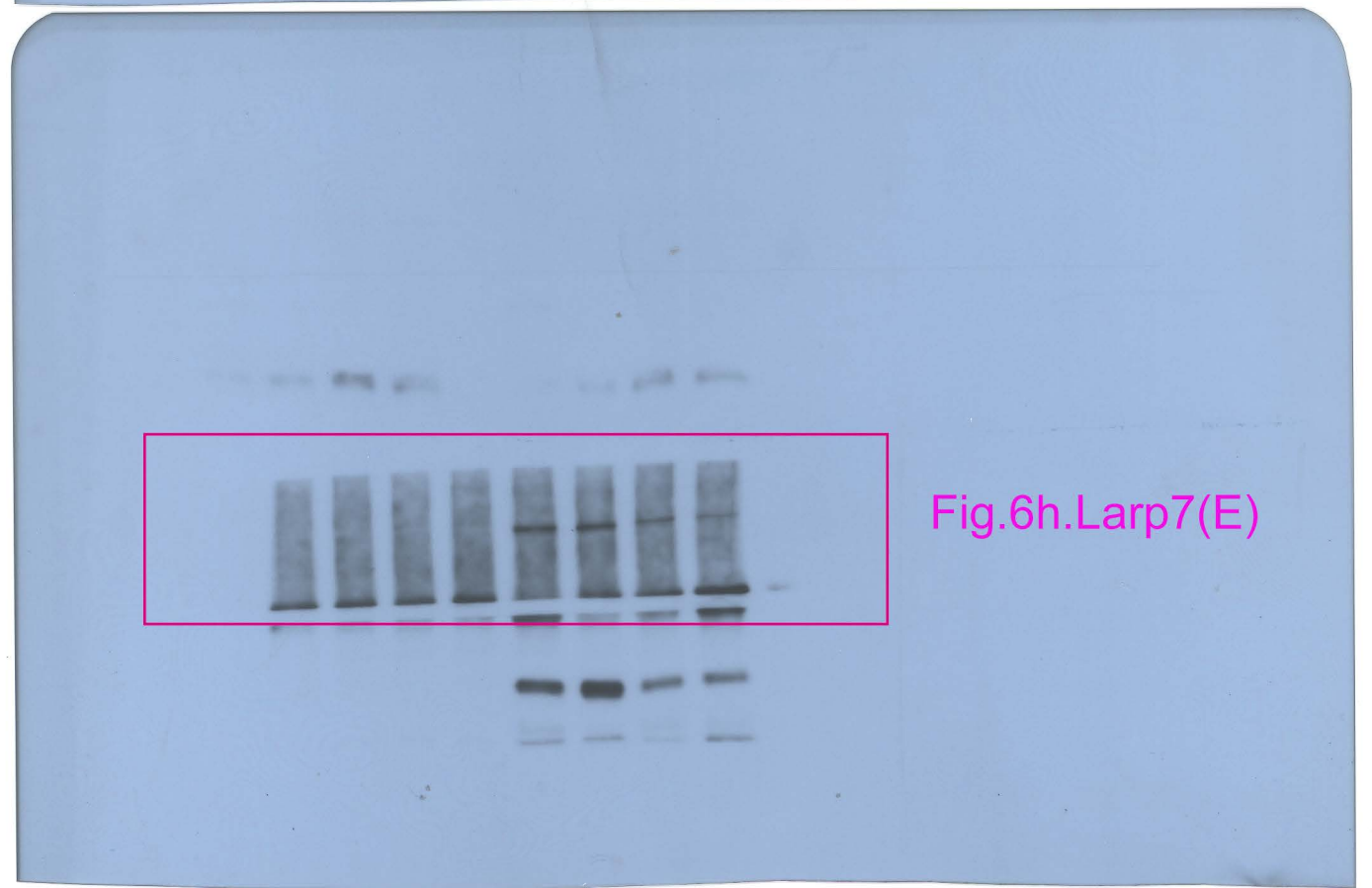

Fig.6h.Larp7(E)

Supplement: Supplementary file 8 — Source Data [file 41467_2021_21529_MOESM8_ESM.zip › Uncropped blot and gel images/Figure6/Figure6h/Larp7.pdf]

Fig.6h.Larp7(In)

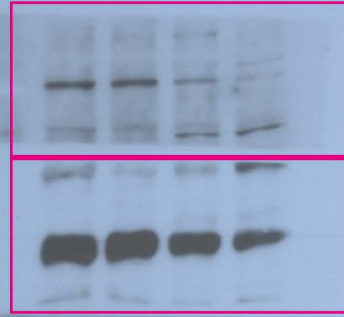

Fig 6h.Smn(In)

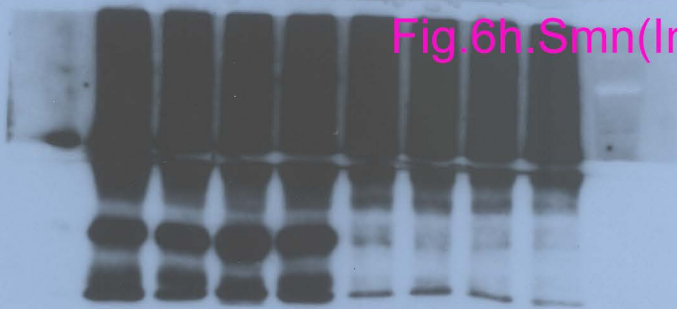

Supplement: Supplementary file 8 — Source Data [file 41467_2021_21529_MOESM8_ESM.zip › Uncropped blot and gel images/Figure6/Figure6h/Larp7_Smn.pdf]

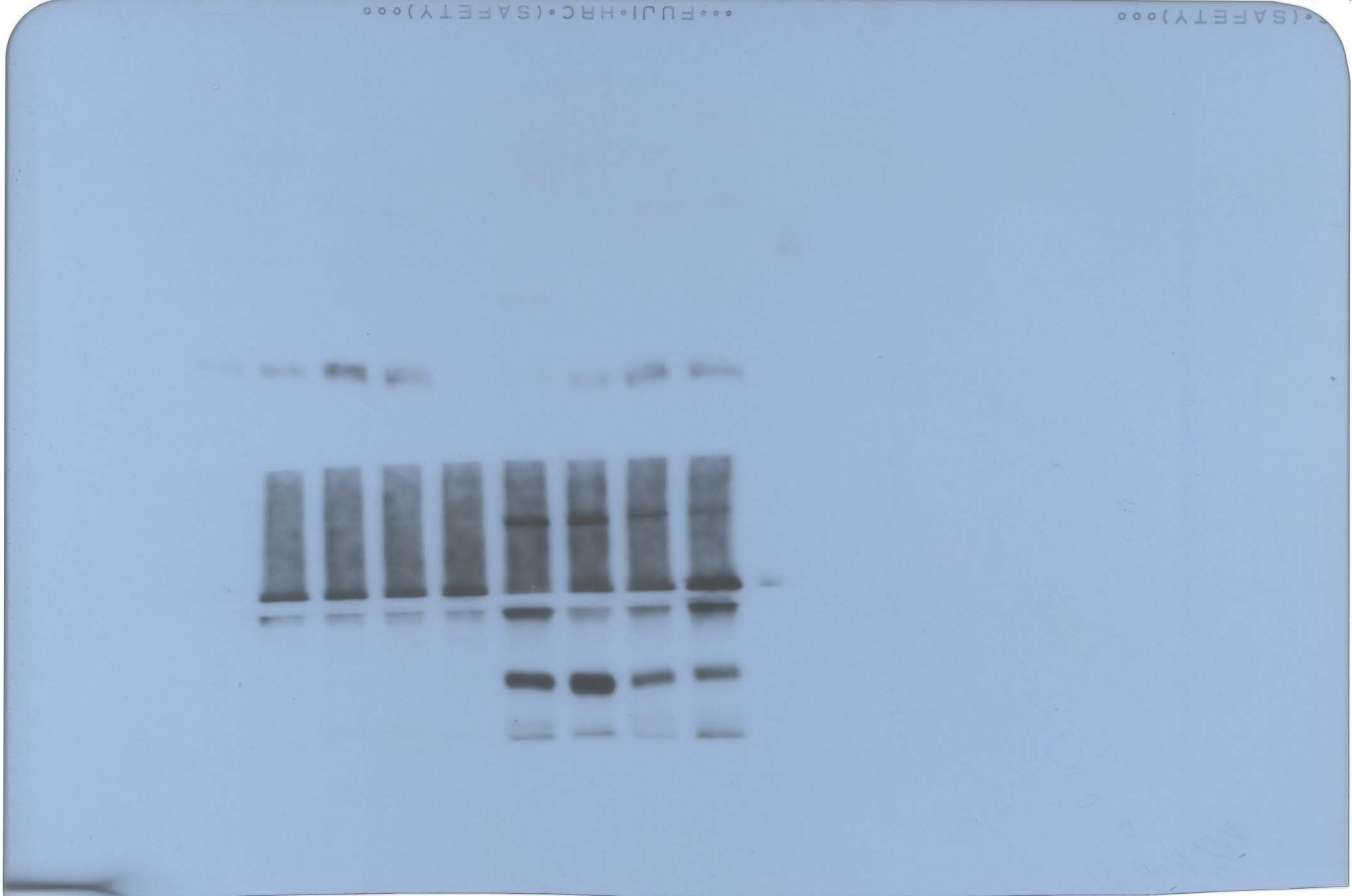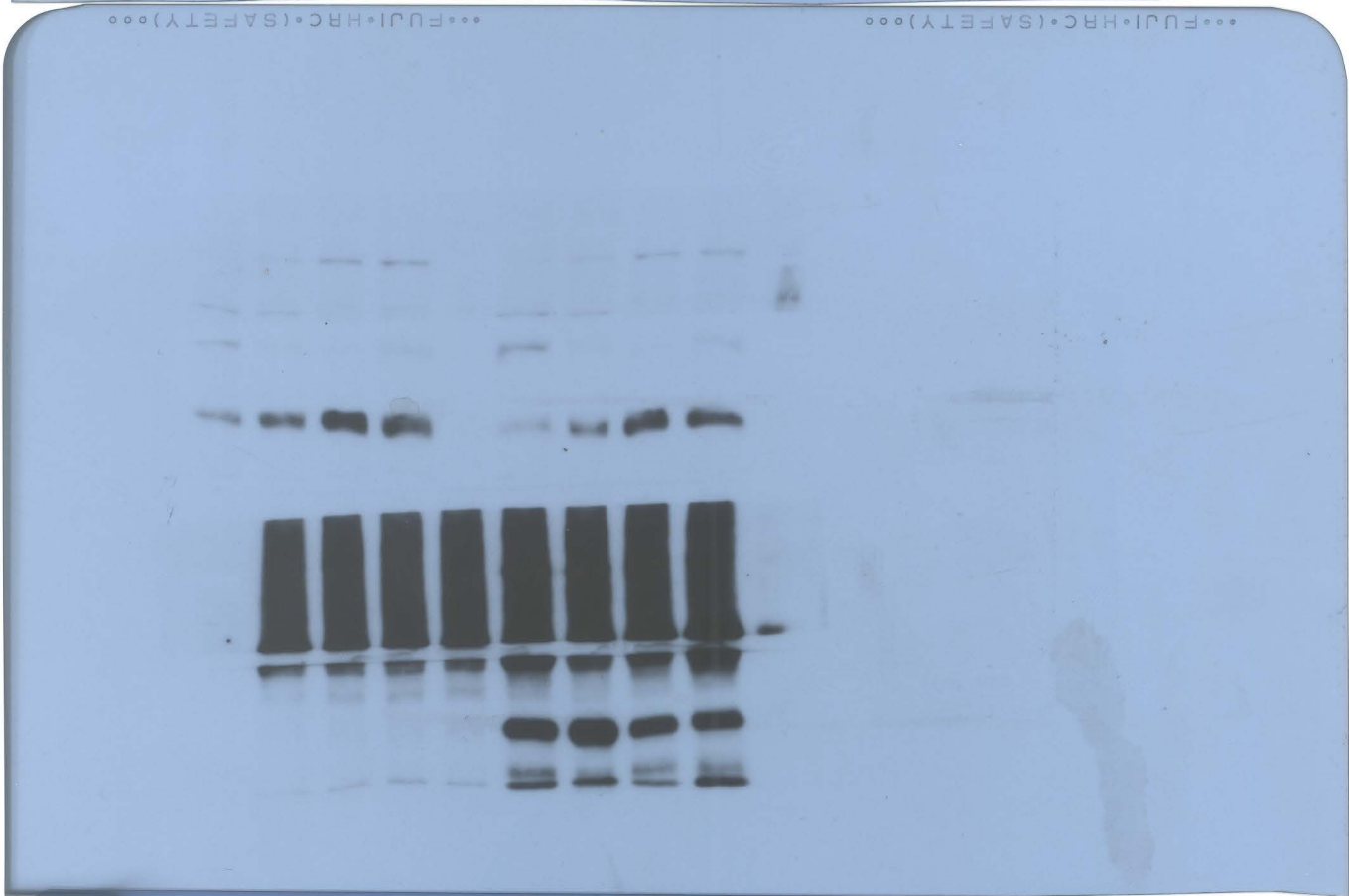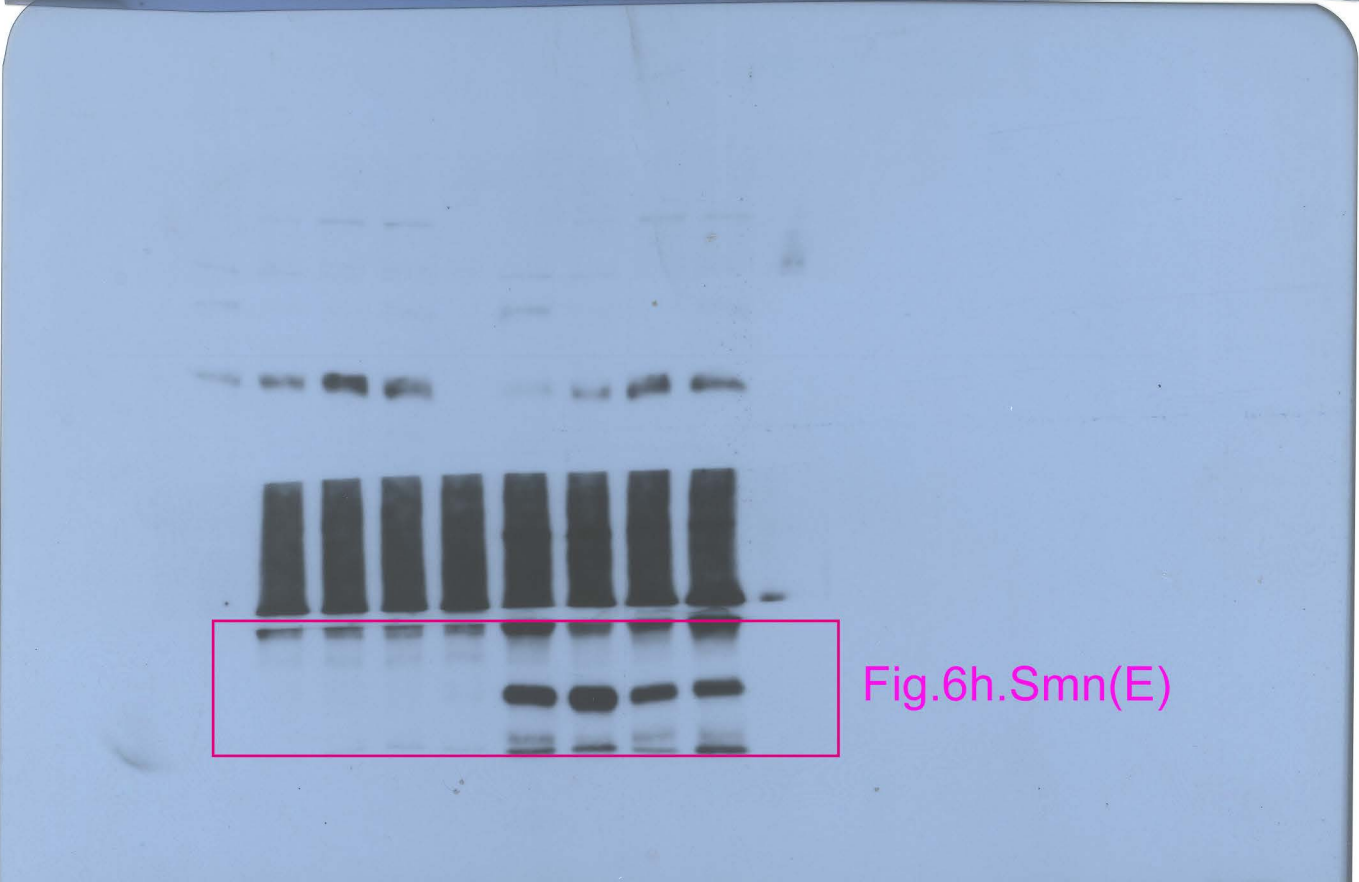

Fig.6h.Smn(E)

Supplement: Supplementary file 8 — Source Data [file 41467_2021_21529_MOESM8_ESM.zip › Uncropped blot and gel images/Figure6/Figure6h/Smn.pdf]

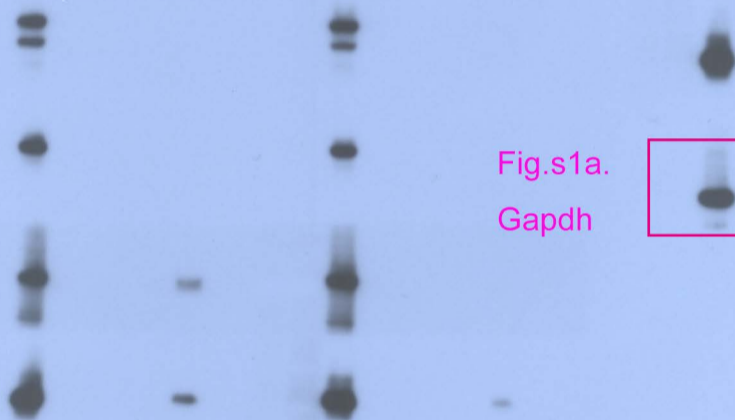

Fig.s1a.  
Gapdh

Supplement: Supplementary file 8 — Source Data [file 41467_2021_21529_MOESM8_ESM.zip › Uncropped blot and gel images/FigureS1/FigureS1a/Gapdh.pdf]

Fig.s1a.  
Hexim1

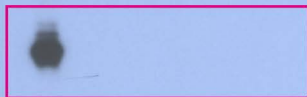

Supplement: Supplementary file 8 — Source Data [file 41467_2021_21529_MOESM8_ESM.zip › Uncropped blot and gel images/FigureS1/FigureS1a/Hexim1.pdf]

Fig.s1a.

hnNRP A1

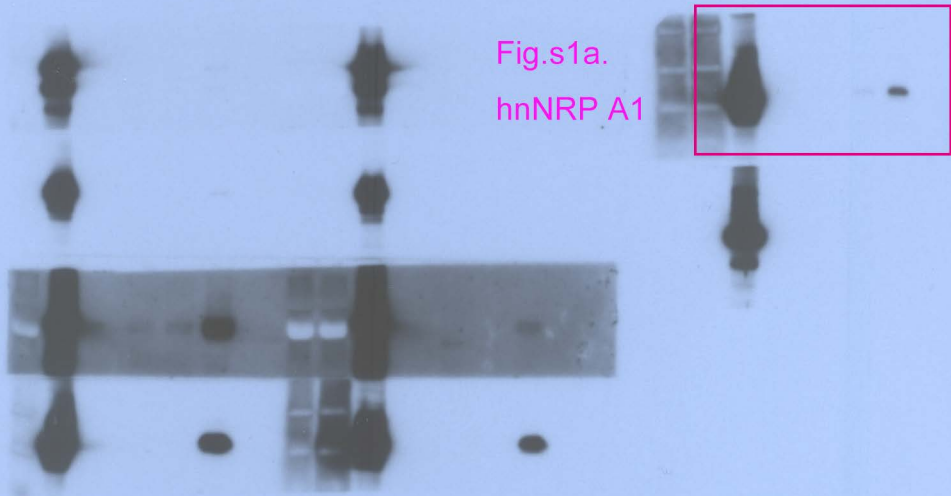

Supplement: Supplementary file 8 — Source Data [file 41467_2021_21529_MOESM8_ESM.zip › Uncropped blot and gel images/FigureS1/FigureS1a/hnRNP A1.pdf]

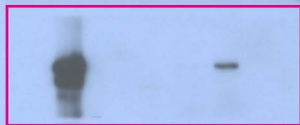

Fig.s1a.

hnRNP R

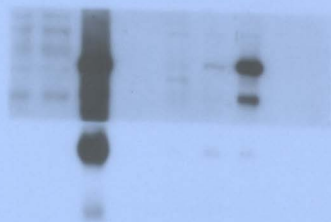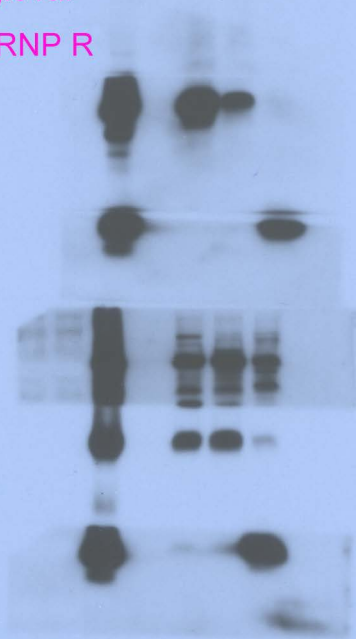

Supplement: Supplementary file 8 — Source Data [file 41467_2021_21529_MOESM8_ESM.zip › Uncropped blot and gel images/FigureS1/FigureS1a/hnRNP R.pdf]

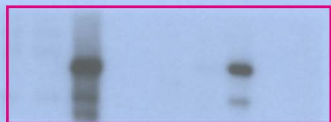

Fig.s1a.

Larp7

Supplement: Supplementary file 8 — Source Data [file 41467_2021_21529_MOESM8_ESM.zip › Uncropped blot and gel images/FigureS1/FigureS1a/Larp7.pdf]

Fig.s2b.Larp7

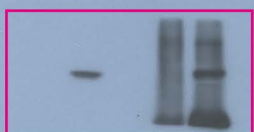

Supplement: Supplementary file 8 — Source Data [file 41467_2021_21529_MOESM8_ESM.zip › Uncropped blot and gel images/FigureS2/FigureS2b/Larp7.pdf]

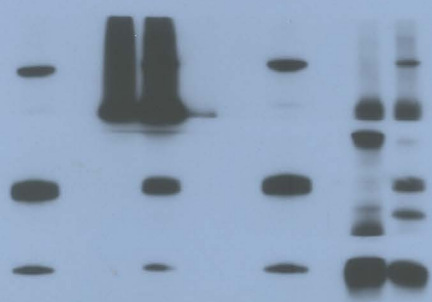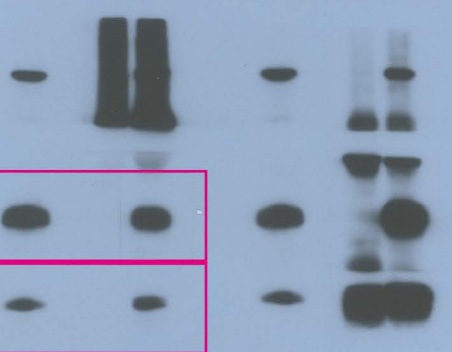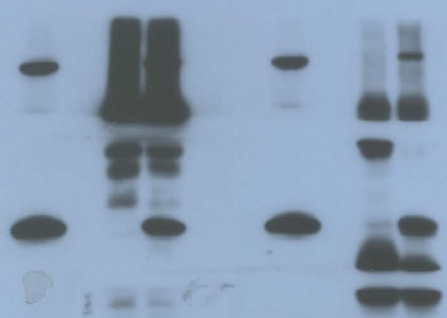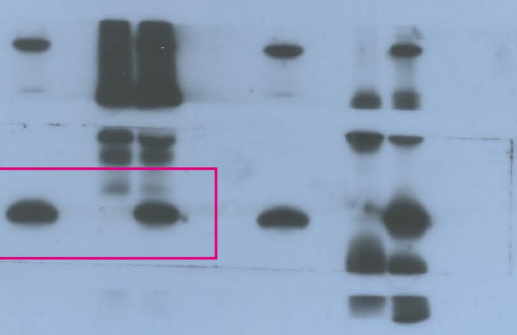

Fig.s2b.Smn

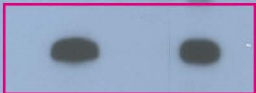

Fig.s2b.SmB/B'

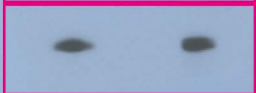

Fig.s2b.Gemin2

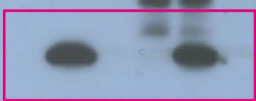

Supplement: Supplementary file 8 — Source Data [file 41467_2021_21529_MOESM8_ESM.zip › Uncropped blot and gel images/FigureS2/FigureS2b/Smn_SmB_Gemin2.pdf]

7

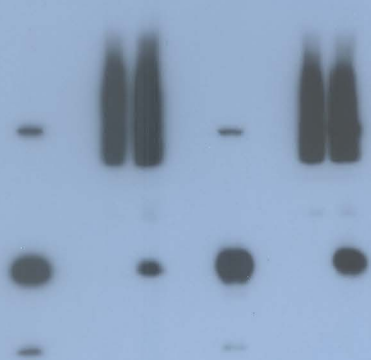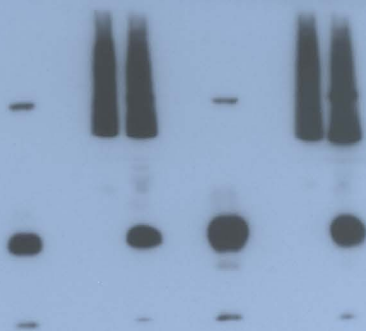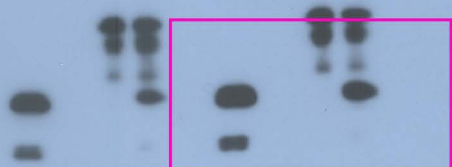

Fig.s2c.

31

GEMIN2

25

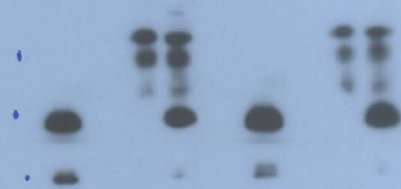

3

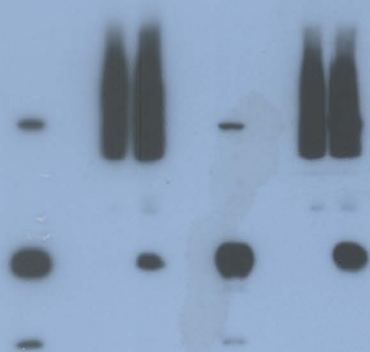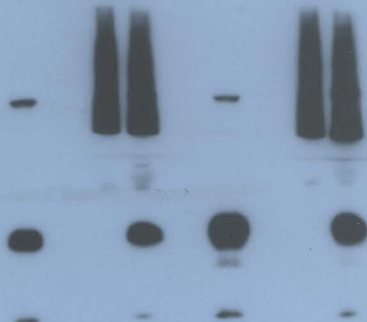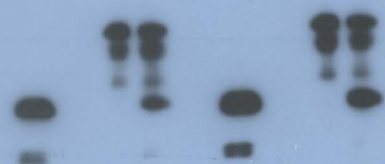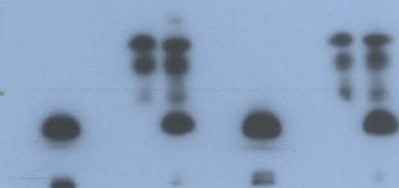

Supplement: Supplementary file 8 — Source Data [file 41467_2021_21529_MOESM8_ESM.zip › Uncropped blot and gel images/FigureS2/FigureS2c/GEMIN2.pdf]

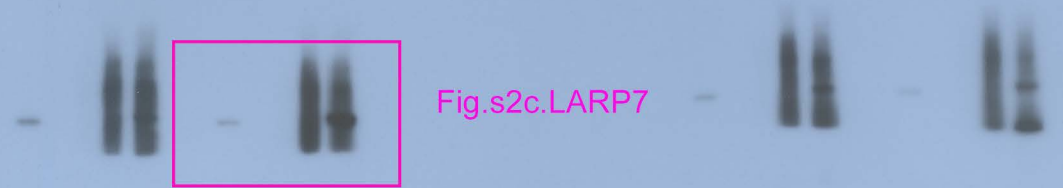

Fig.s2c.LARP7

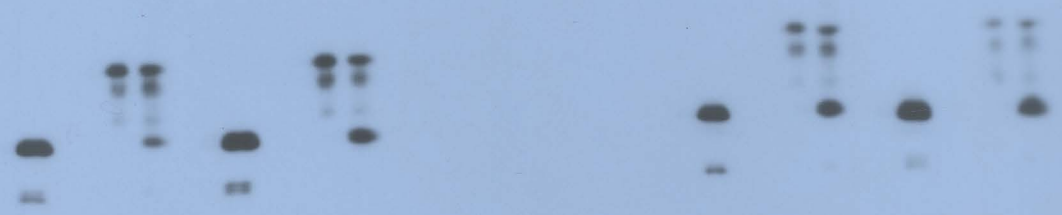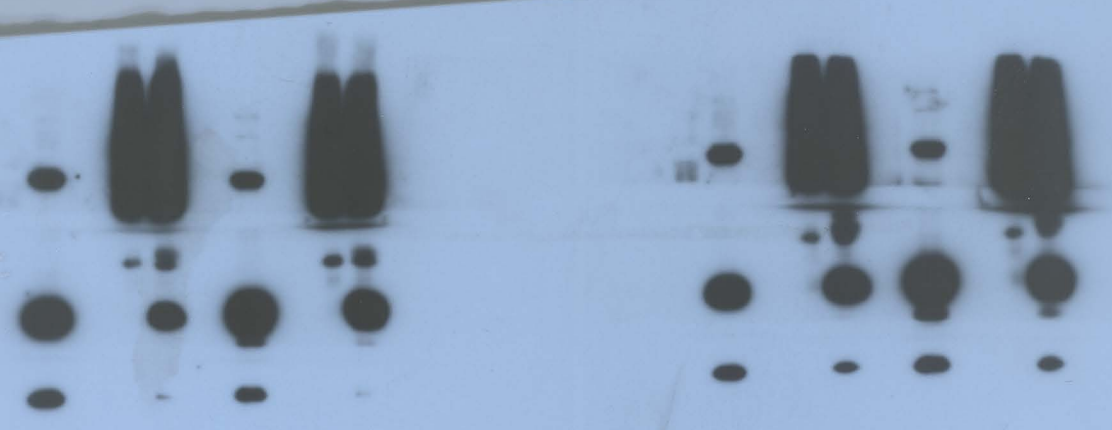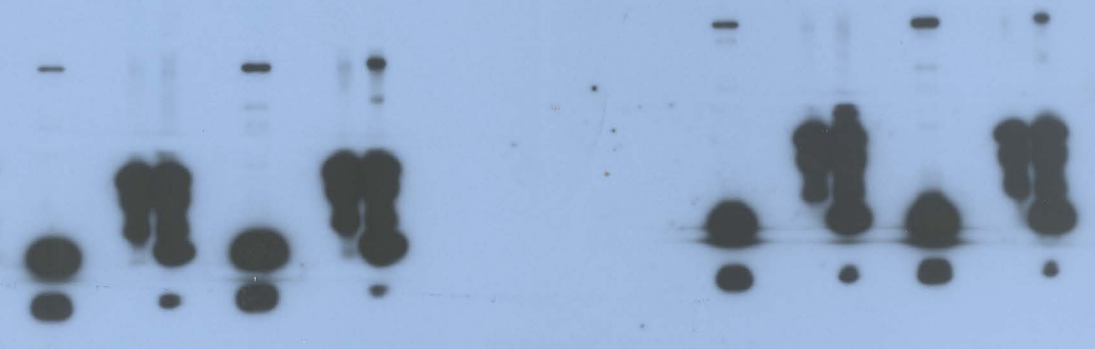

Supplement: Supplementary file 8 — Source Data [file 41467_2021_21529_MOESM8_ESM.zip › Uncropped blot and gel images/FigureS2/FigureS2c/LARP7.pdf]

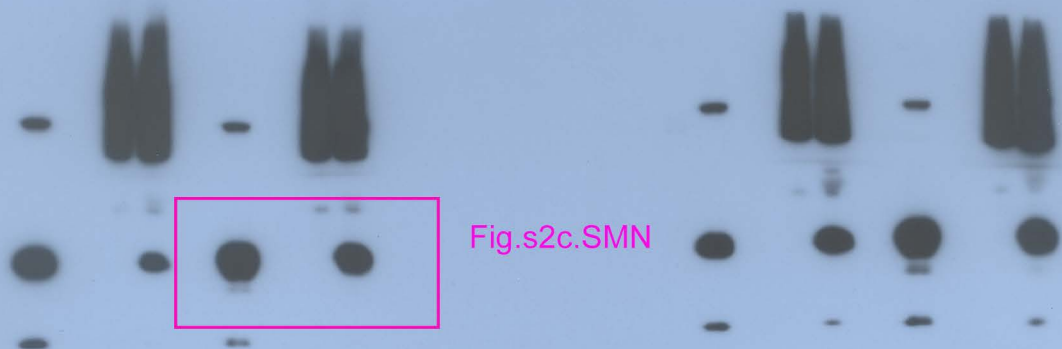

Fig.s2c.SMN

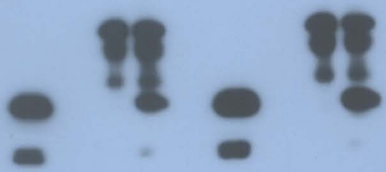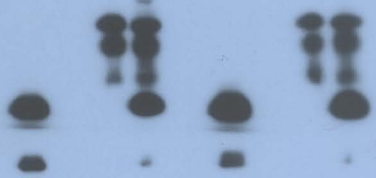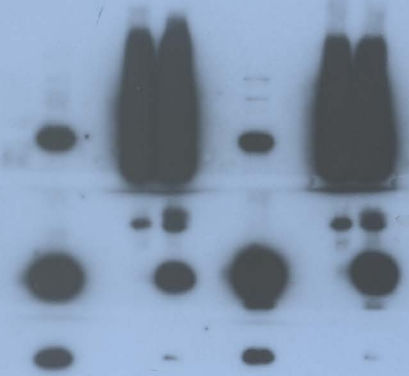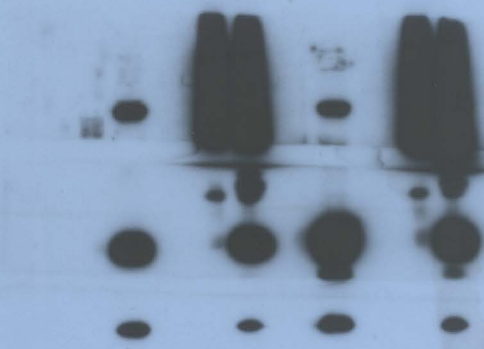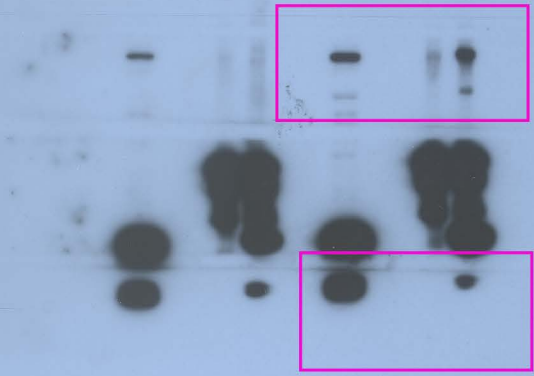

Fig.s2c.GEMIN3

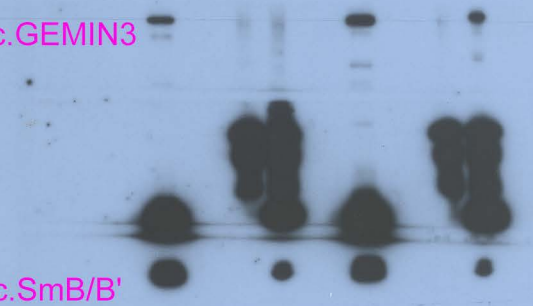

Fig.s2c.SmB/B'

Supplement: Supplementary file 8 — Source Data [file 41467_2021_21529_MOESM8_ESM.zip › Uncropped blot and gel images/FigureS2/FigureS2c/SMN_GEMIN3_SmB.pdf]

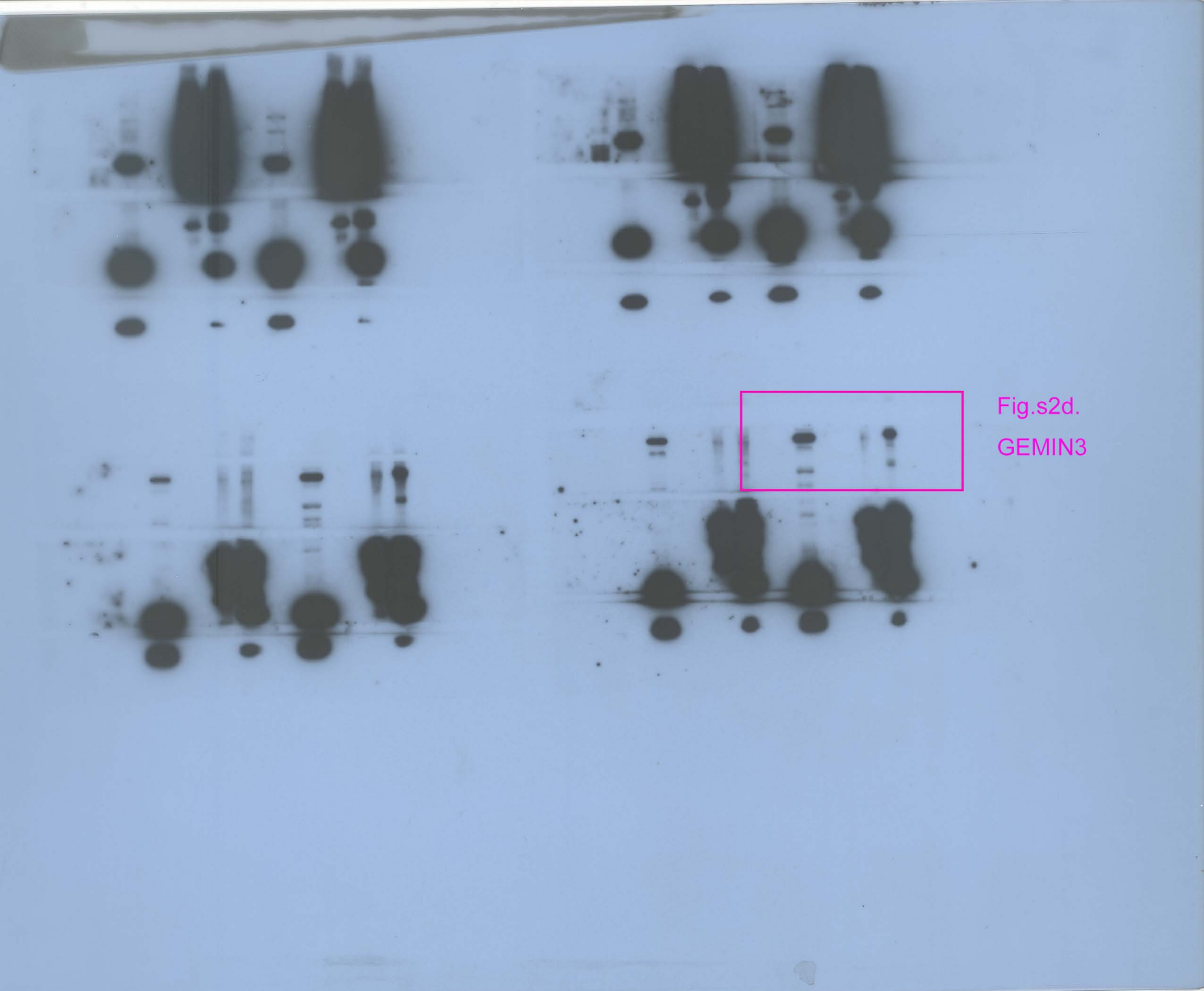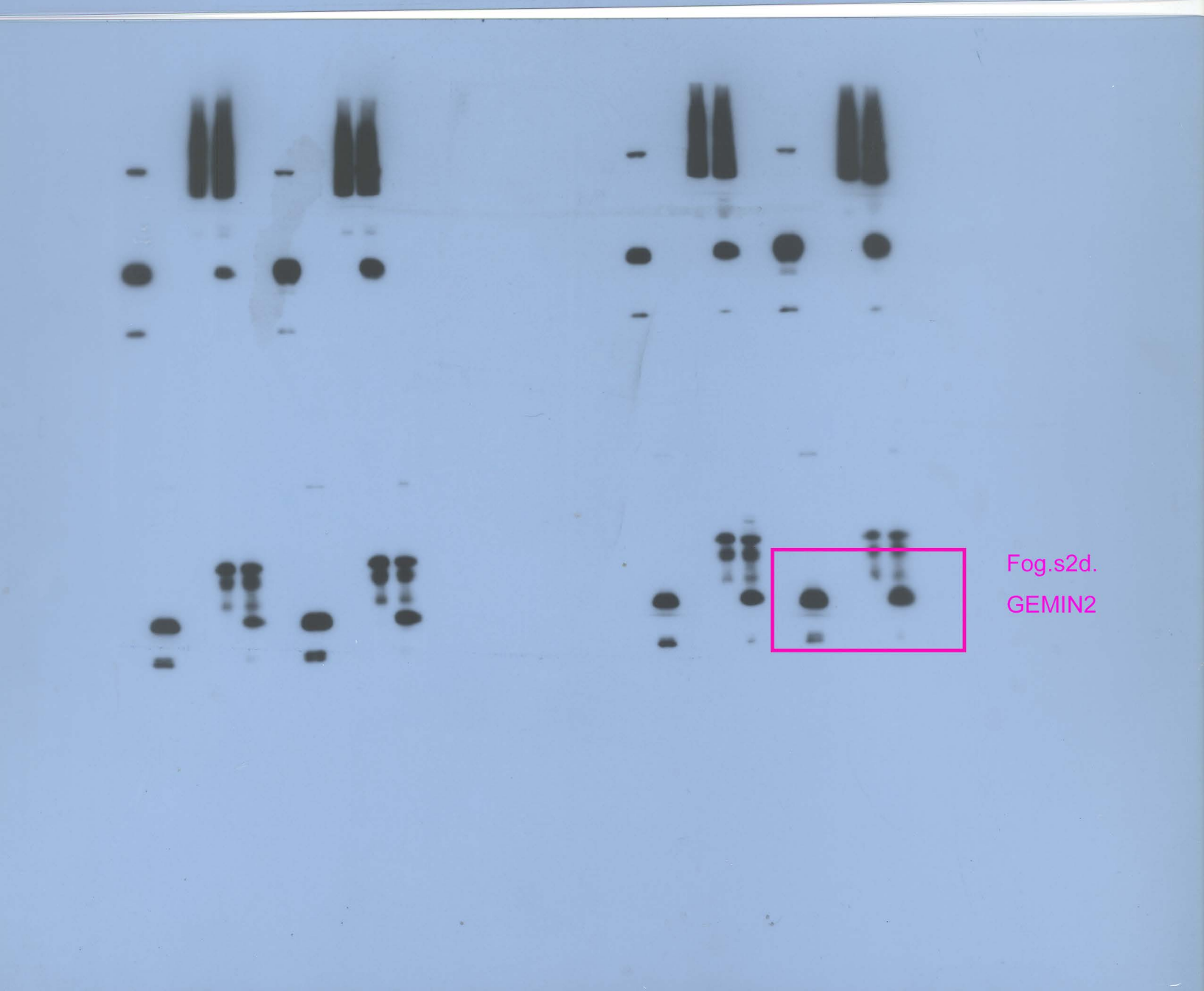

Supplement: Supplementary file 8 — Source Data [file 41467_2021_21529_MOESM8_ESM.zip › Uncropped blot and gel images/FigureS2/FigureS2d/GEMIN2_GEMIN3.pdf]

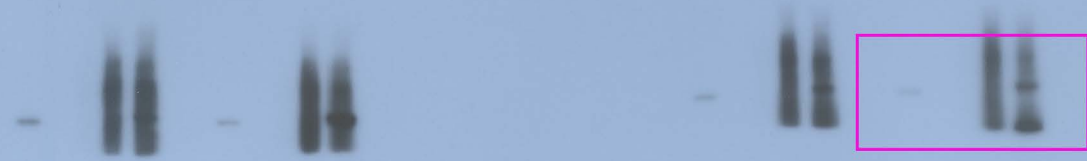

Fig.s2d.  
LARP7

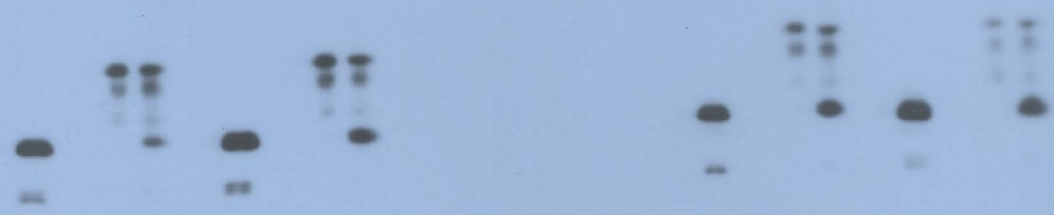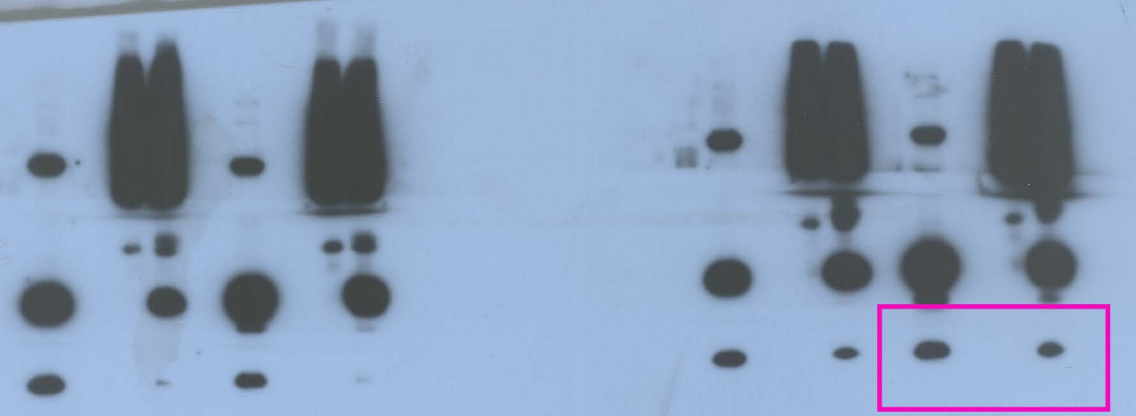

Fig.s2d.  
SmB/B'

Supplement: Supplementary file 8 — Source Data [file 41467_2021_21529_MOESM8_ESM.zip › Uncropped blot and gel images/FigureS2/FigureS2d/LARP7_SmB.pdf]

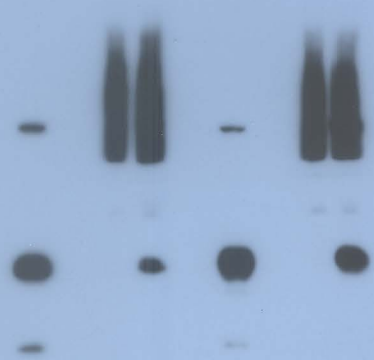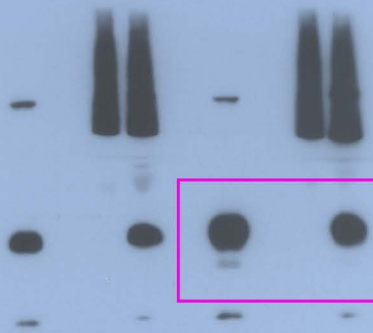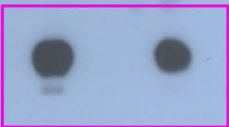

Fig.s2d.  
SMN

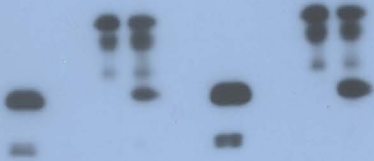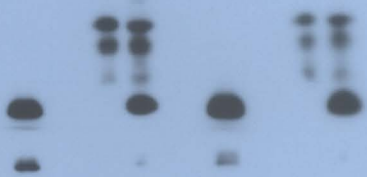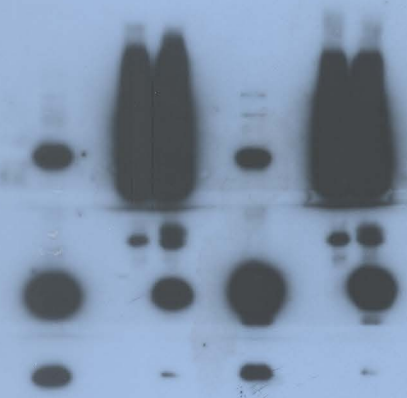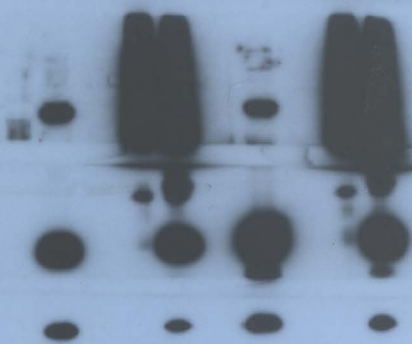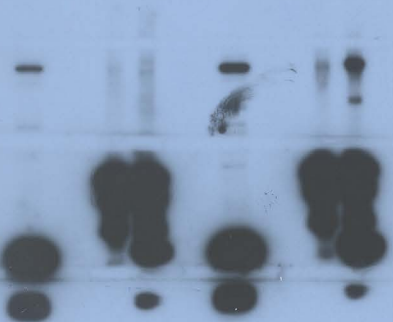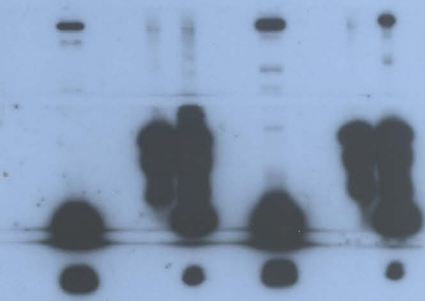

Supplement: Supplementary file 8 — Source Data [file 41467_2021_21529_MOESM8_ESM.zip › Uncropped blot and gel images/FigureS2/FigureS2d/SMN.pdf]

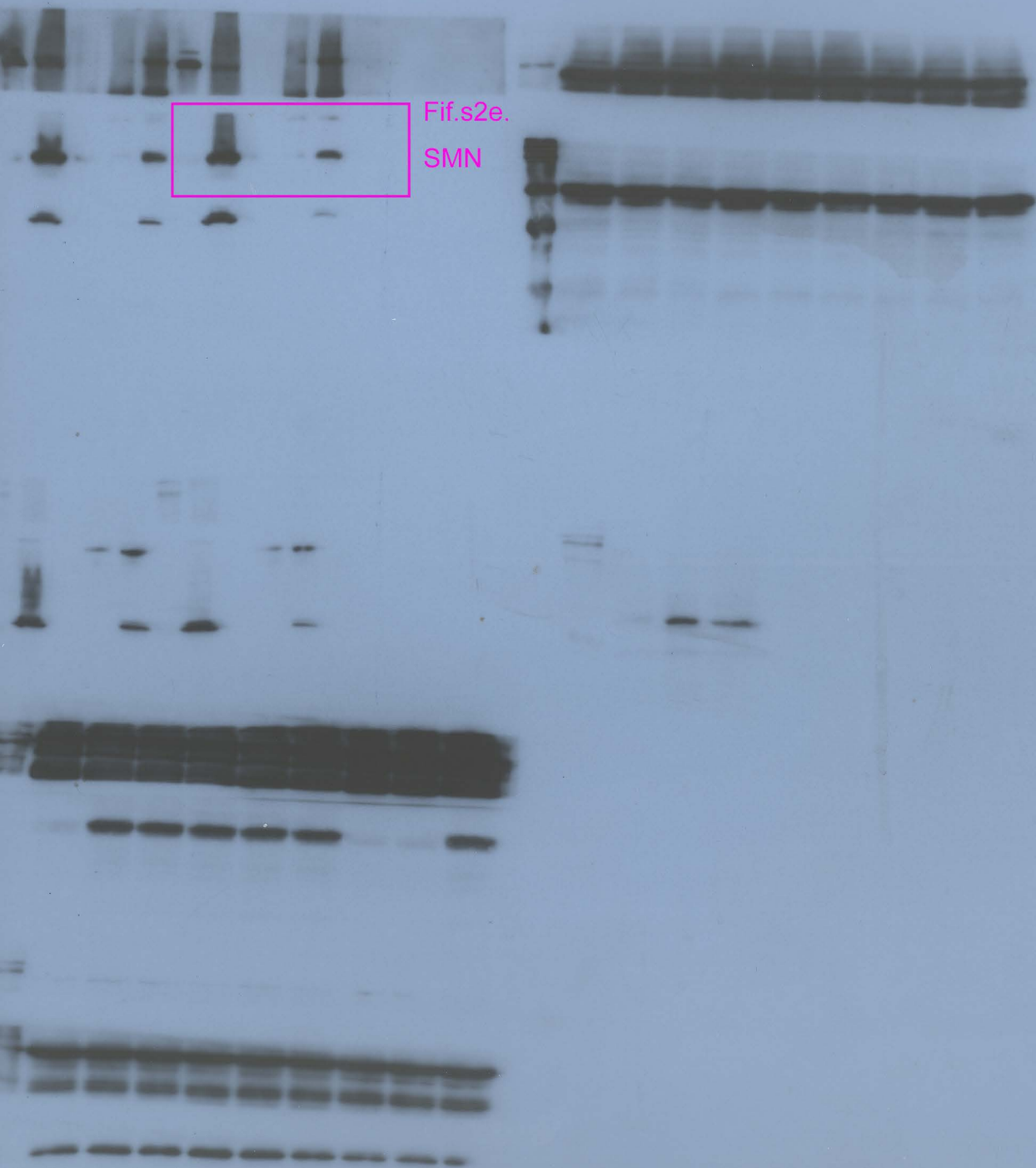

Fig.s2f.LARP7

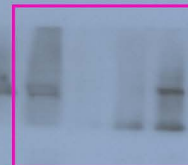

Fig.s2e.  
LARP7

-Calnexin.

35.  
28.

Fas  
SNW

PABPC1

~~SNW~~ TDP43

S-B

Supplement: Supplementary file 8 — Source Data [file 41467_2021_21529_MOESM8_ESM.zip › Uncropped blot and gel images/FigureS2/FigureS2ef/LARP7_SMN.pdf]

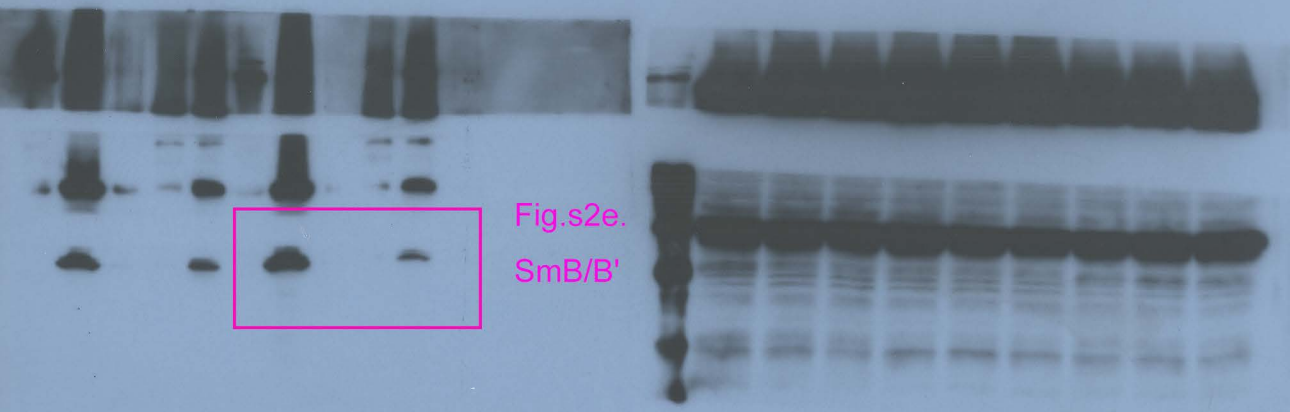

Fig.s2e.  
SmB/B'

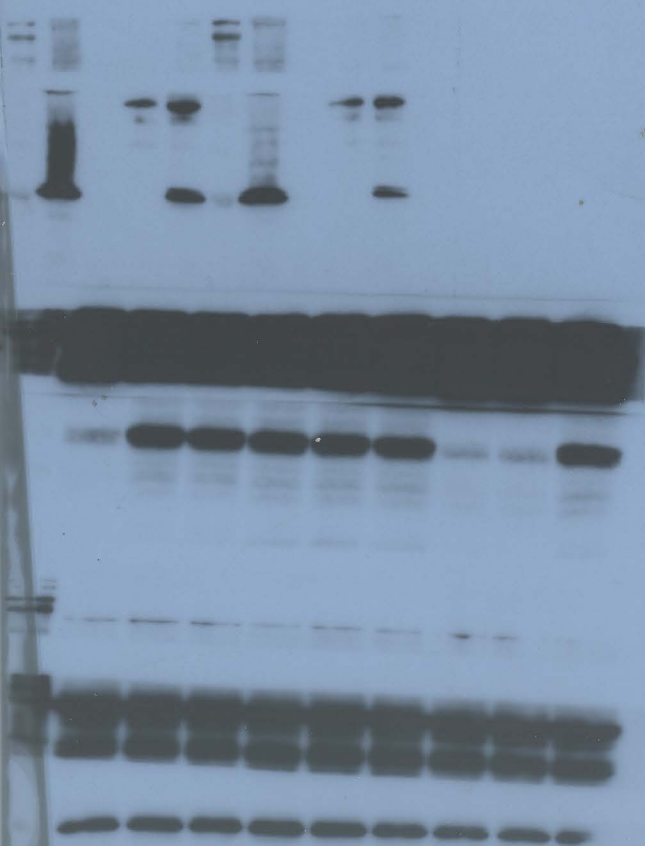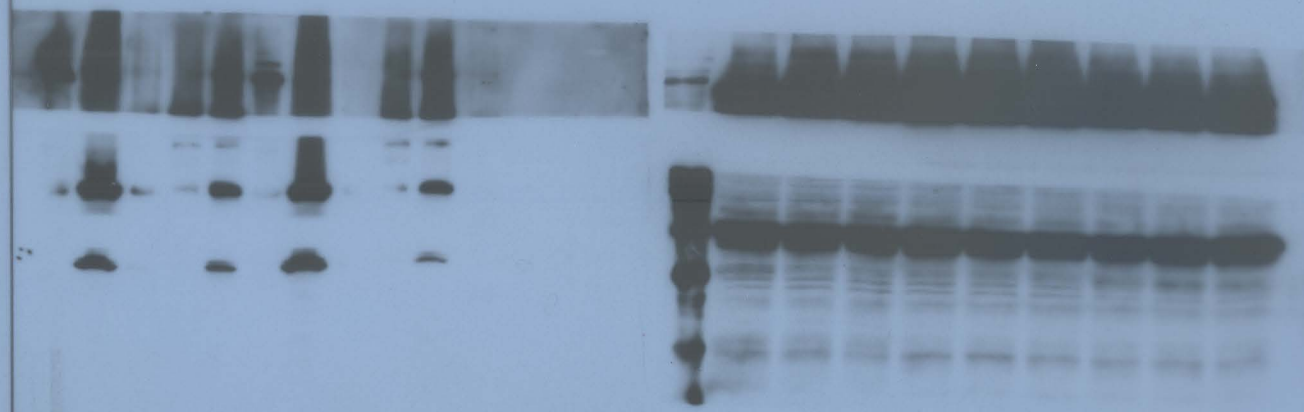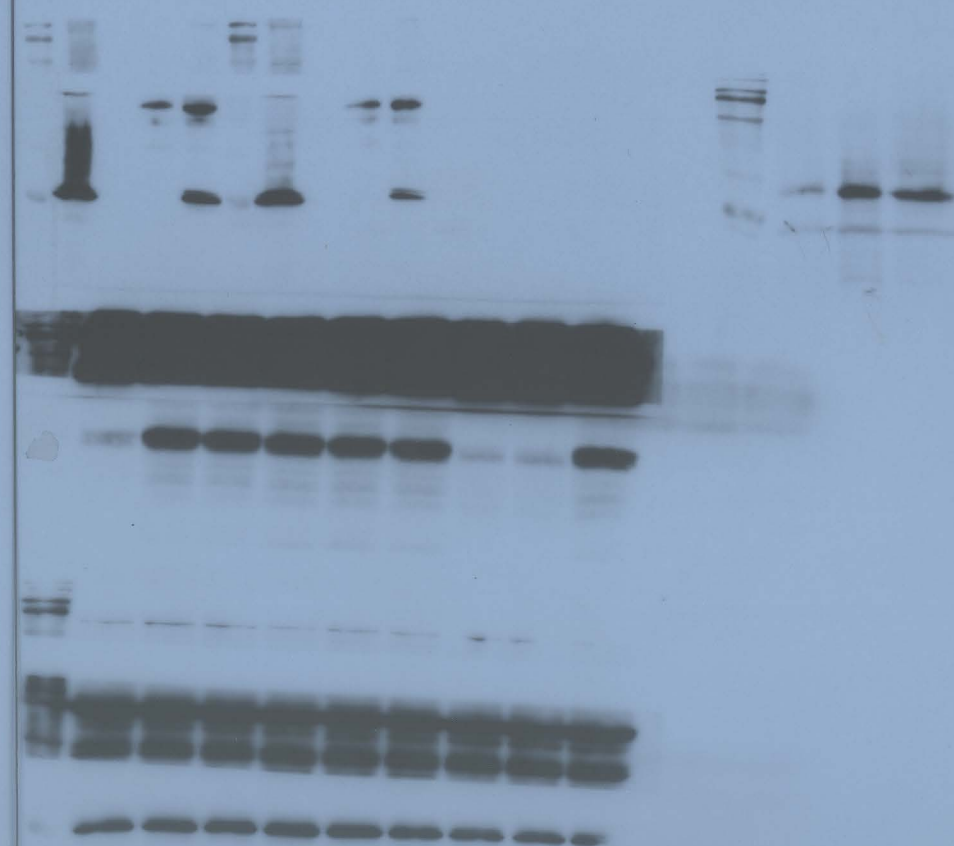

Supplement: Supplementary file 8 — Source Data [file 41467_2021_21529_MOESM8_ESM.zip › Uncropped blot and gel images/FigureS2/FigureS2ef/SmB.pdf]

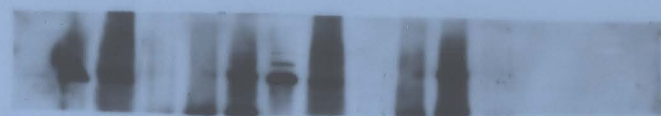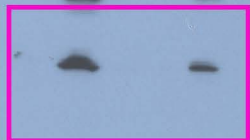

Fig.s2f.SmB/B'

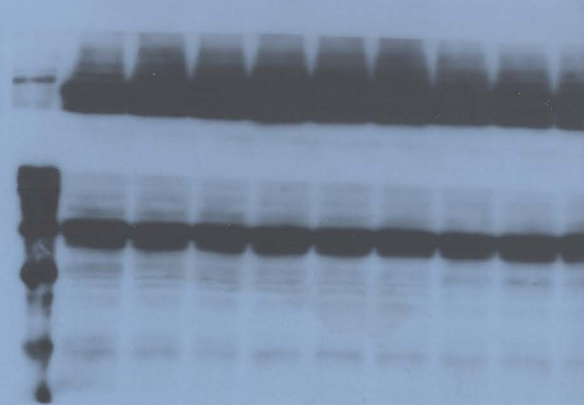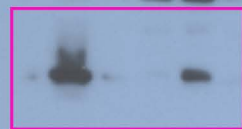

Fig.s2f.SMN

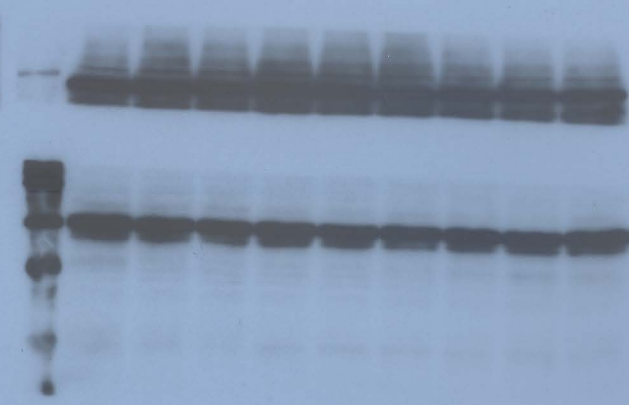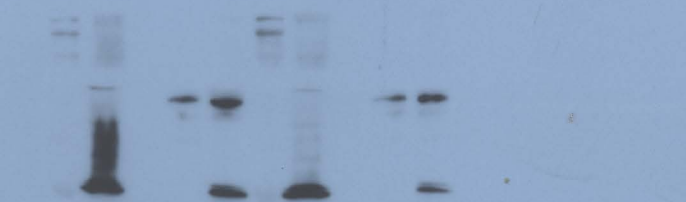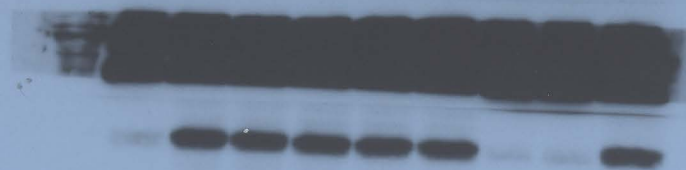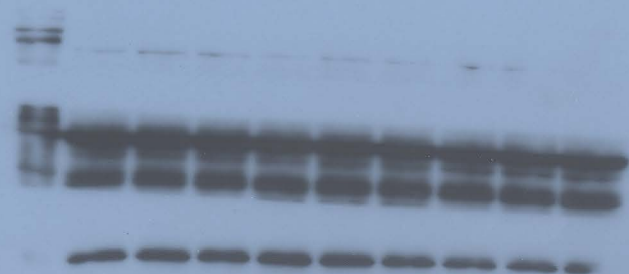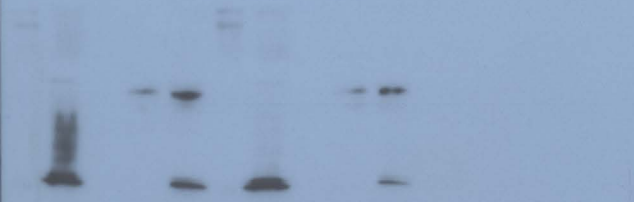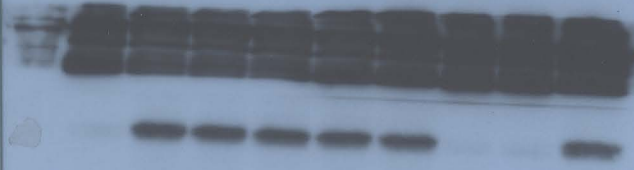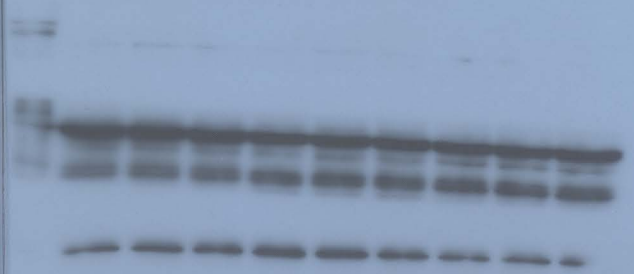

Supplement: Supplementary file 8 — Source Data [file 41467_2021_21529_MOESM8_ESM.zip › Uncropped blot and gel images/FigureS2/FigureS2ef/SMN_SmB.pdf]

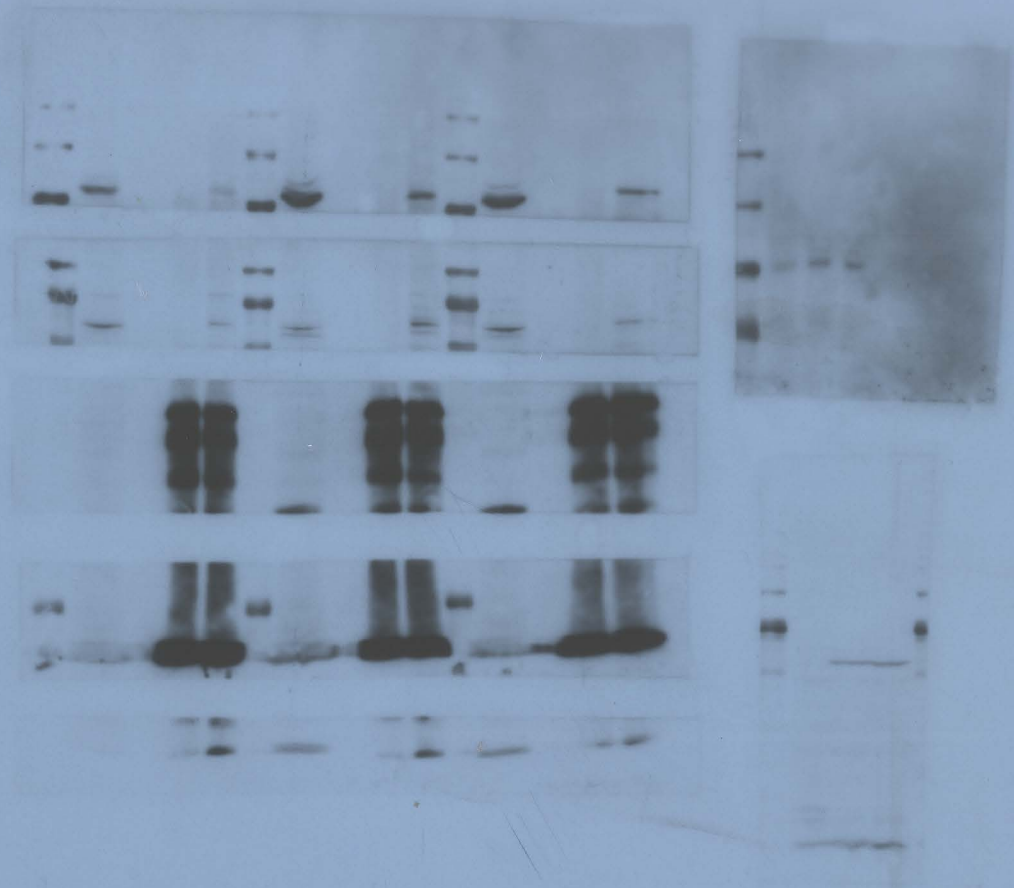

Fig.s2g.Gemin4

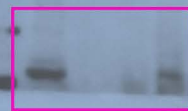

Fig.s2g.  
hnRNP Q

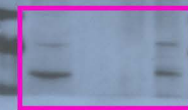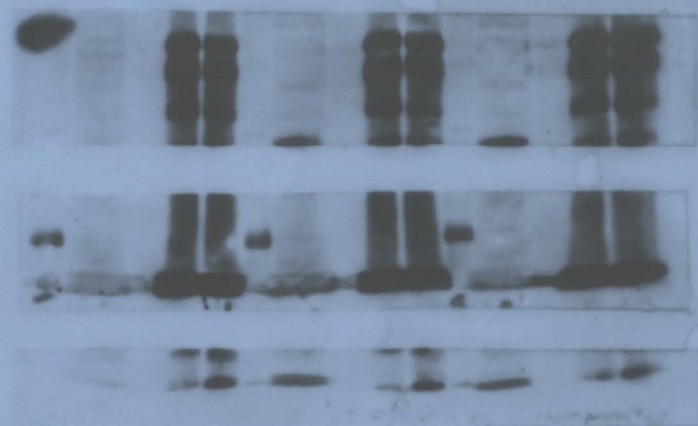

Supplement: Supplementary file 8 — Source Data [file 41467_2021_21529_MOESM8_ESM.zip › Uncropped blot and gel images/FigureS2/FigureS2g/hnRNP Q_Gemin4.pdf]

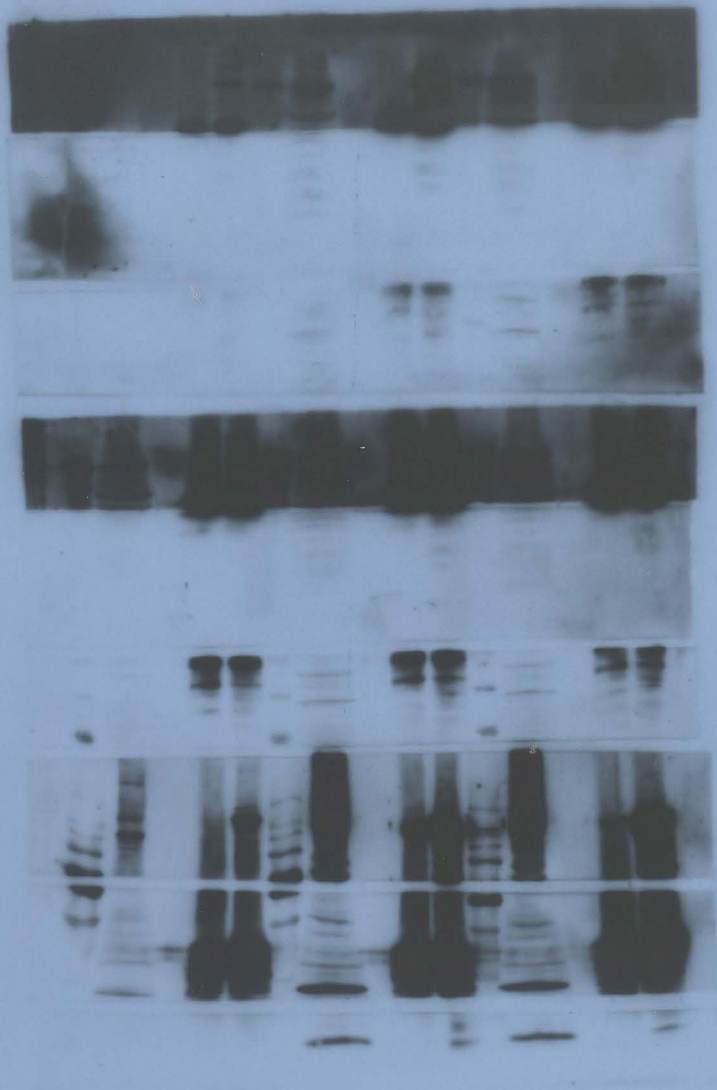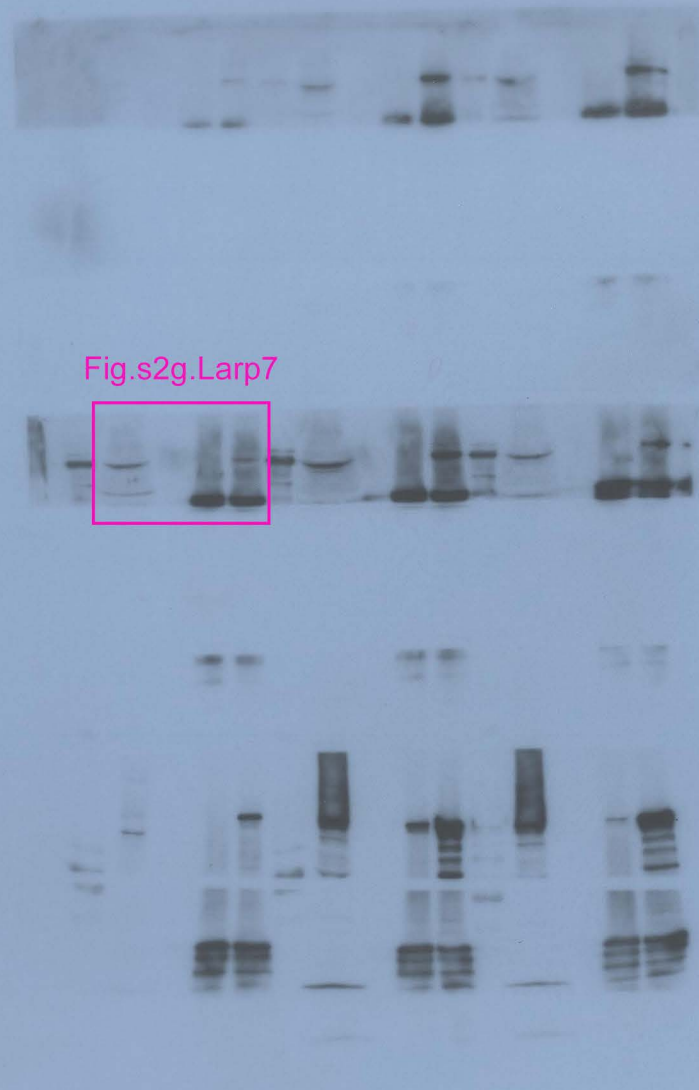

Fig.s2g.Larp7

Supplement: Supplementary file 8 — Source Data [file 41467_2021_21529_MOESM8_ESM.zip › Uncropped blot and gel images/FigureS2/FigureS2g/Larp7.pdf]

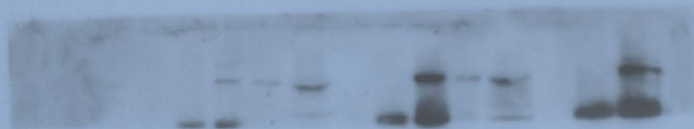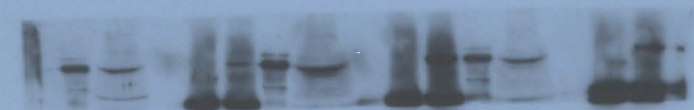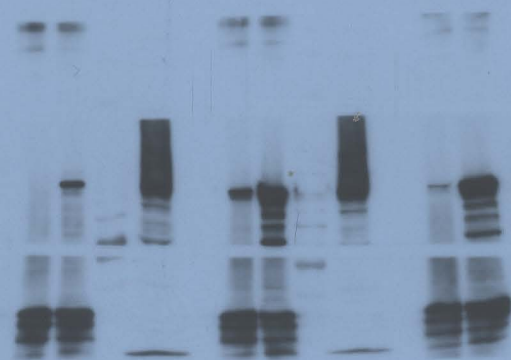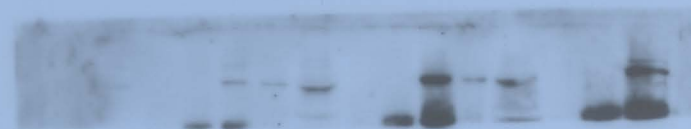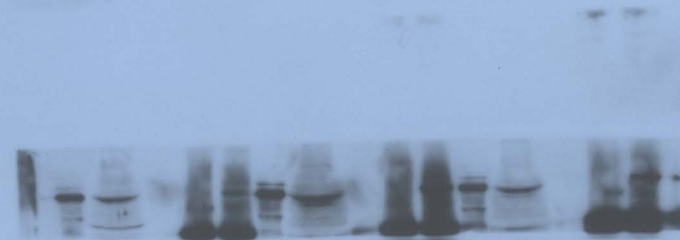

Fig.s2g.RHA

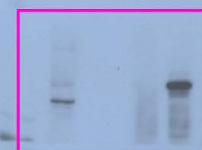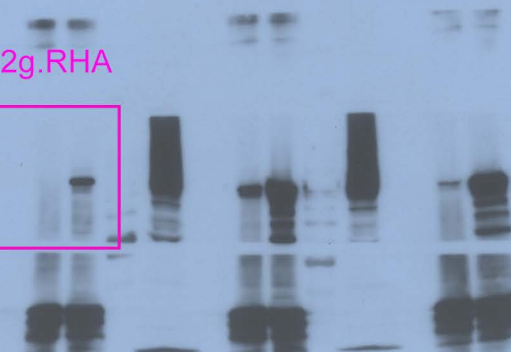

Supplement: Supplementary file 8 — Source Data [file 41467_2021_21529_MOESM8_ESM.zip › Uncropped blot and gel images/FigureS2/FigureS2g/RHA.pdf]

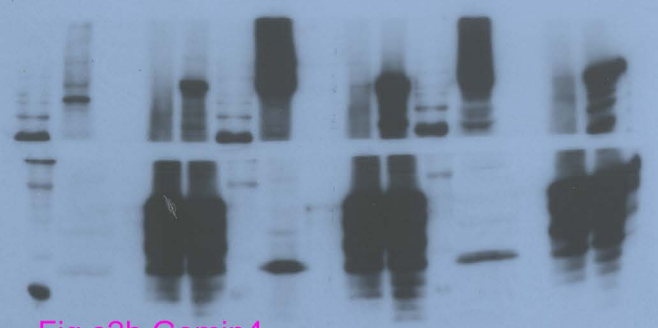

Fig.s2h.Gemin4

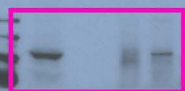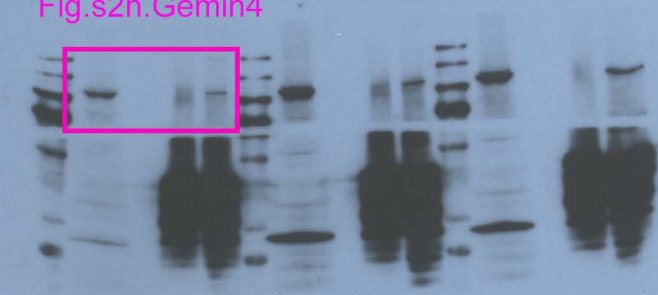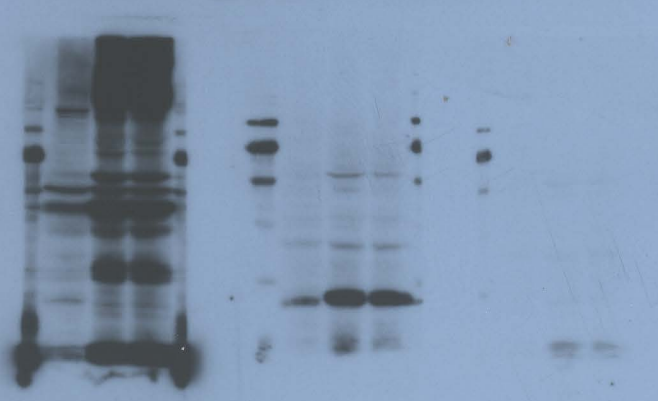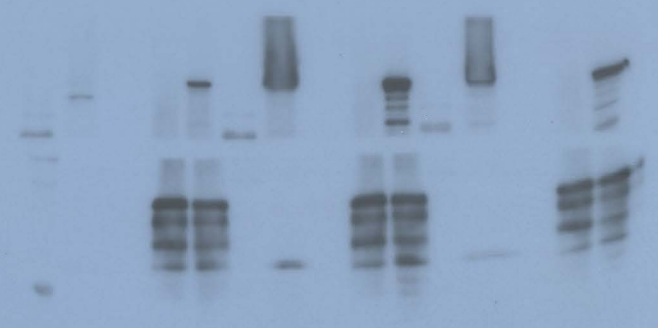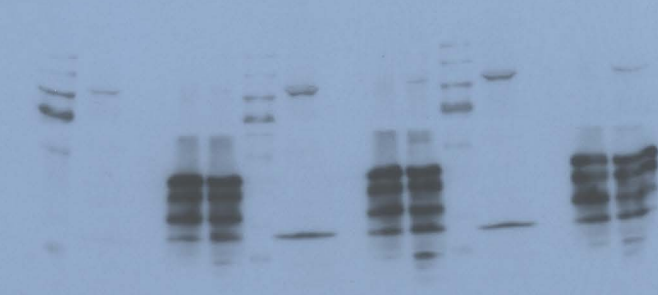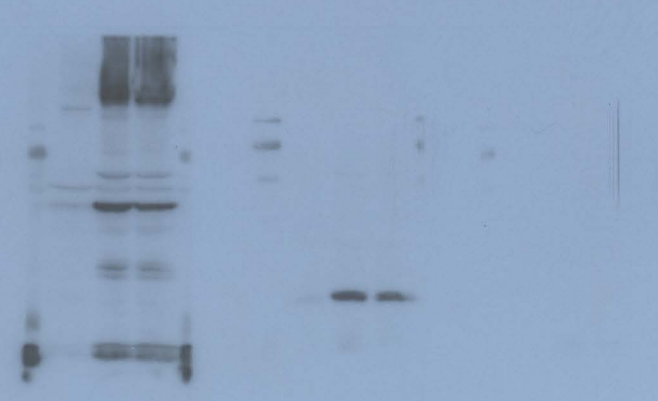

Supplement: Supplementary file 8 — Source Data [file 41467_2021_21529_MOESM8_ESM.zip › Uncropped blot and gel images/FigureS2/FigureS2h/Gemin4.pdf]

Fig.s2h.hnRNP Q

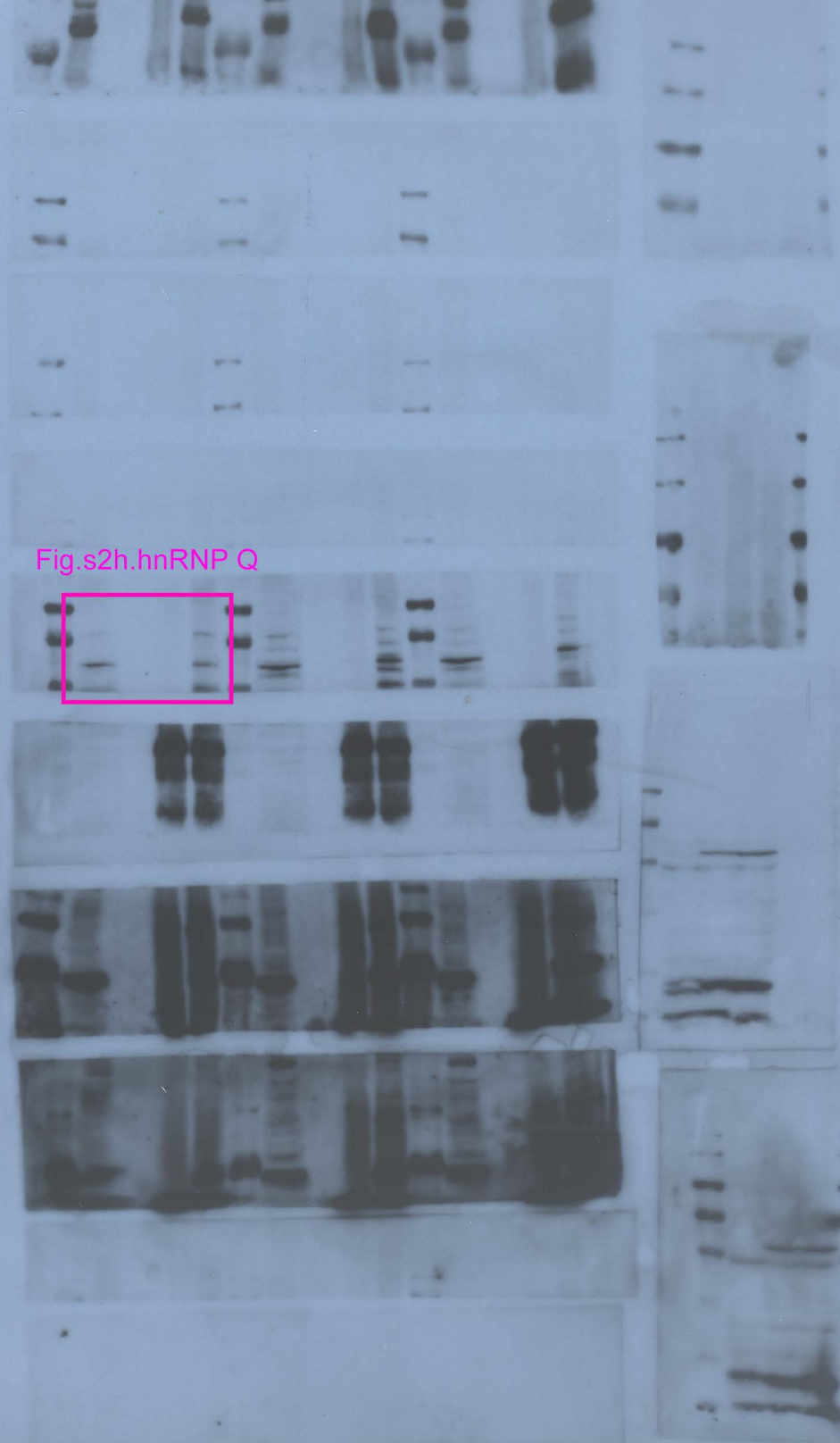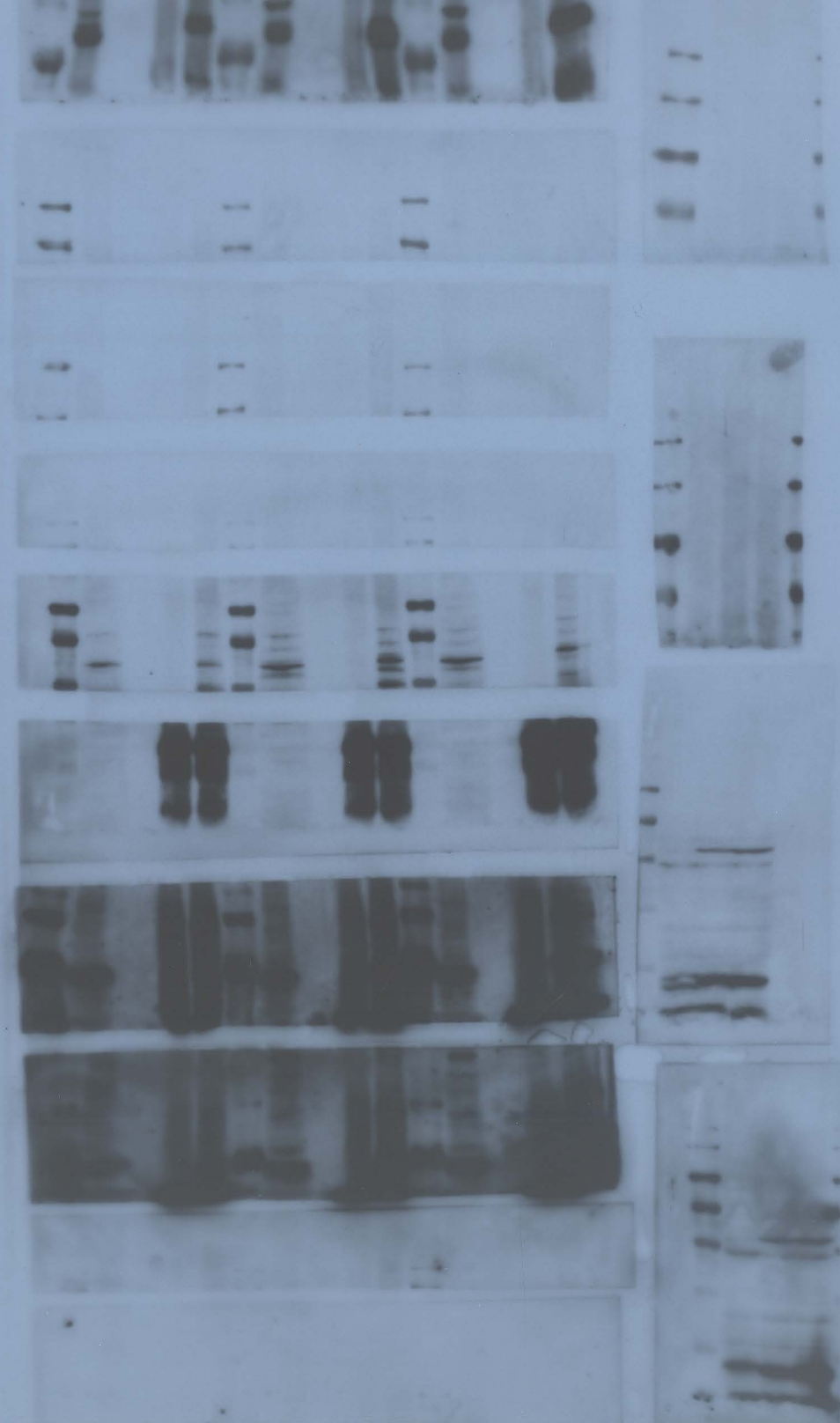

Supplement: Supplementary file 8 — Source Data [file 41467_2021_21529_MOESM8_ESM.zip › Uncropped blot and gel images/FigureS2/FigureS2h/hnRNP Q.pdf]
