# Supplementary material for: Interaction of 7SK with the Smn complex modulates snRNP production
Source: Nat Commun. 2021 Feb 24;12:1278. doi: 10.1038/s41467-021-21529-1 (PMC7904863; doi:10.1038/s41467-021-21529-1)

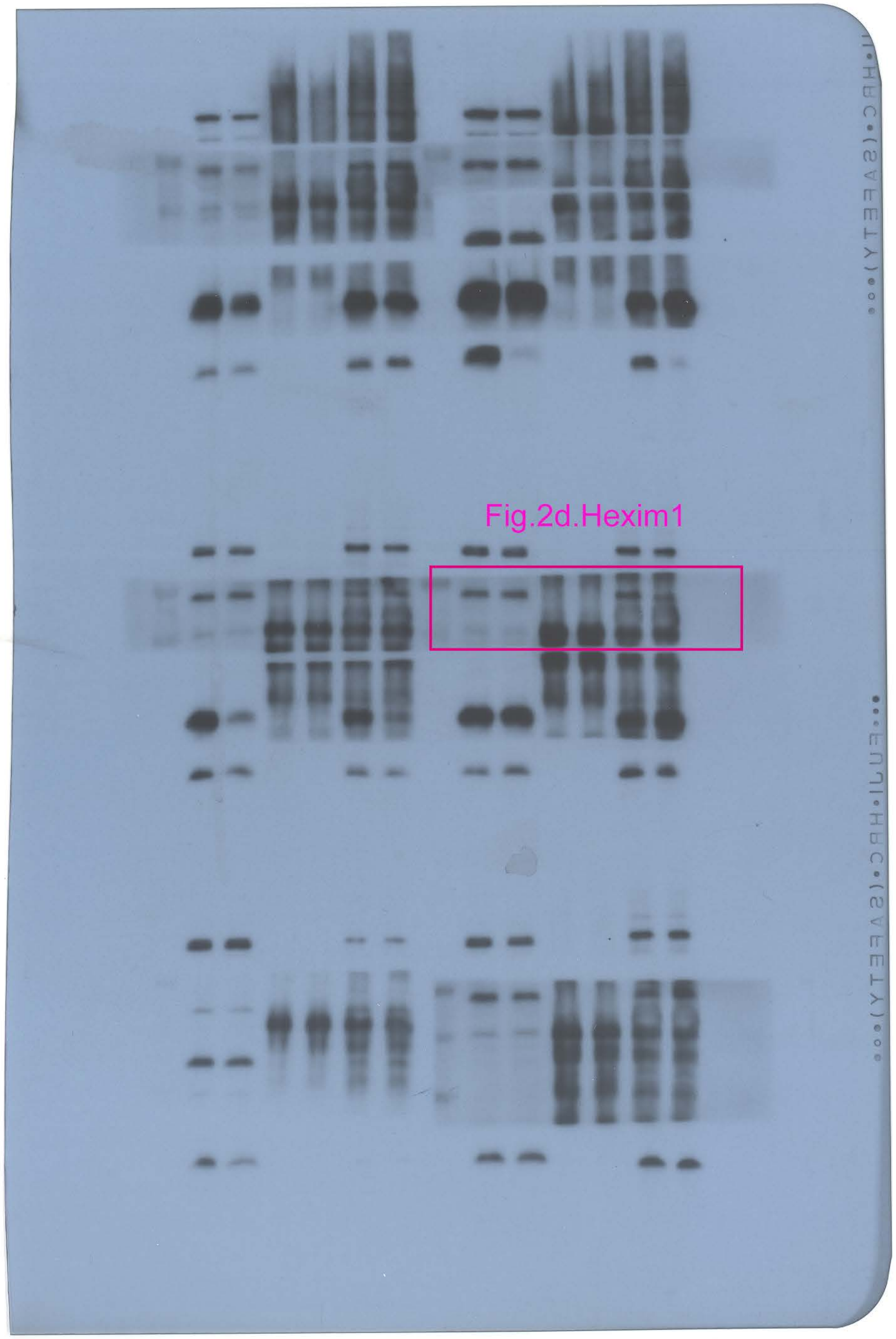
Fig.2d.Hexim1

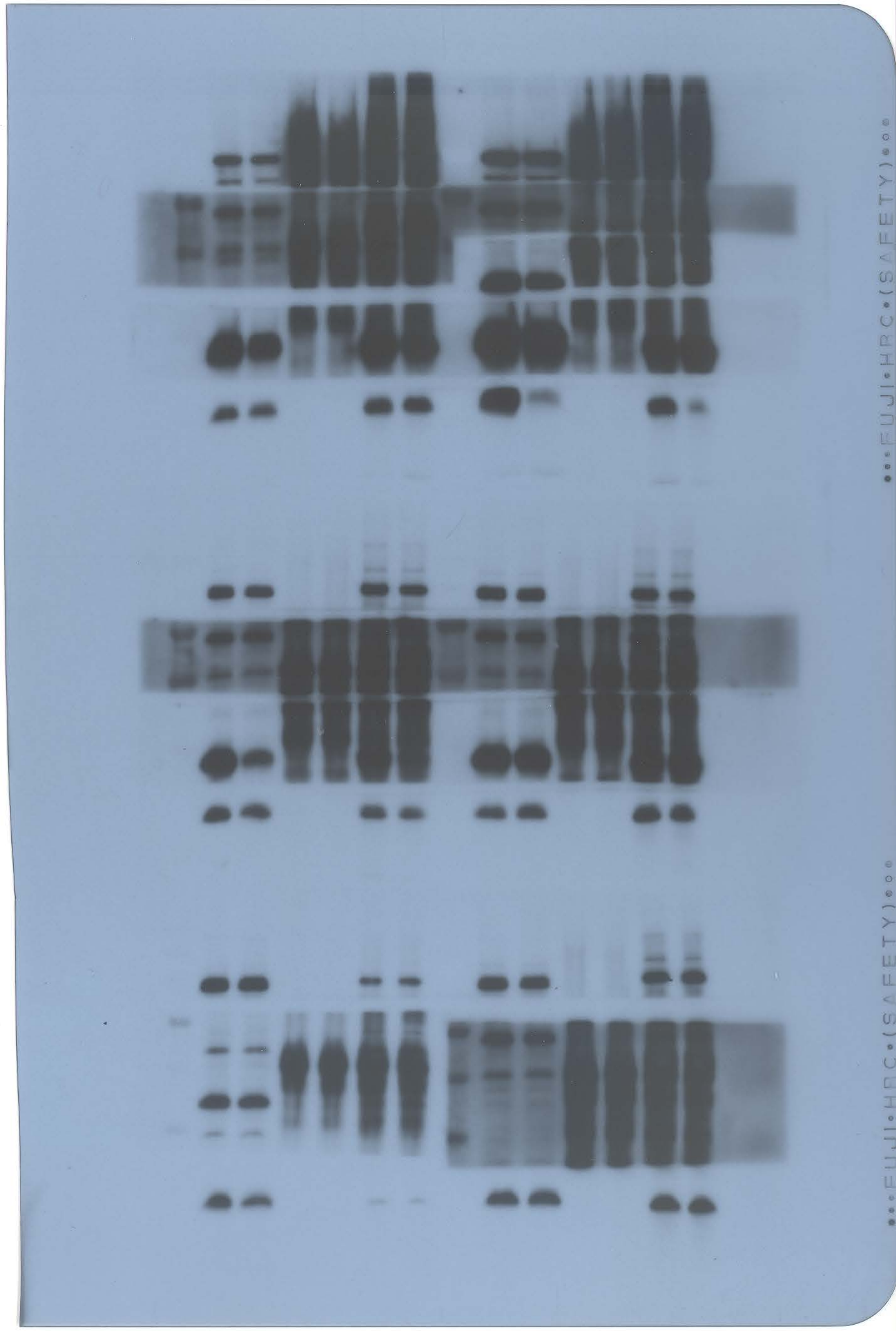

Supplement: Supplementary file 8 — Source Data [file Hexim1.pdf › Uncropped blot and gel images/Figure2/Figure2d/Hexim1.pdf]
